# Molecular landscape and multi-omic measurements of heterogeneity in fetal adenocarcinoma of the lung

**DOI:** 10.1038/s41698-024-00569-y

**Published:** 2024-06-03

**Authors:** Li Sun, Wei Guo, Lei Guo, Xiaoxi Chen, Haitao Zhou, Shi Yan, Gang Zhao, Hua Bao, Xue Wu, Yang Shao, Jianming Ying, Lin Lin

**Affiliations:** 1 Department of Pathology, National Cancer Center/National Clinical Research Center for Cancer/Cancer Hospital, Chinese Academy of Medical Sciences and Peking Union Medical College, Beijing, People’s Republic of China; 2 Department of Thoracic Surgery, National Cancer Center/National Clinical Research Center for Cancer/Cancer Hospital, Chinese Academy of Medical Sciences and Peking Union Medical College, Beijing, People’s Republic of China; 3 Key Laboratory of Minimally Invasive Therapy Research for Lung Cancer, Chinese Academy of Medical Sciences, Beijing, China; 4 Geneseeq Research Institute, Nanjing Geneseeq Technology Inc, Nanjing, China; 5 Department of Thoracic Surgery, Peking University Cancer Hospital and Institute, Beijing, China; 6 Department of Pathology, Tianjin Medical University Cancer Institute and Hospital, Tianjin, China; 7 School of Public Health, Nanjing Medical University, Nanjing, China; 8 Department of Medical Oncology, National Cancer Center/National Clinical Research Center for Cancer/Cancer Hospital, Chinese Academy of Medical Sciences and Peking Union Medical College, Beijing, People’s Republic of China

**Keywords:** Cancer genomics, Tumour heterogeneity, Non-small-cell lung cancer

## Abstract

Fetal adenocarcinoma of the lung (FLAC) is a rare form of lung adenocarcinoma and was divided into high-grade (H-FLAC) and low-grade (L-FLAC) subtypes. Despite the existence of some small case series studies, a comprehensive multi-omics study of FLAC has yet to be undertaken. In this study, we depicted the multi-omics landscapes of this rare lung cancer type by performing multi-regional sampling on 20 FLAC cases. A comparison of multi-omics profiles revealed significant differences between H-FLAC and L-FLAC in a multi-omic landscape. Two subtypes also showed distinct relationships between multi-layer intratumor heterogeneity (ITH). We discovered that a lower genetic ITH was significantly associated with worse recurrence-free survival and overall survival in FLAC patients, whereas higher methylation ITH in H-FLAC patients suggested a short survival. Our findings highlight the complex interplay between genetic and transcriptional heterogeneity in FLAC and suggest that different types of ITH may have distinct implications for patient prognosis.

## Introduction

Fetal adenocarcinoma of the lung (FLAC) is a rare form of adenocarcinoma of lung. FLACs were divided into high-grade fetal adenocarcinomas (H-FLAC) and low-grade fetal adenocarcinomas (L-FLAC) based on their clinicopathological features and biological behaviors^1^. The prevalence of FLAC calculated by a previous study screened 920 lung adenocarcinomas is 0.87%, including 0.54% H-FLAC and 0.32% L-FLAC. L-FLAC has a pure pattern, displaying low nuclear atypia and prominent morule formation. H-FLAC is generally composed of at least 50% fetal morphology and other conventional types of lung adenocarcinoma. H-FLAC occurs more frequently in elderly male patients with a heavy smoking history^2^ whereas L-FLAC tends to occur in young female non-smokers^3^. Patients with L-FLAC usually present with stage I–II disease, with favorable outcomes^3^. Whereas patients with H-FLAC were often at an advanced-stage disease (stage III–IV) at presentation, with a worse prognosis than L-FLAC and conventional lung adenocarcinoma^4^. However, much of the knowledge regarding FLAC is from case reports or small case series^2,3,5,6^ and there were only a few comprehensive studies of FLAC.

Several molecular studies have reported that KRAS, EGFR, and PIK3CA mutation share very low rates in FLAC^7^. Furthermore, L-FLAC was found to constantly exhibit aberrant β-catenin nuclear/cytoplasmic expression and frequent β-catenin gene mutations. On the contrary, H-FLAC demonstrates nuclear p53 overexpression and mutations^5^. The molecular features of FLAC, especially its epigenetic and transcriptional features have not yet been clarified.

Genetic heterogeneity, measured by the number of clones and sub-clonal genetic alterations, was found to predict poor survival in multiple cancer types^8,9^. Similarly, patterns of epigenetic and transcriptional heterogeneity appear to be associated with tumor subtyping and risk of recurrence^10–12^. Several studies have analyzed the extent of intratumor heterogeneity (ITH) of somatic nucleotide variants (SNV), copy number variants (CNV) and DNA methylation in lung adenocarcinomas^9,10,13,14^. Furthermore, a previous study investigating Intratumor heterogeneity patterns in lung squamous cell carcinoma suggests that ITH at different levels synergistically impacted tumor histological features and a multi-level assessment of heterogeneity is necessary to identify the determinants of phenotypic heterogeneity in clinically relevant characteristics^15^. Therefore, an integration analysis of multi-omics ITH may provide valuable information about the pathogenesis and prognosis of FLAC.

In this study, we sought to provide further information on the molecular features of FLAC by analyzing whole‐exome sequencing, DNA methylation, and RNA‐sequencing profiles of paired tumor and normal lung tissue samples from FLAC patients. Moreover, multiregional sequencing has been the most successful strategy to investigate intratumor heterogeneity and clonal evolution in multiple cancer types to date^16–18^. Thus, we also sought to investigate intratumor heterogeneity at genomic, epigenomic and transcriptomic levels based on multiregional sequencing to better understand the complex heterogeneity and identify the determinants of phenotypic heterogeneity and prognosis.

## Results

### Patient characteristics

A total of twenty patients with FLAC were included in the study after being filtered through sample quality control. Twelve of the patients (60%) were high-grade FLAC (H-FLAC) and eight were low-grade FLAC (L-FLAC). Of the 12 H-FLAC cases, 4 cases consisted of H-FLAC in a pure form (pure H-FLAC) and the remaining 8 cases showed H-FLAC combined with other histologies (mixed H-FLAC). The median age of the patients was 64 years (75% males) in H-FLAC cases, and 51 years (75% males) in L-FLAC cases (Table 1). Most of the patients were of early-stage disease (Stage I: 14/20, 70%). Six patients received adjuvant therapy and five patients developed metastatic disease (Supplementary data 1).

**Table 1:** Patient clinical characteristics

Characteristic	All (*n* = 20)	H-FLAC (*n* = 12)	L-FLAC (*n* = 8)
*Age (years)*			
Median (range)	62.5 (31–81)	64 (35–73)	51 (31–81)
*Sex, n (%)*			
Female	5 (25%)	3 (25%)	2 (25%)
Male	15 (75%)	9 (75%)	6 (75%)
*Smoking status, n (%)*			
Smoker	15 (75%)	9 (75%)	6 (75%)
Non-smoker	5 (25%)	3 (25%)	2 (25%)
*T Stage, n (%)*			
T1	9 (45%)	5 (42%)	4 (50%)
T2	6 (30%)	4 (33%)	2 (25%)
T3	2 (10%)	0 (0%)	2 (25%)
T4	3 (15%)	3 (25%)	0 (0%)
*N Stage, n (%)*			
N0	18 (90%)	10 (84%)	8 (100%)
N1	1 (5%)	1 (8%)	0 (0%)
N2	1 (5%)	1 (8%)	0 (0%)
*Stage, n (%)*			
I	14 (70%)	8 (67%)	6 (75%)
II	3 (15%)	1 (8%)	2 (25%)
III	3 (15%)	3 (25%)	0 (0%)
*Tumor Size (cm), n (%)*			
<3	9 (45%)	6 (50%)	3 (38%)
≥3	11 (55%)	6 (50%)	5 (62%)

### Genomic landscape and mutational signature of FLAC

**Fig. 1:**
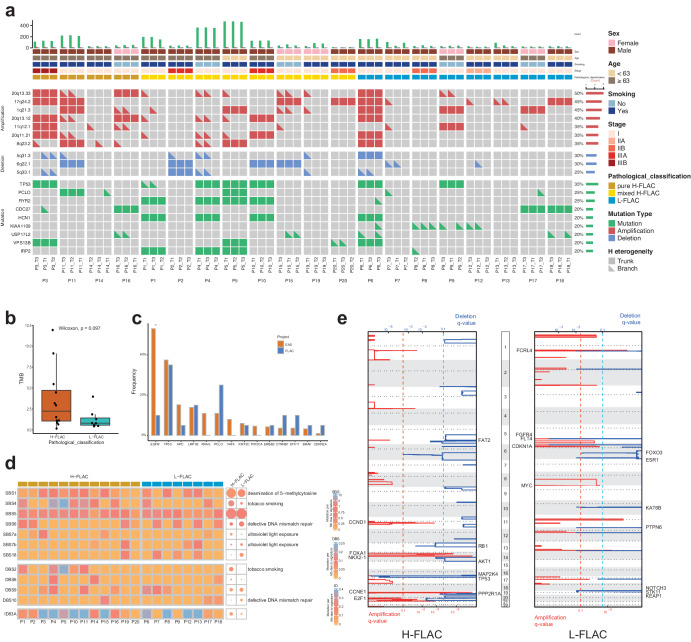
Genomic landscape and mutational signature. **a** Mutational landscape of FLAC (*n* = 20). Alteration frequencies are calculated per patient. **b** The box plot showed the comparison of tumor mutational Burdon (TMB) between H-FLAC and L-FLAC patients. The *p* value is calculated using Wilcoxon rank-sum test. Center line, median; box limits, upper and lower quartiles; whiskers, 1.5x interquartile range. **c** Comparison of mutated gene frequency between FLAC and EAC cohort. The *p* value is calculated using Fisher’s exact test. *p < 0.05; **p < 0.01; ***p < 0.001. **d** COSMIC mutational signatures inferred from somatic base changes in FLAC. The color of each cell or dot represents the median mutation burden of the signature in sample(s) that show the signature. The size of each dot represents the proportion of samples of each subtype that shows the mutational signature. SBS: Single Base Substitution signatures; DBS: Doublet base substitutions signatures. ID: small Insertions and Deletions signatures. **e** Recurrent somatic copy-number variations in H-FLAC and L-FLAC. Amplifications and deletions are plotted in red and blue, respectively.

Across all patients, we identified an exonic mutation rate of 2.6 mutations per megabase on average. The somatic mutations detected by WES are listed in Supplementary Data 2. TP53 was the most frequently mutated gene (Fig. 1a) in all FLAC patients (7/20, 35%) and H-FLAC patients (5/12, 42%), whereas BLTP1 was most frequently mutated in L-FLAC patients (4/8, 50%). Consistent with previous reports, the frequency of TP53 mutations was higher in mixed H-FLAC patients (42%, 5/12) compared to that in pure H-FLAC (25%, 1/4) and L-FLAC (25%, 2/8; Supplementary Fig. 1). The mean tumor mutation burden (TMB) was 3.6 mutations/Mb (range: 0.2–11.9) for H-FLAC, which is slightly higher than that of L-FLAC (1.3 mutations/Mb; range: 0.4–4.0; *P* = 0.097; Fig. 1b; Wilcoxon rank-sum test). We also compared our cohort with a conventional East Asian-ancestry (EAS) lung adenocarcinoma (LUAD)^19^. Only 2 patients (10%) in the FLAC cohort harbored EGFR mutations, which is significantly lower than the frequency in the East Asian cohort (53%, 160/302; Fig. 1c).

To explore the underlying mutagenic driving factors, we used a nonnegative matrix factorization algorithm (SigProfiler)^20^ on FLAC samples to extract potential mutational signatures. We identified four mutational signatures through the de novo extraction, including two Single Base Substitution (SBS) signatures, one Doublet base substitution (DBS) signatures and one small Insertion and Deletion (ID) signatures (Supplementary Table 1). By comparing de novo extracted signatures with COSMIC reference signatures (v3.3, June 2022), we identified 7 COSMIC reference SBS signatures and 4 DBS signatures (Fig. 1d; Supplementary Fig. 2). SBS1 and SBS5 were enriched in the whole cohort and were previously reported to be correlated with age in multiple types of cancer^21^. Two Tobacco-associated signatures, SBS4 and DBS2, were both found enriched in H-FLAC patients compared to L-FLAC, suggesting an association between H-FLAC and smoking status. Whereas SBS6 and DBS10, which were associated with defective DNA mismatch repair, were more enriched in L-FLAC patients.

We identified significantly altered copy number variations (CNVs) using Genome Identification of Significant Targets in Cancer (GISTIC) 2.0 algorithm^22^ (*q* < 0.1; Fig. 1e; Supplementary Data 3; Supplementary Fig. 3). The most significant arm level amplifications were 14q13.3 (FOXA1, NKX2-1), 14q23.1 and 19q12 (CCNE1) in H-FLAC patients and 1q21.3 (FCRL4), 11p11.11 and 13q34 in L-FLAC patients, while the most significant arm level deletions were 17p12(MAP2K4) and 17p13.2 (TP53) in H-FLAC patients and 5q31.3 and 6p22.2 in L-FLAC patients. The significantly amplified driver gene EGFR, KRAS, and ERRB2 as well as deleterious driver gene APC in conventional EAS LUAD were not identified in both H-FLAC and L-FLAC groups, while MYC amplification and STK11 deletion were identified in L-FLAC patients and amplification around CCNE1, NKX2-1 were found in H-FLAC patients.

### DNA methylation and gene expression pattern

**Fig. 2:**
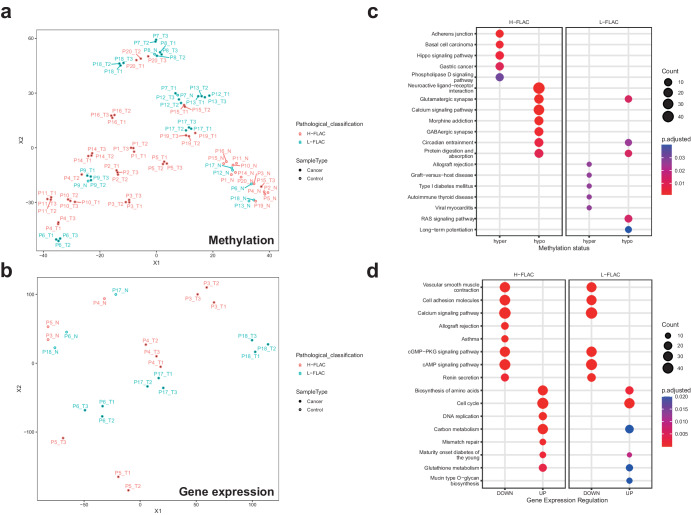
DNA methylation and gene expression pattern. **a**, **b** t-distributed stochastic neighbor embedding (t-SNE) plot showing the extent of epigenomic (**a**) and transcriptomic (**b**) variation within and across tumor and non-malignant tissue regions from the patients. Non-malignant tissues from different patients are shown in hollow circles, whereas tumor tissues from different patients are shown in filled circles. H-FLAC and L-FLAC samples are shown in different colors. **c** Pathway enrichment comparison between H-FLAC and L-FLAC based on differentially methylation region (DMR) results. **d** Pathway enrichment comparison between H-FLAC and L-FLAC based on differentially expressed gene (DEG) results.

The unsupervised t-distributed stochastic neighbor embedding (t-SNE) analysis and hierarchical clustering of normalized methylation levels across all samples (Fig. 2a; Supplementary Fig. 4a) and normalized gene expression across six samples (Fig. 2b; Supplementary Fig. 4b) showed that non-malignant tissues clustered together, suggesting a low level of between-sample variation, as expected in non-diseased tissue specimens. Furthermore, different regions from the same tumors typically cluster together, suggesting that intra-tumor methylation variations were typically smaller than inter-individual differences. Nevertheless, H-FLAC and L-FALC samples were not separately clustered by either methylation levels or gene expression.

We next turned to the epigenetic and transcriptomic landscape of H-FLAC and L-FLAC. We compared the methylation levels and gene expression of tumors samples against match normal tissue samples to identify differentially methylation regions (DMR) and differentially expressed genes (DEG) for both H-FLAC and L-FLAC cohort (Fig. 2c-d; Supplementary Data 4). No gene in DMRs of H-FLAC and L-FLAC exhibited opposite directions while only two genes exhibited opposite directions in DEG analysis of the two groups (Supplementary Fig. 5A-B). Up-regulation of genes related to cell cycle was revealed in both H-FLAC and L-FLAC patients whereas cell adhesion, cAMP signaling pathway, cGMP−PKG signaling pathway and calcium signaling pathway were down-regulated in both groups. For H-FLAC samples, DMR and DEG analysis revealed significant hypomethylation of genes related to neuroactive ligand−receptor interaction and up-regulation of genes related to DNA replication, mismatch repair and cell cycle. For L-FLAC patients, RAS signaling pathway were found to be hypermethylated. Nevertheless, calcium signaling pathway was found down-regulated by DEG analysis but hypomethylated by DMR analysis, suggesting a more diverse mechanism of epigenetic regulation in H-FLAC. The same trend was observed when we compared the pathway enrichment results from samples for which both transcriptomic and methylation data were available (Supplementary Fig. 6). Furthermore, the pure H-FLAC displayed hypermethylation in the Ras and MAPK signaling pathways, Proteoglycans in cancer pathway, as well as both hypermethylation and hypomethylation in the Rap1 signaling pathway and neuroactive ligand-receptor interaction (Supplementary Fig. 7).

Overall, pathway enrichment analysis on DMR and DEG of the two groups revealed that H-FLAC and L-FLAC had a significant difference in epigenomic landscape but resembled each other on the transcriptomic landscape.

### Heterogeneity analysis of genomic alterations

**Fig. 3:**
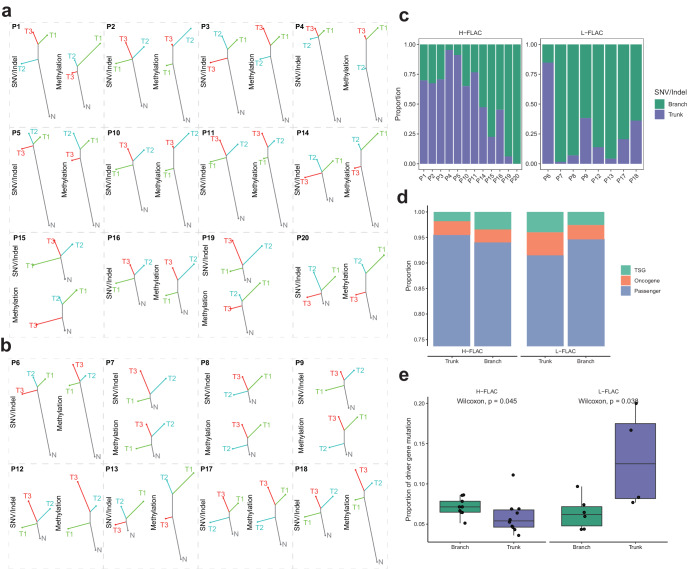
Heterogeneity of genomic alterations. **a**, **b** Phylogenetic tree sand phyloepigenetic trees of H-FLAC (**a**) and L-FLAC (**b**) based on somatic mutations and DNA methylation profiles, respectively. N: normal tissue sample. **c** The proportion of trunk/branch mutations in each patient based on phylogenetic trees. **d** The proportions of putative driver mutations (oncogenes and tumor suppressor genes) versus passenger mutations on the trunks and branches of all H-FLAC or L-FLAC patients. **e** Comparison of the proportions of putative driver mutations (oncogenes and tumor suppressor genes) on the trunks and branches per patients. The *p* value is calculated using Wilcoxon rank-sum test. Center line, median; box limits, upper and lower quartiles; whiskers, 1.5x interquartile range.

To study spatial heterogeneity of genomic alterations, we leveraged the multi-region WES data and performed phylogenetic analysis. The genomic phylogenetic trees were constructed with all somatic mutations (Fig. 3a, b). Lengths of trunks and branches were proportional to the number of detected mutations. We define a somatic genetic alteration shared by all regions as a trunk mutation; otherwise, it was defined as branch mutation. Trunk mutations were dominant in H-FLAC patients (median: 66.3%; 95%CI: 31.8–74.4%) while branch mutations were dominant in L-FLAC patients (median: 82.8%; 95%CI: 48.1–99.8%; Fig. 3c). Proportions of trunk mutations in H-FLAC tend to be higher than those in L-FLAC (*P* = 0.069; Wilcoxon rank-sum test; Supplementary Fig. 8). Passenger mutations were enriched in the branch for both groups, which contributed more to the intra‐tumor heterogeneity (Fig. 3d). Of note, although the proportion of trunk mutations was significantly less than branch mutations in L-FLAC patients, driver gene mutations (oncogene and tumor-suppression gene) were significantly enriched in trunk mutations (*P* = 0.038; Wilcoxon rank-sum test; Fig. 3e).

### Multi-layer analysis of intra-tumor heterogeneity

To study the spatial-temporal genetic, epigenetic, and transcriptional heterogeneity of FLAC, we performed phylogenetic tree analysis on somatic mutations, methylation data and gene expression data, respectively (Fig. 2a, b; Supplementary Fig. 9). Somatic mutation and DNA methylation changes appeared to follow similar patterns, as suggested by the overall structure of most methylation-based phyloepigenetic trees (14/20) aligning with that of phylogenetic trees based on mutations. However, several samples showed notable differences on length of edges (distance between the nodes) between phylogenetic and phylo-epigenetic trees, suggesting an inconsistency of multi-level intratumor heterogeneity.

**Fig. 4:**
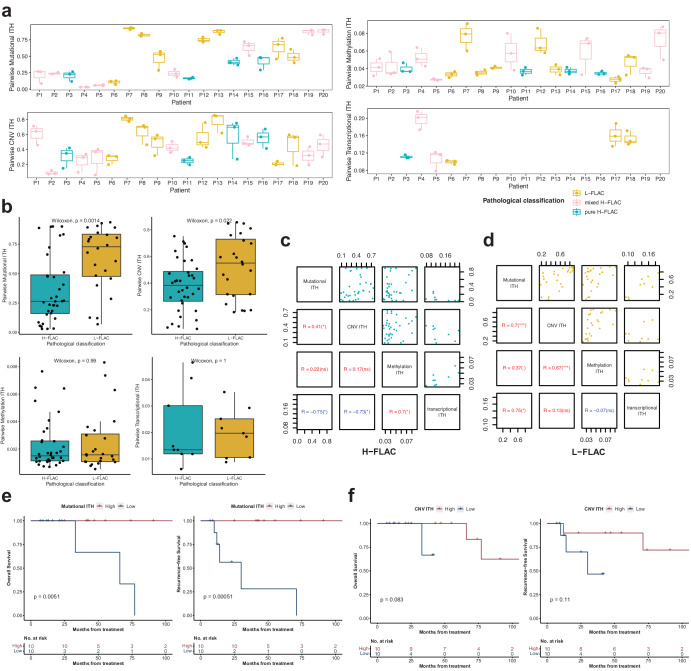
Multi-layer intra-tumor heterogeneity. **a** Pairwise mutational, CNV, methylation and transcriptional intratumor heterogeneity (ITH) of each patient. **b** Comparison of pairwise mutational, CNV, methylation and transcriptional ITH between H-FLAC and L-FLAC patients. The *p* value is calculated using Wilcoxon rank-sum test. Center line, median; box limits, upper and lower quartiles; whiskers, 1.5x interquartile range. **c**, **d** Correlations between pairwise mutational, CNV, methylation and transcriptional ITH in H-FLAC (**c**) and L-FLAC (**d**), assessed by two-tailed Spearman’s correlation analysis. Each dot represents each pairwise comparison of regions within each tumor. ns: not significant; ^•^*p* < 0.1; *p < 0.05; **p < 0.01; ***p < 0.001. **e**, **f** Kaplan–Meier curve of recurrence-free survival (RFS) and overall survival (OS) in patients (*n* = 20) stratified by median mutational ITH (**e**) and CNV ITH (**f**).

We proceeded to evaluate the degree of intratumor heterogeneity (ITH) within each tumor by computing pairwise scores for mutational ITH, CNV ITH, methylation ITH, and transcriptional ITH based on the distances between regions within the tumor. (Fig. 4a; Methods). Consistent with the higher proportion of trunk mutations in H-FLAC, pairwise intratumor mutational and CNV ITH of H-FLAC were significantly lower than those of L-FLAC (*P* = 0.001 and *P* = 0.022, respectively; Fig. 4b). However, there were no significant differences in methylation ITH and transcriptional ITH between the two groups (*P* = 0.99 and *P* = 1, respectively). Furthermore, the analyses of pure H-FLAC and mixed H-FLAC patients did not show any differences in any of the four levels of ITH (Supplementary Fig. 10).

Pairwise mutational ITH and CNV ITH showed a positive correlation in both H-FLAC and L-FLAC samples (*P* = 0.01 and *P* = 0.001, respectively; Fig. 4c, d; Supplementary Fig. 10), which indicated the consistency within genetic heterogeneity. Of note, in L-FLAC samples, pairwise genetic ITH was significantly positively correlated with epigenetic ITH and transcriptional ITH (*P* = 0.02 for mutational vs. transcriptional ITH; *P* = 0.003 for CNV vs methylation ITH), which was in line with the positive correlation of genetic and transcriptomic heterogeneity in lung squamous cell carcinoma^15^. However, in H-FLAC samples, although methylation ITH was positively correlated with transcriptional ITH, the mutational and CNV ITH showed a significantly negative correlation with transcriptional ITH (*P* = 0.02 and. *P* = 0.025, respectively), which indicated an opposite genetic heterogeneity and transcriptional heterogeneity of H-FLAC.

Previous studies showed that high genomic intratumor heterogeneity was associated with worse overall OS in lung adenocarcinoma^23^ and small cell lung cancer^24^. However, L-FLAC patients who had higher genetic ITH compared to H-FLAC (Fig. 4b), did not suffer the event of interest and showed significantly longer OS and RFS (*p* = 0.013 and *p* = 0.0097, respectively; Supplementary Fig. 11). We further divided FLAC patients into high and low ITH groups based on the median of average mutational, CNV or methylation ITH and examined the associations between ITH and survival. Patients with high mutational ITH showed significantly better OS and RFS (median OS: not reached vs 66 months, *P* = 0.005; median RFS: not reached vs 30 months, *P* = 0.0005; Fig. 4e). Patients with high CNV ITH exhibited a non-significant trend towards improved OS and RFS (*P* = 0.083 and *P* = 0.11, respectively; Fig. 4f). Despite the limited sample size, both subgroups exhibited a trend towards poorer overall survival (OS) and recurrence-free survival (RFS) in patients with genetic ITH, although the results were not statistically significant (Supplementary Figs. 12a, b and 13a, b). Additionally, a multivariate Cox regression analysis of FLAC patients revealed the level of mutational ITH to be a significant factor, after adjusting for stage and pathological classification (Supplementary Table 2). Nevertheless, patients with different methylation ITH did not show a significant difference on survival (Supplementary Fig. 14). High methylation ITH patients in H-FLAC had a median RFS of 30 months and a median OS of 33 months, compared to a median RFS of 71 months and a median OS of 71.5 months for all patients combined (*P* = 0.22 and *P* = 0.71, respectively; Supplementary Fig. 12c).

Due to the limited availability of transcriptomic data in our study, we are unable to draw definitive conclusions about the relationship between transcriptional ITH and survival. However, the significant negative correlation observed between genetic ITH and transcriptional ITH suggests a high transcriptional ITH in high-grade or low-genetic-ITH FLAC patients. Therefore, transcriptional ITH may contribute to the worse prognosis observed in H-FLAC patients. In conclusion, our findings highlight the complex interplay between genetic and transcriptional heterogeneity in FLAC and suggest that different types of ITH may have distinct implications for patient prognosis. Further research is needed to fully elucidate these relationships and their clinical implications.

## Discussion

In this study, we performed multi-omics profiling on FLAC, a rare lung adenocarcinoma subtype and depicted the genomic, epigenomic and transcriptomic landscape of FLAC. We also compared FLAC and conventional lung adenocarcinoma and between two subgroups of FLAC, H-FLAC and L-FLAC. Moreover, by multi-region sampling of the tumor samples, we further investigated genetic, epigenetic and transcriptional intratumor heterogeneity in H-FLAC and L-FLAC samples. Although genetic and epigenetic changes showed congruent evolutionary trajectories, the relationships between multi-level ITH were distinct in H-FLAC and L-FLAC. Different levels of ITH showed different relationship with recurrence-free survival and overall survival, and non-genetic ITH, instead of genetic ITH, may contribute to the prognosis in FLAC patients.

In line with the previous reports^7^, no KRAS and PIK3CA mutations were identified in our cohort, and the frequency of EGFR mutations was significantly lower than that of conventional EAS LUAD. Several previous studies showed that CTNNB1 and DICER1 were recurrently mutated in L-FLAC patients^25,26^. However, in our cohort, we only observed CTNNB1 mutations in two patients, P2 (H-FLAC) and P13 (L-FLAC) and DICER1 mutations in two patients, P18 (L-FLAC; p.A752Qfs*6; likely pathogenic) and P19 (H-FLAC; p.K1756N). Moreover, although we identified BLTP1 as the most frequently mutated gene in L-FLAC patients, we must caution that most of these mutations were branch mutations. The implications of these mutations for the pathogenicity of L-FLAC remain uncertain. Previous immunohistochemical results also showed positivity for neuroendocrine markers chromogranin A, synaptophysin and thyroid transcription factor 1 (TTF-1) in FLAC patients^2,7,26^. In this study, we also observed significant enrichment in glutamatergic synapse and/or GABAergic synapse of hypomethylated genes in H-FLAC and L-FLAC groups. Furthermore, NKX2-1 gene, which encodes TTF-1 was also found amplificated in H-FLAC patients.

The differences between H-FLAC and L-FALC described in previous studies were mostly in histopathological, immunohistochemical and genetic aspects^5,25^. In this study, we first compared these two subtypes in a multi-omics and heterogeneity aspect. Using whole-exome sequencing, we observed the most frequently mutated genes in H-FLAC and L-FLAC were TP53 and BLTP1, respectively. TMB were higher in H-FLAC which did not reach statistical significance. Focal-level CNV analysis also identified considerable differences between the conventional H-FLAC patients and L-FLAC patients as no overlapping focal-level CNV region was identified in H-FLAC and L-FLAC. Mutational signature analysis showed that tobacco-associated signatures were more enriched in H-FLAC, which is consistent with the heavy smoking exposure characteristic feature of H-FLAC in the previous study^2^. In contrast, L-FLAC observed more signatures associated with defective DNA mismatch repair. Moreover, differential expression analysis and differential methylation analysis showed a significant amount of overlapping up-regulated/hypomethylated and down-regulated/hypermethylated genes in H-FLAC and L-FLAC groups, which resulted in a significant amount of co-enriched pathway of these two groups identified by pathway enrichment analysis. Other than that, H-FLAC exhibited enrichment in Hippo signaling pathway (hypomethylated), DNA replication and mismatch repair (up-regulated) whereas L-FLAC showed enrichment of RAS signaling pathway (hypomethylated). This shared transcriptomic landscape of the two groups suggests that although H-FLAC and L-FLAC differed significantly at genomic level, they were much more akin at epigenomic and transcriptomics levels.

L-FLAC has a pure pattern^27^, whereas H-FLAC can be composed of miscellaneous histological components (Supplementary Fig. 15). However, multi-regional ITH analysis revealed that H-FLAC tumors shared a higher proportion of trunk mutations than L-FLAC and the genetic ITH was much lower, without regard to whether the H-FLAC was mixed or pure. This result is in agreement with a previous study that showed that FLL genetic profile largely overlapped with that of the CLA component in mixed H-FLAC^25^. This discrepancy between genetic and histological level heterogeneity may due to non-genetic sources of variation^28^. A previous study on the invasive micropapillary carcinoma (IMPC) of the breast showed that morphological heterogeneity responds to specific gene expression profiles rather than the presence of specific chromosomal aberrations. Sharma et al. analyzed genetic and non-genetic ITH in lung squamous cell carcinoma and found that non-genetic heterogeneity at immune and transcriptomic levels is a major determinant of heterogeneity in histopathological characteristics^15^. In this study, we also noticed an opposite correlative relationship between genetic ITH and transcriptional ITH in H-FLAC and L-FLAC patients. Although the transcriptional ITH of H-FLAC was not significantly higher than that of L-FLAC which may be due to the limited number of samples, the negative correlation between transcriptional ITH and genetic ITH in H-FLAC suggested that transcriptional ITH may contribute to the morphological heterogeneity of H-FLAC. Furthermore, although it is more likely that intratumor heterogeneity is contributed to passenger gene mutations, the proportions of driver gene mutations in branch mutations were significantly higher than those in trunk mutations, which may also contribute to the transcriptional heterogeneity and morphological heterogeneity in H-FALC.

High intratumor genetic heterogeneity was reported to be related to worse outcomes in multiple cancer types^9,23,24,29^. However, in this study, we observed that low intratumor genetic heterogeneity was related to worse recurrence-free survival and overall survival in H-FLAC patients. Nevertheless, we observed that H-FLAC, despite having a lower proportion of branch genes indicating lower genetic intratumor heterogeneity, exhibited a significant enrichment of driver gene mutations in branch mutations. It is plausible that these driver gene mutations contribute to the transcriptional diversity of the tumor, enabling it to adapt to various pressures such as treatment, thereby leading to a poorer prognosis. Furthermore, all cases with the event of interest were metastasis. A previous study showed that intratumor morphological heterogeneity was significantly associated with the rate of breast cancer metastasis^30^. Patel et al. found that increased subtype heterogeneity was associated with decreased survival^31^. Therefore, taken together with the negative correlation between genetic ITH and transcriptional ITH in H-FALC patients, we hypothesized that non-genetic ITH, which may result in high morphological heterogeneity and contribute to the worse prognosis of H-FLAC patients.

This study has several limitations. FLAC is a rare type of cancer, and it took us 9 years (from January 2013 to August 2021) to collect these 20 cases qualified for our analyses. Specimens may have different levels of degradation during the period. Furthermore, due to the increasing quality requirement of RNA sequencing^32^, we only performed RNA-Seq on the three most recently collected samples from each FLAC subgroup, resulting in only six tumors and matched normal samples with gene expression data. In our study, we observed certain discrepancies in patient characteristics and disease stage compared to the established literature. In previous studies, H-FLAC is more common in elderly male smokers, while L-FLAC tends to affect young female non-smokers. However, in our sample, there were no differences in smoking history or gender between the L-FLAC and H-FLAC groups. Additionally, while H-FLAC patients usually present at an advanced stage, our study included patients at an early stage of the disease. This discrepancy may be due to the limited sample size of both our study and prior research. Future research with larger and more diverse patient populations is needed to validate our findings on intratumor heterogeneity and prognosis in both H-FLAC and L-FLAC.

To our knowledge, our study is the largest cohort to date utilizing private muti‐omics data from multi‐regional sampling for heterogeneity analysis in FLAC. Overall, our multi‐omics analysis provides new insights into genomic, epigenomic, and transcriptomic landscape and multi-level heterogeneity of FLAC and its subtypes.

## Methods

### Patients and sample collection

A total of 24 patients who underwent tumor resection with curative intent, followed by adjuvant therapy when indicated by the standard of clinical guidelines were retrospectively recruited in this study from January 2013 to August 2021. Pathological classification of FLAC was evaluated according to the criteria of the 2021 WHO Classification. Overall survival was defined as the time from the initial diagnosis until death from any cause. Recurrence-free survival (RFS) was measured from the date of surgery to the radiographic recurrence (local or distant). Patients without events were censored from the time point of the last follow-up. A total of 24 formalin-fixed and paraffin-embedded (FFPE) samples were collected. Four samples and patients were excluded from the study as they did not pass the qualification process. To evaluate the intratumor heterogeneity, three regions were separated by a margin of at least 0.3 cm from each sample. Matched normal tissue was collected from the visible edge of the tumor and did not contain any tumor cells by histopathologic review. The genetic tests were performed in a centralized clinical testing center (Nanjing Geneseeq Technology Inc., China; Certified to CAP, CLIA and ISO15189) according to protocols reviewed.This study was approved by the ethical committee of Cancer Hospital, Chinese Academy of Medical Sciences, and it was performed in compliance with the Declaration of Helsinki Principles. Written informed consent to participate and publication were obtained from all patients.

**Fig. 5:**
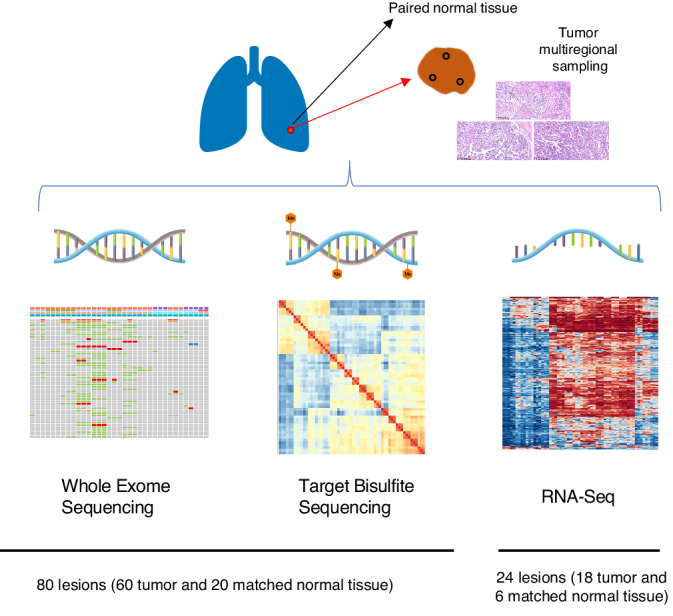
Patient enrollment flowchart. Schematic representation of the study design showing multi-omics analysis based on multi-region profiling of tumor specimens.

To explore the genomic and epigenomic heterogeneity, we performed whole-exome sequencing (WES) and target bisulfite sequencing (Target-BS) on 60 multi-regional tumor formalin-fixed, paraffin-embedded (FFPE) samples and 20 normal FFPE samples from all 20 FLAC patients (Fig. 5). The average whole-exome sequencing depth was 249× and 75× for tumor and normal FFPE samples, respectively. For Target-BS, the average depth was 34× for all samples. We also performed whole transcriptome Shotgun Sequencing (RNA-Seq) on tissue samples from the six recently recruited patients (after April 2021). All sequencing procedures were performed on the same FFPE core for each respective sampling region.

### Sample processing and DNA/RNA extraction

Genomic DNA was extracted for whole-exome sequencing and target-bisulfite sequencing. FFPE tumor samples were de-paraffinized with xylene, and genomic DNA was extracted using QIAamp DNA FFPE Tissue Kit (Qiagen). Purified genomic DNA was qualified by Nanodrop2000 for A260/280 and A260/A230 ratios (Thermo Fisher Scientific). All DNA samples were quantified by Qubit 3.0 using the dsDNA HS Assay Kit (Life Technologies) according to the manufacturer’s recommendations.

Total RNA from tissue samples was also extracted from the sample core using an RNeasy Mini Kit (Qiagen). RNA purity was checked using Nanodrop 2000 for A260/280 and A260/A230 ratios (Thermo Fisher Scientific). All RNA samples were quantified by Qubit 3.0 using the RNA BR Assay Kit (Life Technologies).

### Whole-exome sequencing

The whole-exome sequencing was performed as previously described^33,34^. Sequencing libraries were prepared using KAPA Hyper Prep kit (Roche) with an optimized manufacturer’s protocol and enriched using the xGen Exome Research Panel (Integrated DNA technologies), and Hybridization and Wash Kit. Libraries were then sequenced on an Illumina HiSeq4000 platform using PE150 sequencing chemistry (Illumina). The average whole-exome sequencing depth was 249× and 75× for tumor and normal FFPE samples, respectively. The average coverage size of WES for the tumor mutation burden estimation was 39 Mb.

Formalin-fixed, paraffin-embedded specimens are associated with potential sequencing artifacts, such as C > T (or G > A) alterations from deamination and G > T (or C > A) alterations from oxidation. We applied rigorous quality control procedures before further analyses. The Picard tool (http://broadinstitute.github.io/picard/) was used to evaluate DNA damaging. All samples with Total QScores smaller than 35 or contamination rates greater than 0.02 were excluded. There were four samples excluded in the quality control processes.

Trimmomatic^35^ was used for FASTQ file quality control. Leading/trailing low quality (quality reading below 20) or N bases were removed. Paired-end reads were then aligned to the reference human genome (build hg19) using the Burrows-Wheeler Aligner^36^ with the default parameters. PCR deduplication was performed using Picard (Broad Institute). Local realignment around indels and base quality score recalibration were performed using Genome Analysis Toolkit (GATK 3.4.0). Furthermore, samples with a mean depth <30X were removed. Somatic single nucleotide variant (SNV) and insertion/deletions (indels) calling were performed using Mutect2^37^ and filtered by the following criteria: (i) filtered if SNV supporting reads < 6 or small indel supporting reads < 8; (ii) filtered if present in >1% population frequency in the 1000 Genomes2 or ExAC database. The final list of mutations was annotated using vcf2maf (call VEP for annotation). Tumor mutation burden (TMB) was defined as the total number of missense mutations. To reduce the false negative rate of mutation detections in multi-regional sequencing, we retrieved mutations of low allelic frequencies by the following rule: if a specific mutation passed the filtering criteria in one regional sample and had at least one supporting reads in a paired regional sample, it would be called in both samples. Genes in the COSMIC cancer gene census (v96) were defined as driver genes. The oncogene and tumor suppressor genes (TSG) were classified based on the driver gene list.

### Identification of copy number alterations

Copy number analysis of the WES data was performed using FACETS^38^ (Ver 0.5.13). Copy number alteration (CNA) events for amplification or deletion were assigned based on the sample-ploidy adjusted copy number calculated by the FACETS algorithm. In brief, chromosome arm-level CNA gain was identified if the segments of gain accounted for more than 60% of the total segments of the corresponding chromosome arm. Arm-level CNA loss was identified if the segments of loss accounted for more than 60% of the total segments of the given chromosome. Tumor purity was also calculated by FACETS. For focal CNA, segments contributing to deep amplification and deep deletion events were considered for analysis. Fisher’s exact test was used to compare arm-level CNA differences with FDR adjustment of the p values. Significantly amplified or deleted focal CNA regions across a set of samples were identified using the Genome Identification of Significant Targets in Cancer^39^ (GISTIC, v.2.0) algorithm with parameters considering our sample size as follows: *q*-value < 0.1, log2 ratio = 0.2, broad = 1, brlen = 0.6, and genegistic = 1.

### Mutation signatures

Somatic mutation signature analysis was performed using a comprehensive and integrated suite of bioinformatic tools (SigProfiler) for non-negative matrix factorization of the mutation catalog matrix into de novo mutation signatures and relative exposure of each signature for each cancer genome^20^. Signatures were compared against their respective COSMIC reference signature (v.3.3, June 2022, https://cancer.sanger.ac.uk/ cosmic/signatures/) using the cosine similarity metric. Signatures with cosine similarity to a COSMIC signature >0.6 were considered for matching and were verified through manual inspection of the similarity matrix and the signatures themselves.

### Target-bisulfite sequencing and identification of differentially methylated region (DMR)

To construct the sequencing libraries, 1 μg of DNA per FFPE sample was used and then treated with bisulfite. Targeted bisulfite sequencing was then performed on Illumina Hiseq platform (Illumina, San Diego, CA) using predesigned probes (SeqCap Epi CpGiant, Roche), which targeted a total of ~2.7 × 106 CpG sites within ~80.5 Mb of genome region. Raw sequencing data were first demultiplexed by bck2fastq and then trimmed by Trimmomatic^35^ as part of the quality control (QC) protocol. The qualified reads were then mapped onto the human reference genome (GRCh37/UCSC hg19) using the bisulfite sequence aligner Bismark^40^ after PCR duplicate removal by the Picard toolkit.

Differentially methylated regions (DMRs) were analyzed using the R package methylKit^41^ (version 1.2.0) by comparing tumor and normal tissue for each FLAC subgroup. CpG clusters were initially divided into 100 bp windows with a sliding step of 100 bp based on in-house assay validation. The methylation level of each DMR was determined by dividing the total methylated cytosines by the total number of CpGs within each window. A relatively stringent cutoff of a minimum 0.2 methylation difference was used for DMR calling with a *q*-value-based false discovery rate (FDR) at a 0.05 significance level controlled by the Sliding Linear Model (SLIM). DMRs with low sequencing depth and low-quality reads were filtered.

### whole transcriptome sequencing and differentially expressed gene (DEG) analysis

RNA-Seq were only performed on tissue samples from the six recently recruited patients. RNA integrity was assessed using Bioanalyzer 2100 (Agilent). RIN value (RNA integrity number) higher than 7 was required. RNA sequencing libraries were prepared using KAPA Stranded RNA-Seq Kit with RiboErase (KAPA Biosystems). Briefly, rRNA was first depleted with RiboErase, followed by DNase digestion for DNA removal. Purified RNA was subjected to first-strand cDNA synthesis, followed by second-strand synthesis with dUTP marking for strand-specificity. This is followed by A-tailing, adapter ligation and library amplification. The final library was quantified using the KAPA Library Quantification Kit (KAPA Biosystems), and its fragment size distribution was analyzed using a Bioanalyzer 2100 (Agilent). Sequencing was performed on the Illumina HiSeq4000 platform using PE150 sequencing chemistry (Illumina, USA) to an average of 60 M reads per sample.

FASTQ file quality control was performed using Trimmomatic^35^, where N bases and low-quality (score < 15) bases were removed. Reads aligning to rRNA and tRNA sequences were removed. Cleaned reads were aligned to the human reference genome (hg19) using STAR^39^ (v 2.5.2b), a splice-aware aligner. Transcripts were quantified using RSEM^42^, which uses an expectation maximization algorithm to optimally assign reads that maps to multiple transcripts.

Coverage uniformity were inspected by RSeQC^43^, which showed no sign of bias (Supplementary Fig. 16).

Differential expression analysis of miRNA was performed by using the r package DESeq2 and evaluated by fitting a negative binomial generalized linear model and adjusted for multiple testing using the Benjamini-Hochberg correction with a false discovery rate (FDR) of 0.1 and a minimum fold change of 2.

### Phylogenetic tree construction

Regional genetic and epigenetic evolutionary trees for each patient were built by applying a minimum evolution algorithm using *fastme.ols* from r package ape^44^ to infer phylogenetic relationships based on distances between samples using the Euclidean distance, including a normal tissue sample used as the tree root. For somatic mutations or CNV, binary matrix based on the presence and absence of somatic mutations or CNV were used to calculate the distance. For methylation data, CpG clusters were divided into 500 kb windows and the beta value of each window were calculated and used for distance calculation after correction for tumor purity using an R package ‘InfiniumPurify’^45^.

### Evaluation of multi-level intratumor heterogeneity

Mutational or CNV ITH was evaluated for each patient based on the presence of each somatic mutation or CNV in different tumor regions and presented as the Jaccard distance as described by Zhang et al.^46^. Jaccard distance was calculated by subtracting the Jaccard coefficient from 1.

1 $${d}_{J}\left({V}_{i},{V}_{j}\right)=1-J\left({V}_{i},{V}_{j}\right)=1-\frac{\left|{V}_{i}\cap {V}_{j}\right|}{\left|{V}_{i}\cup {V}_{j}\right|}$$

Jaccard distance is the range from 0 (lowest ITH) to 1 (highest ITH). If the tumor has less shared somatic genetic alterations or CNV after multi-region sequencing, namely less trunk mutations, the ITH of this tumor will be higher. Otherwise, if the tumor has more trunk mutations, the ITH of this tumor will be lower.

To estimate DNA methylation ITH and transcriptional ITH, we computed the Jensen-Shannon distance (JSD) between two probability distributions, P_1_ and P_2_, of the tumor purity-corrected methylation level or tumor purity-corrected gene expression level to quantify the dissimilarity between methylation patterns or gene expression of two samples^47–49^. The JSD was calculated as

2 $${JSD}\,({P}_{1},{P}_{2})=\sqrt{\frac{1}{2}[{D}_{{KL}}({P}_{1},R)+{D}_{{KL}}({P}_{2},R)]}$$

where $R=\frac{P+Q}{2}$ and ${{\rm{D}}}_{{\rm{KL}}}\left({{\rm{M}}}_{1},{{\rm{M}}}_{2}\right)=\sum {{\rm{Q}}}_{1}({\rm{i}}){\log }_{2}\left[\frac{{{\rm{Q}}}_{1}({\rm{i}})}{{{\rm{Q}}}_{2}(i)}\right]$ is the Kullback–Leibler divergence of a probability distribution Q_2_ from a probability distribution Q_1_. The JSD takes values between 0 and 1, with 0 indicating that the probability distributions P1 and P2 are identical and 1 indicating that the supports of the two probability distributions do not intersect each other.

### Statistical analyses

Kaplan–Meier estimate were used to assess the association of ctDNA and clinical variables. All *P* values were based on 2-sided testing, and differences were considered significant at *P* ≤ 0.05. Statistical analysis was performed using R software, version 4.1.3.

### Reporting summary

Further information on research design is available in the Nature Research Reporting Summary linked to this article.

### Supplementary information

REPORTING SUMMARY

Supplementary Figures and Tables

Supplementary Data 1

Supplementary Data 2

Supplementary Data 3

Supplementary Data 4

## Data Availability

All raw DNA-sequencing, target-bisulfite and RNA-seq data have been deposited in the National Genomics Data Center (NGDC) under the accession code: HRA003555. The raw sequencing data contain information unique to individuals and are available under controlled access. Access to the data can be requested by completing the application form via GSA-Human System and is granted by the corresponding Data Access Committee. Additional guidance can be found at the GSA-Human System website [https://ngdc.cncb.ac.cn/gsa-human/document/GSA-Human_Request_Guide_for_Users_us.pdf].

## Code availability

The code that supports the findings of this study are available from the corresponding authors on reasonable request.
