# Supplementary material for: Molecular landscape and multi-omic measurements of heterogeneity in fetal adenocarcinoma of the lung
Source: NPJ Precis Oncol. 2024 Jun 3;8:99. doi: 10.1038/s41698-024-00569-y (PMC11148097; doi:10.1038/s41698-024-00569-y)
Supplement: Supplementary file 1 — REPORTING SUMMARY [file 41698_2024_569_MOESM1_ESM.pdf]

nature portfolio

Corresponding author(s): Lin Lin

Last updated by author(s): Feb 17, 2023

Reporting Summary

Nature Portfolio wishes to improve the reproducibility of the work that we publish. This form provides structure for consistency and transparency in reporting. For further information on Nature Portfolio policies, see our Editorial Policies and the Editorial Policy Checklist.

Statistics

For all statistical analyses, confirm that the following items are present in the figure legend, table legend, main text, or Methods section.

n/a	Confirmed	
☐	☒	The exact sample size (n) for each experimental group/condition, given as a discrete number and unit of measurement
☐	☒	A statement on whether measurements were taken from distinct samples or whether the same sample was measured repeatedly
☐	☒	The statistical test(s) used AND whether they are one- or two-sided<br>*Only common tests should be described solely by name; describe more complex techniques in the Methods section.*
☐	☒	A description of all covariates tested
☒	☐	A description of any assumptions or corrections, such as tests of normality and adjustment for multiple comparisons
☐	☒	A full description of the statistical parameters including central tendency (e.g. means) or other basic estimates (e.g. regression coefficient) AND variation (e.g. standard deviation) or associated estimates of uncertainty (e.g. confidence intervals)
☐	☒	For null hypothesis testing, the test statistic (e.g. F, t, r) with confidence intervals, effect sizes, degrees of freedom and P value noted<br>*Give P values as exact values whenever suitable.*
☒	☐	For Bayesian analysis, information on the choice of priors and Markov chain Monte Carlo settings
☒	☐	For hierarchical and complex designs, identification of the appropriate level for tests and full reporting of outcomes
☐	☒	Estimates of effect sizes (e.g. Cohen's d, Pearson's r), indicating how they were calculated

Our web collection on statistics for biologists contains articles on many of the points above.

Software and code

Policy information about availability of computer code

Data collection	No software are used for data collection.
Data analysis	Trimmomatic was used for FASTQ file quality control. Qualified reads were then mapped to reference human genome (hg19) using Burrows-Wheeler Aligner. PCR duplicates were removed by Picard (Broad Institute, MA, USA) after local realignment around known indels and base quality recalibration using Genome Analysis Toolkit (GATK 3.4.0). Single-nucleotide variations and insertion/deletion were detected using VarScan2. Genomic fusions were identified by FACTERA. All statistical analyses were performed using R version 4.1.3. R package 'survival' (version 3.2-10) was used for survival analysis.

For manuscripts utilizing custom algorithms or software that are central to the research but not yet described in published literature, software must be made available to editors and reviewers. We strongly encourage code deposition in a community repository (e.g. GitHub). See the Nature Portfolio guidelines for submitting code & software for further information.

Data

Policy information about availability of data

All manuscripts must include a data availability statement. This statement should provide the following information, where applicable:

- Accession codes, unique identifiers, or web links for publicly available datasets
- A description of any restrictions on data availability
- For clinical datasets or third party data, please ensure that the statement adheres to our policy

All raw DNA-sequencing, target-bisulfite and RNA-seq data have been deposited in the National Genomics Data Center (NGDC) under the accession code: subHRA005096. The raw sequencing data contain information unique to individuals and are available under controlled access. Access to the data can be requested by completing the application form via GSA-Human System and is granted by the corresponding Data Access Committee. Additional guidance can be found at the GSA-Human System website [https://ngdc.cncb.ac.cn/gsa-human/document/GSA-Human_Request_Guide_for_Users_us.pdf].

Human research participants

Policy information about studies involving human research participants and Sex and Gender in Research.

Reporting on sex and gender	sex and gender were not considered in the study design.
Population characteristics	Overall, twenty patients with FLAC were enrolled from March 2011 to August 2021. Twelve of the patients (60%) were high-grade FLAC (H-FLAC) and eight were low-grade FLAC (L-FLAC). The median age of the patients enrolled was 62.5 years (range: 31-81 years). Five of the patients (15%) were female and fifteen (75%) were smokers. Most of the patients were of early-stage disease (Stage I: 14/20, 70%).
Recruitment	A total of 20 patients who underwent tumor resection with curative intent, followed by adjuvant therapy when indicated by the standard of clinical guidelines were retrospectively recruited in this study from January 2013 to August 2021.
Ethics oversight	The study was approved by the Ethics Committee of Cancer Hospital, Chinese Academy of Medical Sciences and Peking Union Medical College. All patients provided oral and written informed consent.

Note that full information on the approval of the study protocol must also be provided in the manuscript.

Field-specific reporting

Please select the one below that is the best fit for your research. If you are not sure, read the appropriate sections before making your selection.

☒ Life sciences ☐ Behavioural & social sciences ☐ Ecological, evolutionary & environmental sciences

For a reference copy of the document with all sections, see nature.com/documents/nr-reporting-summary-flat.pdf

Life sciences study design

All studies must disclose on these points even when the disclosure is negative.

Sample size	As an retrospective study, no sample calculations were performed. The number used was similar to those reported in previous publications in the field.
Data exclusions	No data was excluded.
Replication	This study is a observational cohort study and therefore is not applicable for experimental replication.
Randomization	Our study was an observational study with no intervention and cohorts based on pre-determined criteria; as such, there is no randomization as part of this study.
Blinding	Our study was an observational study with no intervention and cohorts based on pre-determined criteria; as such, blinding was not relevant.

Reporting for specific materials, systems and methods

We require information from authors about some types of materials, experimental systems and methods used in many studies. Here, indicate whether each material, system or method listed is relevant to your study. If you are not sure if a list item applies to your research, read the appropriate section before selecting a response.

Materials & experimental systems

n/a	Involved in the study
☒	☐ Antibodies
☒	☐ Eukaryotic cell lines
☒	☐ Palaeontology and archaeology
☒	☐ Animals and other organisms
☒	☐ Clinical data
☒	☐ Dual use research of concern

Methods

n/a	Involved in the study
☒	☐ ChIP-seq
☒	☐ Flow cytometry
☒	☐ MRI-based neuroimaging
