# Supplementary material for: Molecular landscape and multi-omic measurements of heterogeneity in fetal adenocarcinoma of the lung
Source: NPJ Precis Oncol. 2024 Jun 3;8:99. doi: 10.1038/s41698-024-00569-y (PMC11148097; doi:10.1038/s41698-024-00569-y)
Supplement: Supplementary file 2 — Supplementary Figures and Tables [file 41698_2024_569_MOESM2_ESM.pdf]

Supplementary Table 1. De novo extracted mutational signatures

De novo extracted signatures	Global NMF Signatures	L1 Error %	L2 Error %	Cosine Similarity
Signature 96-A	Signature SBS1 (3.06%) & Signature SBS4 (76.16%) & Signature SBS5 (20.78%)	0.39	0.41	0.92
Signature 96-B	Signature SBS1 (10.82%) & Signature SBS5 (24.16%) & Signature SBS6 (35.04%) & Signature SBS7a (6.82%) & Signature SBS7b (8.66%) & Signature SBS18 (14.50%)	0.36	0.26	0.97
Signature DINUC-A	Signature DBS2 (48.80%) & Signature DBS6 (14.52%) & Signature DBS9 (24.96%) & Signature DBS10 (11.72%)	0.86	0.47	0.91
Signature INDEL-A	Signature INDEL-A	0	0	1

Supplementary Table 2 Multivariate cox regression analysis of FLAC patients on Regression-free survival and overall survival

Variable	Regression-free survival		Overall survival	
	loglik	P value	loglik	P value
Mutational ITH level	-6.1048	0.0004	-1.7918	0.0005
Stage	-5.1472	0.16	-0.6931	0.13
Pathological classification	-5.1472	0.99	-0.6931	0.99

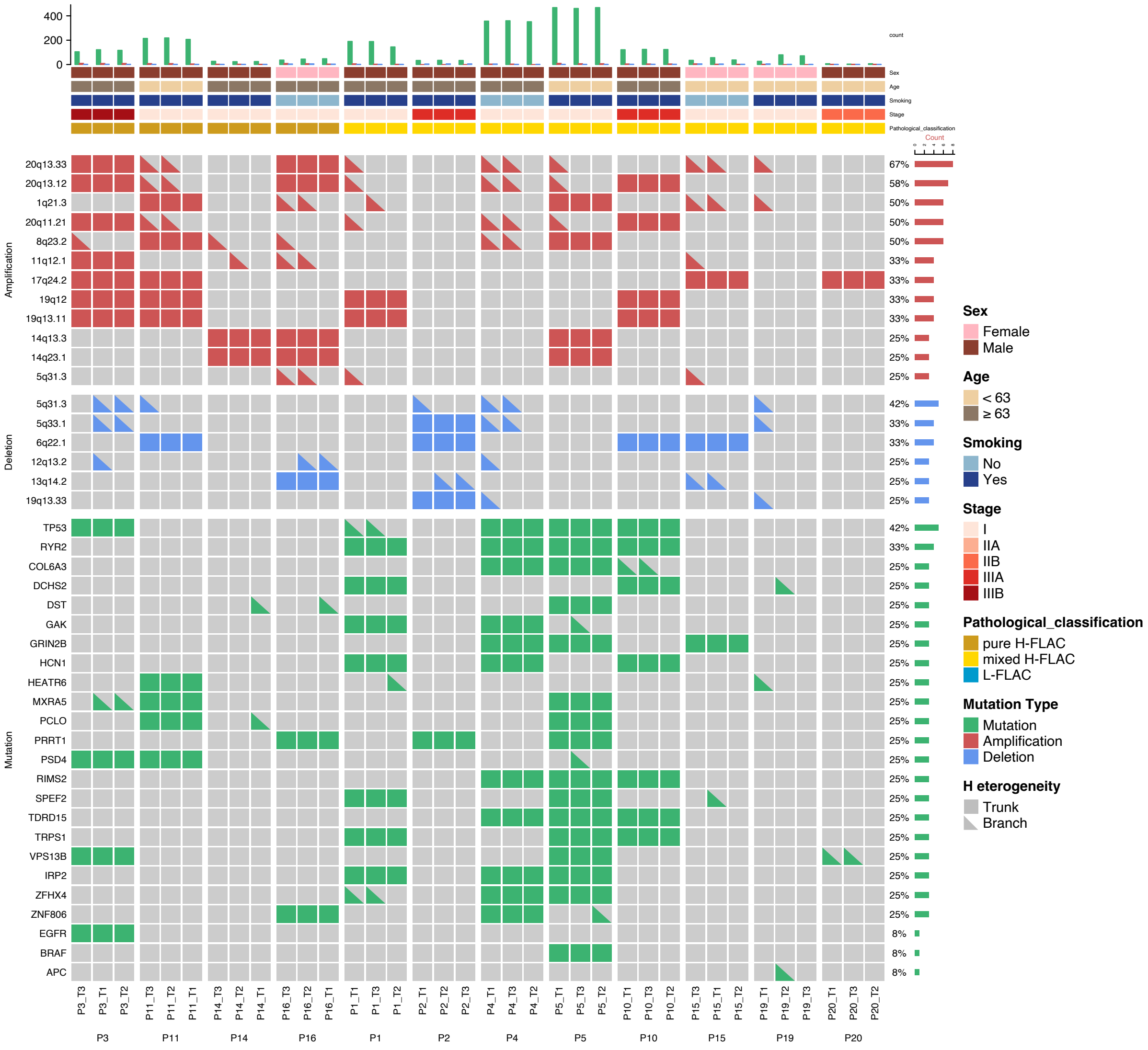

Supplementary Fig 1a Mutational landscape of H-FLAC (n=12). Alteration frequencies are calculated per patient.

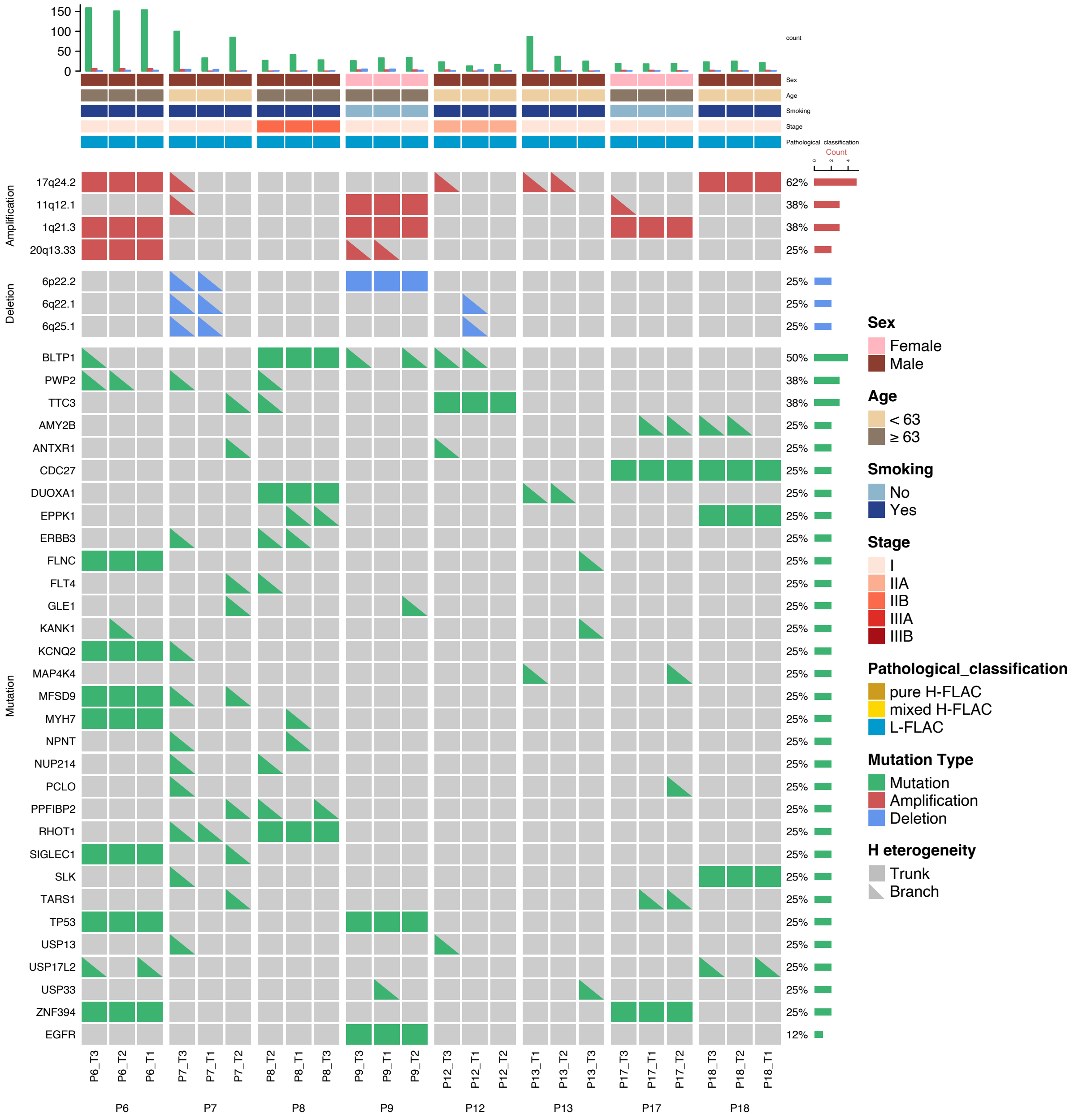

Supplementary Fig 1b Mutational landscape of L-FLAC (n=8). Alteration frequencies are calculated per patient.

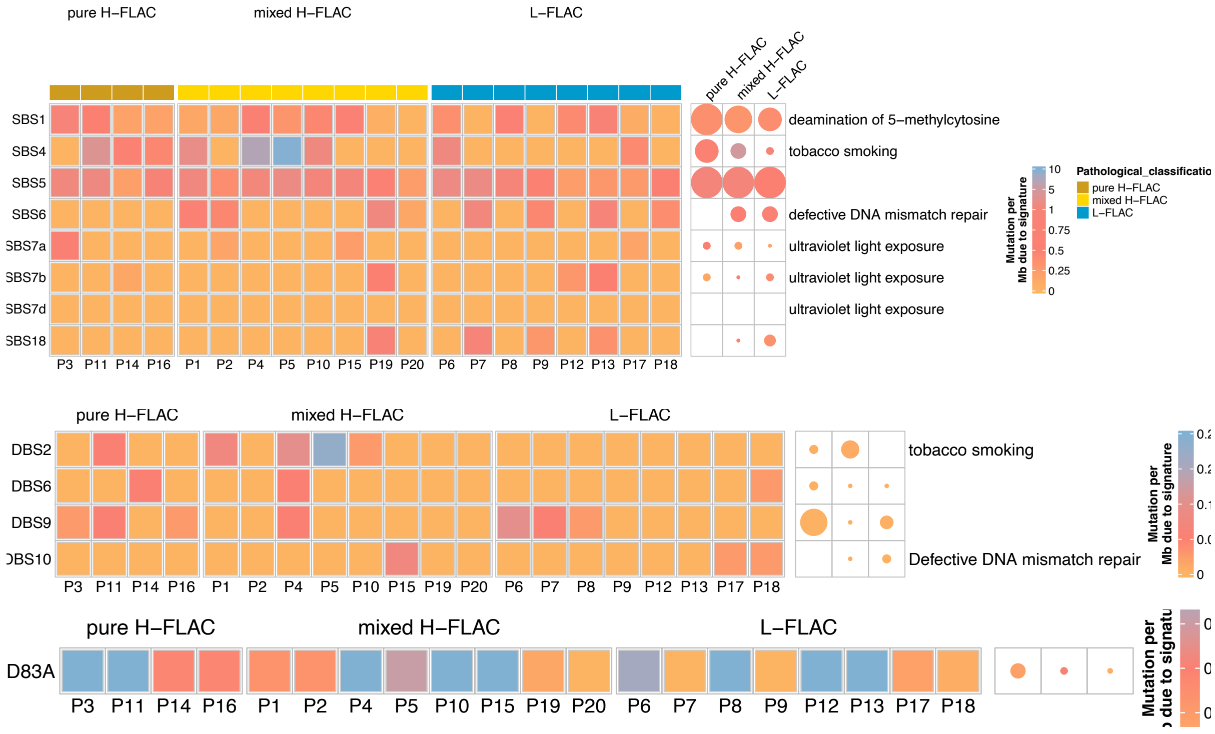

Supplementary Fig 2A COSMIC mutational signatures inferred from somatic base changes in pure and mixed H-FLAC and L-FLAC patients.

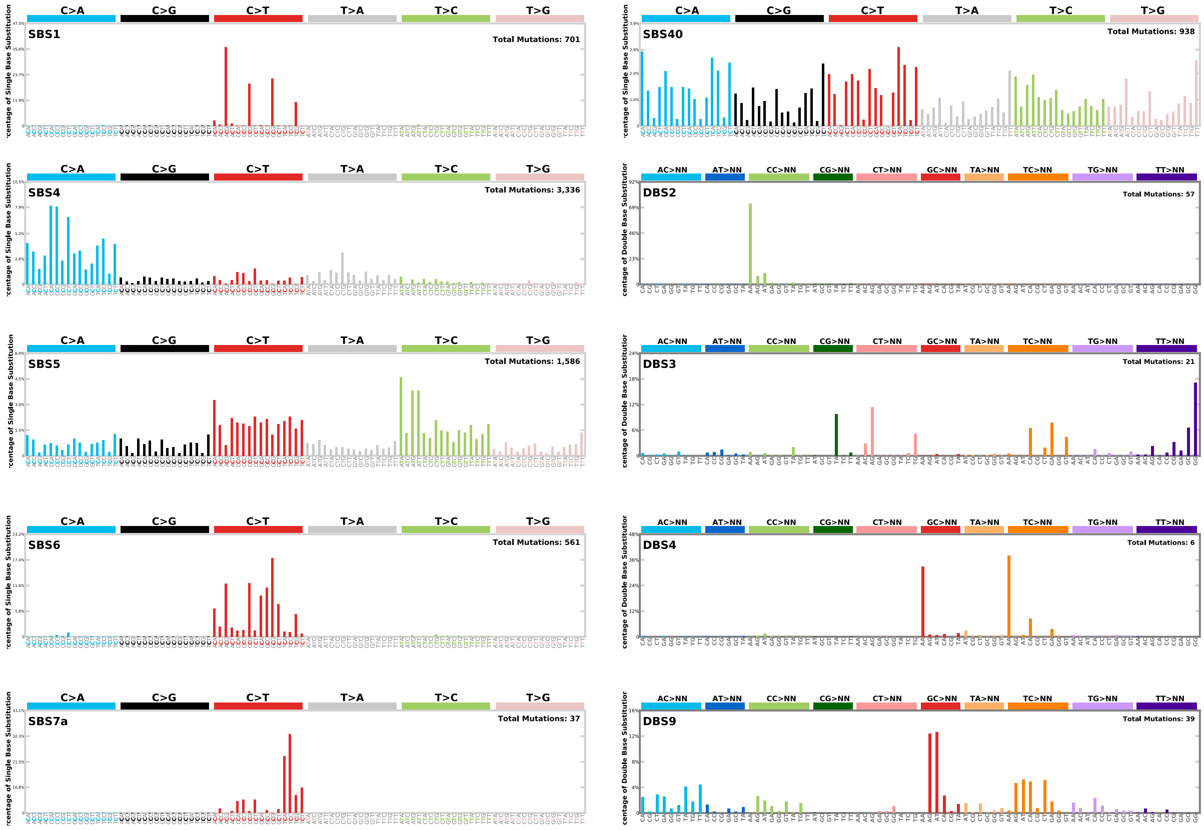

Supplementary Fig 2B COSMIC signatures that the de novo mutational signatures are

composed of.

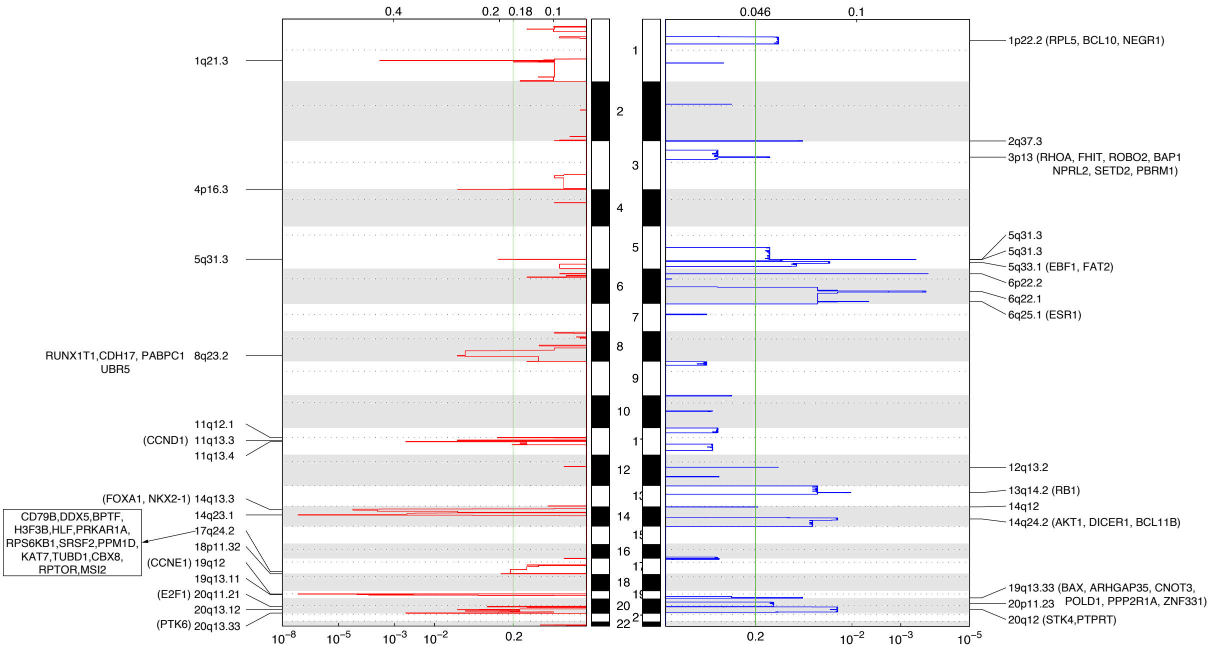

Supplementary Fig 3 Recurrent somatic copy-number variations in FLAC. Amplifications and deletions are plotted in red and blue, respectively.

A

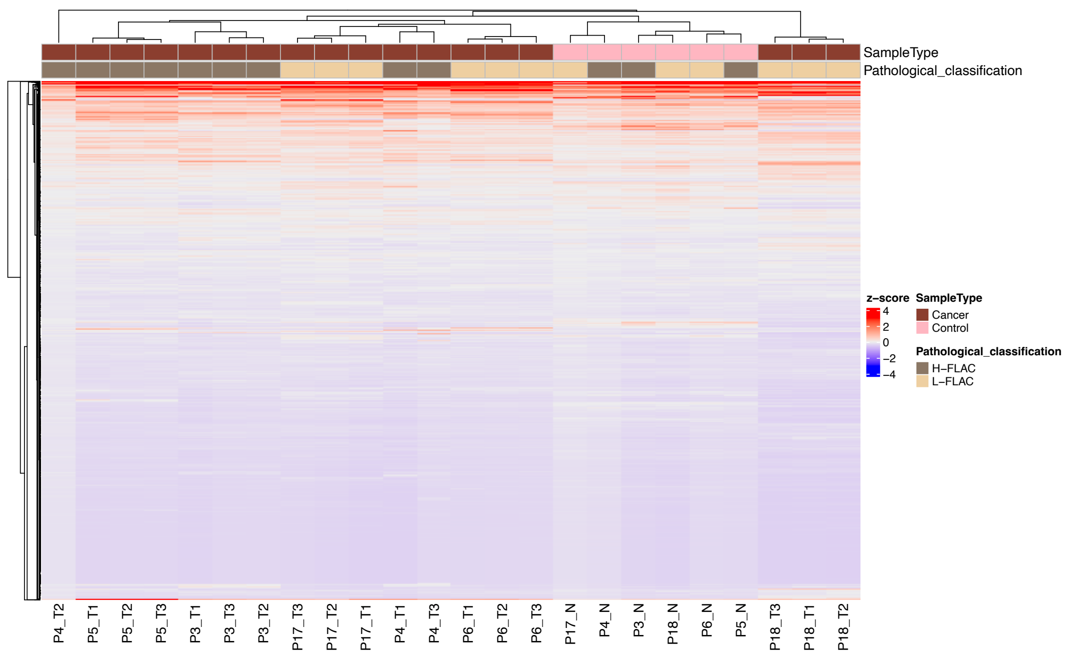

B

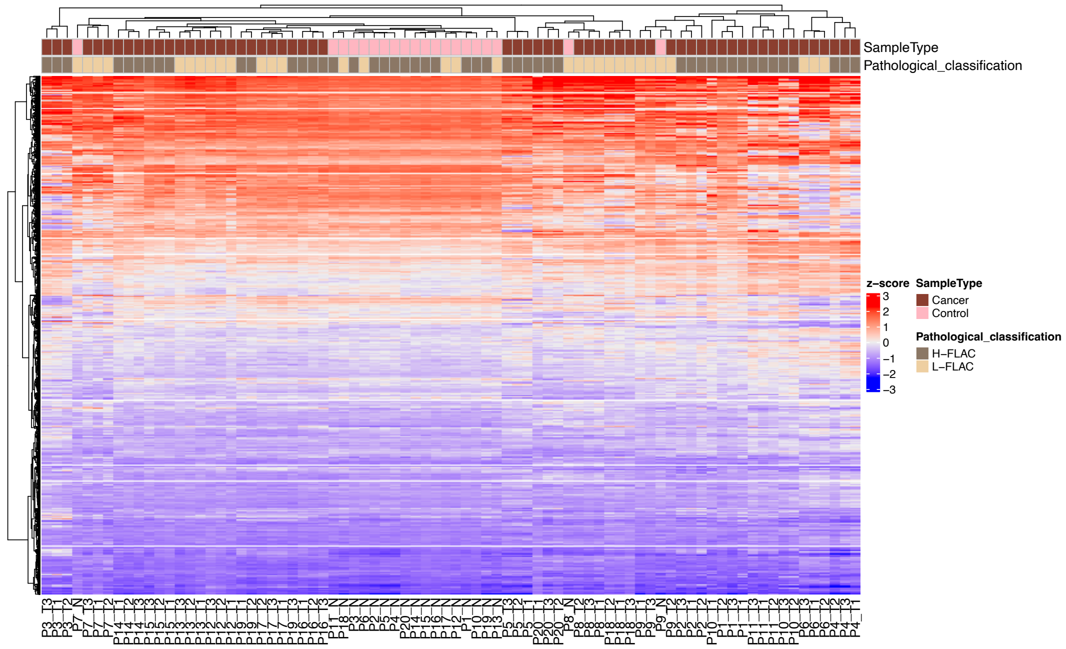

Supplementary Fig 4 Hierarchical clustering plot showing the extent of epigenomic (a) and transcriptomic (b) variation within and across tumor and non-malignant tissue regions from the patients.

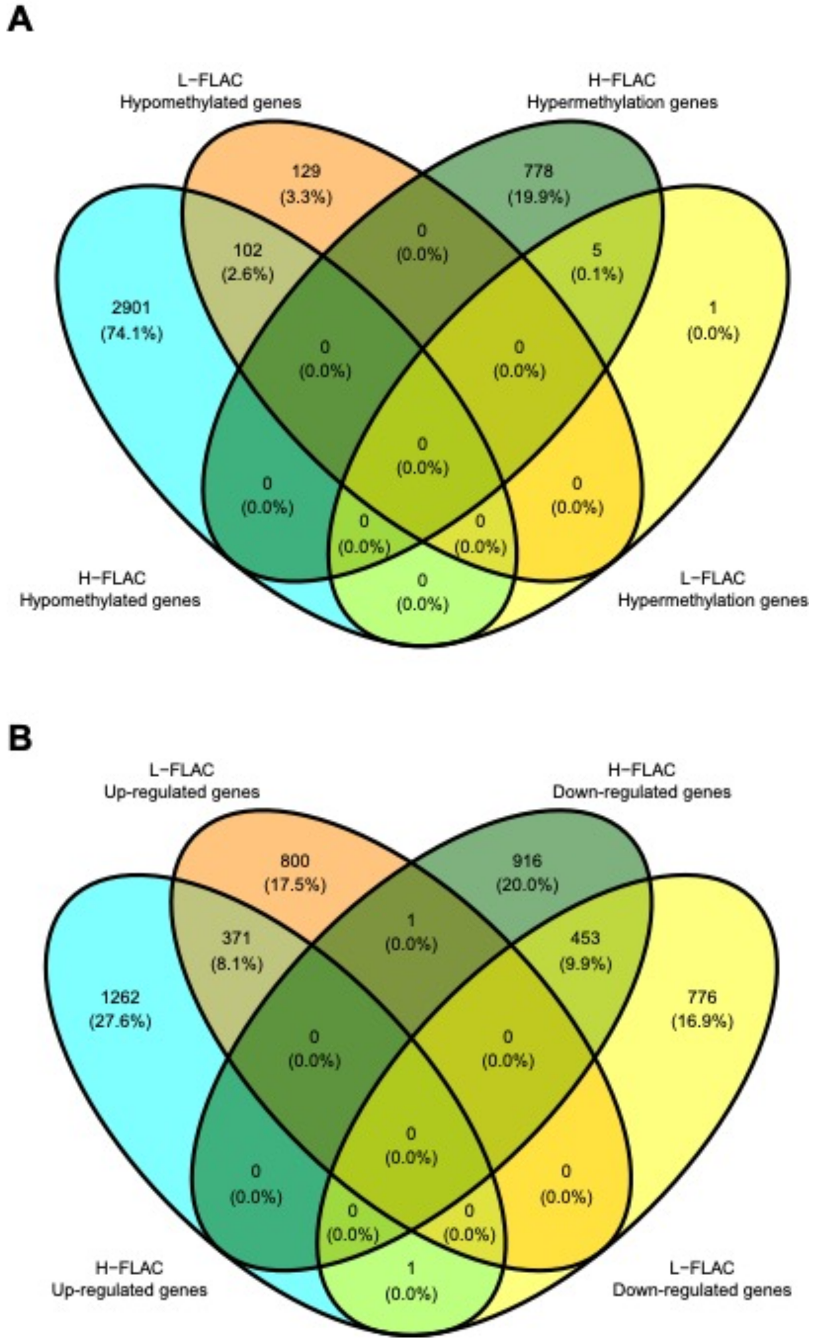

Supplementary Fig 5A-B Venn diagram of the differentially expressed genes.: The number in each circle represents the amount of differentially expressed genes between the different comparisons (test versus control). The overlapping number stands for the mutual differentially expressed genes between the different comparisons and the non-overlapping numbers specify the genes unique to each condition.

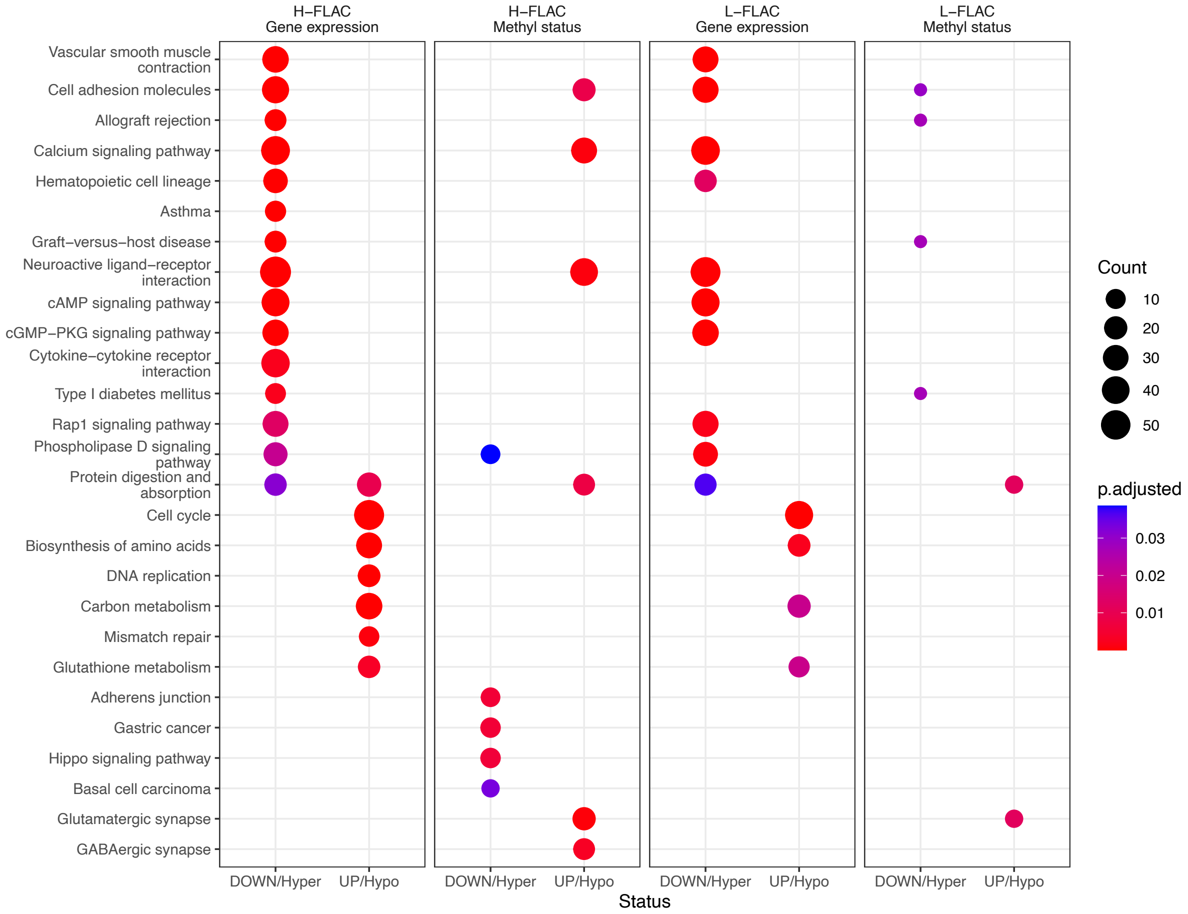

Supplementary Fig 6 Pathway enrichment comparison between H-FLAC and L-FLAC on samples with transcriptional data only based on differentially methylation region (DMR) results and differentially expressed gene (DEG) results

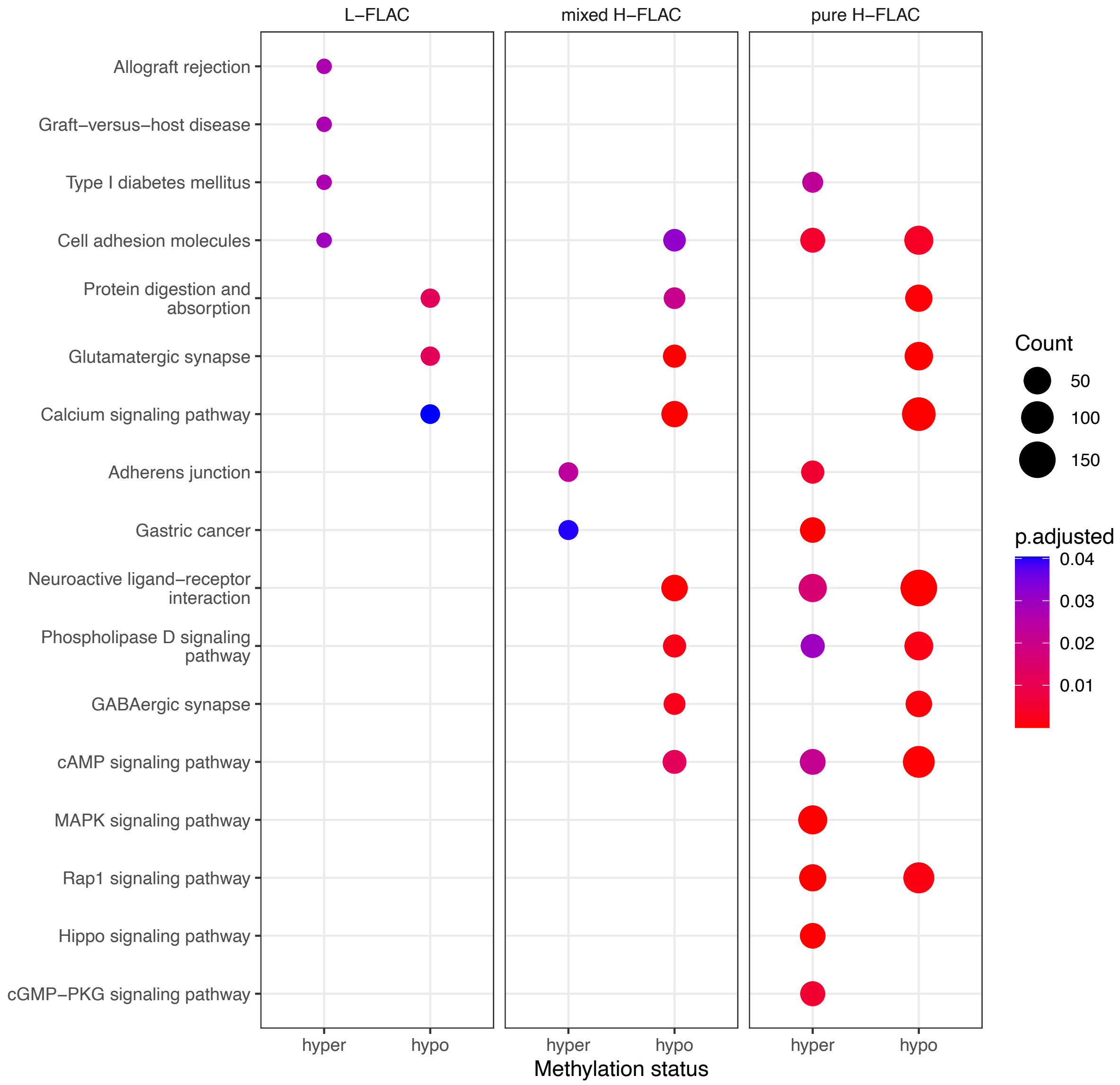

Supplementary Fig 7 Pathway enrichment comparison between pure H-FLAC,mixed H-FLAC and L-FLAC based on differentially methylation region (DMR) results.

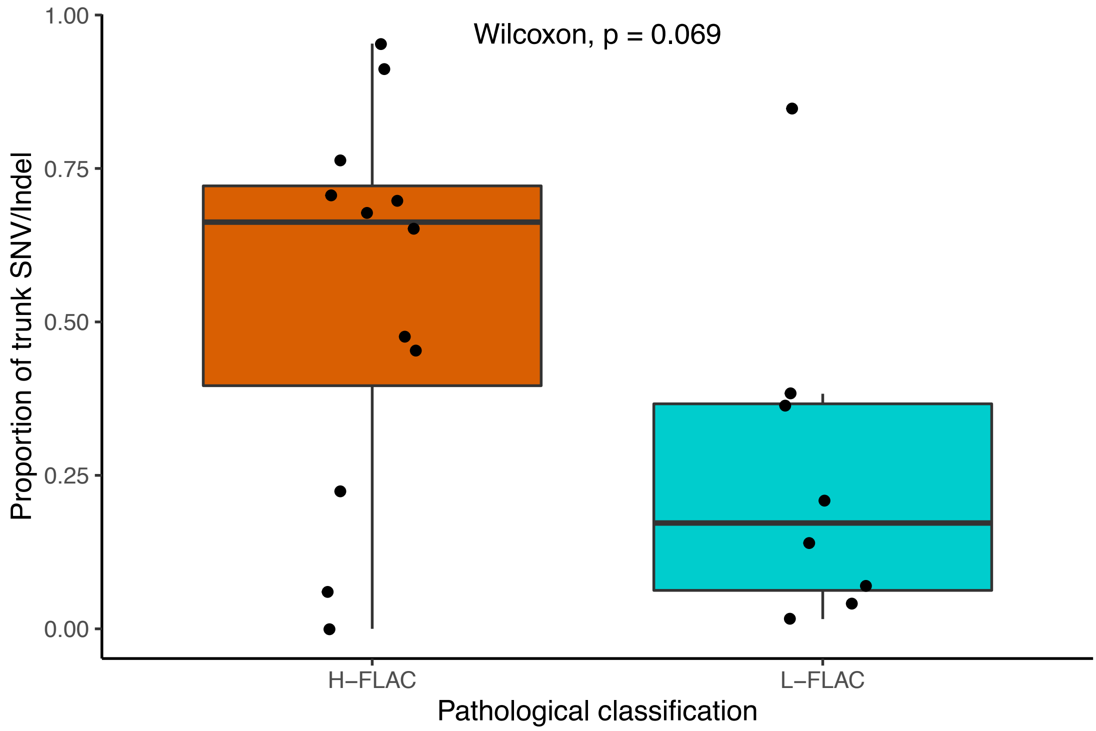

Supplementary Fig 8 Proportions of trunk mutations in H-FLAC tend to be higher than those in L-FLAC. The p value is calculated using Wilcoxon signed rank test. Center line, median; box limits, upper and lower quartiles; whiskers, 1.5x interquartile range.

A

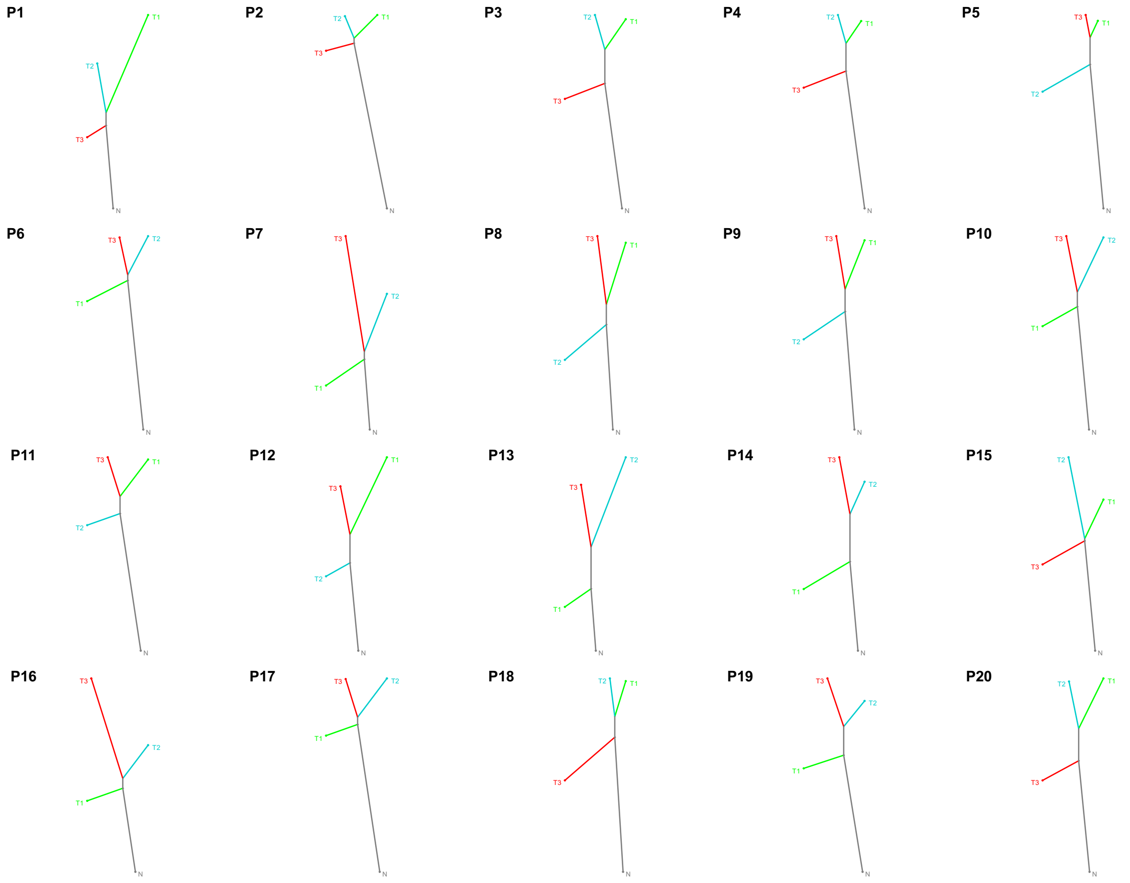

B

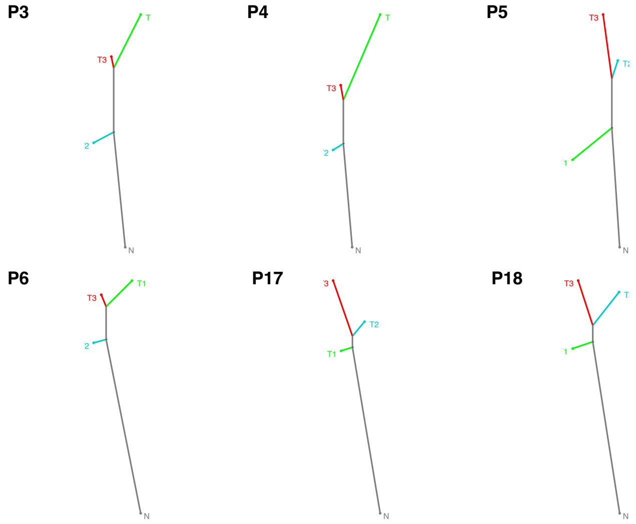

Supplementary Fig 7 Phylogenetic trees of FLAC based on copy number variants (a) and gene expression profiles (b).

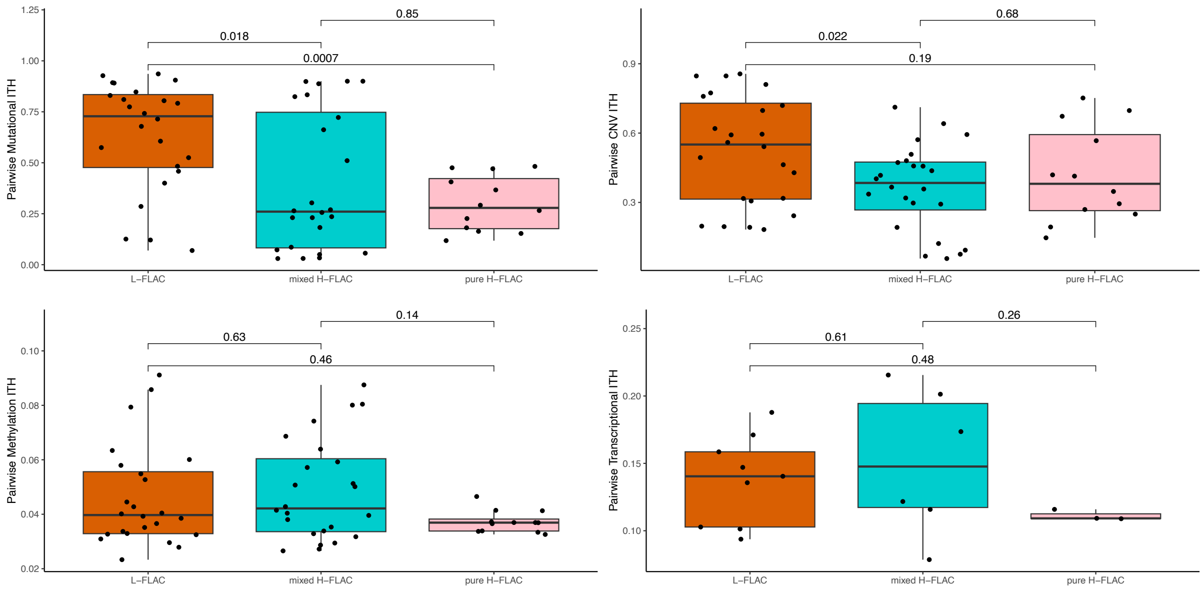

Supplementary Fig 10. Comparison of pairwise mutational, CNV, methylation and transcriptional ITH between pure H-FLAC, mixed H-FLAC and L-FLAC patients. The p value is calculated using Wilcoxon signed rank tesxt. Center line, median; box limits, upper and lower quartiles; whiskers, 1.5x interquartile range.

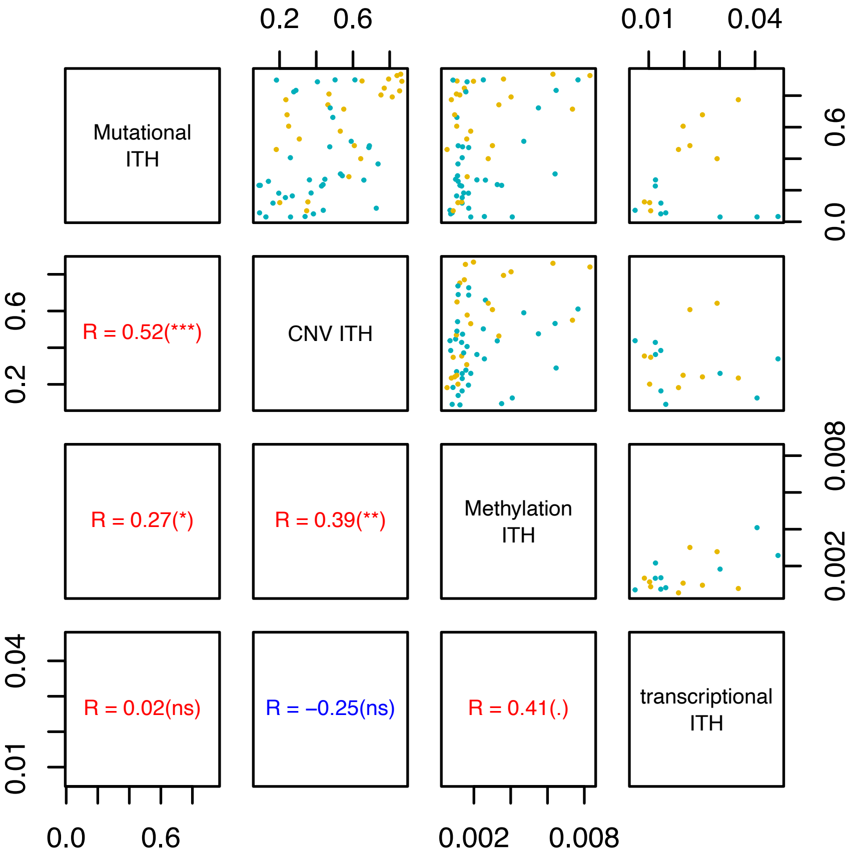

Supplementary Fig 10 Correlations between pairwise mutational, CNV, methylation and transcriptional ITH in all FLAC patients, assessed by two-tailed Spearman's correlation analysis. Each dot represents each pairwise comparison of regions within each tumor. ns: not significant; •: p<0.1; *: p<0.05; **: p<0.01; ***: p<0.001

A

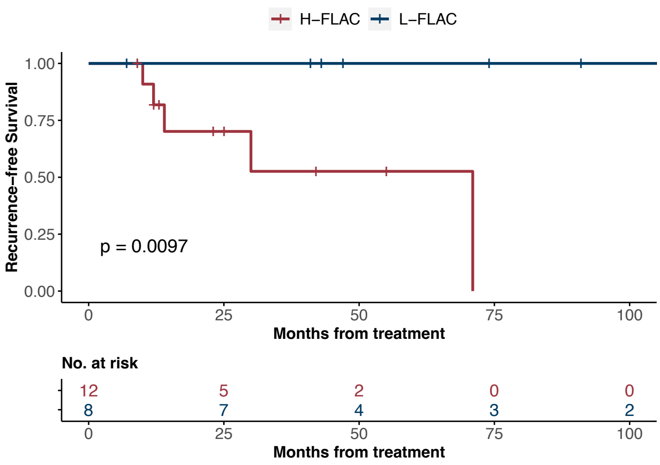

B

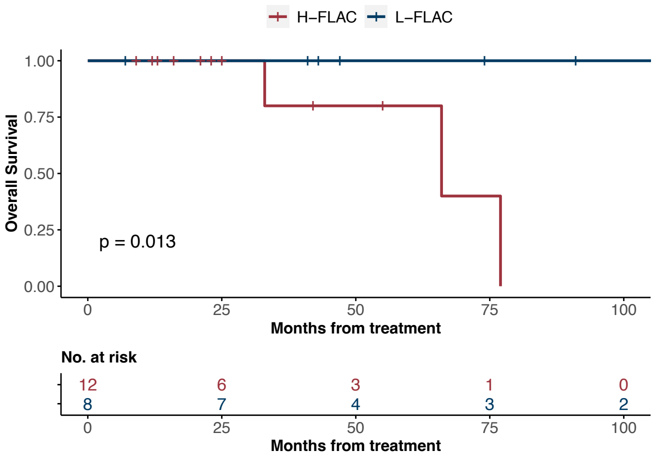

Supplementary Fig 11 Kaplan–Meier curve of recurrence-free survival (a) and overall survival (b) in patients stratified by subgroups.

A

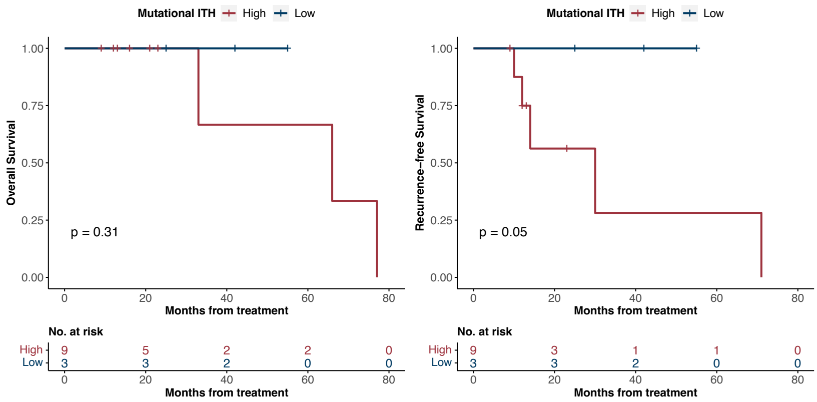

B

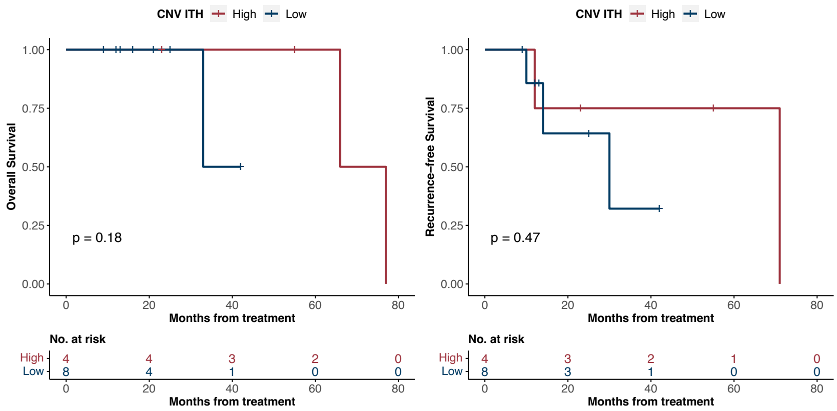

C

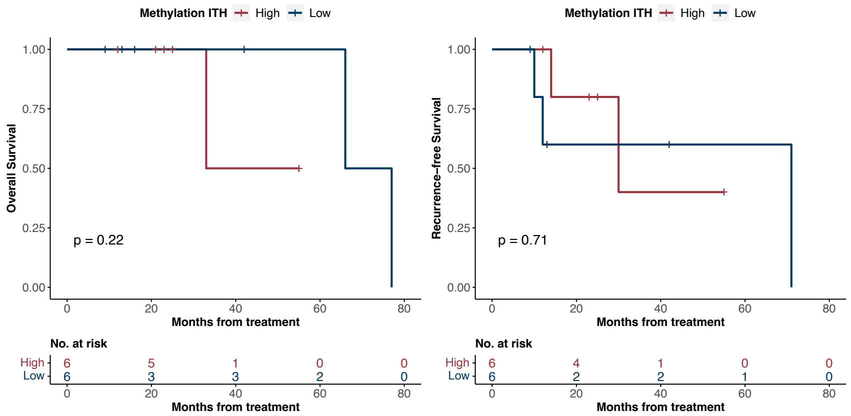

Supplementary Fig 12 Kaplan–Meier curve of recurrence-free survival (DFS) and overall survival (OS) in H-FLAC patients (n=12) stratified by median mutational ITH (a), CNV ITH (v) and methylation ITH (c).

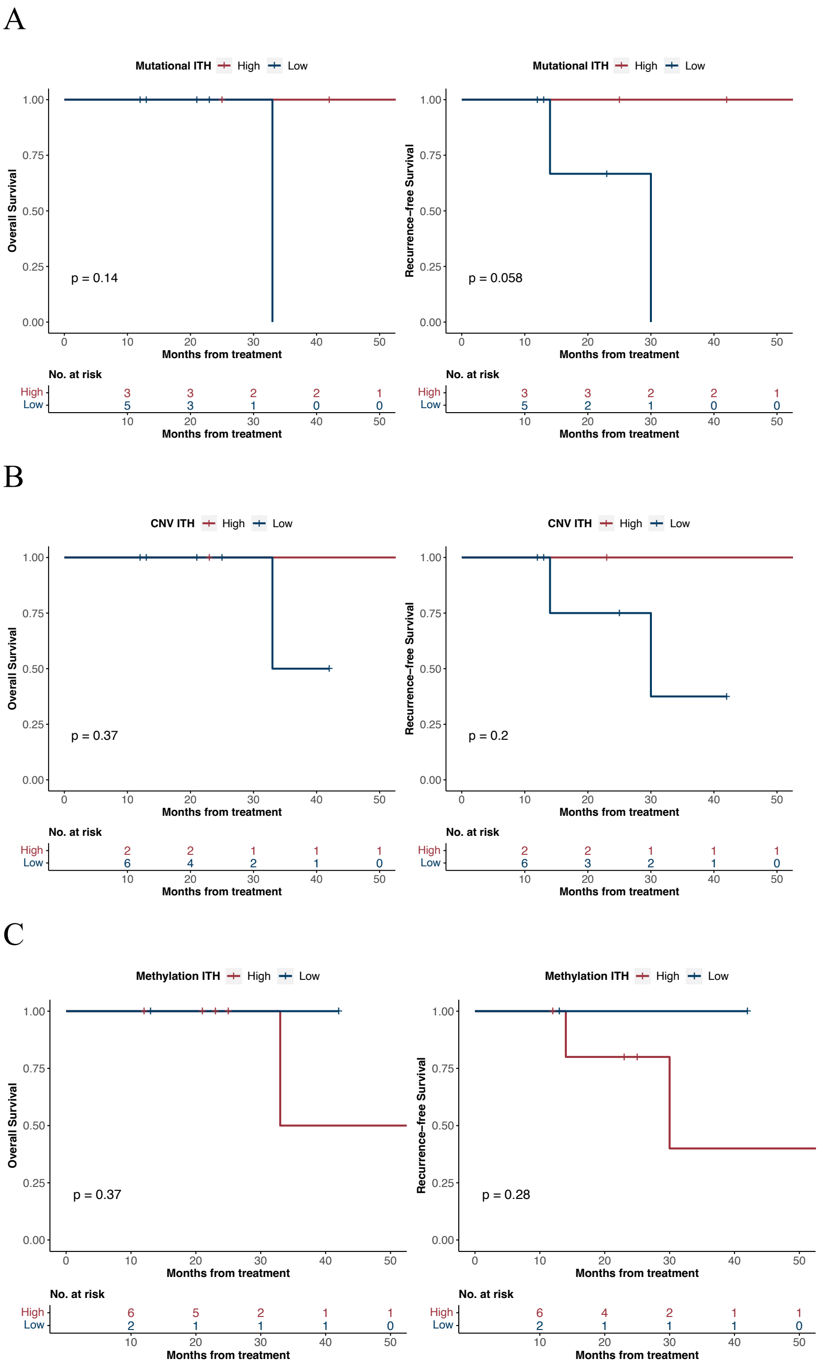

Supplementary Fig 13 Kaplan–Meier curve of recurrence-free survival (DFS) and overall survival (OS) in mixed H-FLAC patients (n=8) stratified by median mutational ITH (a), CNV ITH (v) and methylation ITH (c).

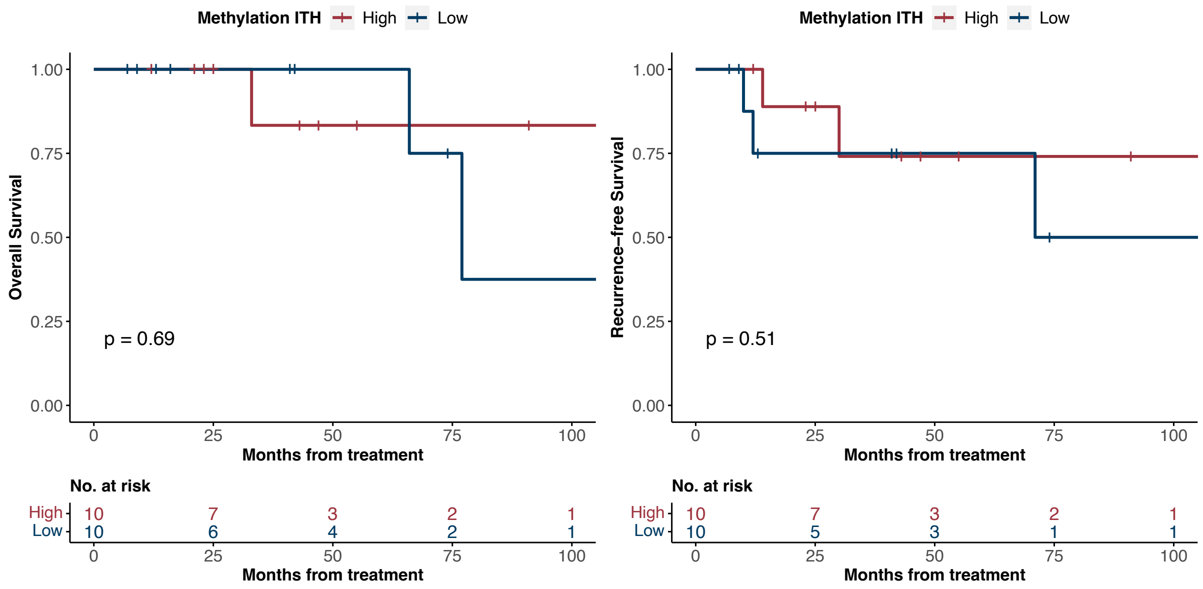

Supplementary Fig 14 Kaplan–Meier curve of recurrence-free survival (DFS) and overall survival (OS) in all FLAC patients (n=20) stratified by median mutational methylation ITH.

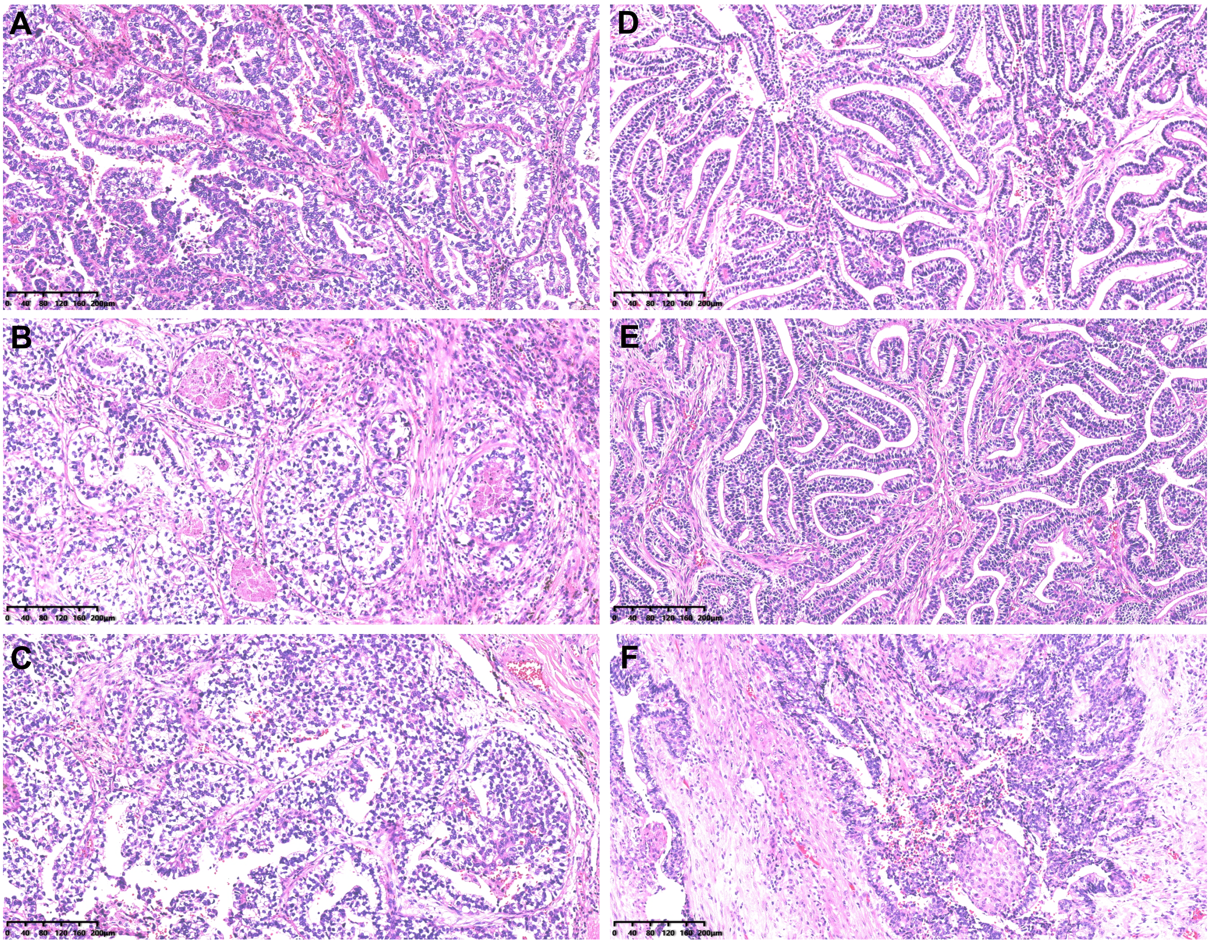

Supplementary Fig 15 Morphologic features of H-FLAC (**a-c**) and L-FLAC (**d-f**) revealed by H&E staining. **a-c** Three regions from tumor specimen of P5 (mixed H-FLAC). Complex glandular structures composed of columnar cells with clear cytoplasm and pseudostratified nuclei resembling fetal lung tubules (**a**), multifocal necrosis (**b**) and miscellaneous histological components (**c**). **d-f** Three regions from tumor specimen of P18 (L-FLAC). Complex glandular structures lined with glycogen-rich columnar cells resembling the developing fetal lung (**d-e**), with low nuclear atypia, and morule formation (**f**).

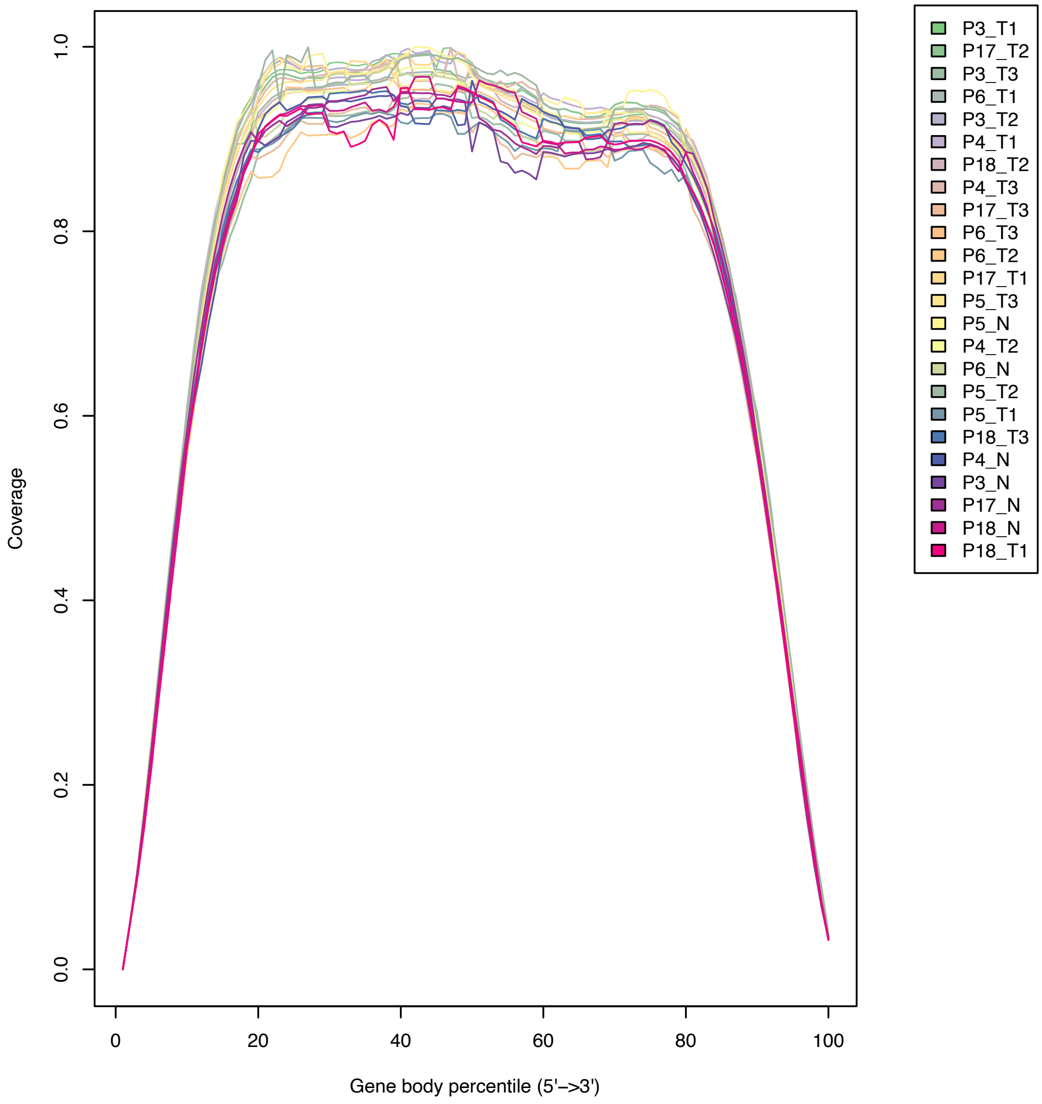

Supplementary Fig 16 Coverage uniformity over gene body. All transcripts were scaled into 100 nt.

Supplementary Data 1. Patient characteristics

Patient	Sex	Age	Pathological classification	Fetal component(%)	Adjuvant Therapy	Recurrence	Recurrence-free survival (Months)	Death	Overall survival (Months)	Smoking	Alcohol	Tumor Size (cm)	T Stage	N Stage	Stage
P1	M	68	mixed H-FLAC	70	No	No	23	No	23	Yes	Yes	2	T1	N0	I
P2	M	73	mixed H-FLAC	80	chemotherapy	Metastasis	14	No	21	Yes	Yes	7	T1	N0	IIIA
P3	M	67	pure H-FLAC	100	chemotherapy	Metastasis	10	No	16	Yes	No	9	T4	N2	IIIB
P4	M	64	mixed H-FLAC	90	No	No	12	No	12	No	No	1.8	T4	N1	I
P5	M	62	mixed H-FLAC	80	No	No	13	No	13	Yes	Yes	2.3	T1	N0	I
P6	M	58	L-FLAC	100	No	No	7	No	7	Yes	No	1.3	T3	N0	I
P7	M	47	L-FLAC	100	No	No	107	No	107	Yes	No	3	T2	N0	I
P8	M	55	L-FLAC	100	No	No	106	No	106	Yes	Yes	2.5	T1	N0	IIB
P9	F	69	L-FLAC	100	No	No	47	No	47	No	No	2	T2	N0	I
P10	M	64	mixed H-FLAC	80	chemotherapy	Metastasis	30	Yes	33	Yes	Yes	7	T2	N0	IIIA
P11	M	63	pure H-FLAC	100	No	No	9	No	9	Yes	Yes	5	T1	N0	I
P12	M	40	L-FLAC	100	No	No	91	No	91	Yes	Yes	4.5	T1	N0	IIA
P13	M	31	L-FLAC	100	chemotherapy	No	74	No	74	Yes	Yes	3.5	T1	N0	I
P14	M	68	pure H-FLAC	100	chemoradiotherapy	Metastasis	12	Yes	66	Yes	No	1.5	T2	N0	I
P15	F	35	mixed H-FLAC	90	No	No	55	No	55	No	No	4.5	T2	N0	I
P16	F	73	pure H-FLAC	100	chemotherapy	Metastasis	71	Yes	77	No	No	2.2	T2	N0	I
P17	F	81	L-FLAC	100	No	No	41	No	41	No	No	3	T3	N0	I
P18	M	41	L-FLAC	100	No	No	43	No	43	Yes	No	3.5	T1	N0	I
P19	F	56	mixed H-FLAC	80	No	No	42	No	42	Yes	No	2.4	T4	N0	I
P20	M	57	mixed H-FLAC	70	No	No	25	No	25	Yes	No	5.3	T1	N0	IIB

Supplementary Data 2. Somatic mutations

PatientID	Hugo_Symbol	Chromosome	Start_Position	End_Position	Variant_Classification	Variant_Type	Reference_Allele	Tumor_Seq_Allele2	T1	T2	T3
P1	NOL9	1	6592759	6592759	Missense_Mutation	SNP	C	A	12.24%	3.23%	1.20%
P1	C1orf167	1	11835551	11835551	Missense_Mutation	SNP	C	A	12.90%		5.10%
P1	KIAA0754	1	39876594	39876594	Silent	SNP	T	C	20.69%	20.00%	23.68%
P1	DNAJC6	1	65855063	65855063	Missense_Mutation	SNP	A	G	14.63%	17.39%	2.99%
P1	NEGR1	1	72076799	72076799	Missense_Mutation	SNP	C	A	11.27%	5.05%	4.72%
P1	ZZZ3	1	78097839	78097839	Missense_Mutation	SNP	C	T	17.46%	3.12%	4.35%
P1	KIFAP3	1	170007430	170007430	Splice_Site	SNP	C	A	19.05%	7.41%	3.12%
P1	LRRN2	1	204587528	204587528	Silent	SNP	G	A	8.33%		4.32%
P1	SUSD4	1	223438081	223438081	Silent	SNP	C	T	23.08%	4.88%	4.92%
P1	SULT6B1	2	37398711	37398711	Missense_Mutation	SNP	T	A	21.95%		13.33%
P1	ALMS1	2	73718595	73718595	Missense_Mutation	SNP	A	G	26.47%	10.26%	12.28%
P1	ST6GAL2	2	107459857	107459857	Missense_Mutation	SNP	C	A	46.51%	25.00%	28.42%
P1	MAP3K19	2	135743391	135743391	Missense_Mutation	SNP	C	A	20.93%		6.38%
P1	NEB	2	152553718	152553718	Missense_Mutation	SNP	G	T	17.65%	6.00%	
P1	SCN2A	2	166246204	166246204	Missense_Mutation	SNP	C	G	23.85%	10.64%	8.26%
P1	XIRP2	2	168107401	168107401	Missense_Mutation	SNP	A	G	17.65%	12.73%	4.26%
P1	LSMEM2	3	50324227	50324227	Silent	SNP	C	T	10.71%		3.78%
P1	ERC2	3	56330314	56330314	Missense_Mutation	SNP	C	A	31.37%	25.40%	11.31%
P1	MAGI1	3	65433718	65433718	Nonsense_Mutation	SNP	C	A	19.15%	2.38%	6.25%
P1	UMPS	3	124456609	124456609	Missense_Mutation	SNP	C	T	9.21%		
P1	EPHB1	3	134885806	134885806	Missense_Mutation	SNP	G	A	25.71%		
P1	HLTF	3	148778454	148778454	Missense_Mutation	SNP	A	G	12.12%		
P1	MAGEF1	3	184429045	184429045	Missense_Mutation	SNP	C	T	23.81%	22.73%	8.33%
P1	RTP1	3	186917407	186917407	Missense_Mutation	SNP	T	A	51.69%	27.27%	29.93%
P1	GAK	4	887198	887198	Silent	SNP	G	T	13.79%	6.25%	7.29%
P1	PCDH7	4	30723153	30723153	Silent	SNP	C	A	8.33%		5.26%
P1	ADH1B	4	100235175	100235175	Missense_Mutation	SNP	C	T	10.29%		2.15%
P1	ANK2	4	113970938	113970938	Silent	SNP	C	A	14.29%	2.74%	9.70%
P1	SEC24D	4	119665305	119665305	Missense_Mutation	SNP	G	C	17.50%	6.15%	7.53%
P1	FHIP1A	4	152571509	152571509	Missense_Mutation	SNP	G	C	20.00%	4.55%	4.39%
P1	SPEF2	5	35712972	35712972	Silent	SNP	C	G	9.38%	3.39%	3.61%
P1	HCN1	5	45262519	45262519	Missense_Mutation	SNP	T	A	12.50%	25.00%	8.79%
P1	ADGRV1	5	89925114	89925115	Missense_Mutation	DNP	GG	TT	23.33%	9.76%	6.12%
P1	ZNF608	5	124080604	124080604	Missense_Mutation	SNP	C	T	9.23%		
P1	LMNB1	5	126145989	126145989	Missense_Mutation	SNP	G	A	18.92%	15.38%	10.34%
P1	PCDHA6	5	140207965	140207965	Missense_Mutation	SNP	G	C	7.14%		4.82%
P1	STK19	6	31939878	31939878	Silent	SNP	C	T	61.54%	40.00%	16.54%
P1	DAAM2	6	39824094	39824094	Missense_Mutation	SNP	A	G	20.69%	26.67%	9.84%
P1	DAAM2	6	39869751	39869751	Nonsense_Mutation	SNP	C	T	13.56%	9.52%	5.79%
P1	HIVEP2	6	143093295	143093295	Missense_Mutation	SNP	C	A	28.12%	15.79%	24.49%
P1	PLG	6	161152906	161152906	Missense_Mutation	SNP	G	T	19.35%	2.94%	3.75%
P1	EIF3B	7	2412412	2412412	Missense_Mutation	SNP	G	T	10.14%	3.92%	7.48%
P1	FBXL18	7	5541269	5541269	Missense_Mutation	SNP	C	A	44.59%	17.39%	22.09%
P1	SP8	7	20824679	20824679	Missense_Mutation	SNP	G	T	8.45%	13.73%	5.32%
P1	DNAH11	7	21737825	21737825	Silent	SNP	C	G	9.09%	14.58%	13.16%
P1	KIAA0895	7	36397059	36397059	Missense_Mutation	SNP	T	A	38.89%	17.54%	31.65%
P1	IKZF1	7	50450246	50450246	Missense_Mutation	SNP	C	G	12.00%		3.18%
P1	FIGNL1	7	50513218	50513218	Missense_Mutation	SNP	C	T	54.84%	7.41%	30.43%
P1	GUSB	7	65446966	65446966	Missense_Mutation	SNP	A	T	11.67%		4.67%
P1	SEMA3E	7	83098597	83098597	Missense_Mutation	SNP	G	T	11.11%	5.66%	7.14%
P1	GRM3	7	86416026	86416027	Frame_Shift_Del	DEL	G	-	8.11%	7.69%	5.78%
P1	HEPACAM2	7	92848475	92848475	Silent	SNP	C	T	15.09%		2.63%
P1	TMEM229A	7	123672305	123672306	Missense_Mutation	DNP	GC	AA	6.33%	13.33%	6.02%
P1	METTL2B	7	128119379	128119380	Missense_Mutation	DNP	TG	CC	4.76%	9.68%	5.56%
P1	AOC1	7	150554104	150554104	Silent	SNP	G	T	16.67%		13.85%
P1	NCAPG2	7	158448088	158448088	Silent	SNP	T	A	33.33%	11.94%	18.47%
P1	ZFHX4	8	77617017	77617017	Missense_Mutation	SNP	C	T	16.67%		4.76%
P1	RUNX1T1	8	93088235	93088235	Missense_Mutation	SNP	T	G	14.04%		
P1	ADCY8	8	131896837	131896837	Missense_Mutation	SNP	C	A	20.00%	2.78%	7.25%
P1	ZNF707	8	144776173	144776173	Missense_Mutation	SNP	G	T	11.27%	8.70%	0.86%
P1	SPATC1	8	145095095	145095095	Missense_Mutation	SNP	C	T	14.00%	9.09%	4.61%
P1	FKBP15	9	115935829	115935829	Missense_Mutation	SNP	C	G	10.00%	16.67%	13.33%
P1	OR1L8	9	125330046	125330046	Missense_Mutation	SNP	T	A	19.35%	13.16%	6.67%
P1	TOR2A	9	130496605	130496605	Silent	SNP	G	A	12.82%	3.57%	12.70%
P1	EBLN1	10	22498710	22498710	Missense_Mutation	SNP	T	A	13.79%	4.35%	1.79%
P1	PIP4K2A	10	22830781	22830781	Missense_Mutation	SNP	C	A	16.00%	5.26%	7.87%
P1	BMS1	10	43292147	43292147	Silent	SNP	A	G	15.00%	17.54%	9.17%
P1	RBP3	10	48389681	48389681	Silent	SNP	G	T	18.75%		9.17%
P1	LRRC4C	11	40137551	40137551	Missense_Mutation	SNP	G	T	17.07%	2.56%	4.44%
P1	OR8H1	11	56058000	56058000	Missense_Mutation	SNP	G	C	16.67%		12.07%
P1	GLYATL1B	11	58861100	58861100	Missense_Mutation	SNP	A	G	11.76%	6.25%	12.50%
P1	SCGB1D2	11	62010780	62010780	Silent	SNP	A	C	12.77%	6.06%	1.49%
P1	FBXL14	12	1702451	1702451	Missense_Mutation	SNP	C	A	13.33%		10.07%
P1	STAT2	12	56742809	56742809	Missense_Mutation	SNP	G	T	14.81%	7.69%	10.71%
P1	SRGAP1	12	64521492	64521492	Missense_Mutation	SNP	G	A	13.89%	7.14%	9.52%
P1	OTOGL	12	80658968	80658968	Missense_Mutation	SNP	C	A	30.00%	12.00%	19.35%
P1	SETD1B	12	122248172	122248172	Nonsense_Mutation	SNP	G	T	9.88%	3.85%	2.24%

P1	NUP58	13	25887767	25887767	Missense_Mutation	SNP	C	T	14.00%	7.32%	5.17%
P1	SPART	13	36886330	36886330	Missense_Mutation	SNP	C	T	9.68%		
P1	PCDH17	13	58298993	58298993	Silent	SNP	T	G	21.05%	11.54%	6.45%
P1	ABCC4	13	95725504	95725504	Missense_Mutation	SNP	C	A	14.71%		11.54%
P1	COL4A1	13	110829018	110829018	Missense_Mutation	SNP	C	T	40.00%	5.88%	12.28%
P1	ASB2	14	94405939	94405939	Missense_Mutation	SNP	C	T	25.00%	11.90%	3.49%
P1	SERPINA9	14	94936088	94936088	Nonsense_Mutation	SNP	G	T	17.65%	13.33%	9.88%
P1	SERPINA5	14	95054157	95054157	Missense_Mutation	SNP	A	T	38.89%	37.50%	18.75%
P1	RYR3	15	34078158	34078158	Silent	SNP	G	A	24.32%		9.09%
P1	THSD4	15	72021017	72021017	Missense_Mutation	SNP	A	T	20.00%	4.35%	12.33%
P1	ZNF592	15	85327839	85327839	Missense_Mutation	SNP	G	T	18.92%	9.09%	3.31%
P1	CCDC154	16	1493628	1493628	Missense_Mutation	SNP	C	A	15.00%	2.63%	6.45%
P1	PRKCB	16	23847559	23847559	Silent	SNP	C	A	17.07%	10.71%	2.22%
P1	CNTNAP4	16	76486500	76486500	Missense_Mutation	SNP	G	C	9.84%	11.29%	6.49%
P1	C16orf46	16	81095011	81095011	Missense_Mutation	SNP	G	T	11.76%	16.67%	9.82%
P1	CTU2	16	88779096	88779096	Missense_Mutation	SNP	T	C	8.62%	7.69%	4.72%
P1	TP53	17	7578263	7578263	Nonsense_Mutation	SNP	G	A	28.12%		20.37%
P1	GUCY2D	17	7918015	7918015	Missense_Mutation	SNP	G	A	9.68%	7.14%	8.24%
P1	MYH8	17	10293800	10293800	Missense_Mutation	SNP	C	G	8.70%	10.53%	6.94%
P1	RNF135	17	29324324	29324324	Silent	SNP	C	T	29.03%	25.00%	22.50%
P1	GPATCH8	17	42475562	42475562	Missense_Mutation	SNP	C	T	17.65%	11.11%	14.63%
P1	SEPTIN9	17	75489112	75489112	Silent	SNP	C	T	18.75%		13.33%
P1	MC5R	18	13826036	13826036	Nonsense_Mutation	SNP	G	A	20.00%		6.12%
P1	ELOA2	18	44559422	44559422	Silent	SNP	C	T	11.63%	11.11%	6.67%
P1	SERPINB3	18	61323033	61323033	Missense_Mutation	SNP	G	T	22.58%	10.71%	12.77%
P1	SERPINB3	18	61324127	61324127	Missense_Mutation	SNP	A	T	11.76%	18.92%	3.45%
P1	PNPLA6	19	7607916	7607916	Missense_Mutation	SNP	A	C	11.39%		3.40%
P1	JAK3	19	17949126	17949126	Silent	SNP	T	C	41.18%	4.17%	16.22%
P1	ZNF726	19	24102293	24102293	Missense_Mutation	SNP	C	T	8.00%		3.57%
P1	FFAR3	19	35849972	35849972	Silent	SNP	G	A	10.96%	6.67%	6.79%
P1	WDR87	19	38377091	38377091	Missense_Mutation	SNP	G	T	6.45%	1.43%	0.51%
P1	ACTN4	19	39214813	39214813	Missense_Mutation	SNP	A	G	40.87%	24.00%	27.88%
P1	CPT1C	19	50214011	50214011	Missense_Mutation	SNP	C	T	38.46%	37.50%	17.78%
P1	SIGLEC10	19	51914556	51914556	Missense_Mutation	SNP	G	T	16.13%	9.23%	8.06%
P1	TMEM150B	19	55824279	55824279	Missense_Mutation	SNP	G	T	32.65%	4.35%	18.71%
P1	ZSCAN5B	19	56704243	56704243	Missense_Mutation	SNP	T	A	46.15%	31.82%	24.30%
P1	NINL	20	25493490	25493490	Missense_Mutation	SNP	C	A	11.84%		5.32%
P1	SON	21	34918578	34918578	Missense_Mutation	SNP	C	T	18.75%	12.50%	1.75%
P1	AIRE	21	45711081	45711081	Missense_Mutation	SNP	G	A	12.28%		13.57%
P1	POFUT2	21	46696957	46696957	Missense_Mutation	SNP	A	C	7.23%		5.78%
P1	TRMT2A	22	20103967	20103967	Missense_Mutation	SNP	C	T	5.49%		3.61%
P1	SLC7A4	22	21385622	21385622	Nonsense_Mutation	SNP	C	T	7.81%		7.69%
P1	SLC5A1	22	32464500	32464500	Missense_Mutation	SNP	G	T	21.95%	7.69%	3.17%
P1	DLG3	X	69669598	69669598	Missense_Mutation	SNP	C	T	23.33%		7.74%
P1	AIFM1	X	129263995	129263995	Missense_Mutation	SNP	C	A	25.93%	54.17%	13.95%
P1	MPP1	X	154020557	154020557	Missense_Mutation	SNP	C	A	17.14%		7.50%
P1	SPEN	1	16261923	16261923	Missense_Mutation	SNP	C	T	24.44%	21.05%	4.08%
P1	AGO4	1	36319170	36319170	Silent	SNP	C	A	26.67%	14.58%	10.00%
P1	ADGRL2	1	82415951	82415951	Missense_Mutation	SNP	A	C	16.67%	12.50%	5.56%
P1	HFM1	1	91784849	91784849	Splice_Site	SNP	C	G	6.45%	9.09%	14.29%
P1	MTMR11	1	149906888	149906888	Missense_Mutation	SNP	C	G	3.70%		16.28%
P1	DDR2	1	162743323	162743323	Missense_Mutation	SNP	G	A	15.15%	9.43%	12.12%
P1	PRG4	1	186277562	186277562	Missense_Mutation	SNP	C	T	33.33%	17.91%	8.33%
P1	ASPM	1	197071708	197071708	Missense_Mutation	SNP	C	G	26.32%	9.84%	8.24%
P1	USH2A	1	215848300	215848300	Missense_Mutation	SNP	T	A	36.36%		24.24%
P1	OR2T34	1	248737845	248737845	Missense_Mutation	SNP	C	T	4.35%	35.00%	12.73%
P1	EML6	2	55096427	55096427	Missense_Mutation	SNP	A	T	20.75%	15.38%	25.64%
P1	FER1L5	2	97361308	97361308	Silent	SNP	C	T	7.14%		10.67%
P1	CPO	2	207823062	207823062	Missense_Mutation	SNP	G	T	19.30%	10.64%	5.63%
P1	ABCA12	2	215812249	215812249	Missense_Mutation	SNP	A	T	43.24%	26.92%	15.79%
P1	C4orf54	4	100574201	100574201	Silent	SNP	G	A	21.15%		6.36%
P1	CFI	4	110670716	110670716	Missense_Mutation	SNP	C	G	56.00%	5.56%	21.21%
P1	ANK2	4	114290761	114290761	Missense_Mutation	SNP	C	A	39.39%	14.71%	17.78%
P1	DCHS2	4	155157514	155157514	Missense_Mutation	SNP	C	A	22.73%	17.39%	7.59%
P1	PCDHB4	5	140502896	140502896	Missense_Mutation	SNP	A	T	1.36%	1.25%	2.02%
P1	CYFIP2	5	156787351	156787351	Missense_Mutation	SNP	G	T	36.36%	7.69%	22.22%
P1	SH3PXD2B	5	171765537	171765537	Missense_Mutation	SNP	C	A	37.04%	21.43%	11.69%
P1	LY6G6F	6	31675867	31675867	Missense_Mutation	SNP	G	T	5.88%	10.00%	7.89%
P1	KIF6	6	39554107	39554107	Missense_Mutation	SNP	C	G	27.59%	5.56%	11.76%
P1	GPRC6A	6	117113976	117113976	Missense_Mutation	SNP	G	A	5.88%	12.00%	13.79%
P1	TAAR6	6	132891596	132891596	Missense_Mutation	SNP	G	A	3.77%	3.70%	10.77%
P1	TMEM196	7	19765324	19765324	Missense_Mutation	SNP	C	G	21.67%	5.41%	10.42%
P1	GLI3	7	42017269	42017269	Nonsense_Mutation	SNP	A	T	37.50%	11.11%	9.20%
P1	MUC12	7	100635574	100635574	Missense_Mutation	SNP	A	C	0.85%	12.94%	4.44%
P1	ZNF425	7	148801530	148801530	Missense_Mutation	SNP	C	A	9.09%	7.14%	3.19%
P1	PTPRN2	7	157874059	157874059	Missense_Mutation	SNP	G	T	3.23%		14.29%
P1	SFMBT2	10	7214537	7214537	Missense_Mutation	SNP	G	A	1.75%		5.96%
P1	PGGHG	11	294187	294187	Missense_Mutation	SNP	C	T	48.94%	9.52%	26.74%
P1	NRIP3	11	9009789	9009789	Missense_Mutation	SNP	T	A	40.00%	25.81%	18.18%

P1	LRRC4C	11	40137521	40137521	Missense_Mutation	SNP	T	A	25.00%	8.57%	11.36%
P1	OR5M10	11	56345046	56345046	Missense_Mutation	SNP	G	T	29.31%	18.37%	9.78%
P1	GLYATL1	11	58722666	58722666	Missense_Mutation	SNP	G	A	31.82%	13.64%	11.11%
P1	SLC22A25	11	62985098	62985098	Missense_Mutation	SNP	C	A	3.70%	2.08%	9.86%
P1	FAT3	11	92600123	92600123	Missense_Mutation	SNP	G	T	27.66%	11.11%	24.43%
P1	PARP4	13	25009467	25009467	Missense_Mutation	SNP	G	A	22.73%	10.42%	11.63%
P1	PRPF39	14	45578927	45578927	Missense_Mutation	SNP	G	T	33.33%	17.39%	10.87%
P1	GOLGA8K	15	32689678	32689678	Silent	SNP	C	T	6.90%		14.55%
P1	TMEM87A	15	42553179	42553179	Missense_Mutation	SNP	G	A	37.14%	9.09%	15.38%
P1	ZNF768	16	30535981	30535981	Frame_Shift_Ins	INS	-	C	2.60%	5.88%	4.27%
P1	ZNF768	16	30535982	30535982	Silent	SNP	G	C	2.60%	5.88%	4.27%
P1	UNC119	17	26875638	26875638	Silent	SNP	G	C	37.93%	15.79%	12.73%
P1	HSD17B1	17	40705145	40705145	Missense_Mutation	SNP	A	T	21.54%	5.88%	5.65%
P1	PIEZO2	18	10705528	10705528	Silent	SNP	G	A	3.57%		3.40%
P1	SPACA6	19	52207344	52207344	Missense_Mutation	SNP	G	T	7.03%	3.70%	2.62%
P1	NLRP12	19	54313534	54313534	Missense_Mutation	SNP	C	G	6.67%	11.76%	7.77%
P1	SSC5D	19	56005026	56005026	Nonsense_Mutation	SNP	G	A	35.24%	25.86%	21.08%
P1	ZIM2	19	57286106	57286106	Missense_Mutation	SNP	G	T	10.71%	4.35%	11.32%
P1	PEG3	19	57335705	57335705	Nonsense_Mutation	SNP	G	A	2.44%		22.95%
P1	CHAF1B	21	37785569	37785569	Missense_Mutation	SNP	G	T	24.00%		5.34%
P1	PIWIL3	22	25150042	25150043	Frame_Shift_Del	DEL	G	-	8.70%		11.43%
P1	MORC2	22	31335990	31335990	Missense_Mutation	SNP	T	C	8.33%	32.00%	17.02%
P1	GATA1	X	48649591	48649591	Silent	SNP	C	A	60.00%		13.04%
P1	RYR2	1	237586428	237586428	Missense_Mutation	SNP	C	A	43.33%	20.51%	34.21%
P1	MITF	3	69928417	69928417	Missense_Mutation	SNP	G	T	33.33%	22.58%	12.70%
P1	ADPRH	3	119305465	119305465	Missense_Mutation	SNP	A	G	32.65%	16.67%	14.71%
P1	PLSCR4	3	145924439	145924439	Silent	SNP	A	T	17.86%	18.60%	
P1	LDB2	4	16513695	16513695	Silent	SNP	C	T	23.68%	14.29%	3.57%
P1	LAMA4	6	112476897	112476897	Missense_Mutation	SNP	C	A	43.40%	21.57%	2.90%
P1	TRPS1	8	116631447	116631447	Missense_Mutation	SNP	T	C	34.43%	22.73%	17.46%
P1	DLG2	11	83252862	83252862	Silent	SNP	C	A	5.56%	18.75%	10.00%
P1	BARX2	11	129321222	129321222	Silent	SNP	C	A	52.38%	22.58%	20.73%
P1	PHETA1	12	111800581	111800581	Silent	SNP	C	A	22.22%	20.59%	14.86%
P1	ZNF100	19	21926903	21926903	Missense_Mutation	SNP	T	A	4.49%	10.17%	
P1	EDEM1	3	5249945	5249945	Nonsense_Mutation	SNP	C	A			7.14%
P1	ATP13A5	3	193002730	193002730	Missense_Mutation	SNP	G	T			11.86%
P1	KIAA0368	9	114131322	114131322	Silent	SNP	C	T			9.09%
P1	ERC1	12	1291204	1291204	Silent	SNP	G	A			6.82%
P1	SOGA1	20	35445830	35445830	Missense_Mutation	SNP	C	T			6.30%
P1	MOV10L1	22	50589322	50589322	Silent	SNP	C	T			7.87%
P1	ZNF735	7	63674420	63674420	Missense_Mutation	SNP	G	A		7.21%	2.73%
P1	FAM25A	10	88784354	88784354	Missense_Mutation	SNP	A	G		6.82%	2.08%
P1	HEATR6	17	58145052	58145052	Silent	SNP	C	T		5.04%	
P2	INTS11	1	1254853	1254853	Silent	SNP	C	T	3.53%	10.53%	6.25%
P2	NPR1	1	153662449	153662449	Missense_Mutation	SNP	C	G		5.22%	
P2	JMJD4	1	227920324	227920324	Silent	SNP	G	C	2.44%	15.38%	2.53%
P2	CTNNB1	3	41266097	41266097	Missense_Mutation	SNP	G	A	13.95%	14.81%	5.41%
P2	COL6A6	3	130284225	130284225	Missense_Mutation	SNP	A	C	18.42%	11.11%	18.18%
P2	TLR6	4	38828713	38828713	Silent	SNP	C	T	18.18%	23.81%	18.18%
P2	PRRT1	6	32118502	32118502	Silent	SNP	G	A	29.92%	33.02%	25.95%
P2	ARMC10	7	102715737	102715737	Silent	SNP	C	T		15.38%	
P2	SHOC1	9	114500703	114500703	Missense_Mutation	SNP	G	C		7.79%	
P2	GLI1	12	57859612	57859612	Missense_Mutation	SNP	G	C	21.05%	29.17%	24.24%
P2	SACS	13	23910626	23910626	Silent	SNP	A	T	8.77%	18.42%	11.54%
P2	NTAN1	16	15141742	15141742	Missense_Mutation	SNP	T	G	37.78%	29.03%	16.67%
P2	OR4D1	17	56233437	56233437	Missense_Mutation	SNP	G	T	9.52%	36.36%	20.00%
P2	CCDC8	19	46914700	46914700	Silent	SNP	G	C	18.84%	21.62%	22.22%
P2	ANKRD60	20	56796518	56796518	Silent	SNP	G	A	13.21%	21.62%	24.32%
P2	NELFCD	20	57564970	57564970	Silent	SNP	C	T		5.17%	
P2	MEGF6	1	3415775	3415775	Silent	SNP	G	A	4.95%	12.94%	3.49%
P2	RPE65	1	68912434	68912434	Missense_Mutation	SNP	G	C	9.38%	33.90%	32.76%
P2	PDZK1	1	145748542	145748542	Missense_Mutation	SNP	G	A	24.49%	28.95%	35.42%
P2	WDR64	1	241959568	241959568	Missense_Mutation	SNP	C	T	7.14%	24.19%	16.67%
P2	UNC80	2	210858961	210858961	Silent	SNP	G	A	15.38%	4.65%	
P2	XIRP1	3	39228897	39228897	Silent	SNP	C	T	11.11%	9.68%	16.99%
P2	GABRB1	4	47427696	47427696	Silent	SNP	C	T	26.98%	39.06%	32.03%
P2	ANK2	4	114278629	114278629	Missense_Mutation	SNP	C	G	7.07%	30.00%	12.22%
P2	GPX5	6	28500105	28500105	Missense_Mutation	SNP	C	A	14.29%	28.57%	29.73%
P2	GRM1	6	146755239	146755239	Silent	SNP	G	A	29.10%	35.06%	28.99%
P2	KRTAP5-9	11	71259886	71259886	Silent	SNP	C	T	21.05%	38.71%	37.27%
P2	SPESP1	15	69238442	69238442	Missense_Mutation	SNP	T	C	9.80%	25.00%	22.22%
P2	GDPD2	X	69646530	69646530	Silent	SNP	C	T	14.29%	10.53%	22.39%
P2	TENT5D	X	79698542	79698542	Missense_Mutation	SNP	G	T	14.00%	44.12%	23.08%
P2	RD3	1	211654686	211654686	Missense_Mutation	SNP	C	G	15.84%	43.55%	32.85%
P2	BRAP	12	112103531	112103531	Missense_Mutation	SNP	G	A	15.22%	21.92%	12.00%
P2	RILPL1	12	124017898	124017898	Silent	SNP	C	T	30.30%	32.35%	29.13%
P2	ATP8A2	13	26116184	26116184	Missense_Mutation	SNP	G	C	16.39%	6.67%	16.33%
P2	TMEM132E	17	32953491	32953491	Missense_Mutation	SNP	C	G		1.59%	4.41%
P2	KLHL17	1	899873	899873	Missense_Mutation	SNP	G	A	3.92%		

P2	BCL11A	2	60688247	60688247	Silent	SNP	G	A	3.93%		
P2	SORCS3	10	106974243	106974243	Missense_Mutation	SNP	G	A	7.79%		
P2	EMSY	11	76169296	76169296	Silent	SNP	G	A	9.68%		
P2	ZMYM4	1	35881097	35881097	Missense_Mutation	SNP	A	T			6.90%
P2	TLR3	4	187003955	187003955	Missense_Mutation	SNP	G	T			14.00%
P2	CTRL	16	67964672	67964672	Missense_Mutation	SNP	C	T			3.14%
P2	AKAP17A	X	1718255	1718255	Missense_Mutation	SNP	G	A			1.21%
P3	CASZ1	1	10713945	10713945	Silent	SNP	G	A	6.49%	4.46%	8.84%
P3	PRAMEF11	1	12888482	12888482	Silent	SNP	C	G	14.55%	3.51%	21.59%
P3	FNBP1L	1	94000337	94000337	Missense_Mutation	SNP	C	G	12.86%	7.35%	2.56%
P3	KIRREL1	1	158063499	158063499	Missense_Mutation	SNP	G	A	5.64%	4.32%	5.83%
P3	SUCO	1	172555015	172555015	Missense_Mutation	SNP	G	A	19.77%	10.81%	4.26%
P3	RNASEL	1	182544605	182544605	Silent	SNP	G	A	10.91%	7.25%	
P3	LEFTY1	1	226076763	226076763	Missense_Mutation	SNP	G	T	7.62%	13.64%	1.47%
P3	ASAP2	2	9519104	9519104	Missense_Mutation	SNP	A	C	53.61%	37.25%	32.18%
P3	LRATD1	2	14774742	14774742	Missense_Mutation	SNP	G	T	7.08%	7.55%	5.48%
P3	EFR3B	2	25358440	25358440	Silent	SNP	C	T	21.95%	11.32%	
P3	ZNF513	2	27602969	27602969	Missense_Mutation	SNP	C	G	9.14%	4.23%	14.78%
P3	PRKD3	2	37481395	37481395	Missense_Mutation	SNP	C	G	4.55%	10.45%	
P3	USP34	2	61510585	61510585	Splice_Site	SNP	C	A	5.10%	13.43%	5.80%
P3	PSD4	2	113950138	113950138	Missense_Mutation	SNP	T	A	40.00%	42.50%	39.62%
P3	TTN	2	179569471	179569471	Missense_Mutation	SNP	T	C	4.55%	12.77%	4.76%
P3	CWC22	2	180810187	180810187	Missense_Mutation	SNP	C	G	4.61%	3.73%	5.76%
P3	OXSM	3	25835730	25835730	Silent	SNP	A	T	10.53%	11.32%	3.23%
P3	EPHA3	3	89259498	89259498	Silent	SNP	C	T	10.20%	12.96%	
P3	IL20RB	3	136729020	136729020	Silent	SNP	G	A	16.67%	12.12%	
P3	EEF1AKMT4-ECE2	3	183995973	183995973	Missense_Mutation	SNP	A	T	83.33%	65.62%	50.00%
P3	IDUA	4	996873	996873	Silent	SNP	C	T	8.64%	9.26%	2.33%
P3	RGS12	4	3344721	3344721	Missense_Mutation	SNP	C	T	17.11%	10.81%	17.81%
P3	FBXW7	4	153332502	153332518	Frame_Shift_Del	DEL	GGTCCACAATACTACT	-	62.50%	60.26%	33.33%
P3	NRN1	6	6002692	6002692	Missense_Mutation	SNP	C	T	3.76%	4.46%	1.52%
P3	HLA-A	6	29910607	29910607	Silent	SNP	G	C	1.86%	2.51%	
P3	ZNF316	7	6693674	6693674	Missense_Mutation	SNP	C	T	5.83%	9.26%	
P3	GRM3	7	86394685	86394685	Missense_Mutation	SNP	C	A	3.41%	12.07%	10.81%
P3	TPK1	7	144532671	144532671	Nonsense_Mutation	SNP	C	A	9.09%	12.50%	
P3	SLC4A2	7	150772721	150772721	Silent	SNP	C	T	4.43%	7.96%	3.57%
P3	SHH	7	155596099	155596099	Missense_Mutation	SNP	G	A	22.54%	10.53%	25.53%
P3	NEFM	8	24771432	24771432	Silent	SNP	C	T	6.48%	3.70%	15.65%
P3	ZNF703	8	37554839	37554840	Nonsense_Mutation	DNP	GA	CT	6.73%	5.26%	10.20%
P3	VPS13B	8	100147911	100147911	Missense_Mutation	SNP	G	A	14.00%	17.95%	7.50%
P3	OXR1	8	107719075	107719075	Missense_Mutation	SNP	T	A	16.81%	9.46%	21.62%
P3	TEX10	9	103070871	103070871	Silent	SNP	A	G	11.96%	6.56%	25.32%
P3	GRIN3A	9	104500007	104500007	Silent	SNP	A	T	75.00%	63.30%	63.33%
P3	NOTCH1	9	139417337	139417337	Missense_Mutation	SNP	G	A	8.93%	14.58%	1.75%
P3	CELF2	10	11207540	11207540	Missense_Mutation	SNP	C	T	6.32%	9.52%	21.43%
P3	C10orf90	10	128335197	128335197	Missense_Mutation	SNP	G	C	23.08%	14.29%	11.54%
P3	SYT8	11	1857478	1857478	Silent	SNP	G	A	6.12%	4.60%	7.22%
P3	NAA40	11	63719764	63719764	Silent	SNP	G	C		3.85%	
P3	CHEK1	11	125513774	125513774	Missense_Mutation	SNP	C	G	11.11%	6.98%	
P3	KRT7	12	52629013	52629013	Missense_Mutation	SNP	G	T	45.90%	31.86%	19.15%
P3	KCNC2	12	75601633	75601633	Missense_Mutation	SNP	C	G		5.71%	
P3	UTP14C	13	52603470	52603470	Nonsense_Mutation	SNP	G	A	66.29%	58.90%	57.45%
P3	CHAMP1	13	115091014	115091014	Missense_Mutation	SNP	C	T	16.07%	12.96%	12.00%
P3	NPAS3	14	34247679	34247679	Missense_Mutation	SNP	G	A	25.00%	21.21%	
P3	SIX4	14	61180892	61180892	Missense_Mutation	SNP	C	G	14.81%	25.00%	4.65%
P3	GSC	14	95235295	95235295	Missense_Mutation	SNP	C	A	6.67%	8.47%	17.91%
P3	MCTP2	15	94841644	94841644	Silent	SNP	C	T	9.40%	8.60%	2.50%
P3	CBLN1	16	49313455	49313455	Missense_Mutation	SNP	C	T	8.08%	7.27%	1.64%
P3	ZNF423	16	49669696	49669696	Missense_Mutation	SNP	G	A		8.41%	
P3	ELMO3	16	67236661	67236661	Silent	SNP	G	A	47.03%	52.58%	46.59%
P3	TP53	17	7578457	7578457	Missense_Mutation	SNP	C	A	69.39%	65.38%	70.51%
P3	KCNH6	17	61623067	61623067	Missense_Mutation	SNP	C	A	5.95%	4.00%	
P3	AATK	17	79095252	79095252	Silent	SNP	C	T	39.78%	41.54%	32.53%
P3	PLK5	19	1531834	1531834	Silent	SNP	C	T	12.16%	9.38%	2.56%
P3	ACTL9	19	8808008	8808008	Silent	SNP	G	A	21.84%	19.51%	30.00%
P3	DYRK1B	19	40319100	40319100	Missense_Mutation	SNP	C	T		2.36%	
P3	RBM39	20	34312530	34312530	Nonsense_Mutation	SNP	G	A		3.21%	
P3	EYA2	20	45702848	45702848	Missense_Mutation	SNP	G	A	19.42%	20.83%	14.29%
P3	SULF2	20	46386057	46386057	Silent	SNP	G	C	33.53%	34.20%	27.33%
P3	MC3R	20	54824521	54824521	Missense_Mutation	SNP	G	A	3.17%	1.62%	6.83%
P3	DGCR6	22	18894172	18894172	Missense_Mutation	SNP	G	C	18.12%	13.88%	9.82%
P3	EFCAB6	22	44063095	44063095	Silent	SNP	C	T	14.89%	13.89%	
P3	PARVG	22	44581707	44581707	Silent	SNP	G	T	19.05%	40.26%	22.73%
P3	MXRA5	X	3240761	3240761	Missense_Mutation	SNP	C	T	29.03%	23.08%	
P3	DCAF8L1	X	27997871	27997871	Missense_Mutation	SNP	C	A	25.00%	14.81%	
P3	ZNF711	X	84525945	84525945	Missense_Mutation	SNP	T	C	6.45%	13.64%	6.45%
P3	ARMCX2	X	100912489	100912489	Missense_Mutation	SNP	C	T	82.61%	69.05%	56.25%
P3	UTY	Y	15447848	15447848	Missense_Mutation	SNP	T	G	30.77%	22.22%	
P3	GPR89B	1	147415556	147415556	Missense_Mutation	SNP	C	T	34.43%	29.51%	19.15%

P3	DISP1	1	223178548	223178548	Missense_Mutation	SNP	G	A	22.41%	22.78%	16.22%
P3	EPHX1	1	226027664	226027667	In_Frame_Del	DEL	AGG	-	12.32%	8.41%	15.25%
P3	OR2M5	1	248309368	248309368	Nonsense_Mutation	SNP	A	T	9.15%	1.23%	9.76%
P3	RSAD2	2	7036058	7036058	Silent	SNP	G	C	3.61%	1.45%	10.14%
P3	POTEF	2	130832482	130832482	Missense_Mutation	SNP	A	T	5.70%	2.52%	6.38%
P3	ZDHHC23	3	113672800	113672800	Missense_Mutation	SNP	C	G	27.06%	27.63%	18.37%
P3	SI	3	164760880	164760880	Silent	SNP	T	A	40.74%	33.33%	15.79%
P3	PCDHA13	5	140264012	140264012	Missense_Mutation	SNP	C	T	16.80%	18.29%	8.54%
P3	VWC2	7	49815577	49815577	Silent	SNP	G	A	7.19%	1.79%	9.38%
P3	EGFR	7	55242433	55242433	Missense_Mutation	SNP	G	A	27.78%	31.43%	20.51%
P3	BRI3	7	97920492	97920492	Silent	SNP	G	A	7.02%	2.46%	8.20%
P3	WASHC2A	10	51885937	51885937	Nonsense_Mutation	SNP	C	T	47.37%	50.98%	51.72%
P3	CFAP46	10	134648191	134648191	Missense_Mutation	SNP	G	A	6.09%	6.41%	7.41%
P3	TENM4	11	78574105	78574105	Missense_Mutation	SNP	G	T	13.13%	1.69%	11.54%
P3	CEP295	11	93463408	93463408	Silent	SNP	G	A	15.62%	5.38%	11.59%
P3	IL22	12	68647066	68647066	Missense_Mutation	SNP	G	A	5.66%	1.56%	22.50%
P3	GALNS	16	88891232	88891232	Silent	SNP	G	A	9.47%	1.56%	15.22%
P3	CACNA1G	17	48668851	48668851	Missense_Mutation	SNP	C	T	3.86%	9.58%	3.70%
P3	SOCS6	18	67992621	67992621	Missense_Mutation	SNP	C	A	33.75%	21.05%	24.32%
P3	ODAD3	19	11537005	11537005	Missense_Mutation	SNP	C	T	20.00%	3.08%	12.70%
P3	CBLC	19	45284468	45284468	Missense_Mutation	SNP	G	A	9.27%	9.16%	9.00%
P3	ZNF813	19	53993724	53993724	Missense_Mutation	SNP	C	T	15.00%	19.77%	7.14%
P3	PLA2G6	22	38536114	38536114	Silent	SNP	C	T	20.00%	22.68%	6.58%
P3	PNMA6E	X	152662421	152662421	Silent	SNP	G	A	10.91%	8.11%	13.89%
P3	MFSD6	2	191301825	191301825	Missense_Mutation	SNP	G	A	8.64%	8.06%	2.44%
P3	ITPR1	3	4752053	4752053	Silent	SNP	G	A	6.00%	8.70%	
P3	H2AC8	6	26217264	26217264	Missense_Mutation	SNP	G	C	2.84%	1.39%	
P3	ZBED9	6	28554284	28554284	Missense_Mutation	SNP	T	A	79.59%	71.05%	66.67%
P3	PSMA2	7	42961479	42961479	Missense_Mutation	SNP	C	G	10.53%	1.92%	
P3	HOOK3	8	42873586	42873586	Missense_Mutation	SNP	C	T	6.09%	6.11%	7.46%
P3	RFX3	9	3263041	3263041	Missense_Mutation	SNP	G	A	10.64%	29.55%	4.44%
P3	GRAMD1B	11	123480990	123480990	Missense_Mutation	SNP	C	G	12.50%	11.70%	4.88%
P3	FIGNL2	12	52214414	52214414	Missense_Mutation	SNP	C	T	10.14%	5.00%	21.57%
P3	FARP1	13	99092999	99092999	Missense_Mutation	SNP	G	A	19.51%	12.90%	24.27%
P3	FBXL16	16	746880	746880	Missense_Mutation	SNP	C	T	43.59%	36.36%	35.71%
P3	CORO7	16	4405321	4405321	Missense_Mutation	SNP	T	A	36.96%	47.37%	40.00%
P3	PIK3R5	17	8785136	8785136	Silent	SNP	G	A	14.04%	16.67%	3.45%
P3	EPB41L3	18	5478342	5478342	Silent	SNP	G	A	21.21%	7.69%	17.86%
P3	NFATC1	18	77246596	77246596	Missense_Mutation	SNP	C	A	5.04%	5.97%	1.64%
P3	ANGPTL4	19	8436404	8436404	Missense_Mutation	SNP	C	A	52.31%	44.74%	35.29%
P3	TSHZ3	19	31767771	31767771	Silent	SNP	G	A	26.02%	16.28%	18.97%
P3	PSG7	19	43441177	43441178	Frame_Shift_Del	DEL	G	-	6.11%	3.51%	1.39%
P3	CD177	19	43858478	43858478	Missense_Mutation	SNP	C	T	4.21%	10.34%	3.00%
P3	ZNF671	19	58238871	58238871	Missense_Mutation	SNP	G	A	9.69%	10.58%	5.21%
P3	USP11	X	47104814	47104814	Missense_Mutation	SNP	C	A	9.09%	9.86%	52.38%
P3	TRIM66	11	8662341	8662341	Silent	SNP	C	T			5.93%
P3	TSPAN18	11	44931359	44931359	Missense_Mutation	SNP	A	G			7.69%
P3	KMT2A	11	118390479	118390479	Missense_Mutation	SNP	G	A	1.06%		9.09%
P3	BRCA2	13	32911956	32911956	Missense_Mutation	SNP	C	G			9.52%
P3	CCDC168	13	103398149	103398149	Missense_Mutation	SNP	A	C			15.00%
P3	CCDC88C	14	91779547	91779547	Silent	SNP	C	T	1.31%		10.84%
P3	LRRC37A3	17	62856073	62856073	Missense_Mutation	SNP	A	C			3.72%
P3	PRX	19	40904471	40904471	Missense_Mutation	SNP	G	A			7.96%
P3	ARL13B	3	93768297	93768297	Missense_Mutation	SNP	C	T	7.06%		2.86%
P3	KCNU1	8	36698509	36698509	Missense_Mutation	SNP	C	T	8.75%		25.45%
P3	CABP1	12	121079119	121079119	Missense_Mutation	SNP	A	G	15.00%		5.00%
P3	OLFM3	1	102269808	102269808	Missense_Mutation	SNP	C	T	14.58%		
P3	GLRB	4	158057641	158057641	Silent	SNP	C	T	8.11%		
P3	CDC27	17	45219686	45219686	Missense_Mutation	SNP	A	C	4.17%		
P3	CDC27	17	45219688	45219688	Missense_Mutation	SNP	T	C	4.20%		
P3	JAG1	20	10620558	10620558	Missense_Mutation	SNP	A	C	3.19%		
P4	CCNL2	1	1322783	1322783	Missense_Mutation	SNP	C	A	27.11%	16.30%	26.74%
P4	ATAD3B	1	1417579	1417579	Missense_Mutation	SNP	G	T	28.36%	13.57%	21.01%
P4	PRXL2B	1	2519828	2519829	Frame_Shift_Del	DEL	G	-	28.95%	16.15%	25.79%
P4	MFN2	1	12067279	12067279	Missense_Mutation	SNP	G	C	25.83%	18.23%	23.33%
P4	VWA5B1	1	20656787	20656787	Missense_Mutation	SNP	C	A	30.53%	17.29%	22.22%
P4	ARID1A	1	27056345	27056346	Frame_Shift_Del	DEL	T	-	22.73%	17.54%	18.05%
P4	EYA3	1	28384527	28384527	Missense_Mutation	SNP	T	C	28.95%	13.46%	27.27%
P4	PTPRU	1	29650240	29650241	Missense_Mutation	DNP	TG	CT	21.15%	17.09%	17.10%
P4	CSMD2	1	33985462	33985462	Silent	SNP	A	C	30.70%	19.91%	20.32%
P4	RPE65	1	68910288	68910289	Frame_Shift_Del	DEL	C	-	20.99%	10.13%	15.31%
P4	MSH4	1	76363667	76363667	Missense_Mutation	SNP	C	A	25.49%	14.52%	22.73%
P4	COL24A1	1	86497584	86497584	Missense_Mutation	SNP	G	T	5.71%	16.13%	18.37%
P4	CHD1L	1	146736111	146736111	Nonsense_Mutation	SNP	G	T	25.30%	9.57%	28.04%
P4	SHE	1	154459051	154459051	Missense_Mutation	SNP	T	C	24.39%	18.75%	35.68%
P4	IFI16	1	159021618	159021618	Silent	SNP	A	T	27.71%	12.50%	19.75%
P4	ASTN1	1	176838129	176838129	Silent	SNP	G	A	5.88%	12.90%	10.00%
P4	RGSL1	1	182443654	182443654	Nonsense_Mutation	SNP	A	T	25.00%	16.98%	23.53%
P4	KCNT2	1	196300363	196300363	Missense_Mutation	SNP	G	T	42.22%	12.50%	13.21%

P4	LAD1	1	201351820	201351820	Missense_Mutation	SNP	C	A	30.95%	13.79%	30.65%
P4	IPO9	1	201823833	201823833	Missense_Mutation	SNP	G	T	20.48%	13.43%	32.47%
P4	GPR37L1	1	202097296	202097296	Missense_Mutation	SNP	G	T	29.91%	15.33%	24.63%
P4	SMYD2	1	214478567	214478567	Missense_Mutation	SNP	G	T	35.85%	8.47%	14.47%
P4	RYR2	1	237798239	237798239	Missense_Mutation	SNP	G	T	20.83%	7.45%	21.43%
P4	RYR2	1	237837441	237837441	Missense_Mutation	SNP	C	A	32.86%	13.64%	20.90%
P4	NLRP3	1	247607278	247607278	Missense_Mutation	SNP	G	A	15.56%	11.86%	20.45%
P4	OR2L8	1	248112309	248112309	Missense_Mutation	SNP	G	T	26.64%	14.76%	26.85%
P4	OR2T3	1	248637089	248637089	Nonsense_Mutation	SNP	C	A	28.12%	17.13%	14.07%
P4	PXDN	2	1652032	1652032	Missense_Mutation	SNP	T	C	22.07%	16.67%	20.40%
P4	SMYD1	2	88387505	88387505	Missense_Mutation	SNP	C	G	24.74%	21.18%	23.21%
P4	FHL2	2	106002907	106002907	Nonsense_Mutation	SNP	C	A	26.79%	13.30%	28.18%
P4	RGPD4	2	108487486	108487486	Missense_Mutation	SNP	G	T	28.74%	7.89%	22.45%
P4	MYO7B	2	128367257	128367257	Silent	SNP	G	A	28.12%	14.77%	19.03%
P4	RAPGEF4	2	173832106	173832107	Frame_Shift_Del	DEL	A	-	31.45%	18.93%	26.70%
P4	MYL1	2	211167237	211167237	Missense_Mutation	SNP	G	C	19.40%	10.91%	10.81%
P4	PRICKLE2	3	64132944	64132944	Nonsense_Mutation	SNP	C	A	36.36%	15.32%	29.17%
P4	MECOM	3	168840471	168840471	Missense_Mutation	SNP	T	A	29.09%	8.47%	18.06%
P4	LRRC15	3	194081637	194081637	Missense_Mutation	SNP	C	A	32.54%	12.86%	31.46%
P4	LETM1	4	1817424	1817424	Missense_Mutation	SNP	C	A	22.77%	13.56%	24.30%
P4	CRMP1	4	5838534	5838534	Missense_Mutation	SNP	G	T	10.43%	14.93%	9.78%
P4	WFS1	4	6302666	6302666	Silent	SNP	C	T	29.63%	15.42%	12.44%
P4	KIAA0232	4	6826248	6826248	Missense_Mutation	SNP	G	T	23.53%	15.79%	28.00%
P4	SLC2A9	4	9889266	9889266	Missense_Mutation	SNP	C	A	26.32%	8.77%	7.02%
P4	CCDC149	4	24810132	24810132	Missense_Mutation	SNP	T	G	28.93%	14.76%	22.80%
P4	KIT	4	55573300	55573300	Missense_Mutation	SNP	C	A	20.83%	11.86%	11.90%
P4	TRIML2	4	189012854	189012854	Silent	SNP	G	T	21.05%	16.02%	10.97%
P4	ICE1	5	5437222	5437222	Missense_Mutation	SNP	G	T	35.85%	15.38%	7.32%
P4	DNAH5	5	13944524	13944524	Missense_Mutation	SNP	C	A	25.00%	8.00%	13.33%
P4	CDH6	5	31313447	31313447	Missense_Mutation	SNP	G	C	29.03%	17.54%	8.77%
P4	HCN1	5	45262355	45262355	Silent	SNP	G	T	34.47%	16.49%	28.57%
P4	ARAP3	5	141041683	141041683	Silent	SNP	G	A	25.78%	22.16%	31.21%
P4	RREB1	6	7226827	7226827	Missense_Mutation	SNP	G	T	14.14%	6.25%	13.82%
P4	DSP	6	7565632	7565632	Missense_Mutation	SNP	A	T	46.08%	26.37%	27.37%
P4	TNXB	6	32015695	32015695	Silent	SNP	G	A	40.34%	34.75%	33.73%
P4	ADGRF4	6	47682617	47682617	Missense_Mutation	SNP	C	A	24.32%	8.51%	22.97%
P4	DSE	6	116757507	116757507	Nonsense_Mutation	SNP	G	T	23.81%	12.86%	16.44%
P4	SAMD3	6	130505655	130505655	Missense_Mutation	SNP	G	T	24.32%	7.25%	23.86%
P4	IGF2R	6	160479948	160479948	Missense_Mutation	SNP	G	T	18.46%	7.50%	20.75%
P4	DNAH11	7	21621583	21621583	Missense_Mutation	SNP	G	T	22.06%	12.96%	20.00%
P4	PDE1C	7	31890301	31890301	Missense_Mutation	SNP	C	A	22.73%	9.09%	15.87%
P4	ABCA13	7	48520705	48520706	Missense_Mutation	DNP	GG	AT	22.13%	9.28%	11.88%
P4	CALN1	7	71275445	71275445	Missense_Mutation	SNP	C	A	40.00%	13.56%	22.41%
P4	ARPC1A	7	98951590	98951590	Missense_Mutation	SNP	A	C	30.43%	16.79%	19.67%
P4	STRIP2	7	129096478	129096478	Nonsense_Mutation	SNP	C	T	31.93%	13.68%	27.74%
P4	DENND2A	7	140301551	140301551	Missense_Mutation	SNP	C	T	30.88%	16.76%	24.34%
P4	FAM131B	7	143054471	143054471	Missense_Mutation	SNP	C	A	12.07%	8.33%	20.00%
P4	NOBOX	7	144097312	144097312	Missense_Mutation	SNP	G	T	26.72%	19.65%	21.78%
P4	CUL1	7	148484116	148484116	Silent	SNP	A	G	18.37%	8.64%	23.00%
P4	GIMAP2	7	150389504	150389504	Silent	SNP	A	C	29.52%	18.29%	32.12%
P4	GIMAP1	7	150417515	150417515	Missense_Mutation	SNP	G	T	27.31%	18.09%	23.81%
P4	KMT2C	7	151945168	151945168	Missense_Mutation	SNP	G	T	5.00%	3.85%	3.53%
P4	XKR5	8	6669165	6669165	Missense_Mutation	SNP	T	A	13.92%	13.03%	8.11%
P4	ARFGEF1	8	68178443	68178443	Splice_Site	SNP	C	A	31.03%	12.82%	35.38%
P4	KCNB2	8	73849948	73849948	Silent	SNP	C	T	27.27%	17.27%	27.78%
P4	CNBD1	8	88218268	88218268	Missense_Mutation	SNP	T	A	32.00%	9.84%	12.86%
P4	MMP16	8	89068388	89068388	Missense_Mutation	SNP	G	C	20.69%	19.05%	11.11%
P4	RUNX1T1	8	93107267	93107267	Missense_Mutation	SNP	C	A	48.04%	27.97%	44.62%
P4	PKHD1L1	8	110477264	110477264	Missense_Mutation	SNP	G	C	42.00%	29.25%	31.21%
P4	ADCY8	8	131964138	131964138	Missense_Mutation	SNP	T	C	14.89%	5.50%	5.56%
P4	COL22A1	8	139890260	139890260	Missense_Mutation	SNP	G	C	17.97%	12.85%	15.35%
P4	FREM1	9	14841480	14841480	Missense_Mutation	SNP	C	A	33.33%	21.43%	23.33%
P4	FAM205A	9	34724219	34724219	Missense_Mutation	SNP	G	T	25.87%	18.03%	22.18%
P4	WNK2	9	96051307	96051307	Missense_Mutation	SNP	A	T	43.24%	20.28%	37.17%
P4	CYLC2	9	105767753	105767753	Silent	SNP	A	T	39.94%	25.61%	26.99%
P4	KIF5B	10	32308871	32308871	Missense_Mutation	SNP	T	A	27.91%	7.37%	10.23%
P4	WASHC2C	10	46272823	46272823	Missense_Mutation	SNP	G	T	21.05%	8.74%	9.49%
P4	GDF10	10	48428667	48428667	Nonsense_Mutation	SNP	C	A	35.59%	9.86%	24.36%
P4	LRRTM3	10	68857546	68857546	Missense_Mutation	SNP	C	G	12.77%	8.00%	10.87%
P4	BTAF1	10	93713611	93713611	Missense_Mutation	SNP	G	T	27.06%	12.90%	6.32%
P4	PKD2L1	10	102089732	102089732	Silent	SNP	C	A	30.26%	15.65%	23.50%
P4	DOCK1	10	128841405	128841405	Missense_Mutation	SNP	C	G	14.75%	11.48%	21.57%
P4	TRPM5	11	2436602	2436602	Silent	SNP	G	A	28.42%	18.69%	22.69%
P4	CCKBR	11	6292406	6292406	Missense_Mutation	SNP	T	C	31.79%	16.23%	20.21%
P4	MPPED2	11	30601851	30601851	Missense_Mutation	SNP	G	C	12.00%	25.76%	23.64%
P4	ALX4	11	44331237	44331237	Silent	SNP	G	T	34.08%	17.78%	23.57%
P4	PHF21A	11	45991405	45991405	Silent	SNP	G	C	35.29%	10.64%	18.37%
P4	MADD	11	47306055	47306055	Missense_Mutation	SNP	G	T	29.20%	16.52%	18.89%
P4	OR4C15	11	55322174	55322174	Missense_Mutation	SNP	C	A	23.73%	12.73%	23.19%

P4	OR5L1	11	55579853	55579853	Missense_Mutation	SNP	T	C	39.66%	15.79%	21.69%
P4	OR8H3	11	55890133	55890133	Silent	SNP	G	T	27.88%	18.91%	22.11%
P4	PKNOX2	11	125301075	125301075	Missense_Mutation	SNP	G	T	27.59%	10.53%	19.15%
P4	ARHGAP32	11	128843227	128843227	Silent	SNP	C	A	23.33%	8.00%	24.11%
P4	WNK1	12	987413	987413	Missense_Mutation	SNP	G	T	22.73%	5.77%	22.32%
P4	KCNA5	12	5153742	5153742	Missense_Mutation	SNP	C	A	18.28%	16.32%	18.30%
P4	CLEC9A	12	10213757	10213757	Missense_Mutation	SNP	G	T	22.54%	14.00%	21.43%
P4	BICD1	12	32369211	32369211	Nonsense_Mutation	SNP	A	T	20.00%	8.70%	15.00%
P4	ESYT1	12	56536106	56536106	Missense_Mutation	SNP	C	A		3.90%	
P4	TRHDE	12	72666859	72666859	Missense_Mutation	SNP	C	A	5.36%	3.90%	8.00%
P4	NAV3	12	78400750	78400750	Frame_Shift_Ins	INS	-	T	24.56%	15.18%	15.95%
P4	CUX2	12	111760317	111760317	Silent	SNP	C	T	25.86%	17.54%	17.94%
P4	TMEM132D	12	129559419	129559419	Silent	SNP	G	T	25.00%	8.16%	10.64%
P4	PIWIL1	12	130839146	130839146	Missense_Mutation	SNP	G	T	58.82%	24.59%	35.42%
P4	POGLUT2	13	103441466	103441466	Missense_Mutation	SNP	G	T	27.27%	12.68%	23.73%
P4	GABRA5	15	27126105	27126105	Missense_Mutation	SNP	G	T	27.66%	13.21%	16.95%
P4	RYR3	15	34072462	34072462	Missense_Mutation	SNP	C	A	37.93%	28.03%	22.87%
P4	AP4E1	15	51291395	51291395	Missense_Mutation	SNP	A	T	27.12%	14.04%	13.56%
P4	CORO2B	15	69011543	69011543	Missense_Mutation	SNP	C	A	24.42%	6.33%	17.86%
P4	MYO9A	15	72190966	72190966	Missense_Mutation	SNP	C	T	14.58%	7.69%	1.20%
P4	RASGRF1	15	79356767	79356767	Missense_Mutation	SNP	A	T	28.12%	6.58%	19.35%
P4	PGAP6	16	427683	427683	Missense_Mutation	SNP	G	A	7.98%	5.39%	5.11%
P4	MYH11	16	15814741	15814741	Silent	SNP	G	T	13.48%	3.14%	8.70%
P4	AQP8	16	25228714	25228714	Missense_Mutation	SNP	C	A	9.95%	2.53%	14.03%
P4	C16orf87	16	46843616	46843616	Missense_Mutation	SNP	T	A	29.22%	22.27%	21.93%
P4	BRD7	16	50402155	50402155	Missense_Mutation	SNP	G	C	18.50%	19.44%	22.34%
P4	LOC729159	16	60392397	60392397	Missense_Mutation	SNP	C	T	32.24%	22.77%	24.71%
P4	MC1R	16	89986016	89986016	Missense_Mutation	SNP	A	T	18.18%	11.97%	12.32%
P4	TP53	17	7577090	7577090	Missense_Mutation	SNP	C	G	17.50%	20.42%	30.26%
P4	DNAH9	17	11700914	11700914	Missense_Mutation	SNP	G	T	10.71%	18.60%	10.00%
P4	NOS2	17	26106010	26106010	Silent	SNP	C	A	29.36%	14.55%	23.46%
P4	SLFN14	17	33880056	33880056	Missense_Mutation	SNP	A	G	25.25%	9.09%	20.69%
P4	TBKBP1	17	45776062	45776062	Silent	SNP	A	T	19.90%	15.81%	7.45%
P4	SMG8	17	57290752	57290752	Silent	SNP	T	A	13.58%	5.62%	1.68%
P4	MED13	17	60038313	60038313	Missense_Mutation	SNP	C	G	40.00%	16.36%	32.43%
P4	OTOP3	17	72942994	72942994	Silent	SNP	G	T	27.08%	3.33%	22.83%
P4	FBF1	17	73922164	73922165	Frame_Shift_Del	DEL	C	-	25.86%	23.13%	21.76%
P4	TNRC6C	17	76071301	76071301	Missense_Mutation	SNP	G	A	18.46%	15.19%	7.33%
P4	PSMG2	18	12720590	12720590	Missense_Mutation	SNP	G	T	31.17%	9.41%	17.91%
P4	SEH1L	18	12984098	12984098	Missense_Mutation	SNP	A	T	24.07%	10.42%	29.31%
P4	ATP8B3	19	1791751	1791751	Missense_Mutation	SNP	T	C	22.22%	13.95%	31.37%
P4	ADGRE5	19	14507161	14507161	Silent	SNP	C	T	21.48%	11.55%	17.62%
P4	FAM187B	19	35719520	35719520	Missense_Mutation	SNP	T	C	38.46%	13.64%	26.44%
P4	PSG1	19	43382306	43382306	Silent	SNP	G	A	45.12%	27.27%	29.63%
P4	PPP5D1	19	47030297	47030297	Silent	SNP	G	A	10.71%	3.02%	
P4	VRK3	19	50500811	50500811	Missense_Mutation	SNP	C	A	31.25%	16.28%	22.58%
P4	PRKCG	19	54406370	54406370	Missense_Mutation	SNP	C	A	42.31%	9.76%	18.18%
P4	RSPO4	20	941074	941074	Silent	SNP	G	T	36.32%	18.93%	28.94%
P4	ADAM33	20	3652854	3652854	Silent	SNP	G	T	30.18%	15.87%	24.41%
P4	PLCB4	20	9360749	9360749	Missense_Mutation	SNP	G	C	23.40%	8.20%	21.98%
P4	SLC32A1	20	37356824	37356824	Missense_Mutation	SNP	C	A	32.01%	20.18%	28.75%
P4	ISX	22	35481659	35481659	Missense_Mutation	SNP	G	C	25.45%	21.12%	25.96%
P4	CT47B1	X	120009285	120009285	Missense_Mutation	SNP	G	T	53.39%	28.61%	45.25%
P4	CNGA2	X	150908066	150908066	Missense_Mutation	SNP	G	C	46.67%	17.65%	46.55%
P4	CFAP74	1	1877809	1877809	Missense_Mutation	SNP	C	T	28.57%	11.11%	27.83%
P4	SLC2A7	1	9073625	9073626	Missense_Mutation	DNP	CC	AA	29.13%	13.21%	31.01%
P4	PRAMEF2	1	12919983	12919983	Missense_Mutation	SNP	G	T	12.32%	10.61%	13.11%
P4	NKAIN1	1	31660999	31660999	Silent	SNP	C	T	16.50%	15.82%	13.89%
P4	C8A	1	57378090	57378090	Missense_Mutation	SNP	C	A	23.21%	11.17%	21.40%
P4	NBPF4	1	108771625	108771625	Missense_Mutation	SNP	T	A	15.71%	6.78%	12.63%
P4	RPRD2	1	150443487	150443487	Missense_Mutation	SNP	A	T	40.54%	19.23%	34.09%
P4	EIF2D	1	206769156	206769156	Missense_Mutation	SNP	C	G	29.41%	25.32%	11.59%
P4	ERO1B	1	236399099	236399099	Silent	SNP	C	A	20.45%	11.01%	32.00%
P4	MTR	1	237058805	237058805	Nonsense_Mutation	SNP	G	T	27.44%	12.86%	23.08%
P4	OR2T7	1	248605041	248605041	Silent	SNP	C	A	24.60%	14.00%	19.22%
P4	OTOF	2	26699108	26699109	Frame_Shift_Del	DEL	G	-	25.22%	14.71%	24.53%
P4	ABCG8	2	44102541	44102541	Missense_Mutation	SNP	G	T	26.50%	17.44%	18.60%
P4	NRXN1	2	51255164	51255164	Missense_Mutation	SNP	A	T	26.75%	10.50%	20.32%
P4	CCDC85A	2	56419815	56419815	Missense_Mutation	SNP	G	C	28.23%	12.50%	26.17%
P4	CYP26B1	2	72360389	72360389	Missense_Mutation	SNP	G	C	26.03%	16.46%	31.06%
P4	SH3RF3	2	110107370	110107370	Missense_Mutation	SNP	C	A	15.52%	15.47%	19.65%
P4	CKAP2L	2	113513666	113513666	Nonsense_Mutation	SNP	G	A	31.25%	18.18%	25.36%
P4	DPP10	2	116599793	116599793	Missense_Mutation	SNP	C	T	27.45%	9.30%	14.29%
P4	ZNF806	2	133075025	133075025	Missense_Mutation	SNP	G	T	25.56%	20.93%	26.85%
P4	XIRP2	2	168106503	168106503	Missense_Mutation	SNP	G	C	21.19%	15.33%	22.10%
P4	COL6A3	2	238253157	238253157	Missense_Mutation	SNP	C	A	27.50%	26.67%	24.59%
P4	KIF1A	2	241659255	241659255	Missense_Mutation	SNP	C	T	35.29%	19.35%	22.63%
P4	TATDN2	3	10320621	10320621	Missense_Mutation	SNP	T	A	46.88%	15.91%	28.24%
P4	OR5K2	3	98216888	98216888	Missense_Mutation	SNP	C	A	40.35%	15.15%	29.41%

P4	CD200R1	3	112647776	112647776	Missense_Mutation	SNP	C	T	44.68%	17.81%	39.76%
P4	UROC1	3	126219562	126219562	Missense_Mutation	SNP	A	C	23.97%	17.82%	27.10%
P4	CLSTN2	3	140282802	140282802	Splice_Site	SNP	G	A	41.82%	17.71%	24.79%
P4	CHST2	3	142840968	142840968	Missense_Mutation	SNP	G	A	22.62%	9.59%	18.01%
P4	CHST2	3	142840971	142840971	Missense_Mutation	SNP	G	T	22.35%	8.30%	17.78%
P4	PAK2	3	196509577	196509577	Missense_Mutation	SNP	C	G		4.49%	4.96%
P4	PCDH7	4	31144125	31144125	Missense_Mutation	SNP	C	A	19.79%	13.37%	16.94%
P4	FAM114A1	4	38930932	38930932	Missense_Mutation	SNP	C	A	33.33%	10.34%	6.45%
P4	GABRA4	4	46973205	46973205	Missense_Mutation	SNP	C	A	26.32%	12.99%	10.14%
P4	CRACD	4	57182448	57182448	Missense_Mutation	SNP	G	T	32.48%	15.14%	19.43%
P4	WDFY3	4	85660252	85660252	Missense_Mutation	SNP	A	G	19.23%	3.57%	25.81%
P4	ADAMTS16	5	5239387	5239387	Missense_Mutation	SNP	C	G	26.09%	20.00%	25.20%
P4	ADCY2	5	7414831	7414831	Missense_Mutation	SNP	C	G	16.79%	14.29%	7.84%
P4	PCDHA7	5	140216189	140216189	Missense_Mutation	SNP	T	A	33.33%	15.47%	35.23%
P4	PCDHB7	5	140554312	140554312	Missense_Mutation	SNP	G	T	9.31%	4.13%	6.89%
P4	GEMIN5	5	154278154	154278154	Missense_Mutation	SNP	T	A	40.32%	22.58%	27.56%
P4	ADAM19	5	156908899	156908900	Frame_Shift_Del	DEL	G	-	52.10%	19.20%	31.34%
P4	H4C5	6	26205170	26205171	Missense_Mutation	DNP	GG	TT	15.43%	9.77%	13.96%
P4	SUPT3H	6	44971515	44971515	Missense_Mutation	SNP	T	A	10.53%	4.65%	9.09%
P4	COL12A1	6	75799881	75799881	Silent	SNP	C	A	20.25%	12.58%	26.45%
P4	TAAR5	6	132910089	132910089	Missense_Mutation	SNP	T	A	30.85%	19.00%	24.62%
P4	SDK1	7	4247799	4247799	Silent	SNP	C	T	23.81%	14.50%	24.62%
P4	RADIL	7	4841590	4841590	Missense_Mutation	SNP	C	A	21.62%	13.89%	15.87%
P4	GRID2IP	7	6579483	6579483	Missense_Mutation	SNP	G	T	25.45%	11.51%	19.64%
P4	COL28A1	7	7550755	7550755	Silent	SNP	C	A	38.71%	11.58%	16.67%
P4	HOXA1	7	27134393	27134393	Missense_Mutation	SNP	T	G	30.00%	15.47%	22.50%
P4	COL1A2	7	94038727	94038727	Missense_Mutation	SNP	C	A	35.71%	9.52%	26.28%
P4	NUP205	7	135282850	135282850	Missense_Mutation	SNP	G	T	20.69%	11.90%	30.11%
P4	RP1L1	8	10467080	10467080	Missense_Mutation	SNP	C	T	4.17%	1.48%	7.56%
P4	LZTS1	8	20110797	20110797	Silent	SNP	C	T	24.34%	13.40%	21.88%
P4	PHYHIP	8	22079391	22079391	Nonsense_Mutation	SNP	G	T	33.67%	25.00%	27.56%
P4	RAB11FIP1	8	37732593	37732593	Missense_Mutation	SNP	C	A	31.18%	20.83%	30.10%
P4	SLCO5A1	8	70744741	70744741	Missense_Mutation	SNP	G	C	36.43%	20.83%	31.76%
P4	ZFHX4	8	77766816	77766816	Silent	SNP	C	A	31.25%	8.33%	26.69%
P4	RIMS2	8	105025831	105025831	Missense_Mutation	SNP	C	G	48.28%	20.00%	40.31%
P4	NUDCD1	8	110257578	110257578	Nonsense_Mutation	SNP	C	A	37.74%	24.21%	42.75%
P4	HAS2	8	122641029	122641029	Missense_Mutation	SNP	C	T	43.62%	20.34%	44.17%
P4	TG	8	133945804	133945804	Splice_Site	SNP	A	T	10.76%	9.24%	15.38%
P4	MROH6	8	144654862	144654862	Missense_Mutation	SNP	C	A	47.96%	27.06%	44.55%
P4	FOXD4	9	117364	117364	Silent	SNP	C	A	11.82%	5.98%	11.72%
P4	TAF1L	9	32633108	32633108	Missense_Mutation	SNP	C	A	21.85%	6.88%	14.75%
P4	TTLL11	9	124855624	124855624	Missense_Mutation	SNP	C	T	42.70%	25.47%	36.56%
P4	GPR158	10	25887307	25887307	Missense_Mutation	SNP	G	T	32.48%	10.68%	15.57%
P4	ZEB1	10	31815859	31815859	Missense_Mutation	SNP	G	T	31.45%	14.65%	31.69%
P4	RBP3	10	48390758	48390758	Nonsense_Mutation	SNP	G	T	21.77%	8.47%	22.79%
P4	GDF10	10	48429024	48429024	Missense_Mutation	SNP	C	A	39.10%	18.85%	21.64%
P4	ZNF365	10	64415143	64415143	Missense_Mutation	SNP	A	T	31.91%	21.13%	23.62%
P4	TACC2	10	123970673	123970673	Missense_Mutation	SNP	G	A	30.56%	14.09%	20.90%
P4	PHRF1	11	608843	608844	Nonsense_Mutation	DNP	GG	TT	32.52%	15.65%	35.38%
P4	OR51F1	11	4790211	4790211	Nonstop_Mutation	SNP	A	G	25.42%	25.53%	12.50%
P4	SYT9	11	7324308	7324308	Missense_Mutation	SNP	T	A	23.31%	13.57%	18.75%
P4	OTOG	11	17615155	17615155	Splice_Site	SNP	A	G	23.73%	14.56%	30.65%
P4	DCDC1	11	30921930	30921930	Silent	SNP	G	A	32.50%	12.82%	14.55%
P4	SYT13	11	45274009	45274009	Missense_Mutation	SNP	C	G	29.56%	17.31%	27.93%
P4	OR8I2	11	55861061	55861061	Missense_Mutation	SNP	C	A	27.52%	18.12%	22.14%
P4	SLC22A11	11	64329570	64329570	Missense_Mutation	SNP	G	T	11.02%	0.78%	17.21%
P4	MRGPRF	11	68773098	68773098	Missense_Mutation	SNP	C	A	27.50%	6.49%	23.95%
P4	OMP	11	76813972	76813972	Silent	SNP	G	T	33.11%	14.97%	28.21%
P4	TRIM49C	11	89768446	89768446	Missense_Mutation	SNP	A	T	33.78%	10.94%	30.22%
P4	OR10G9	11	123894018	123894018	Missense_Mutation	SNP	T	A	28.57%	11.79%	22.05%
P4	ADAMTS15	11	130343360	130343360	Nonsense_Mutation	SNP	G	T	30.28%	12.98%	24.22%
P4	KDM5A	12	416940	416940	Missense_Mutation	SNP	G	T	25.47%	13.04%	20.22%
P4	ESPL1	12	53679833	53679833	Missense_Mutation	SNP	G	T	11.96%	17.22%	21.68%
P4	LGR5	12	71978097	71978097	Silent	SNP	C	A	25.93%	16.67%	16.06%
P4	PHETA1	12	111801056	111801056	Missense_Mutation	SNP	C	T	27.33%	17.61%	26.94%
P4	OAS1	12	113357432	113357432	Missense_Mutation	SNP	G	T	55.22%	28.36%	34.74%
P4	SLITRK5	13	88329509	88329509	Silent	SNP	G	T	25.68%	15.82%	21.71%
P4	NIN	14	51224896	51224896	Missense_Mutation	SNP	C	A	33.33%	7.02%	20.66%
P4	SLC8A3	14	70633382	70633382	Missense_Mutation	SNP	C	A	29.31%	26.87%	9.09%
P4	MAP3K9	14	71199475	71199475	Missense_Mutation	SNP	G	A	23.36%	10.84%	20.38%
P4	POMT2	14	77746222	77746222	Missense_Mutation	SNP	C	A	24.82%	18.11%	25.50%
P4	SERPINA12	14	94955965	94955965	Missense_Mutation	SNP	C	A	14.81%	11.54%	4.80%
P4	GJD2	15	35045163	35045163	Missense_Mutation	SNP	C	A	7.59%	1.77%	6.47%
P4	LTK	15	41801251	41801251	Missense_Mutation	SNP	C	A	29.17%	15.75%	35.59%
P4	LOXL1	15	74238807	74238807	Missense_Mutation	SNP	C	T	21.13%	13.50%	20.73%
P4	ST8SIA2	15	93007521	93007521	Missense_Mutation	SNP	C	A	24.21%	19.73%	26.24%
P4	ADAMTS17	15	100649209	100649209	Silent	SNP	C	T	22.86%	10.97%	22.60%
P4	ITGAX	16	31368026	31368026	Missense_Mutation	SNP	C	A	30.14%	31.52%	35.42%
P4	TP53	17	7578403	7578403	Missense_Mutation	SNP	C	T	24.22%	11.41%	22.32%

P4	KRT16	17	39767230	39767230	Missense_Mutation	SNP	G	C	22.73%	10.45%	16.59%
P4	CD300LG	17	41931270	41931270	Missense_Mutation	SNP	G	C	23.15%	9.14%	17.98%
P4	EPN3	17	48616591	48616591	Missense_Mutation	SNP	G	T	26.83%	17.45%	23.12%
P4	SPATA20	17	48629372	48629372	Silent	SNP	G	T	34.73%	18.57%	27.08%
P4	SCN4A	17	62018154	62018154	Missense_Mutation	SNP	C	T	24.53%	3.36%	22.11%
P4	RGS9	17	63221296	63221296	Silent	SNP	G	T	28.12%	9.56%	26.04%
P4	CD300LB	17	72518980	72518980	Missense_Mutation	SNP	G	A	17.19%	12.07%	32.52%
P4	BAHCC1	17	79425324	79425324	Missense_Mutation	SNP	G	C	24.69%	7.97%	21.00%
P4	P4HB	17	79803493	79803493	Missense_Mutation	SNP	C	A	22.28%	12.44%	23.39%
P4	SLC14A2	18	43249342	43249342	Missense_Mutation	SNP	A	G	23.21%	16.49%	27.68%
P4	STK11	19	1219379	1219379	Frame_Shift_Ins	INS	-	G	36.96%	15.53%	33.33%
P4	CRLF1	19	18709270	18709270	Missense_Mutation	SNP	A	T	45.35%	17.39%	34.78%
P4	NPHS1	19	36330212	36330212	Missense_Mutation	SNP	C	A	48.94%	18.10%	31.31%
P4	ADAM33	20	3652900	3652900	Missense_Mutation	SNP	G	T	29.23%	16.02%	31.60%
P4	MYH7B	20	33568858	33568858	Silent	SNP	G	T	29.79%	16.33%	25.00%
P4	PTPRT	20	40710567	40710567	Silent	SNP	G	T	18.49%	15.38%	29.29%
P4	PTPRT	20	40827934	40827934	Missense_Mutation	SNP	C	A	30.08%	10.48%	26.32%
P4	FITM2	20	42939685	42939685	Missense_Mutation	SNP	C	T		0.60%	2.16%
P4	CCN5	20	43353544	43353544	Missense_Mutation	SNP	C	A	29.41%	11.38%	25.34%
P4	CEBPB	20	48808422	48808422	Silent	SNP	C	T	22.91%	10.77%	28.65%
P4	BMP7	20	55803340	55803340	Missense_Mutation	SNP	C	T	26.32%	15.98%	23.76%
P4	LKAAEAR1	20	62714966	62714966	Silent	SNP	C	A	31.71%	16.16%	35.00%
P4	TPTE	21	10951379	10951379	Silent	SNP	G	T	14.17%	6.21%	11.22%
P4	UPB1	22	24919618	24919618	Silent	SNP	G	T	42.70%	17.39%	32.26%
P4	MYO18B	22	26422936	26422936	Missense_Mutation	SNP	C	A	36.94%	16.13%	32.10%
P4	EP300	22	41545780	41545780	Missense_Mutation	SNP	T	G	31.63%	19.01%	40.34%
P4	EZHIP	X	51150439	51150439	Missense_Mutation	SNP	A	G	58.62%	21.18%	48.45%
P4	ARHGEF9	X	62926259	62926259	Missense_Mutation	SNP	T	A	67.50%	35.37%	43.90%
P4	IGSF1	X	130409468	130409468	Silent	SNP	C	A	58.97%	34.38%	43.24%
P4	BMP8B	1	40253923	40253924	Missense_Mutation	DNP	CC	AG	20.00%	5.56%	11.11%
P4	FAAH	1	46860144	46860144	Missense_Mutation	SNP	G	T	22.36%	19.55%	14.43%
P4	RPE65	1	68912434	68912434	Missense_Mutation	SNP	G	T	28.32%	16.24%	19.05%
P4	LHX8	1	75614346	75614347	Frame_Shift_Del	DEL	C	-	25.17%	10.07%	15.87%
P4	LHX8	1	75614347	75614347	Missense_Mutation	SNP	T	A	25.50%	7.91%	15.15%
P4	NES	1	156640283	156640283	Missense_Mutation	SNP	G	T	40.00%	14.44%	25.88%
P4	EDEM3	1	184677394	184677394	Missense_Mutation	SNP	G	C	38.96%	14.89%	31.25%
P4	PSEN2	1	227076626	227076626	Missense_Mutation	SNP	G	T	34.41%	17.65%	22.50%
P4	OR2T6	1	248551590	248551590	Silent	SNP	G	A	33.01%	11.76%	11.54%
P4	APOB	2	21229724	21229724	Missense_Mutation	SNP	T	C	27.78%	12.75%	13.86%
P4	TDRD15	2	21364130	21364130	Missense_Mutation	SNP	T	A	7.38%	2.54%	2.08%
P4	FOXI3	2	88748324	88748324	Missense_Mutation	SNP	T	A	3.60%	0.64%	11.49%
P4	TTC30B	2	178416877	178416877	Silent	SNP	A	G	6.21%	2.76%	4.63%
P4	COL3A1	2	189864061	189864061	Silent	SNP	C	A	26.89%	13.08%	16.13%
P4	NECTIN3	3	110852698	110852698	Missense_Mutation	SNP	G	A	41.56%	21.52%	23.16%
P4	GAK	4	870972	870972	Missense_Mutation	SNP	T	G	31.29%	14.61%	15.53%
P4	MSANTD1	4	3251252	3251252	Missense_Mutation	SNP	C	A	25.86%	10.37%	18.18%
P4	TACR3	4	104510950	104510950	Silent	SNP	T	C	4.67%	1.65%	10.40%
P4	NR3C2	4	149357803	149357803	Silent	SNP	T	C	49.35%	15.74%	15.28%
P4	MARCHF6	5	10390488	10390488	Missense_Mutation	SNP	C	T	22.22%	15.56%	19.86%
P4	CKMT2	5	80555027	80555027	Missense_Mutation	SNP	C	A	48.44%	16.39%	25.00%
P4	PCDHGA11	5	140800984	140800984	Missense_Mutation	SNP	G	C	41.11%	15.65%	19.82%
P4	TRIM41	5	180659873	180659873	Missense_Mutation	SNP	G	A	21.09%	9.40%	10.14%
P4	CARMIL1	6	25510769	25510769	Missense_Mutation	SNP	G	T	42.00%	18.60%	25.51%
P4	NCOA7	6	126249377	126249377	Nonsense_Mutation	SNP	G	T	20.59%	12.57%	20.44%
P4	LPA	6	161054931	161054931	Silent	SNP	C	T	9.68%	5.08%	1.59%
P4	RPS6KA2	6	166952194	166952194	Nonsense_Mutation	SNP	C	A	18.75%	11.48%	21.43%
P4	DDC	7	50605566	50605566	Missense_Mutation	SNP	C	A	12.73%	19.39%	22.02%
P4	GTF2I	7	74113349	74113349	Silent	SNP	A	T	27.21%	11.36%	21.77%
P4	PON2	7	95039353	95039353	Missense_Mutation	SNP	G	C	10.39%	3.33%	4.00%
P4	CPA5	7	129987689	129987689	Splice_Site	SNP	G	A	47.73%	16.95%	20.00%
P4	CA8	8	61178542	61178542	Missense_Mutation	SNP	C	A	29.73%	12.82%	30.26%
P4	CSMD3	8	113504865	113504865	Missense_Mutation	SNP	T	A	9.59%	18.82%	11.82%
P4	FAM135B	8	139164882	139164882	Silent	SNP	G	A	10.00%	2.97%	20.99%
P4	LHX2	9	126776404	126776404	Frame_Shift_Ins	INS	-	A	44.93%	17.95%	33.70%
P4	HMCN2	9	133302752	133302752	Silent	SNP	G	C	45.45%	25.00%	30.30%
P4	PLPP7	9	134183329	134183329	Missense_Mutation	SNP	G	T	42.53%	24.24%	28.89%
P4	PARD3	10	34636964	34636964	Missense_Mutation	SNP	T	C	25.21%	18.18%	18.69%
P4	PCDH15	10	55581921	55581922	Frame_Shift_Del	DEL	G	-	29.00%	12.37%	11.11%
P4	VCL	10	75874122	75874122	Missense_Mutation	SNP	C	T	24.54%	12.44%	6.32%
P4	GPR26	10	125426324	125426324	Missense_Mutation	SNP	C	A	31.85%	13.08%	22.31%
P4	OR6A2	11	6816440	6816440	Missense_Mutation	SNP	A	G	40.37%	10.34%	21.83%
P4	OR5L1	11	55579644	55579644	Missense_Mutation	SNP	G	T	28.04%	19.35%	19.44%
P4	MYRF	11	61544913	61544913	Missense_Mutation	SNP	C	A	29.63%	13.91%	26.17%
P4	CCDC88B	11	64109145	64109145	Silent	SNP	G	T	21.82%	15.13%	8.70%
P4	GRIN2B	12	13761717	13761717	Missense_Mutation	SNP	C	A	33.67%	14.52%	12.50%
P4	CNTN1	12	41302252	41302252	Silent	SNP	G	T	16.00%	4.84%	10.00%
P4	KRT78	12	53242690	53242690	Missense_Mutation	SNP	G	T	4.00%	0.66%	12.57%
P4	OTOGL	12	80615985	80615985	Missense_Mutation	SNP	C	A	5.22%	1.05%	8.40%
P4	SACS	13	23904737	23904737	Missense_Mutation	SNP	C	A	29.09%	12.61%	17.50%

P4	SEL1L	14	81952714	81952714	Missense_Mutation	SNP	C	G	36.25%	16.92%	14.71%
P4	FAM181A	14	94394865	94394865	Missense_Mutation	SNP	G	T	28.00%	10.34%	19.35%
P4	DNAAF4	15	55724695	55724695	Missense_Mutation	SNP	C	A	22.22%	14.29%	24.00%
P4	AGBL1	15	86807705	86807705	Nonsense_Mutation	SNP	G	T	28.22%	14.91%	19.48%
P4	ACAN	15	89402148	89402149	Nonsense_Mutation	DNP	TC	GA	31.87%	14.16%	23.81%
P4	TPSD1	16	1306310	1306311	Missense_Mutation	DNP	GC	AT	3.85%	2.16%	2.37%
P4	ACSM5	16	20422896	20422896	Silent	SNP	T	A	19.15%	2.83%	10.09%
P4	NDUFAB1	16	23607570	23607570	Silent	SNP	G	C	39.08%	21.05%	27.59%
P4	RAD51D	17	33428385	33428385	Splice_Site	SNP	C	A	17.26%	10.27%	11.98%
P4	BAHCC1	17	79409048	79409048	Missense_Mutation	SNP	G	A	24.43%	11.32%	22.58%
P4	SLC35G4	18	11610608	11610608	Silent	SNP	G	A	13.33%	8.00%	16.67%
P4	ROCK1	18	18571232	18571232	Missense_Mutation	SNP	C	A	18.18%	17.81%	23.75%
P4	CDH7	18	63548130	63548130	Nonstop_Mutation	SNP	G	T	18.00%	6.78%	24.00%
P4	GNA11	19	3121001	3121001	Missense_Mutation	SNP	G	A	38.10%	13.64%	30.43%
P4	ANKRD24	19	4199752	4199752	Missense_Mutation	SNP	A	G	43.75%	16.00%	21.88%
P4	HAO1	20	7875820	7875820	Nonsense_Mutation	SNP	G	T	18.18%	9.09%	25.81%
P4	CYP2D7	22	42539504	42539504	Missense_Mutation	SNP	C	T	2.54%	0.44%	0.89%
P4	THOC2	X	122766751	122766751	Silent	SNP	T	A	61.33%	25.58%	31.82%
P4	RYR2	1	237617717	237617717	Missense_Mutation	SNP	C	T	6.25%		10.34%
P4	NEB	2	152410419	152410419	Missense_Mutation	SNP	G	A			3.70%
P4	SPHKAP	2	228882071	228882071	Missense_Mutation	SNP	C	A	9.41%		24.31%
P4	P4HTM	3	49042315	49042315	Silent	SNP	G	A			4.17%
P4	VPS41	7	38791788	38791788	Nonsense_Mutation	SNP	G	T			3.90%
P4	CCT6A	7	56123474	56123474	Missense_Mutation	SNP	C	T			5.56%
P4	AKAP6	14	33069853	33069853	Missense_Mutation	SNP	G	T	4.76%		15.09%
P4	PRR5	22	45128210	45128210	Missense_Mutation	SNP	C	T			3.51%
P4	RESP18	2	220195559	220195559	Missense_Mutation	SNP	G	T	8.00%		16.28%
P4	RIMBP2	12	130926627	130926627	Missense_Mutation	SNP	C	A	7.94%		7.02%
P4	NBPF1	1	16903848	16903848	Missense_Mutation	SNP	T	A	6.45%		
P4	USP17L7	8	11990033	11990033	Missense_Mutation	SNP	C	G	4.12%		
P4	LACTB	15	63419085	63419085	Missense_Mutation	SNP	G	A	10.53%		
P5	PRKCZ	1	2082237	2082237	Silent	SNP	G	A	5.75%	14.75%	2.22%
P5	KCNAB2	1	6132840	6132840	Missense_Mutation	SNP	G	T	10.29%	11.86%	18.57%
P5	MAD2L2	1	11735715	11735715	Missense_Mutation	SNP	C	A	7.87%	5.08%	1.94%
P5	CTRC	1	15772213	15772213	Missense_Mutation	SNP	G	T	23.00%	26.01%	21.92%
P5	P3H1	1	43218214	43218214	Silent	SNP	C	G	44.00%	38.10%	33.90%
P5	IPO13	1	44415709	44415709	Silent	SNP	G	C	17.02%	10.97%	12.85%
P5	LRRC7	1	70509655	70509655	Missense_Mutation	SNP	A	T	12.77%	13.89%	15.52%
P5	ERICH3	1	75108750	75108750	Missense_Mutation	SNP	C	A	20.00%	9.09%	5.66%
P5	BCAR3	1	94048218	94048218	Missense_Mutation	SNP	C	A	24.05%	27.96%	27.38%
P5	NTNG1	1	108023278	108023278	Missense_Mutation	SNP	C	T	6.67%	5.80%	2.99%
P5	KCND3	1	112524255	112524255	Missense_Mutation	SNP	A	T	28.49%	23.71%	21.47%
P5	CTTNBP2NL	1	112998752	112998752	Missense_Mutation	SNP	G	T	32.22%	19.74%	23.03%
P5	ZNF687	1	151259976	151259976	Silent	SNP	G	T	19.90%	19.33%	18.64%
P5	KPRP	1	152732509	152732509	Missense_Mutation	SNP	C	A	29.25%	37.68%	22.68%
P5	SYT11	1	155851016	155851016	Missense_Mutation	SNP	A	G	21.69%	23.91%	16.33%
P5	CADM3	1	159166757	159166757	Missense_Mutation	SNP	C	G	3.12%	7.20%	3.64%
P5	NECTIN4	1	161049612	161049612	Silent	SNP	C	A	18.89%	22.57%	24.02%
P5	RCSD1	1	167666589	167666589	Missense_Mutation	SNP	C	A	18.80%	22.86%	22.26%
P5	CACNA1E	1	181764140	181764140	Silent	SNP	C	A	35.16%	33.33%	35.06%
P5	RO60	1	193038653	193038653	Missense_Mutation	SNP	A	T	42.42%	35.29%	31.40%
P5	CFHR2	1	196928097	196928098	Missense_Mutation	DNP	GG	TT	11.86%	14.55%	17.31%
P5	KCNH1	1	210857370	210857370	Missense_Mutation	SNP	G	T	17.29%	19.12%	15.95%
P5	KCNK2	1	215408295	215408295	Missense_Mutation	SNP	G	T	8.42%	22.37%	19.42%
P5	CAPN8	1	223814717	223814717	Missense_Mutation	SNP	C	A	4.22%	1.57%	2.78%
P5	JMJD4	1	227920692	227920692	Frame_Shift_Ins	INS	-	A	40.30%	33.07%	36.00%
P5	NID1	1	236187447	236187447	Missense_Mutation	SNP	G	T	32.97%	35.20%	28.12%
P5	RYR2	1	237494226	237494226	Missense_Mutation	SNP	C	T	29.51%	44.32%	32.46%
P5	OR2W5	1	247655039	247655039	Missense_Mutation	SNP	G	T	29.23%	32.82%	32.52%
P5	OR13G1	1	247835712	247835712	Missense_Mutation	SNP	G	T	41.18%	34.57%	36.89%
P5	OR1C1	1	247921672	247921672	Missense_Mutation	SNP	C	A	29.73%	25.45%	22.79%
P5	TRIM58	1	248039403	248039403	Missense_Mutation	SNP	G	T	26.80%	23.40%	33.12%
P5	OR2AJ1	1	248097872	248097872	Missense_Mutation	SNP	C	A	20.78%	15.38%	7.65%
P5	OR2M2	1	248344204	248344204	Missense_Mutation	SNP	G	C	23.94%	29.81%	28.19%
P5	OR2M3	1	248367197	248367197	Silent	SNP	A	G	4.00%	7.51%	4.61%
P5	OR2T3	1	248636674	248636674	Missense_Mutation	SNP	C	A	16.07%	23.81%	22.95%
P5	OR14I1	1	248845227	248845227	Missense_Mutation	SNP	G	T	34.13%	16.67%	29.06%
P5	TDRD15	2	21363322	21363322	Nonsense_Mutation	SNP	G	T	14.29%	8.33%	8.47%
P5	IFT172	2	27688344	27688344	Missense_Mutation	SNP	G	T	18.75%	22.73%	12.66%
P5	ACTG2	2	74129498	74129498	Silent	SNP	G	A	8.20%	23.26%	20.00%
P5	CD8B	2	87080136	87080136	Missense_Mutation	SNP	C	A	12.50%	12.12%	15.38%
P5	FAM178B	2	97586908	97586908	Missense_Mutation	SNP	C	T	6.00%	4.40%	8.62%
P5	GPR45	2	105858951	105858951	Silent	SNP	G	A	27.69%	25.71%	22.58%
P5	SULT1C3	2	108863813	108863813	Missense_Mutation	SNP	C	A	15.69%	6.52%	25.00%
P5	CYP27C1	2	127956972	127956973	Missense_Mutation	DNP	GC	TT	23.21%	17.00%	11.65%
P5	MYO7B	2	128321830	128321830	Missense_Mutation	SNP	G	T	9.26%	12.12%	18.92%
P5	POTEF	2	130832756	130832756	Silent	SNP	G	T	17.66%	13.86%	14.65%
P5	AMER3	2	131521877	131521877	Missense_Mutation	SNP	G	C	10.29%	7.58%	1.14%
P5	POTEE	2	132021090	132021091	Frame_Shift_Del	DEL	G	-	22.79%	14.67%	16.41%

P5	LRP1B	2	141079557	141079557	Silent	SNP	G	T	6.15%	9.43%	20.59%
P5	KYNU	2	143676245	143676245	Missense_Mutation	SNP	A	T	14.58%	26.42%	4.00%
P5	FIGN	2	164467646	164467646	Missense_Mutation	SNP	C	A	21.66%	26.73%	15.86%
P5	CALCRL	2	188225433	188225433	Missense_Mutation	SNP	A	T	5.00%	5.84%	2.48%
P5	DNER	2	230312111	230312111	Silent	SNP	A	G	26.22%	25.74%	17.41%
P5	SP140	2	231134555	231134555	Missense_Mutation	SNP	C	A	20.51%	28.74%	12.21%
P5	UGT1A9	2	234580833	234580833	Missense_Mutation	SNP	G	T	30.83%	23.96%	13.39%
P5	COL6A3	2	238287625	238287625	Silent	SNP	C	A	4.62%	5.26%	3.79%
P5	MLPH	2	238451219	238451219	Missense_Mutation	SNP	A	T	27.81%	20.39%	20.29%
P5	TRAF3IP1	2	239234476	239234476	Silent	SNP	A	G	9.88%	13.56%	5.08%
P5	WNT7A	3	13896181	13896181	Nonsense_Mutation	SNP	G	A	24.24%	20.57%	27.13%
P5	GRIP2	3	14538063	14538063	Silent	SNP	G	T	28.29%	17.43%	22.41%
P5	EFHB	3	19974768	19974768	Missense_Mutation	SNP	G	T	42.00%	20.37%	27.59%
P5	TGFBR2	3	30713327	30713327	Missense_Mutation	SNP	A	G	29.48%	15.15%	22.47%
P5	ARPP21	3	35770964	35770964	Silent	SNP	C	A	29.22%	18.10%	18.38%
P5	GPR15	3	98251683	98251683	Nonsense_Mutation	SNP	T	A	6.82%	9.09%	3.64%
P5	ILDR1	3	121720672	121720672	Missense_Mutation	SNP	T	C	34.31%	20.45%	22.66%
P5	KALRN	3	124175510	124175510	Missense_Mutation	SNP	G	T	25.00%	23.44%	17.65%
P5	EEFSEC	3	128060522	128060522	Missense_Mutation	SNP	G	T	28.74%	25.76%	21.84%
P5	KY	3	134329198	134329198	Silent	SNP	C	A	26.19%	21.21%	19.90%
P5	DZIP1L	3	137799417	137799417	Missense_Mutation	SNP	G	A	19.51%	9.09%	23.26%
P5	TRIM42	3	140407054	140407054	Silent	SNP	G	T	25.68%	25.42%	32.12%
P5	LRRC15	3	194080914	194080914	Missense_Mutation	SNP	C	A	38.39%	26.92%	23.58%
P5	PAK2	3	196509577	196509577	Missense_Mutation	SNP	C	G	8.57%	3.39%	
P5	OTOP1	4	4199709	4199709	Missense_Mutation	SNP	C	A	8.25%	13.64%	8.60%
P5	TADA2B	4	7045526	7045526	Nonsense_Mutation	SNP	G	T	21.62%	25.68%	12.28%
P5	SH3TC1	4	8226934	8226934	Nonsense_Mutation	SNP	C	T	9.89%	22.50%	9.17%
P5	GPR78	4	8588859	8588860	Frame_Shift_Del	DEL	G	-	36.92%	33.03%	34.38%
P5	TLR10	4	38774908	38774908	Missense_Mutation	SNP	C	A	6.67%	15.71%	6.19%
P5	GABRB1	4	47163461	47163461	Missense_Mutation	SNP	G	A	14.06%	8.33%	5.80%
P5	LRRC66	4	52862277	52862277	Missense_Mutation	SNP	C	A	23.24%	15.48%	29.94%
P5	CRACD	4	57182569	57182569	Silent	SNP	C	A	39.02%	29.94%	33.12%
P5	SPINK2	4	57677872	57677872	Missense_Mutation	SNP	C	G	4.61%		
P5	RAB33B	4	140375475	140375475	Silent	SNP	C	T	36.80%	36.84%	26.50%
P5	SLC6A18	5	1225618	1225618	Missense_Mutation	SNP	C	T	25.43%	19.55%	11.54%
P5	TERT	5	1294167	1294167	Silent	SNP	G	T	35.29%	39.55%	38.06%
P5	CTNND2	5	10973806	10973807	Missense_Mutation	DNP	GG	TT	27.69%	31.43%	29.91%
P5	FBXL7	5	15928263	15928263	Missense_Mutation	SNP	C	A	36.49%	35.03%	30.57%
P5	PLK2	5	57753078	57753078	Missense_Mutation	SNP	C	A	46.88%	36.62%	27.18%
P5	CCDC125	5	68616187	68616187	Missense_Mutation	SNP	G	C	8.65%		8.82%
P5	PCSK1	5	95743981	95743981	Missense_Mutation	SNP	G	T	43.90%	31.91%	26.13%
P5	GFRA3	5	137589537	137589537	Missense_Mutation	SNP	T	A	11.32%	14.81%	5.36%
P5	PCDHB12	5	140588575	140588575	Missense_Mutation	SNP	C	A	5.71%		
P5	PCDHGA6	5	140755357	140755357	Silent	SNP	A	G	2.65%		0.41%
P5	CDHR2	5	176011658	176011660	Frame_Shift_Del	DEL	AG	-	44.14%	22.22%	26.96%
P5	HK3	5	176318488	176318488	Missense_Mutation	SNP	G	T	31.30%	18.87%	36.54%
P5	SIRT5	6	13595795	13595795	Missense_Mutation	SNP	G	A	12.07%		2.38%
P5	H2AC16	6	27833484	27833484	Missense_Mutation	SNP	C	G	26.86%	24.46%	21.34%
P5	MPIG6B	6	31691561	31691561	Silent	SNP	C	T	21.19%	14.71%	13.53%
P5	COL11A2	6	33137268	33137268	Splice_Site	SNP	C	A	27.74%	20.93%	17.98%
P5	GRM4	6	34100921	34100921	Missense_Mutation	SNP	T	A	19.87%	17.01%	14.29%
P5	ABCC10	6	43414130	43414130	Missense_Mutation	SNP	A	G	23.20%	18.85%	19.22%
P5	DST	6	56492946	56492946	Missense_Mutation	SNP	C	T	13.04%	29.27%	21.57%
P5	PRIM2	6	57512591	57512591	Silent	SNP	C	A	16.33%	28.00%	18.00%
P5	RIMS1	6	72945355	72945356	Frame_Shift_Del	DEL	A	-	12.50%	27.66%	14.81%
P5	MMS22L	6	97616011	97616011	Missense_Mutation	SNP	T	A	11.69%	12.50%	20.78%
P5	GRIK2	6	102074490	102074491	Missense_Mutation	DNP	GG	TT	18.75%	17.50%	20.63%
P5	GPR6	6	110301083	110301083	Silent	SNP	G	A	16.36%	28.17%	13.24%
P5	LAMA2	6	129802568	129802568	Missense_Mutation	SNP	G	T	10.29%	11.86%	20.78%
P5	ERMARD	6	170176730	170176730	Missense_Mutation	SNP	G	T	16.67%	24.03%	18.48%
P5	MAD1L1	7	1937856	1937856	Missense_Mutation	SNP	C	A	6.50%	1.14%	2.42%
P5	FOXK1	7	4801882	4801882	Silent	SNP	G	T	23.53%	27.18%	20.81%
P5	FERD3L	7	19184612	19184612	Missense_Mutation	SNP	C	A	28.72%	23.01%	26.29%
P5	ABCB5	7	20768079	20768079	Splice_Site	SNP	G	A	16.98%	40.43%	10.42%
P5	SP4	7	21469353	21469353	Silent	SNP	G	A	29.81%	31.71%	32.14%
P5	IGF2BP3	7	23391108	23391108	Nonsense_Mutation	SNP	G	A	36.05%	20.45%	19.91%
P5	BMPER	7	34118728	34118728	Silent	SNP	G	T	33.33%	25.64%	29.52%
P5	HECW1	7	43484941	43484941	Missense_Mutation	SNP	A	T	24.85%	27.54%	24.28%
P5	LIMK1	7	73535279	73535279	Missense_Mutation	SNP	C	T	32.63%	31.95%	20.21%
P5	ZP3	7	76062956	76062956	Missense_Mutation	SNP	C	A	44.44%	30.00%	22.56%
P5	PCLO	7	82582039	82582039	Missense_Mutation	SNP	A	G	6.56%	7.46%	10.53%
P5	PCLO	7	82584334	82584334	Nonsense_Mutation	SNP	C	A	8.65%	4.71%	8.99%
P5	SAMD9	7	92733705	92733706	Missense_Mutation	DNP	CC	AA	35.87%	33.33%	36.23%
P5	ALKBH4	7	102100098	102100098	Missense_Mutation	SNP	T	C	33.33%	44.09%	33.97%
P5	PIK3CG	7	106509963	106509963	Missense_Mutation	SNP	G	T	10.42%	20.00%	7.14%
P5	IFRD1	7	112115551	112115551	Missense_Mutation	SNP	G	T	25.42%	37.96%	26.32%
P5	PTPRZ1	7	121651600	121651600	Missense_Mutation	SNP	C	A	16.22%	4.44%	15.79%
P5	RBM28	7	127977211	127977211	Missense_Mutation	SNP	T	A	8.33%		
P5	FLNC	7	128496705	128496705	Splice_Site	SNP	G	A	35.23%	37.50%	22.22%

P5	EXOC4	7	133749170	133749170	Silent	SNP	G	C	4.96%		
P5	OR2F2	7	143633035	143633035	Missense_Mutation	SNP	C	G	30.10%	29.11%	20.41%
P5	ZNF282	7	148907766	148907766	Nonsense_Mutation	SNP	G	T	10.77%	5.71%	9.89%
P5	ZNF862	7	149557727	149557727	Missense_Mutation	SNP	G	C	6.41%	11.11%	15.38%
P5	GIMAP1	7	150417935	150417935	Silent	SNP	G	A	45.69%	32.95%	22.43%
P5	SLC4A2	7	150763808	150763808	Missense_Mutation	SNP	G	T	35.19%	42.11%	25.35%
P5	HGSNAT	8	43052163	43052163	Missense_Mutation	SNP	A	T	4.17%	4.10%	5.31%
P5	PRKDC	8	48777209	48777209	Frame_Shift_Ins	INS	-	AA	34.38%	36.15%	33.52%
P5	PRKDC	8	48777212	48777213	Frame_Shift_Del	DEL	T	-	35.87%	37.01%	33.89%
P5	SNTG1	8	51314824	51314824	Silent	SNP	C	A	4.55%	1.91%	1.53%
P5	PXDNL	8	52321104	52321104	Missense_Mutation	SNP	G	C	40.46%	38.73%	40.10%
P5	ST18	8	53062538	53062538	Splice_Site	SNP	C	A	51.90%	44.44%	31.43%
P5	OPRK1	8	54142354	54142354	Missense_Mutation	SNP	C	T	30.15%	31.25%	15.04%
P5	RP1	8	55533931	55533931	Silent	SNP	G	T	17.62%	15.87%	13.68%
P5	XKR4	8	56436546	56436546	Silent	SNP	T	G	2.15%	0.70%	0.43%
P5	CHCHD7	8	57127209	57127209	Missense_Mutation	SNP	A	G	3.01%	7.64%	5.65%
P5	CYP7A1	8	59404080	59404080	Missense_Mutation	SNP	G	T	5.41%	0.94%	4.08%
P5	PREX2	8	69104655	69104655	Missense_Mutation	SNP	A	T	15.79%	20.31%	22.73%
P5	SBSPON	8	74005238	74005238	Missense_Mutation	SNP	C	A	50.52%	41.22%	43.88%
P5	STAU2	8	74516031	74516031	Missense_Mutation	SNP	T	C	4.84%	3.55%	5.22%
P5	CRISPLD1	8	75929134	75929134	Missense_Mutation	SNP	A	T	35.11%	34.72%	44.21%
P5	ZFHX4	8	77766442	77766442	Silent	SNP	C	T	16.89%	13.30%	13.08%
P5	ZFHX4	8	77775815	77775816	Missense_Mutation	DNP	CC	AA	49.66%	39.62%	34.06%
P5	VIRMA	8	95556073	95556073	Missense_Mutation	SNP	G	A	22.44%	30.30%	25.44%
P5	VPS13B	8	100883033	100883033	Missense_Mutation	SNP	C	G	60.00%	51.92%	42.71%
P5	RIMS2	8	104922381	104922381	Missense_Mutation	SNP	G	C	6.94%	22.62%	23.64%
P5	ZFPM2	8	106814533	106814533	Missense_Mutation	SNP	G	A	33.33%	28.36%	34.81%
P5	TRPS1	8	116616340	116616340	Missense_Mutation	SNP	G	C	43.64%	38.54%	46.34%
P5	SLC30A8	8	118169985	118169985	Silent	SNP	A	T	15.68%	21.21%	14.97%
P5	CCN4	8	134225321	134225321	Missense_Mutation	SNP	C	T	2.67%	0.61%	0.50%
P5	SCRIB	8	144889127	144889127	Silent	SNP	G	T	25.00%	7.62%	10.77%
P5	IZUMO3	9	24544237	24544237	Missense_Mutation	SNP	C	A	42.86%	33.33%	18.42%
P5	ANKRD18B	9	33524679	33524679	Silent	SNP	C	T	31.40%	38.46%	33.57%
P5	CNTNAP3	9	39078872	39078872	Missense_Mutation	SNP	C	A	20.35%	15.20%	8.89%
P5	WNK2	9	96070761	96070762	Frame_Shift_Del	DEL	G	-	45.59%	24.53%	14.67%
P5	TRIM32	9	119461189	119461189	Missense_Mutation	SNP	C	T	35.77%	32.79%	27.69%
P5	ENG	9	130586606	130586606	Missense_Mutation	SNP	C	G	12.31%		2.90%
P5	HMCN2	9	133269962	133269963	Splice_Site	DNP	GG	TT	11.11%	14.71%	18.18%
P5	CACNA1B	9	141010078	141010078	Silent	SNP	C	A	23.87%	23.61%	18.92%
P5	CUBN	10	16946029	16946029	Missense_Mutation	SNP	C	A	12.77%	16.07%	20.00%
P5	RET	10	43615187	43615187	Missense_Mutation	SNP	G	T	30.46%	32.21%	21.21%
P5	LRRC18	10	50121966	50121967	Missense_Mutation	DNP	CC	AG	20.61%	20.62%	19.86%
P5	TYSND1	10	71902454	71902454	Missense_Mutation	SNP	G	A	22.99%	17.27%	21.13%
P5	KCNMA1	10	78872137	78872137	Silent	SNP	C	A	14.00%	7.69%	13.04%
P5	NUTM2B	10	81471531	81471531	Missense_Mutation	SNP	T	G	2.75%	4.27%	4.10%
P5	TUBGCP2	10	135095811	135095811	Silent	SNP	C	A	20.92%	13.07%	21.47%
P5	OR51E2	11	4703621	4703621	Silent	SNP	T	A	32.58%	16.48%	33.53%
P5	ZNF408	11	46726911	46726911	Missense_Mutation	SNP	C	A	3.26%	0.60%	
P5	LRP4	11	46905423	46905423	Missense_Mutation	SNP	C	A	17.14%	17.95%	15.38%
P5	OR4S2	11	55418402	55418402	Missense_Mutation	SNP	C	T	16.28%	14.29%	28.57%
P5	OR9Q2	11	57958364	57958364	Silent	SNP	C	A	28.24%	20.39%	23.57%
P5	GANAB	11	62393814	62393814	Missense_Mutation	SNP	C	A	22.73%	21.10%	17.86%
P5	ZFTA	11	63532687	63532687	Missense_Mutation	SNP	C	G	6.41%	12.50%	6.80%
P5	PCNX3	11	65392717	65392717	Silent	SNP	C	T	3.85%	7.04%	8.30%
P5	KLC2	11	66033637	66033637	Missense_Mutation	SNP	T	A	26.38%	21.94%	25.68%
P5	MRGPRF	11	68773391	68773391	Silent	SNP	G	T	29.58%	26.17%	20.95%
P5	ARAP1	11	72425219	72425219	Silent	SNP	C	A	6.25%	5.38%	3.07%
P5	SLCO2B1	11	74907700	74907700	Silent	SNP	C	G	2.36%	1.80%	
P5	THAP12	11	76062366	76062366	Missense_Mutation	SNP	A	C	5.71%	1.06%	
P5	MYO7A	11	76893475	76893475	Missense_Mutation	SNP	C	A	23.08%	19.57%	18.06%
P5	GRM5	11	88337980	88337980	Nonsense_Mutation	SNP	C	A	59.40%	46.77%	43.59%
P5	IQSEC3	12	176052	176052	Missense_Mutation	SNP	G	A	5.88%	5.16%	8.38%
P5	GRIN2B	12	14019129	14019129	Missense_Mutation	SNP	G	C	3.98%	0.92%	1.65%
P5	ATF7IP	12	14589093	14589093	Missense_Mutation	SNP	T	A	46.34%	51.67%	40.38%
P5	PTPRO	12	15669846	15669846	Missense_Mutation	SNP	A	C	10.94%	5.26%	9.62%
P5	TMTC1	12	29659837	29659837	Missense_Mutation	SNP	G	T	42.31%	39.29%	36.67%
P5	KCNH3	12	49937232	49937232	Silent	SNP	C	A	17.13%	20.42%	17.21%
P5	SLC4A8	12	51882624	51882624	Silent	SNP	A	C	16.98%	4.08%	7.46%
P5	HOXC13	12	54333017	54333017	Silent	SNP	C	A	16.67%	26.32%	22.22%
P5	TMPO	12	98940155	98940155	Nonsense_Mutation	SNP	G	T	15.00%	21.57%	12.50%
P5	OAS1	12	113346449	113346449	Nonsense_Mutation	SNP	G	T	23.44%	33.33%	28.78%
P5	DDX54	12	113601069	113601069	Missense_Mutation	SNP	G	A	3.45%	1.30%	3.07%
P5	ANAPC5	12	121783706	121783706	Missense_Mutation	SNP	C	T	36.61%	29.47%	30.17%
P5	HCAR2	12	123187392	123187392	Missense_Mutation	SNP	T	G	14.78%	16.40%	9.41%
P5	HIP1R	12	123343459	123343459	Missense_Mutation	SNP	A	T	24.40%	18.54%	19.35%
P5	SLC15A4	12	129278771	129278771	Silent	SNP	G	C	10.81%	34.09%	23.68%
P5	ULK1	12	132403104	132403104	Missense_Mutation	SNP	G	T	21.35%	25.56%	19.86%
P5	GOLGA3	12	133360836	133360836	Silent	SNP	G	A	29.25%	33.63%	24.24%
P5	TUBA3C	13	19752441	19752441	Missense_Mutation	SNP	T	C	38.81%	30.95%	28.80%

P5	THSD1	13	52960273	52960273	Missense_Mutation	SNP	C	A	9.21%	7.27%	4.62%
P5	KLHL1	13	70314688	70314688	Missense_Mutation	SNP	C	G	26.92%	19.23%	16.22%
P5	CARS2	13	111340074	111340074	Missense_Mutation	SNP	C	A	5.00%	0.83%	7.86%
P5	OR4K13	14	20502336	20502336	Silent	SNP	G	T	22.86%	14.29%	22.58%
P5	MDGA2	14	47342678	47342678	Missense_Mutation	SNP	A	G	60.69%	49.56%	46.67%
P5	JDP2	14	75904726	75904726	Missense_Mutation	SNP	C	A	6.19%	1.09%	
P5	NDN	15	23931645	23931645	Silent	SNP	G	C	30.56%	20.00%	21.11%
P5	APBA2	15	29406117	29406117	Silent	SNP	G	A	22.54%	20.54%	14.69%
P5	SCG5	15	32936002	32936002	Missense_Mutation	SNP	G	A	16.67%	5.88%	9.68%
P5	ACTC1	15	35084445	35084445	Silent	SNP	C	G	14.04%	17.19%	10.29%
P5	PRR35	16	614716	614716	Silent	SNP	G	T	34.09%	19.13%	16.67%
P5	IFT140	16	1570293	1570293	Missense_Mutation	SNP	C	A	26.06%	19.50%	18.93%
P5	TBC1D24	16	2550328	2550328	Missense_Mutation	SNP	G	T	21.71%	31.82%	25.71%
P5	ITGAX	16	31371667	31371667	Missense_Mutation	SNP	G	T	30.61%	22.78%	16.39%
P5	LONP2	16	48368147	48368147	Nonsense_Mutation	SNP	G	T	11.11%	25.61%	18.42%
P5	MMP2	16	55539343	55539343	Missense_Mutation	SNP	C	G	13.04%	35.29%	8.33%
P5	COG8	16	69368722	69368722	Missense_Mutation	SNP	T	A	28.86%	33.85%	23.81%
P5	COG8	16	69368742	69368742	Silent	SNP	A	C	28.87%	31.67%	25.40%
P5	HPR	16	72110547	72110547	Missense_Mutation	SNP	T	A	25.56%	22.45%	17.73%
P5	SPATA22	17	3372615	3372615	Missense_Mutation	SNP	T	G	11.11%	8.22%	17.65%
P5	SPATA22	17	3372617	3372617	Translation_Start_Site	SNP	C	A	11.29%	8.22%	18.00%
P5	TP53	17	7578257	7578257	Nonsense_Mutation	SNP	C	A	49.41%	38.55%	43.43%
P5	KDM6B	17	7752859	7752859	Missense_Mutation	SNP	A	T	19.43%	35.48%	24.43%
P5	ARHGEF15	17	8218273	8218273	Silent	SNP	C	T	33.33%	24.69%	26.76%
P5	MYH4	17	10358321	10358321	Missense_Mutation	SNP	C	A	26.39%	28.57%	15.56%
P5	TRPV2	17	16327042	16327042	Silent	SNP	C	A	23.56%	11.02%	25.30%
P5	TRIM16L	17	18638419	18638419	Missense_Mutation	SNP	A	T	25.50%	40.51%	23.34%
P5	TVP23B	17	18694294	18694294	Missense_Mutation	SNP	T	A	19.82%	20.31%	16.13%
P5	KCNJ12	17	21319266	21319266	Silent	SNP	G	T	10.13%	8.11%	9.21%
P5	SLC13A2	17	26818552	26818552	Nonsense_Mutation	SNP	C	A	26.95%	21.13%	23.39%
P5	UNC45B	17	33495327	33495327	Nonsense_Mutation	SNP	A	T	26.22%	32.35%	22.35%
P5	KRTAP3-3	17	39150132	39150132	Missense_Mutation	SNP	G	T	22.66%	33.45%	19.86%
P5	KRT33B	17	39522856	39522856	Missense_Mutation	SNP	G	A	2.21%		
P5	BRCA1	17	41246780	41246780	Missense_Mutation	SNP	C	A	29.63%	22.45%	23.20%
P5	MEIOC	17	42734553	42734553	Missense_Mutation	SNP	G	T	18.40%	28.46%	12.97%
P5	HOXB9	17	46703361	46703361	Nonsense_Mutation	SNP	C	A	34.44%	37.14%	20.91%
P5	KIF2B	17	51901016	51901017	Frame_Shift_Del	DEL	C	-	29.68%	20.30%	19.88%
P5	KCNH6	17	61611490	61611490	Missense_Mutation	SNP	G	T	27.34%	13.01%	24.79%
P5	HELZ	17	65074528	65074529	Frame_Shift_Del	DEL	C	-	23.08%	15.89%	22.51%
P5	CD300LB	17	72521920	72521920	Missense_Mutation	SNP	G	C	13.79%	7.35%	17.81%
P5	TMEM94	17	73488750	73488750	Missense_Mutation	SNP	G	T	29.55%	36.84%	20.71%
P5	ACTG1	17	79478986	79478986	Silent	SNP	T	A	26.46%	40.44%	26.48%
P5	SMCHD1	18	2740722	2740722	Missense_Mutation	SNP	A	G	16.36%	6.98%	17.39%
P5	DSC1	18	28728581	28728581	Missense_Mutation	SNP	C	T	17.65%	23.53%	15.09%
P5	SETBP1	18	42533061	42533061	Missense_Mutation	SNP	C	A	16.57%	28.57%	14.63%
P5	LOXHD1	18	44057512	44057512	Missense_Mutation	SNP	T	C	23.23%	28.95%	34.81%
P5	ST8SIA5	18	44260359	44260359	Missense_Mutation	SNP	G	C	23.70%	15.18%	11.22%
P5	ELOA2	18	44559467	44559467	Silent	SNP	G	A	40.00%	29.82%	29.53%
P5	MRO	18	48326415	48326415	Nonsense_Mutation	SNP	G	T	10.81%	15.38%	15.38%
P5	CDH20	18	59221582	59221582	Missense_Mutation	SNP	G	T	27.43%	21.05%	20.86%
P5	CDH7	18	63527052	63527052	Missense_Mutation	SNP	G	T	14.58%	18.64%	11.63%
P5	NETO1	18	70532066	70532066	Missense_Mutation	SNP	C	G	6.48%	0.97%	4.32%
P5	ZNF236	18	74607150	74607150	Silent	SNP	G	C	27.14%	24.68%	26.63%
P5	NFATC1	18	77170696	77170696	Missense_Mutation	SNP	G	T	22.22%	15.34%	16.30%
P5	PRSS57	19	694887	694887	Missense_Mutation	SNP	C	A	34.00%	32.95%	40.00%
P5	SPPL2B	19	2337583	2337583	Missense_Mutation	SNP	A	C	31.31%	27.52%	28.57%
P5	LMNB2	19	2438277	2438277	Missense_Mutation	SNP	C	A	39.27%	39.66%	24.60%
P5	CATSPERD	19	5776275	5776275	Missense_Mutation	SNP	G	T	31.43%	36.59%	23.93%
P5	DUS3L	19	5788182	5788183	Missense_Mutation	DNP	CC	AT	36.76%	35.54%	27.45%
P5	CD209	19	7810963	7810963	Silent	SNP	G	A	32.67%	21.28%	39.37%
P5	MUC16	19	9066155	9066155	Silent	SNP	A	G	34.09%	34.74%	22.62%
P5	COL5A3	19	10097038	10097039	Frame_Shift_Del	DEL	C	-	31.53%	35.21%	35.71%
P5	GCDH	19	13004463	13004463	Missense_Mutation	SNP	G	C	39.02%	19.35%	31.18%
P5	TECR	19	14675931	14675931	Silent	SNP	C	T	34.27%	25.83%	34.48%
P5	ZNF536	19	31038955	31038955	Missense_Mutation	SNP	G	T	23.13%	29.82%	18.80%
P5	FCGBP	19	40408670	40408670	Missense_Mutation	SNP	C	A	17.32%	8.40%	8.11%
P5	FCGBP	19	40411712	40411712	Missense_Mutation	SNP	T	A	22.96%	18.10%	24.37%
P5	SPTBN4	19	41008162	41008163	Frame_Shift_Del	DEL	G	-	26.67%	18.26%	18.57%
P5	HNRNPUL1	19	41808824	41808824	Missense_Mutation	SNP	G	T	31.01%	22.70%	25.41%
P5	ZNF112	19	44844609	44844609	Missense_Mutation	SNP	C	A	18.27%	19.54%	23.81%
P5	CEACAM20	19	45017293	45017293	Silent	SNP	G	T	20.00%	10.34%	20.93%
P5	KPTN	19	47980189	47980189	Silent	SNP	C	A	24.44%	21.35%	31.62%
P5	ZNF541	19	48052635	48052635	Missense_Mutation	SNP	G	T	4.05%	9.38%	
P5	BICRA	19	48184057	48184057	Missense_Mutation	SNP	G	T	31.68%	29.41%	21.62%
P5	FGF21	19	49260233	49260233	Missense_Mutation	SNP	G	T	21.32%	25.64%	27.86%
P5	PTOV1	19	50363420	50363420	Missense_Mutation	SNP	C	A	30.00%	28.83%	22.56%
P5	ATF5	19	50436232	50436232	Missense_Mutation	SNP	G	C	7.61%	3.95%	
P5	SHANK1	19	51219672	51219672	Missense_Mutation	SNP	G	T	31.96%	16.36%	24.37%
P5	KLK4	19	51411943	51411943	Missense_Mutation	SNP	C	T	28.81%	23.08%	17.24%

P5	SIGLECL1	19	51769093	51769093	Nonsense_Mutation	SNP	G	T	24.84%	7.69%	24.65%
P5	ZNF677	19	53747058	53747058	Silent	SNP	C	A	10.71%	12.31%	12.50%
P5	NLRP12	19	54304542	54304542	Missense_Mutation	SNP	G	T	15.35%	24.06%	14.61%
P5	TMC4	19	54669244	54669244	Missense_Mutation	SNP	T	C	38.29%	29.58%	28.03%
P5	LILRB2	19	54782852	54782852	Missense_Mutation	SNP	T	A	25.93%	25.26%	20.35%
P5	NLRP2	19	55494417	55494417	Missense_Mutation	SNP	A	T	27.36%	20.44%	20.47%
P5	ZNF581	19	56156253	56156253	Missense_Mutation	SNP	A	G	7.14%	2.58%	3.85%
P5	NLRP11	19	56307596	56307596	Missense_Mutation	SNP	C	T	13.16%	21.43%	36.36%
P5	SLC24A3	20	19664964	19664964	Missense_Mutation	SNP	G	T	33.33%	29.47%	28.85%
P5	CFAP61	20	20079354	20079354	Missense_Mutation	SNP	T	C	30.17%	24.83%	22.62%
P5	DEFB118	20	29960758	29960758	Missense_Mutation	SNP	G	C	5.93%	6.60%	3.73%
P5	PHF20	20	34526967	34526967	Silent	SNP	G	T	28.66%	19.64%	13.71%
P5	ARHGAP40	20	37272407	37272407	Missense_Mutation	SNP	A	T	28.12%	28.24%	21.55%
P5	PABPC1L	20	43559166	43559166	Silent	SNP	G	T	29.04%	20.18%	23.30%
P5	RIPOR3	20	49224991	49224991	Silent	SNP	C	G	26.46%	28.24%	29.41%
P5	CASS4	20	55033441	55033441	Nonsense_Mutation	SNP	G	T	23.74%	24.09%	19.12%
P5	BMP7	20	55803452	55803452	Missense_Mutation	SNP	G	T	33.70%	18.18%	21.88%
P5	EDN3	20	57876690	57876690	Missense_Mutation	SNP	G	C	25.58%	21.71%	22.27%
P5	DIDO1	20	61528082	61528082	Missense_Mutation	SNP	C	T	31.37%	26.15%	28.11%
P5	ZBTB46	20	62421693	62421693	Nonsense_Mutation	SNP	C	A	19.42%	22.28%	15.57%
P5	MYT1	20	62838994	62838994	Missense_Mutation	SNP	G	A	33.33%	30.65%	29.79%
P5	KRTAP19-8	21	32410707	32410707	Missense_Mutation	SNP	C	A	33.63%	32.81%	28.70%
P5	HLCS	21	38137470	38137470	Missense_Mutation	SNP	C	A	21.43%	30.00%	23.81%
P5	CBS	21	44483161	44483161	Missense_Mutation	SNP	T	C	28.78%	27.27%	35.83%
P5	EFCAB6	22	44073961	44073961	Missense_Mutation	SNP	T	C	6.00%	4.29%	1.20%
P5	TTLL8	22	50480094	50480094	Missense_Mutation	SNP	C	A	21.74%	14.17%	15.97%
P5	MXRA5	X	3235483	3235483	Missense_Mutation	SNP	C	G	48.84%	49.32%	50.96%
P5	GSPT2	X	51487332	51487332	Missense_Mutation	SNP	G	A	58.33%	42.37%	37.10%
P5	EDA2R	X	65819576	65819576	Missense_Mutation	SNP	T	A	51.76%	54.35%	40.24%
P5	TEX13D	X	123467112	123467112	Silent	SNP	C	A	54.43%	50.54%	43.75%
P5	SASH3	X	128922497	128922497	Silent	SNP	C	A	57.14%	43.08%	32.91%
P5	CXorf66	X	139038647	139038647	Missense_Mutation	SNP	G	A	53.26%	51.00%	49.35%
P5	FMR1NB	X	147063101	147063101	Missense_Mutation	SNP	C	A	42.24%	41.67%	40.00%
P5	F8	X	154157840	154157840	Missense_Mutation	SNP	T	A	64.71%	39.53%	61.36%
P5	BRCC3	X	154317584	154317584	Silent	SNP	A	C	61.90%	43.00%	45.87%
P5	CROCC	1	17265501	17265502	Frame_Shift_Del	DEL	G	-	11.32%	14.08%	11.25%
P5	TRIM63	1	26383758	26383758	Missense_Mutation	SNP	C	A	25.86%	32.35%	14.52%
P5	KCNA10	1	111061039	111061039	Missense_Mutation	SNP	G	T	12.50%	16.67%	14.29%
P5	TDRKH	1	151747609	151747609	Nonsense_Mutation	SNP	C	A	16.67%	12.90%	13.70%
P5	ATP8B2	1	154310014	154310014	Missense_Mutation	SNP	A	G	25.00%	15.79%	15.22%
P5	MNDA	1	158817637	158817637	Silent	SNP	C	A	18.75%	24.62%	12.84%
P5	ATP1A4	1	160125028	160125028	Missense_Mutation	SNP	C	A	25.32%	9.09%	21.43%
P5	LGR6	1	202288168	202288168	Nonsense_Mutation	SNP	G	T	20.54%	9.68%	10.81%
P5	FCMR	1	207087179	207087179	Missense_Mutation	SNP	C	T	21.13%	19.05%	17.65%
P5	FLVCR1	1	213046047	213046047	Missense_Mutation	SNP	C	T	19.05%	18.75%	16.36%
P5	GPATCH2	1	217604709	217604709	Splice_Site	SNP	T	A	21.43%	10.53%	12.87%
P5	EPRS1	1	220213588	220213588	Missense_Mutation	SNP	C	A	16.36%	13.41%	11.46%
P5	TPO	2	1499970	1499970	Splice_Site	SNP	G	T	15.13%	13.33%	22.50%
P5	TAF1B	2	10050920	10050920	Silent	SNP	G	T	18.67%	7.69%	8.45%
P5	PCARE	2	29295272	29295272	Missense_Mutation	SNP	A	T	22.33%	10.64%	26.92%
P5	SPRED2	2	65571999	65571999	Missense_Mutation	SNP	C	G	10.81%	6.98%	7.96%
P5	REG1B	2	79314673	79314673	Splice_Site	SNP	A	T	20.31%	16.95%	22.22%
P5	GPAT2	2	96691992	96691992	Missense_Mutation	SNP	C	T	20.69%	22.58%	13.73%
P5	FER1L5	2	97356314	97356314	Missense_Mutation	SNP	G	T	22.06%	13.64%	29.51%
P5	FIGN	2	164467368	164467368	Missense_Mutation	SNP	C	A	17.95%	19.57%	13.56%
P5	XIRP2	2	168102256	168102256	Missense_Mutation	SNP	T	A	16.92%	11.48%	22.97%
P5	EVX2	2	176945427	176945427	Missense_Mutation	SNP	G	A	1.75%	10.59%	
P5	PTH2R	2	209355379	209355379	Missense_Mutation	SNP	A	G	40.62%	8.93%	9.38%
P5	ABCA12	2	215917248	215917248	Missense_Mutation	SNP	C	A	31.88%	20.59%	15.09%
P5	SLC6A11	3	10857999	10857999	Nonsense_Mutation	SNP	G	T	27.08%	16.67%	25.40%
P5	DYNC1LI1	3	32612207	32612207	Missense_Mutation	SNP	G	A	21.43%	24.24%	16.13%
P5	PRR23A	3	138724358	138724358	Silent	SNP	G	T	26.74%	24.10%	23.56%
P5	SI	3	164700076	164700076	Missense_Mutation	SNP	G	T	23.26%	16.67%	10.71%
P5	FYTTD1	3	197505316	197505316	Missense_Mutation	SNP	T	A	2.78%	10.77%	
P5	CPEB2	4	15055806	15055806	Missense_Mutation	SNP	A	G	15.25%	11.67%	14.93%
P5	BEND4	4	42119622	42119622	Missense_Mutation	SNP	G	T	14.53%	7.69%	11.02%
P5	LRRC66	4	52863988	52863988	Missense_Mutation	SNP	C	A	10.00%	11.54%	8.22%
P5	C4orf54	4	100575257	100575257	Missense_Mutation	SNP	C	A	36.84%	15.69%	41.43%
P5	OTUD4	4	146062562	146062562	Silent	SNP	T	A	32.50%	19.51%	28.95%
P5	MARCHF1	4	164507074	164507074	Missense_Mutation	SNP	G	T	41.67%	17.78%	38.46%
P5	CTNND2	5	10992693	10992693	Missense_Mutation	SNP	G	T	38.39%	40.10%	39.88%
P5	SPEF2	5	35694414	35694414	Missense_Mutation	SNP	G	T	20.41%	13.75%	18.67%
P5	C9	5	39341665	39341665	Silent	SNP	G	A	25.23%	28.95%	23.58%
P5	MAP1B	5	71493031	71493031	Silent	SNP	C	A	1.72%	14.58%	
P5	PCDHA9	5	140230104	140230104	Missense_Mutation	SNP	C	T	1.73%	4.05%	
P5	PCDHB5	5	140516868	140516868	Missense_Mutation	SNP	G	T	3.28%	10.81%	5.00%
P5	DSP	6	7585693	7585693	Missense_Mutation	SNP	C	A	18.31%	17.07%	22.22%
P5	BMP6	6	7862704	7862704	Missense_Mutation	SNP	G	T	17.14%	23.72%	18.60%
P5	POM121L2	6	27279832	27279832	Silent	SNP	G	T	21.64%	26.36%	21.34%

P5	DNAH8	6	38705748	38705748	Missense_Mutation	SNP	G	T	23.26%	21.05%	21.88%
P5	EYS	6	64940626	64940626	Missense_Mutation	SNP	G	T	16.67%	18.00%	22.81%
P5	RADIL	7	4917306	4917306	Silent	SNP	C	T	23.08%	28.46%	36.57%
P5	OSBPL3	7	24905036	24905036	Missense_Mutation	SNP	A	T	23.08%	34.29%	11.25%
P5	MAGI2	7	78636457	78636457	Missense_Mutation	SNP	G	A	3.85%	7.46%	16.42%
P5	GRHL2	8	102649156	102649156	Missense_Mutation	SNP	G	T	14.93%	7.79%	10.00%
P5	PKHD1L1	8	110465104	110465104	Splice_Site	SNP	G	T	6.98%	16.00%	9.09%
P5	FAM135B	8	139164761	139164761	Missense_Mutation	SNP	C	A	20.17%	36.90%	29.91%
P5	FREM1	9	14851591	14851591	Missense_Mutation	SNP	C	G	24.00%	16.36%	17.33%
P5	PLIN2	9	19121046	19121046	Missense_Mutation	SNP	C	A	12.39%	8.57%	11.58%
P5	MUC5AC	11	1218795	1218795	Nonsense_Mutation	SNP	C	A	24.27%	23.81%	21.37%
P5	SYT8	11	1858594	1858594	Missense_Mutation	SNP	G	T	24.76%	22.56%	31.16%
P5	OR51L1	11	5020429	5020429	Missense_Mutation	SNP	C	A	33.33%	12.96%	21.25%
P5	SPON1	11	14277284	14277284	Missense_Mutation	SNP	G	C	23.75%	6.35%	11.84%
P5	LUZP2	11	24759791	24759791	Missense_Mutation	SNP	G	T	21.05%	8.06%	5.56%
P5	SLC17A8	12	100790176	100790176	Silent	SNP	G	T	18.97%	19.15%	19.35%
P5	OR4N2	14	20296350	20296350	Missense_Mutation	SNP	T	A	5.83%	4.65%	4.89%
P5	CCDC175	14	59992066	59992066	Silent	SNP	T	C	8.55%	3.68%	5.51%
P5	SERPINA11	14	94909015	94909015	Silent	SNP	G	C	31.48%	17.50%	27.27%
P5	AQR	15	35159810	35159810	Missense_Mutation	SNP	C	G	19.30%	22.50%	5.26%
P5	PTX4	16	1537536	1537536	Missense_Mutation	SNP	G	C	21.30%	21.66%	33.81%
P5	CDH8	16	61935254	61935254	Silent	SNP	G	T	18.95%	28.30%	17.76%
P5	HYDIN	16	70913201	70913201	Missense_Mutation	SNP	T	A	18.37%	21.05%	12.50%
P5	BCO1	16	81298249	81298249	Missense_Mutation	SNP	A	T	2.99%	10.67%	
P5	MEAK7	16	84529514	84529514	Missense_Mutation	SNP	G	T	23.08%	9.09%	7.50%
P5	ACSF3	16	89167540	89167540	Missense_Mutation	SNP	G	C	10.81%	9.52%	7.94%
P5	GPS2	17	7216550	7216550	Missense_Mutation	SNP	T	A	16.16%	9.43%	16.36%
P5	MYH10	17	8415841	8415841	Silent	SNP	A	G	15.73%	7.02%	12.36%
P5	DNAH9	17	11568158	11568158	Silent	SNP	G	T	28.81%	13.95%	22.22%
P5	EPN2	17	19215363	19215363	Splice_Site	SNP	A	T	36.76%	35.90%	18.42%
P5	STAT3	17	40497675	40497680	Splice_Site	DEL	TCTAT	-	32.56%	15.38%	38.03%
P5	SDK2	17	71431713	71431713	Missense_Mutation	SNP	G	T	5.88%	3.80%	4.46%
P5	GPR142	17	72365654	72365654	Missense_Mutation	SNP	C	G	21.48%	11.36%	21.09%
P5	SALL3	18	76753528	76753528	Missense_Mutation	SNP	C	A	25.71%	27.82%	13.75%
P5	TYK2	19	10473236	10473236	Nonsense_Mutation	SNP	G	A	26.55%	33.64%	36.45%
P5	ZNF628	19	55994286	55994286	Missense_Mutation	SNP	G	C	21.82%	23.08%	20.71%
P5	TPX2	20	30381691	30381691	Missense_Mutation	SNP	C	A	3.12%	6.45%	1.92%
P5	DSCAM	21	41423892	41423892	Silent	SNP	C	A	35.85%	11.32%	16.67%
P5	MYO18B	22	26164423	26164423	Silent	SNP	C	T	8.62%	6.15%	4.12%
P5	TIMP3	22	33253261	33253261	Missense_Mutation	SNP	C	A	23.73%	9.09%	8.96%
P5	CYSLTR1	X	77528808	77528808	Missense_Mutation	SNP	C	G	29.79%	51.40%	37.98%
P5	IL9R	X	155235766	155235766	Missense_Mutation	SNP	G	T	10.89%	10.17%	11.36%
P5	SLC30A2	1	26366354	26366354	Missense_Mutation	SNP	G	C	26.42%	35.48%	8.82%
P5	ZNF593	1	26496476	26496476	Translation_Start_Site	SNP	T	G	35.71%	27.27%	13.24%
P5	LRRC7	1	70300517	70300517	Missense_Mutation	SNP	C	A	21.52%	16.90%	9.64%
P5	PEAR1	1	156879827	156879827	Missense_Mutation	SNP	T	A	16.43%	17.76%	19.05%
P5	FASLG	1	172635039	172635039	Missense_Mutation	SNP	C	A	29.58%	25.42%	13.95%
P5	NCF2	1	183536059	183536062	In_Frame_Del	DEL	GGC	-	15.62%	30.43%	9.52%
P5	HHIPL2	1	222717425	222717425	Missense_Mutation	SNP	T	G	19.29%	14.02%	22.86%
P5	DYSF	2	71797817	71797817	Silent	SNP	G	C	16.02%	13.86%	20.22%
P5	FER1L5	2	97364550	97364550	Missense_Mutation	SNP	C	A	14.36%	13.77%	23.90%
P5	STK11IP	2	220472845	220472845	Silent	SNP	G	A	16.27%	12.28%	21.35%
P5	TRIP12	2	230643235	230643235	Missense_Mutation	SNP	C	A	23.85%	13.97%	23.36%
P5	THAP4	2	242572814	242572814	Missense_Mutation	SNP	C	A	18.18%	11.40%	26.00%
P5	CNTN3	3	74344351	74344351	Missense_Mutation	SNP	C	A	32.86%	30.00%	34.83%
P5	ZBTB11	3	101383944	101383944	Missense_Mutation	SNP	A	C	2.20%		6.60%
P5	P2RY1	3	152554150	152554150	Silent	SNP	G	T	26.50%	20.59%	22.60%
P5	SLITRK3	3	164907575	164907575	Silent	SNP	G	C	19.64%	18.42%	25.95%
P5	FAM53A	4	1657303	1657303	Missense_Mutation	SNP	G	C	1.31%	1.75%	3.54%
P5	BOD1L1	4	13593577	13593577	Missense_Mutation	SNP	C	A	12.23%	5.00%	6.38%
P5	ANAPC4	4	25419334	25419334	Silent	SNP	G	C	43.94%	20.73%	41.24%
P5	GABRG1	4	46086014	46086014	Missense_Mutation	SNP	G	A	36.54%	8.57%	18.42%
P5	FREM3	4	144620638	144620638	Silent	SNP	A	G	32.89%	18.52%	42.47%
P5	SKP2	5	36166681	36166681	Silent	SNP	G	T	22.31%	25.47%	23.27%
P5	C5orf52	5	157106997	157106997	Missense_Mutation	SNP	C	G	30.43%	32.86%	32.04%
P5	GABRG2	5	161524759	161524759	Missense_Mutation	SNP	C	A	27.16%	28.74%	39.77%
P5	PRSS16	6	27219776	27219776	Missense_Mutation	SNP	G	T	21.54%	14.04%	6.84%
P5	PRRT1	6	32118496	32118496	Silent	SNP	G	C	12.61%	11.11%	5.34%
P5	SYNCRIP	6	86350259	86350259	Missense_Mutation	SNP	C	A	22.64%	19.70%	17.50%
P5	AOAH	7	36634021	36634021	Silent	SNP	G	A	20.41%	28.95%	14.29%
P5	BRAF	7	140476827	140476827	Missense_Mutation	SNP	T	C	18.31%	3.33%	6.41%
P5	MGAM2	7	141898451	141898451	Missense_Mutation	SNP	C	A	2.17%	2.13%	15.91%
P5	DPP6	7	154585852	154585852	Missense_Mutation	SNP	C	A	6.45%	10.80%	4.97%
P5	TRPA1	8	72981279	72981279	Silent	SNP	G	T	20.00%	19.75%	31.03%
P5	PKHD1L1	8	110416866	110416866	Missense_Mutation	SNP	C	A	13.43%	20.25%	9.20%
P5	CDKN2A	9	21974771	21974771	Missense_Mutation	SNP	G	T	4.93%	3.65%	4.73%
P5	PFKFB3	10	6188410	6188410	Silent	SNP	G	T	20.55%	22.86%	10.11%
P5	OR5B3	11	58170097	58170097	Silent	SNP	G	A	23.17%	17.24%	33.33%
P5	OR5B2	11	58190613	58190613	Missense_Mutation	SNP	A	T	28.72%	25.00%	30.00%

P5	CCDC86	11	60610310	60610310	Missense_Mutation	SNP	G	C	28.87%	23.85%	30.09%
P5	LRP5	11	68197155	68197155	Silent	SNP	G	T	18.18%	29.27%	20.27%
P5	LRRIQ1	12	85515500	85515500	Missense_Mutation	SNP	G	C	20.00%	21.62%	10.34%
P5	SDSL	12	113871993	113871993	Missense_Mutation	SNP	G	A	28.33%	39.13%	15.69%
P5	OR4Q3	14	20216148	20216148	Missense_Mutation	SNP	C	A	21.74%	20.34%	8.05%
P5	OR4N2	14	20295797	20295797	Missense_Mutation	SNP	G	T	8.56%	7.82%	8.74%
P5	RNASE9	14	21025071	21025071	Missense_Mutation	SNP	C	T	23.66%	29.23%	36.59%
P5	GOLGA8S	15	23606505	23606505	Missense_Mutation	SNP	G	T	2.25%	1.83%	3.48%
P5	KLF13	15	31664373	31664373	Silent	SNP	G	C	15.48%	24.49%	28.04%
P5	CEMIP	15	81201512	81201512	Silent	SNP	G	A	21.15%		11.63%
P5	UNC45A	15	91496233	91496233	Missense_Mutation	SNP	G	T	29.33%	18.52%	11.67%
P5	SETD1A	16	30977342	30977342	Missense_Mutation	SNP	G	T	31.11%	19.48%	25.20%
P5	CDH5	16	66436759	66436759	Missense_Mutation	SNP	G	T	23.14%	18.03%	25.81%
P5	CNTROB	17	7843025	7843025	Missense_Mutation	SNP	G	T	8.50%	5.00%	3.92%
P5	MYO15A	17	18030452	18030452	Missense_Mutation	SNP	G	A	14.46%	13.64%	31.00%
P5	INTS2	17	59999151	59999151	Missense_Mutation	SNP	G	A	29.37%	16.67%	24.26%
P5	RELCH	18	59854773	59854773	Missense_Mutation	SNP	G	T	17.05%	24.14%	9.52%
P5	LIPE	19	42931244	42931244	Missense_Mutation	SNP	T	A	15.13%	14.29%	7.08%
P5	NLRP9	19	56244187	56244187	Missense_Mutation	SNP	G	T	14.29%	18.99%	13.79%
P5	RALGAPA2	20	20616149	20616149	Missense_Mutation	SNP	C	A	10.40%	12.32%	7.34%
P5	CDH22	20	44839145	44839145	Missense_Mutation	SNP	T	G	2.06%	1.89%	5.94%
P5	ZNF831	20	57829789	57829789	Missense_Mutation	SNP	A	G	29.00%	22.86%	30.85%
P5	NPBWR2	20	62737188	62737188	Missense_Mutation	SNP	A	T	1.90%		5.51%
P5	LPAR4	X	78011189	78011189	Missense_Mutation	SNP	G	A	40.91%	42.00%	40.82%
P5	TBX22	X	79286343	79286343	Missense_Mutation	SNP	G	T	37.21%	51.28%	54.69%
P5	RERE	1	8716182	8716182	Missense_Mutation	SNP	C	G		5.26%	
P5	ZNF806	2	133074768	133074768	Nonsense_Mutation	SNP	A	T		7.53%	
P5	NAB1	2	191524277	191524277	Missense_Mutation	SNP	A	T		6.42%	
P5	DGKQ	4	959001	959001	Missense_Mutation	SNP	C	T		2.48%	
P5	HSPA4	5	132437554	132437554	Missense_Mutation	SNP	G	C		11.11%	
P5	SURF6	9	136199406	136199406	Missense_Mutation	SNP	G	T		3.78%	
P5	ARRB1	11	74994490	74994490	Silent	SNP	C	G		2.54%	
P5	ALG10B	12	38714872	38714872	Missense_Mutation	SNP	C	A		6.25%	
P5	SNRPN	15	25223030	25223030	Missense_Mutation	SNP	C	T		6.90%	
P5	HYAL3	3	50332189	50332189	Missense_Mutation	SNP	A	T		1.56%	6.67%
P5	PRR18	6	166721302	166721302	Missense_Mutation	SNP	G	A		8.33%	24.24%
P5	KCNJ4	22	38823801	38823801	Missense_Mutation	SNP	G	T		0.92%	5.26%
P5	ZSWIM5	1	45486350	45486350	Missense_Mutation	SNP	C	T			6.32%
P5	CALM2	2	47387923	47387923	Missense_Mutation	SNP	C	T			5.94%
P5	PSD4	2	113958877	113958877	Nonsense_Mutation	SNP	C	A			4.17%
P5	GAK	4	860794	860794	Missense_Mutation	SNP	G	C			2.06%
P5	MGAM2	7	141896548	141896548	Missense_Mutation	SNP	T	C			6.32%
P5	RGS22	8	101014464	101014464	Missense_Mutation	SNP	G	T			6.59%
P5	GNA14	9	80040582	80040582	Missense_Mutation	SNP	G	T			11.11%
P6	AJAP1	1	4772228	4772228	Missense_Mutation	SNP	C	T	23.57%	30.28%	30.56%
P6	ANGPTL7	1	11252422	11252422	Missense_Mutation	SNP	C	A	26.00%	22.86%	17.39%
P6	ARHGEF10L	1	17991081	17991081	Silent	SNP	G	T	24.29%	27.05%	30.21%
P6	COL11A1	1	103377734	103377734	Missense_Mutation	SNP	G	T	13.51%	15.91%	27.08%
P6	LCE2B	1	152659355	152659355	Silent	SNP	C	A	15.85%	16.30%	22.47%
P6	HMCN1	1	185897706	185897706	Missense_Mutation	SNP	G	T	19.35%	22.50%	11.63%
P6	C1orf116	1	207196362	207196362	Missense_Mutation	SNP	T	A	40.94%	51.56%	40.65%
P6	TAF1A	1	222732099	222732099	Missense_Mutation	SNP	C	G	14.29%	26.67%	15.62%
P6	TAF5L	1	229738285	229738285	Missense_Mutation	SNP	T	A	24.19%	20.93%	13.75%
P6	GCSAML	1	247719712	247719713	Frame_Shift_Del	DEL	C	-	25.50%	28.83%	16.15%
P6	OR2M3	1	248366613	248366613	Missense_Mutation	SNP	G	C	22.17%	24.54%	17.24%
P6	MFSD9	2	103348810	103348810	Missense_Mutation	SNP	C	A	15.38%	14.55%	30.77%
P6	TFCP2L1	2	122004403	122004403	Silent	SNP	C	T	24.18%	23.57%	26.67%
P6	POTEF	2	130877699	130877699	Missense_Mutation	SNP	C	A	24.72%	19.15%	22.37%
P6	GPR39	2	133174958	133174958	Missense_Mutation	SNP	T	G	1.82%	8.22%	1.77%
P6	MBD5	2	149227273	149227273	Missense_Mutation	SNP	G	T	31.58%	16.98%	13.51%
P6	TBR1	2	162273279	162273279	Missense_Mutation	SNP	C	T	26.10%	27.93%	23.83%
P6	HOXD4	2	177016574	177016574	Silent	SNP	G	T	26.13%	13.46%	22.00%
P6	LARS2	3	45557746	45557746	Missense_Mutation	SNP	G	T	15.69%	17.39%	
P6	RAP2B	3	152880613	152880613	Missense_Mutation	SNP	T	C	37.69%	45.30%	52.07%
P6	RNF4	4	2515543	2515543	Silent	SNP	A	C	18.31%	20.93%	29.82%
P6	MAN2B2	4	6621763	6621763	Missense_Mutation	SNP	G	A	7.10%	9.41%	2.21%
P6	SLIT2	4	20569146	20569146	Missense_Mutation	SNP	G	T	14.81%	13.04%	25.64%
P6	GC	4	72629552	72629552	Missense_Mutation	SNP	G	A	19.05%	25.81%	19.57%
P6	DNAH5	5	13716645	13716645	Missense_Mutation	SNP	T	C	61.82%	66.67%	66.67%
P6	OR14J1	6	29274633	29274633	Missense_Mutation	SNP	C	T	33.33%	19.15%	38.71%
P6	KIAA0895	7	36373500	36373500	Missense_Mutation	SNP	C	A	13.64%	20.59%	20.59%
P6	HGF	7	81332039	81332039	Missense_Mutation	SNP	T	C	18.18%	17.02%	29.41%
P6	RELN	7	103216027	103216027	Missense_Mutation	SNP	G	A	10.29%	14.29%	8.82%
P6	ZNF800	7	127014179	127014179	Nonsense_Mutation	SNP	G	C	17.27%	12.00%	38.30%
P6	FLNC	7	128496876	128496876	Missense_Mutation	SNP	C	T	27.36%	25.33%	32.02%
P6	C8orf74	8	10555198	10555198	Missense_Mutation	SNP	T	C	32.26%	50.54%	42.06%
P6	EBF2	8	25718631	25718631	Missense_Mutation	SNP	C	G	18.95%	16.07%	26.67%
P6	VPS13B	8	100520033	100520033	Missense_Mutation	SNP	G	C	46.25%	50.00%	50.00%
P6	KCNK9	8	140631283	140631283	Missense_Mutation	SNP	C	A	50.00%	46.02%	47.67%

P6	CYP11B1	8	143961087	143961087	Missense_Mutation	SNP	C	T	14.91%	15.43%	17.46%
P6	PARP10	8	145059468	145059468	Missense_Mutation	SNP	G	T	18.63%	18.10%	18.63%
P6	KANK1	9	740869	740869	Missense_Mutation	SNP	G	C		4.84%	
P6	GLIS3	9	4286193	4286193	Missense_Mutation	SNP	C	T	5.88%	5.80%	
P6	FRMPD1	9	37744634	37744634	Missense_Mutation	SNP	T	A	37.68%	20.51%	36.99%
P6	FAM107B	10	14563895	14563895	Silent	SNP	T	C	23.43%	23.45%	23.78%
P6	ACBD7	10	15121039	15121039	Missense_Mutation	SNP	C	T	11.11%	14.71%	25.37%
P6	SORCS1	10	108439411	108439411	Missense_Mutation	SNP	G	T	22.08%	19.51%	15.62%
P6	OR52N2	11	5841766	5841766	Silent	SNP	G	T	25.93%	20.00%	22.73%
P6	KIF18A	11	28058055	28058055	Missense_Mutation	SNP	G	T	20.99%	23.53%	43.24%
P6	OR8H3	11	55890449	55890449	Missense_Mutation	SNP	A	T	18.56%	14.81%	26.92%
P6	GLYAT	11	58480343	58480343	Missense_Mutation	SNP	A	T	14.81%	20.00%	14.29%
P6	CACNA1C	12	2224535	2224535	Missense_Mutation	SNP	G	T	54.46%	52.82%	47.47%
P6	NRIP2	12	2944004	2944004	Missense_Mutation	SNP	C	A	45.30%	50.00%	50.00%
P6	CLEC4C	12	7883509	7883509	Splice_Site	SNP	C	A	54.39%	61.40%	37.21%
P6	LMNTD1	12	25699344	25699344	Missense_Mutation	SNP	G	C	39.74%	31.03%	61.90%
P6	TMCC3	12	94975880	94975880	Silent	SNP	G	A	8.54%	27.85%	18.31%
P6	LMO7	13	76397910	76397910	Silent	SNP	C	T	31.15%	22.50%	24.32%
P6	YLPM1	14	75248606	75248606	Silent	SNP	C	T	21.67%	35.78%	35.90%
P6	GOLGA8R	15	30700409	30700409	Missense_Mutation	SNP	C	G	10.00%	18.18%	18.18%
P6	TRPM1	15	31339445	31339445	Missense_Mutation	SNP	G	A	8.20%	14.29%	12.73%
P6	RYR3	15	34145792	34145792	Missense_Mutation	SNP	C	A	9.38%	16.22%	13.46%
P6	SECISBP2L	15	49284736	49284736	Missense_Mutation	SNP	T	A	25.00%	16.00%	17.07%
P6	CSNK1G1	15	64464185	64464185	Splice_Site	SNP	C	A	24.59%	24.32%	35.19%
P6	KBTBD13	15	65369979	65369979	Nonsense_Mutation	SNP	G	T	17.65%	10.53%	10.45%
P6	CHRNB4	15	78921508	78921508	Missense_Mutation	SNP	G	C	33.33%	36.90%	34.13%
P6	RBFOX1	16	7703882	7703882	Missense_Mutation	SNP	C	T		3.87%	
P6	FCSK	16	70497601	70497601	Missense_Mutation	SNP	G	C	29.19%	25.52%	26.17%
P6	DPEP1	16	89704331	89704331	Missense_Mutation	SNP	G	C	26.99%	25.55%	32.00%
P6	TP53	17	7577559	7577559	Missense_Mutation	SNP	G	C	56.04%	55.81%	60.16%
P6	NCOR1	17	15989699	15989699	Missense_Mutation	SNP	G	C	46.94%	42.39%	55.34%
P6	CPD	17	28749981	28749981	Missense_Mutation	SNP	G	A		13.04%	
P6	CACNA1G	17	48677153	48677153	Missense_Mutation	SNP	T	C	26.34%	22.03%	26.48%
P6	MRC2	17	60767647	60767647	Silent	SNP	G	A	5.43%	2.70%	0.42%
P6	C17orf80	17	71233110	71233110	Missense_Mutation	SNP	C	T	37.93%	13.89%	3.66%
P6	SETBP1	18	42643367	42643367	Missense_Mutation	SNP	G	T	29.35%	30.00%	22.99%
P6	CYP4F12	19	15794449	15794449	Missense_Mutation	SNP	A	G	29.03%	39.22%	34.71%
P6	KMT2B	19	36224189	36224189	Missense_Mutation	SNP	G	A	24.53%	23.40%	34.62%
P6	SIGLEC1	20	3677870	3677870	Missense_Mutation	SNP	C	G	33.54%	38.94%	50.00%
P6	RALGAPA2	20	20505488	20505488	Missense_Mutation	SNP	C	T	6.15%	15.52%	11.67%
P6	KCNQ2	20	62071054	62071054	Missense_Mutation	SNP	A	G	37.04%	17.95%	55.36%
P6	KCNQ2	20	62073860	62073860	Missense_Mutation	SNP	C	A	36.50%	38.02%	43.56%
P6	NPBWR2	20	62738026	62738026	Silent	SNP	G	A	43.75%	34.69%	38.99%
P6	LTN1	21	30330779	30330779	Missense_Mutation	SNP	C	A	17.86%	16.33%	39.58%
P6	PWP2	21	45540964	45540964	Silent	SNP	G	A		3.55%	0.29%
P6	HMOX1	22	35782935	35782935	Silent	SNP	C	T	18.47%	36.84%	14.01%
P6	FAM227A	22	39022305	39022306	Missense_Mutation	DNP	GC	AA	19.28%	17.31%	20.29%
P6	FAM47A	X	34150285	34150287	Frame_Shift_Del	DEL	GC	-	34.38%	51.85%	52.10%
P6	NTRK1	1	156851342	156851342	Nonsense_Mutation	SNP	G	T	35.71%	22.67%	17.57%
P6	SPTA1	1	158612271	158612271	Missense_Mutation	SNP	T	C	6.42%	14.12%	21.79%
P6	NIBAN1	1	184863211	184863211	Missense_Mutation	SNP	C	G	13.64%	26.32%	26.67%
P6	NBEAL1	2	203972762	203972762	Missense_Mutation	SNP	T	G	44.44%	30.77%	29.51%
P6	DES	2	220283541	220283541	Missense_Mutation	SNP	C	A	20.59%	22.95%	21.19%
P6	CRACD	4	57180606	57180606	Missense_Mutation	SNP	G	T	28.25%	15.66%	25.53%
P6	DKK2	4	107847018	107847018	Missense_Mutation	SNP	C	T	11.54%	34.00%	33.33%
P6	HCN1	5	45267200	45267200	Missense_Mutation	SNP	C	G	9.89%	4.26%	9.84%
P6	HECW1	7	43483993	43483993	Missense_Mutation	SNP	C	A	27.97%	25.29%	29.68%
P6	ZNF394	7	99091155	99091155	Silent	SNP	T	C	5.75%	1.52%	14.61%
P6	DLD	7	107557263	107557264	Missense_Mutation	DNP	AG	TA	18.37%	19.05%	12.20%
P6	ZNF777	7	149152503	149152503	Missense_Mutation	SNP	A	G	20.00%	24.56%	23.57%
P6	DNAJB6	7	157202534	157202534	Missense_Mutation	SNP	G	T	26.36%	30.12%	24.09%
P6	MSR1	8	16032798	16032798	Missense_Mutation	SNP	T	G	37.97%	47.83%	51.02%
P6	MSR1	8	16032800	16032800	Missense_Mutation	SNP	T	C	38.16%	48.89%	51.06%
P6	NRG1	8	32406328	32406328	Silent	SNP	G	A	47.79%	31.40%	32.14%
P6	RB1CC1	8	53596517	53596517	Missense_Mutation	SNP	T	A	37.72%	39.34%	37.29%
P6	KIFC2	8	145693964	145693964	Nonsense_Mutation	SNP	C	T	18.38%	15.45%	18.92%
P6	JAK2	9	5054832	5054832	Missense_Mutation	SNP	G	C	10.81%	26.00%	3.92%
P6	JAK2	9	5054867	5054867	Missense_Mutation	SNP	G	C	12.50%	28.57%	7.69%
P6	ECM2	9	95277450	95277450	Missense_Mutation	SNP	C	T	7.89%	2.56%	7.41%
P6	OR51I2	11	5474859	5474859	Silent	SNP	G	A	13.73%	20.83%	23.33%
P6	NLRP14	11	7063903	7063903	Missense_Mutation	SNP	C	G	14.29%	28.38%	20.97%
P6	PLEKHA7	11	16838733	16838733	Missense_Mutation	SNP	C	T	27.98%	16.67%	25.32%
P6	GYS2	12	21693384	21693385	Frame_Shift_Del	DEL	T	-	34.44%	47.83%	53.33%
P6	GYS2	12	21693385	21693385	Nonsense_Mutation	SNP	C	A	33.70%	48.98%	53.19%
P6	DIP2B	12	51089096	51089096	Missense_Mutation	SNP	T	C	11.25%	2.63%	11.11%
P6	BTBD11	12	107712938	107712938	Missense_Mutation	SNP	T	A	50.56%	39.13%	49.48%
P6	RNFT2	12	117187719	117187719	Missense_Mutation	SNP	G	T	12.98%	12.16%	13.17%
P6	NEMF	14	50318562	50318562	Missense_Mutation	SNP	C	T	12.20%	46.15%	29.41%
P6	GSC	14	95236255	95236255	Missense_Mutation	SNP	A	T	32.76%	25.81%	39.53%

P6	HCFC1R1	16	3072908	3072908	Missense_Mutation	SNP	G	A	26.50%	15.66%	27.91%
P6	TANGO6	16	68914513	68914513	Missense_Mutation	SNP	T	A	8.22%	25.45%	16.48%
P6	USP43	17	9549051	9549051	Silent	SNP	G	T	44.93%	50.00%	69.05%
P6	MYH2	17	10424727	10424727	Missense_Mutation	SNP	A	T	46.67%	60.98%	48.33%
P6	KIAA0100	17	26950801	26950801	Missense_Mutation	SNP	C	A	37.89%	39.13%	34.85%
P6	TNRC6C	17	76071288	76071288	Missense_Mutation	SNP	G	A	1.97%	0.84%	
P6	NETO1	18	70451095	70451095	Missense_Mutation	SNP	C	T	29.03%	30.77%	9.68%
P6	STK11	19	1220502	1220502	Nonsense_Mutation	SNP	G	T	40.79%	50.00%	29.23%
P6	FUT6	19	5832268	5832268	Missense_Mutation	SNP	G	T	35.34%	33.33%	45.53%
P6	ZNF335	20	44582470	44582470	Missense_Mutation	SNP	C	T	3.23%	1.37%	0.52%
P6	CD40	20	44756802	44756802	Silent	SNP	G	T	18.39%	15.87%	25.55%
P6	WDR4	21	44273777	44273777	Missense_Mutation	SNP	A	T	24.24%	26.88%	24.32%
P6	COL6A1	21	47422240	47422240	Silent	SNP	G	C	24.11%	18.92%	20.00%
P6	MICAL3	22	18324631	18324631	Missense_Mutation	SNP	T	A	23.31%	20.41%	28.47%
P6	SLITRK2	X	144904011	144904011	Missense_Mutation	SNP	A	C	52.63%	51.16%	58.00%
P6	DNAI3	1	85559162	85559162	Silent	SNP	A	G	25.00%	30.77%	19.05%
P6	LMNA	1	156104748	156104748	Missense_Mutation	SNP	G	T	15.20%	17.76%	21.60%
P6	AHCTF1	1	247039480	247039480	Missense_Mutation	SNP	C	A	20.24%	1.92%	13.89%
P6	LRP1B	2	141771263	141771263	Nonsense_Mutation	SNP	C	A	28.72%	28.81%	15.38%
P6	TTN	2	179422730	179422730	Silent	SNP	G	A	3.33%	23.53%	13.95%
P6	SCN5A	3	38651268	38651268	Missense_Mutation	SNP	G	T	25.00%	26.47%	38.38%
P6	FTMT	5	121187966	121187966	Missense_Mutation	SNP	G	T	33.33%	33.33%	35.96%
P6	BTBD9	6	38561910	38561910	Missense_Mutation	SNP	C	T	20.93%	29.41%	15.22%
P6	COL9A1	6	70944596	70944597	Missense_Mutation	DNP	CC	AG	25.77%	23.53%	28.72%
P6	OR2A4	6	132022422	132022423	Frame_Shift_Del	DEL	C	-	25.00%	25.53%	34.88%
P6	ANK1	8	41655054	41655054	Translation_Start_Site	SNP	C	A	34.12%	30.61%	51.56%
P6	EHMT1	9	140707503	140707503	Missense_Mutation	SNP	A	C	15.85%	16.13%	23.38%
P6	MUC2	11	1096350	1096350	Silent	SNP	G	C	23.71%	22.50%	33.33%
P6	NLRP10	11	7982082	7982082	Silent	SNP	C	A	24.42%	40.00%	16.07%
P6	FEN1	11	61563191	61563191	Nonsense_Mutation	SNP	G	T	17.18%	23.42%	24.84%
P6	OR8B4	11	124294598	124294598	Missense_Mutation	SNP	G	T	18.75%	13.25%	10.53%
P6	ARHGDIB	12	15102748	15102748	Missense_Mutation	SNP	T	C	11.61%	18.97%	10.26%
P6	MYH7	14	23887606	23887606	Missense_Mutation	SNP	C	A	14.67%	15.28%	16.75%
P6	DAZAP1	19	1418347	1418347	Missense_Mutation	SNP	C	A	34.88%	37.50%	46.38%
P6	EFCAB8	20	31455517	31455517	Missense_Mutation	SNP	G	C	23.81%	16.95%	12.99%
P6	PTPRT	20	40944430	40944430	Missense_Mutation	SNP	C	G	4.84%	35.71%	18.75%
P6	CYLC1	X	83127921	83127921	Missense_Mutation	SNP	G	T	49.09%	51.43%	20.59%
P6	DENND4B	1	153916659	153916659	Silent	SNP	G	T	3.90%		
P6	USP17L2	8	11994842	11994842	Silent	SNP	T	C	3.09%		1.18%
P6	RAB2A	8	61484607	61484607	Missense_Mutation	SNP	G	A	6.38%		
P6	PRSS3	9	33796672	33796673	Missense_Mutation	DNP	TG	CA	6.19%		10.67%
P6	TUBA3C	13	19751578	19751578	Missense_Mutation	SNP	A	C	4.38%		
P6	EPB42	15	43500904	43500904	Missense_Mutation	SNP	C	T	4.08%		
P6	NFATC1	18	77170743	77170743	Silent	SNP	G	A	2.93%		
P6	PRUNE1	1	150999783	150999783	Missense_Mutation	SNP	G	A			7.14%
P6	RYR2	1	237948272	237948272	Missense_Mutation	SNP	G	T			9.52%
P6	PXDN	2	1647287	1647287	Missense_Mutation	SNP	A	T			5.77%
P6	HOXD3	2	177036381	177036381	Missense_Mutation	SNP	G	C			5.34%
P6	BLTP1	4	123128763	123128763	Silent	SNP	A	G			8.57%
P6	TENM3	4	183268029	183268029	Nonsense_Mutation	SNP	C	A			6.19%
P6	AGXT2	5	35035390	35035390	Missense_Mutation	SNP	G	T			6.48%
P6	POU5F1B	8	128428248	128428248	Missense_Mutation	SNP	G	A			2.97%
P6	PHF20L1	8	133848892	133848892	Missense_Mutation	SNP	G	C			5.71%
P6	GOLGA2	9	131019553	131019553	Silent	SNP	G	A			6.57%
P6	MICALCL	11	12316253	12316253	Silent	SNP	C	A			3.29%
P6	ARL1	12	101799704	101799704	Silent	SNP	A	T			12.90%
P7	CEP104	1	3753074	3753074	Silent	SNP	C	G			1.92%
P7	KIF1B	1	10357292	10357292	Silent	SNP	G	A			7.08%
P7	SRRM1	1	24987243	24987243	Missense_Mutation	SNP	C	A			6.96%
P7	STMN1	1	26227557	26227557	Missense_Mutation	SNP	G	A			4.49%
P7	PRDX1	1	45981333	45981333	Missense_Mutation	SNP	G	T			1.68%
P7	PATJ	1	62350042	62350042	Nonsense_Mutation	SNP	C	A			15.79%
P7	TNN	1	175116081	175116081	Silent	SNP	G	A			4.40%
P7	IPO9	1	201837891	201837891	Silent	SNP	C	T			4.04%
P7	MTR	1	236979825	236979825	Missense_Mutation	SNP	C	A		3.03%	7.07%
P7	SRSF7	2	38975259	38975259	Missense_Mutation	SNP	G	T			21.31%
P7	UGP2	2	64069335	64069335	Missense_Mutation	SNP	C	A			3.90%
P7	GGCX	2	85787963	85787963	Silent	SNP	G	A			4.73%
P7	MFSD9	2	103343322	103343322	Silent	SNP	G	A			1.70%
P7	CSRNP3	2	166533010	166533010	Silent	SNP	G	A			12.50%
P7	CASP10	2	202073819	202073819	Missense_Mutation	SNP	C	A			3.68%
P7	TRPM8	2	234873306	234873306	Missense_Mutation	SNP	G	A			10.17%
P7	IQCA1	2	237246962	237246962	Silent	SNP	G	T			12.50%
P7	DCP1A	3	53324865	53324865	Missense_Mutation	SNP	G	A			3.57%
P7	MIX23	3	122078754	122078754	Missense_Mutation	SNP	G	A			2.75%
P7	USP13	3	179481932	179481932	Missense_Mutation	SNP	G	T			2.18%
P7	TAPT1	4	16204186	16204186	Silent	SNP	G	T			3.24%
P7	FIP1L1	4	54325493	54325493	Missense_Mutation	SNP	T	G			10.71%
P7	NPNT	4	106879653	106879653	Silent	SNP	G	A			11.96%

P7	PJA2	5	108673025	108673025	Missense_Mutation	SNP	G	T			8.33%
P7	PDE6A	5	149294520	149294520	Missense_Mutation	SNP	G	T			13.73%
P7	DTNBP1	6	15638018	15638018	Missense_Mutation	SNP	G	A			17.65%
P7	GRIK2	6	102337524	102337524	Missense_Mutation	SNP	C	A			2.47%
P7	SMOC2	6	168910652	168910652	Missense_Mutation	SNP	C	A			13.95%
P7	TYW1	7	66463162	66463162	Nonsense_Mutation	SNP	C	T			15.09%
P7	PCLO	7	82785683	82785683	Missense_Mutation	SNP	G	C			2.45%
P7	ABCB1	7	87145870	87145870	Silent	SNP	T	C			15.52%
P7	NRF1	7	129297394	129297394	Missense_Mutation	SNP	C	T			5.42%
P7	INTS9	8	28651392	28651392	Silent	SNP	G	A			5.22%
P7	KIF13B	8	29053710	29053710	Silent	SNP	C	T			10.67%
P7	HEY1	8	80677995	80677995	Missense_Mutation	SNP	C	T			4.62%
P7	EFR3A	8	132966120	132966120	Missense_Mutation	SNP	G	A			6.72%
P7	FREM1	9	14756409	14756409	Silent	SNP	G	A			4.43%
P7	APTX	9	32985972	32985972	Missense_Mutation	SNP	C	T			4.71%
P7	GPR21	9	125797853	125797853	Silent	SNP	C	T			8.54%
P7	NUP214	9	134067622	134067622	Missense_Mutation	SNP	C	A			4.90%
P7	DHTKD1	10	12142224	12142224	Silent	SNP	C	T		1.07%	1.94%
P7	MTPAP	10	30638181	30638181	Silent	SNP	G	T			1.95%
P7	NFKB2	10	104159049	104159049	Silent	SNP	C	T			6.38%
P7	SLK	10	105752374	105752374	Missense_Mutation	SNP	G	A			1.90%
P7	SWAP70	11	9754113	9754113	Silent	SNP	C	T			10.53%
P7	PAX6	11	31811528	31811528	Missense_Mutation	SNP	G	T			3.97%
P7	FNBP4	11	47786850	47786850	Silent	SNP	G	T			4.55%
P7	C2CD3	11	73824850	73824850	Silent	SNP	G	T			7.06%
P7	ARHGEF12	11	120340047	120340047	Missense_Mutation	SNP	C	A			4.32%
P7	ERBB3	12	56492679	56492679	Silent	SNP	G	T			3.30%
P7	OTOGL	12	80632677	80632677	Silent	SNP	G	A			13.16%
P7	NEDD1	12	97306563	97306563	Missense_Mutation	SNP	A	G			8.33%
P7	PWP1	12	108104257	108104257	Missense_Mutation	SNP	C	T			8.16%
P7	KNTC1	12	123024289	123024289	Missense_Mutation	SNP	G	T			5.00%
P7	SKA3	13	21732131	21732131	Missense_Mutation	SNP	G	A		4.76%	12.09%
P7	RNF17	13	25356007	25356007	Missense_Mutation	SNP	C	A			5.15%
P7	MYCBP2	13	77818045	77818045	Missense_Mutation	SNP	C	T			2.54%
P7	PSMB5	14	23495571	23495571	Silent	SNP	C	T			5.79%
P7	ARIH1	15	72855748	72855748	Missense_Mutation	SNP	T	C			4.92%
P7	NDE1	16	15758700	15758700	Missense_Mutation	SNP	C	T			2.04%
P7	NDE1	16	15758710	15758710	Nonsense_Mutation	SNP	C	A			1.44%
P7	RSPRY1	16	57250030	57250030	Silent	SNP	C	A			1.05%
P7	NF1	17	29563032	29563032	Missense_Mutation	SNP	C	A			1.55%
P7	RHOT1	17	30521074	30521074	Nonsense_Mutation	SNP	C	T	3.51%		2.97%
P7	EZH1	17	40856661	40856661	Missense_Mutation	SNP	T	C			12.50%
P7	NPEPPS	17	45623351	45623351	Missense_Mutation	SNP	G	T			3.61%
P7	SPAG9	17	49074010	49074010	Silent	SNP	C	T			10.53%
P7	AFG3L2	18	12344183	12344183	Missense_Mutation	SNP	G	T			15.22%
P7	ST8SIA3	18	55021734	55021734	Missense_Mutation	SNP	G	T			12.00%
P7	ALPK2	18	56278980	56278980	Missense_Mutation	SNP	G	T			6.60%
P7	RTTN	18	67809569	67809569	Missense_Mutation	SNP	C	T		6.41%	8.97%
P7	EXOSC5	19	41895800	41895800	Missense_Mutation	SNP	C	T			3.50%
P7	IGSF23	19	45116963	45116963	Missense_Mutation	SNP	C	T			8.82%
P7	LIG1	19	48660311	48660311	Silent	SNP	G	A			6.74%
P7	TMC2	20	2552858	2552858	Silent	SNP	T	A			5.00%
P7	SEC23B	20	18505636	18505636	Missense_Mutation	SNP	C	A			8.00%
P7	COMMD7	20	31292279	31292279	Missense_Mutation	SNP	C	T			4.02%
P7	KCNQ2	20	62065152	62065152	Silent	SNP	G	A			11.54%
P7	TMPRSS15	21	19653399	19653399	Missense_Mutation	SNP	C	T			13.33%
P7	MORC3	21	37734525	37734525	Missense_Mutation	SNP	G	A			1.68%
P7	PWP2	21	45547826	45547826	Silent	SNP	G	A			6.12%
P7	ATP6V1E1	22	18077327	18077327	Missense_Mutation	SNP	G	T			13.21%
P7	PPARA	22	46594466	46594466	Silent	SNP	C	T			9.09%
P7	TMSB4X	X	12994913	12994913	Missense_Mutation	SNP	C	A			2.69%
P7	PAPPA2	1	176671841	176671841	Missense_Mutation	SNP	C	T	1.41%	0.84%	0.47%
P7	ABCC5	3	183707033	183707033	Missense_Mutation	SNP	G	A	3.33%	5.33%	15.45%
P7	STOX2	4	184930357	184930357	Silent	SNP	G	A		6.52%	1.47%
P7	DDX4	5	55112305	55112305	Silent	SNP	G	A		5.28%	0.91%
P7	VPS52	6	33219669	33219669	Missense_Mutation	SNP	C	T		2.21%	1.80%
P7	PAPSS2	10	89473011	89473011	Silent	SNP	C	T		4.05%	1.22%
P7	ACSL5	10	114154733	114154733	Missense_Mutation	SNP	C	T		4.38%	1.27%
P7	TMEM126B	11	85346816	85346816	Missense_Mutation	SNP	C	T		2.55%	2.81%
P7	TULP3	12	3018747	3018747	Splice_Site	SNP	G	A		5.94%	2.20%
P7	NBEA	13	36242549	36242549	Silent	SNP	G	A		14.58%	2.41%
P7	CSF2RA	X	1404677	1404677	Missense_Mutation	SNP	G	A		1.61%	1.02%
P7	ABCD3	1	94964224	94964224	Missense_Mutation	SNP	G	A	10.53%	2.33%	1.27%
P7	PAPOLG	2	61009827	61009827	Missense_Mutation	SNP	C	A	5.79%		0.57%
P7	HSBP1	16	83843017	83843017	Nonsense_Mutation	SNP	C	T	4.08%		0.29%
P7	TNRC6B	22	40666263	40666263	Missense_Mutation	SNP	G	A	3.12%		0.38%
P7	TNRC6B	22	40666264	40666264	Missense_Mutation	SNP	C	T	3.57%		4.06%
P7	ZMYM4	1	35836056	35836056	Missense_Mutation	SNP	G	A		8.00%	
P7	MAGI3	1	114224839	114224839	Missense_Mutation	SNP	G	A		2.68%	

P7	ANGEL2		1	213180529	213180529	Missense_Mutation	SNP	C	A		10.39%
P7	PUS10		2	61187544	61187544	Missense_Mutation	SNP	G	A		6.38%
P7	ANTXR1		2	69420536	69420536	Missense_Mutation	SNP	C	A		21.82%
P7	MFSD9		2	103343367	103343367	Missense_Mutation	SNP	C	A		2.91%
P7	GCC2		2	109124030	109124030	Missense_Mutation	SNP	G	A		5.31%
P7	TSN		2	122520651	122520651	Silent	SNP	C	T		2.94%
P7	SCN1A		2	166895986	166895986	Nonsense_Mutation	SNP	C	A		8.42%
P7	GIGYF2		2	233621008	233621008	Missense_Mutation	SNP	G	A		6.38%
P7	ATG16L1		2	234178679	234178679	Missense_Mutation	SNP	G	A		4.00%
P7	PRKCI		3	169988343	169988343	Missense_Mutation	SNP	G	T		0.92%
P7	PRKCI		3	169988347	169988347	Nonsense_Mutation	SNP	C	T		1.57%
P7	JAKMIP1		4	6086661	6086661	Missense_Mutation	SNP	C	T		4.30%
P7	CNGA1		4	47944090	47944090	Silent	SNP	A	G		5.04%
P7	KIT		4	55598156	55598156	Missense_Mutation	SNP	T	C		1.17%
P7	NEIL3		4	178274862	178274862	Nonsense_Mutation	SNP	C	A		5.08%
P7	TARS1		5	33445503	33445503	Missense_Mutation	SNP	G	A		5.44%
P7	PJA2		5	108691687	108691687	Missense_Mutation	SNP	C	T		5.13%
P7	PCDHGA9		5	140784887	140784887	Missense_Mutation	SNP	C	T		9.68%
P7	FLT4		5	180047673	180047673	Missense_Mutation	SNP	C	T		9.84%
P7	HIVEP1		6	12164631	12164631	Silent	SNP	C	T		6.37%
P7	UHRF1BP1		6	34831788	34831788	Missense_Mutation	SNP	G	A		2.82%
P7	UBR2		6	42559905	42559905	Missense_Mutation	SNP	C	A		4.14%
P7	CRISP1		6	49815941	49815941	Silent	SNP	G	A		6.30%
P7	TFAP2B		6	50803947	50803947	Missense_Mutation	SNP	C	T		4.84%
P7	ICA1		7	8183506	8183506	Missense_Mutation	SNP	G	T		7.79%
P7	POU6F2		7	39504222	39504222	Silent	SNP	G	A		15.15%
P7	TRRAP		7	98548623	98548623	Missense_Mutation	SNP	C	T		2.60%
P7	HBP1		7	106826913	106826913	Missense_Mutation	SNP	G	T		8.22%
P7	FOXP2		7	114271627	114271627	Silent	SNP	G	A		6.36%
P7	SLC13A1		7	122769465	122769465	Nonsense_Mutation	SNP	G	A		0.70%
P7	FSCN3		7	127234049	127234049	Nonsense_Mutation	SNP	C	T		4.62%
P7	TRAPPC9		8	141445308	141445308	Silent	SNP	G	C		6.93%
P7	AOPEP		9	97555172	97555172	Missense_Mutation	SNP	C	T		3.61%
P7	TNC		9	117803242	117803242	Silent	SNP	A	G		4.83%
P7	OR1L3		9	125438350	125438350	Silent	SNP	C	T		2.61%
P7	GLE1		9	131285026	131285026	Missense_Mutation	SNP	G	A		7.08%
P7	MTPAP		10	30638199	30638199	Silent	SNP	C	T		2.44%
P7	BTAF1		10	93776196	93776196	Missense_Mutation	SNP	C	T		8.33%
P7	COL17A1		10	105815150	105815150	Missense_Mutation	SNP	C	T		3.10%
P7	SMC3		10	112360284	112360284	Missense_Mutation	SNP	C	T		5.03%
P7	PPFIBP2		11	7631622	7631622	Missense_Mutation	SNP	G	A		5.71%
P7	WNK1		12	988754	988754	Missense_Mutation	SNP	C	G		3.06%
P7	LNX2		13	28134010	28134010	Missense_Mutation	SNP	G	A		4.85%
P7	EAPP		14	34993932	34993932	Silent	SNP	G	A		5.45%
P7	COQ6		14	74426216	74426216	Silent	SNP	C	T		6.56%
P7	HIGD2B		15	72968712	72968712	Missense_Mutation	SNP	C	T		2.63%
P7	LDAF1		16	21181897	21181897	Missense_Mutation	SNP	C	T		19.05%
P7	MNT		17	2297635	2297635	Missense_Mutation	SNP	C	A		5.38%
P7	NLK		17	26449689	26449689	Silent	SNP	C	T		10.45%
P7	PSMD3		17	38146438	38146438	Silent	SNP	C	T		2.41%
P7	SLC35B1		17	47783676	47783676	Missense_Mutation	SNP	C	T	8.70%	1.96%
P7	PITPNC1		17	65665672	65665672	Missense_Mutation	SNP	G	A		5.13%
P7	RNF213		17	78263518	78263518	Missense_Mutation	SNP	G	A		6.94%
P7	NEDD4L		18	56010201	56010201	Missense_Mutation	SNP	C	A		3.86%
P7	CYP2F1		19	41626396	41626396	Missense_Mutation	SNP	C	T		12.80%
P7	SIGLEC1		20	3674901	3674901	Nonsense_Mutation	SNP	G	A		1.95%
P7	TPX2		20	30363777	30363777	Missense_Mutation	SNP	C	T		5.37%
P7	TTC3		21	38498383	38498383	Missense_Mutation	SNP	G	A		2.05%
P7	UMODL1		21	43549839	43549839	Missense_Mutation	SNP	G	A		3.47%
P7	UMODL1		21	43549840	43549840	Silent	SNP	G	A		2.97%
P7	TNRC6B		22	40666250	40666250	Silent	SNP	C	T		3.09%
P7	BEX5	X		101409193	101409193	Silent	SNP	T	C		4.02%
P7	PNCK	X		152938496	152938496	Silent	SNP	G	A		11.54%
P7	TNN		1	175116097	175116097	Missense_Mutation	SNP	G	A	11.86%	4.94%
P7	TSNAX		1	231700283	231700283	Missense_Mutation	SNP	G	A	7.87%	0.33%
P7	FANCD2		3	10103889	10103889	Silent	SNP	C	T	4.50%	1.42%
P7	SLC25A13		7	95775911	95775911	Missense_Mutation	SNP	G	A	4.88%	1.30%
P7	ERI3		1	44804979	44804979	Missense_Mutation	SNP	C	T	17.95%	
P7	PRDX1		1	45981345	45981345	Missense_Mutation	SNP	G	A	8.82%	
P7	STRIP1		1	110592094	110592094	Missense_Mutation	SNP	G	A	10.34%	
P7	PELI1		2	64331871	64331871	Silent	SNP	G	A	5.26%	
P7	SUCLG1		2	84676872	84676872	Silent	SNP	C	T	10.00%	
P7	ACVR1		2	158637099	158637099	Silent	SNP	C	T	27.66%	
P7	CACNA2D3		3	54420774	54420774	Silent	SNP	G	A	8.54%	
P7	ACTL6A		3	179291240	179291240	Missense_Mutation	SNP	C	T	6.38%	
P7	ATP13A4		3	193272540	193272540	Missense_Mutation	SNP	C	T	13.56%	
P7	CCDC192		5	127089989	127089989	Silent	SNP	G	A	15.56%	
P7	KIFC1		6	33377445	33377445	Missense_Mutation	SNP	C	T	1.14%	
P7	ABCB4		7	87056229	87056229	Missense_Mutation	SNP	C	T	5.47%	

P7	ZNF277	7	111926944	111926944	Silent	SNP	C	A	4.93%		
P7	EFR3A	8	132966096	132966096	Missense_Mutation	SNP	G	A	14.06%		
P7	ZDHHC13	11	19191997	19191997	Missense_Mutation	SNP	G	A	11.11%		
P7	CLSTN3	12	7294706	7294706	Missense_Mutation	SNP	A	G	5.26%		
P7	MUC19	12	40942407	40942407	Missense_Mutation	SNP	C	T	14.29%		
P7	TEP1	14	20836971	20836971	Missense_Mutation	SNP	G	A	4.83%		
P7	VIPAS39	14	77901708	77901708	Missense_Mutation	SNP	C	T	12.16%		
P7	BEX5	X	101409207	101409207	Missense_Mutation	SNP	C	T	9.52%		
P8	FNBP1L	1	93989834	93989834	Missense_Mutation	SNP	G	A	4.64%		
P8	PDCL3	2	101185442	101185442	Silent	SNP	C	T	5.22%		0.74%
P8	CHCHD5	2	113343556	113343556	Missense_Mutation	SNP	C	A	10.99%		
P8	CCDC141	2	179736905	179736905	Silent	SNP	C	T	26.32%		
P8	ZDHHC23	3	113667733	113667733	Nonsense_Mutation	SNP	C	A	16.42%		
P8	NPNT	4	106819121	106819121	Nonsense_Mutation	SNP	C	A	17.95%		
P8	BLTP1	4	123128704	123128704	Missense_Mutation	SNP	G	A	9.21%		
P8	TRIO	5	14388733	14388733	Missense_Mutation	SNP	C	A	11.43%		
P8	SPZ1	5	79616641	79616641	Missense_Mutation	SNP	G	A	9.66%		
P8	FAF2	5	175926041	175926041	Silent	SNP	G	A	6.21%	2.61%	1.15%
P8	MUCL3	6	30916997	30916997	Silent	SNP	C	G	2.62%		
P8	CNKSR3	6	154762453	154762453	Nonsense_Mutation	SNP	G	T	6.87%		
P8	PALS2	7	24663325	24663325	Silent	SNP	G	A	6.36%		
P8	POLM	7	44113575	44113575	Missense_Mutation	SNP	G	A	7.32%		
P8	ASB4	7	95125116	95125116	Silent	SNP	C	A	2.34%	2.41%	0.83%
P8	SLC12A9	7	100459514	100459514	Silent	SNP	C	T	3.07%		0.73%
P8	C7orf66	7	108524521	108524521	Silent	SNP	T	C	3.61%		
P8	AHCYL2	7	129028909	129028909	Missense_Mutation	SNP	C	A	8.00%		
P8	NSMAF	8	59518509	59518509	Missense_Mutation	SNP	T	G	2.36%		
P8	PABPC1	8	101724942	101724942	Silent	SNP	G	T	4.00%		
P8	CYLC2	9	105767619	105767619	Missense_Mutation	SNP	G	A	4.73%		
P8	GRID1	10	87628803	87628803	Silent	SNP	G	T	15.69%		
P8	TRIM5	11	5687249	5687249	Missense_Mutation	SNP	C	T	6.74%		
P8	C2CD5	12	22670986	22670986	Missense_Mutation	SNP	G	A	6.52%		
P8	ERBB3	12	56489571	56489571	Missense_Mutation	SNP	G	A	3.59%	1.40%	
P8	HSPH1	13	31722604	31722604	Missense_Mutation	SNP	G	T	5.11%	0.88%	
P8	MYH7	14	23899017	23899017	Missense_Mutation	SNP	G	A	9.23%		
P8	SYNM	15	99670257	99670257	Missense_Mutation	SNP	G	C	8.59%		
P8	RHOT1	17	30521071	30521071	Nonsense_Mutation	SNP	C	T	3.61%	3.16%	0.78%
P8	MFSD11	17	74737065	74737065	Missense_Mutation	SNP	C	A	4.76%		
P8	CCDC9	19	47774889	47774889	Missense_Mutation	SNP	C	A	9.09%		
P8	ADGRG2	X	19018069	19018069	Missense_Mutation	SNP	G	T	12.07%		
P8	CASK	X	41383226	41383226	Missense_Mutation	SNP	G	C	18.75%		
P8	TRIM73	7	75028240	75028241	Missense_Mutation	DNP	TG	CA	7.50%	8.63%	4.51%
P8	LAPTM4B	8	98817631	98817631	Silent	SNP	G	A	6.67%		3.50%
P8	EPPK1	8	144940498	144940498	Silent	SNP	G	A	1.56%		3.09%
P8	NCAM1	11	113146065	113146065	Missense_Mutation	SNP	C	T	22.00%		5.32%
P8	DUOXA1	15	45409813	45409813	Missense_Mutation	SNP	T	C	3.19%	2.10%	8.13%
P8	KDM6B	17	7750273	7750273	Missense_Mutation	SNP	G	A	6.82%		7.03%
P8	PTPRG	3	62267261	62267261	Nonsense_Mutation	SNP	G	A	0.48%	6.45%	
P8	CHD6	20	40161878	40161878	Missense_Mutation	SNP	C	T	3.45%	4.88%	
P8	ZNF362	1	33745852	33745852	Silent	SNP	G	A		2.56%	6.73%
P8	NFU1	2	69642353	69642353	Missense_Mutation	SNP	G	A			13.33%
P8	BMPR2	2	203420160	203420160	Missense_Mutation	SNP	G	A			3.85%
P8	MGST2	4	140587195	140587195	Silent	SNP	C	T			5.07%
P8	MGST2	4	140587217	140587217	Missense_Mutation	SNP	C	T			4.32%
P8	RAD17	5	68692355	68692355	Nonsense_Mutation	SNP	G	A		3.28%	6.67%
P8	ADGRB3	6	70049228	70049228	Silent	SNP	G	A			8.24%
P8	TTYH3	7	2701363	2701363	Missense_Mutation	SNP	G	A			4.14%
P8	PCM1	8	17822138	17822138	Missense_Mutation	SNP	G	A			15.00%
P8	UBXN8	8	30608934	30608934	Missense_Mutation	SNP	T	A			18.60%
P8	ABLIM1	10	116225500	116225500	Silent	SNP	G	A			7.41%
P8	PPFIBP2	11	7570757	7570757	Silent	SNP	C	T		0.90%	3.61%
P8	GALNT8	12	4835759	4835759	Silent	SNP	G	A			6.06%
P8	AK7	14	96949450	96949450	Missense_Mutation	SNP	G	A			4.03%
P8	KNOP1	16	19722707	19722707	Missense_Mutation	SNP	G	A			6.45%
P8	PRODH2	19	36304134	36304134	Missense_Mutation	SNP	A	G			33.85%
P8	BLTP1	4	123155972	123155972	Missense_Mutation	SNP	G	A		5.26%	1.16%
P8	USP48	1	22083110	22083110	Missense_Mutation	SNP	C	T		15.79%	
P8	SDCCAG8	1	243456493	243456493	Missense_Mutation	SNP	C	T		6.93%	
P8	XIRP2	2	168104828	168104828	Missense_Mutation	SNP	C	T		6.45%	
P8	RNF14	5	141363078	141363078	Silent	SNP	G	A		11.54%	
P8	FLT4	5	180047710	180047710	Missense_Mutation	SNP	C	T		7.61%	
P8	THBS2	6	169648973	169648973	Missense_Mutation	SNP	G	A		6.90%	
P8	LRGUK	7	133948686	133948686	Missense_Mutation	SNP	G	A		4.49%	
P8	NUP214	9	134062715	134062715	Silent	SNP	A	T		3.61%	
P8	TMEM80	11	700622	700622	Silent	SNP	G	A		11.11%	
P8	PDS5B	13	33344486	33344486	Silent	SNP	G	A		7.50%	
P8	RFX1	19	14104641	14104641	Silent	SNP	C	T		6.67%	
P8	PRPF6	20	62657389	62657389	Missense_Mutation	SNP	G	A		10.17%	
P8	TTC3	21	38524272	38524272	Missense_Mutation	SNP	G	A		8.96%	

P8	PWP2	21	45548163	45548163	Missense_Mutation	SNP	G	A		7.23%	
P9	RFX5	1	151314930	151314930	Missense_Mutation	SNP	G	T	19.12%	28.12%	15.85%
P9	GON4L	1	155785681	155785681	Missense_Mutation	SNP	A	G		13.79%	
P9	IL17RD	3	57143635	57143635	Silent	SNP	G	A		34.78%	10.20%
P9	SLU7	5	159830318	159830318	Missense_Mutation	SNP	G	A	14.29%	28.12%	3.03%
P9	ZFAND3	6	38029502	38029502	Silent	SNP	G	A		8.82%	
P9	AGMO	7	15240916	15240916	Missense_Mutation	SNP	C	A		4.88%	
P9	OSBPL3	7	24849459	24849459	Missense_Mutation	SNP	C	T		20.45%	
P9	CTTNBP2	7	117432449	117432449	Silent	SNP	C	T		8.57%	
P9	GRM8	7	126173521	126173521	Missense_Mutation	SNP	T	A	13.33%	18.18%	
P9	NSMCE2	8	126194431	126194431	Silent	SNP	T	C	24.56%	15.22%	5.97%
P9	ZFAT	8	135521949	135521949	Missense_Mutation	SNP	G	C	6.56%	23.81%	17.36%
P9	GLE1	9	131285000	131285000	Silent	SNP	G	A		9.38%	
P9	GDI2	10	5827897	5827897	Nonsense_Mutation	SNP	G	A		9.09%	
P9	OR5T1	11	56043439	56043439	Missense_Mutation	SNP	T	A	11.11%	6.98%	11.11%
P9	DTX1	12	113531466	113531466	Missense_Mutation	SNP	G	A		10.20%	7.38%
P9	PLEK2	14	67859440	67859440	Missense_Mutation	SNP	G	A	19.05%	50.00%	12.68%
P9	NFAT5	16	69726335	69726335	Silent	SNP	A	G	14.43%	18.42%	10.14%
P9	TP53	17	7578413	7578413	Missense_Mutation	SNP	C	T	37.01%	53.85%	24.42%
P9	MYH8	17	10297741	10297741	Missense_Mutation	SNP	A	G	23.31%	25.00%	10.08%
P9	ERBB2	17	37866662	37866662	Missense_Mutation	SNP	G	T	41.29%	40.00%	25.81%
P9	GRIA3	X	122538620	122538620	Missense_Mutation	SNP	A	G		16.00%	4.71%
P9	F8	X	154157272	154157272	Missense_Mutation	SNP	T	A		21.62%	1.92%
P9	CLSPN	1	36226698	36226698	Missense_Mutation	SNP	T	G	11.11%	3.45%	9.43%
P9	GALNT2	1	230384985	230384985	Missense_Mutation	SNP	G	T	12.00%	11.11%	11.59%
P9	EGFR	7	55259515	55259515	Missense_Mutation	SNP	T	G	31.65%	57.58%	18.89%
P9	SAMD9	7	92731657	92731657	Missense_Mutation	SNP	T	G	4.93%	12.68%	6.42%
P9	CYP17A1	10	104592362	104592362	Missense_Mutation	SNP	G	A	11.59%	14.29%	
P9	ACOT11	1	55065006	55065006	Missense_Mutation	SNP	G	A	15.74%	18.75%	14.29%
P9	BLTP1	4	123207735	123207735	Missense_Mutation	SNP	G	A		15.19%	10.81%
P9	MTFR2	6	136564103	136564103	Silent	SNP	C	T		3.51%	9.23%
P9	ACTB	7	5568942	5568942	Missense_Mutation	SNP	G	C		38.64%	4.90%
P9	USP42	7	6189916	6189916	Missense_Mutation	SNP	A	C	14.53%	2.86%	4.81%
P9	CEP126	11	101832855	101832855	Silent	SNP	T	G	18.75%	25.00%	10.61%
P9	GTPBP3	19	17448868	17448868	Silent	SNP	C	T	18.18%	11.11%	24.24%
P9	USP33	1	78163079	78163079	Nonsense_Mutation	SNP	G	A	7.69%		
P9	CELSR2	1	109803850	109803850	Missense_Mutation	SNP	G	A	5.22%		
P9	NBAS	2	15676599	15676599	Nonsense_Mutation	SNP	C	T	6.25%		
P9	SLC6A20	3	45801444	45801444	Missense_Mutation	SNP	C	T	13.64%		10.00%
P9	KIAA0825	5	93807384	93807384	Missense_Mutation	SNP	G	T	11.11%		
P9	OR9A2	7	142724129	142724129	Missense_Mutation	SNP	A	C	8.25%		
P9	CNPY1	7	155295790	155295790	Missense_Mutation	SNP	T	G	3.68%		
P9	PRMT3	11	20486028	20486028	Missense_Mutation	SNP	C	A	5.56%		
P9	TMEM151A	11	66061895	66061895	Missense_Mutation	SNP	G	A	6.74%		
P9	RAB20	13	111213780	111213780	Silent	SNP	G	T	8.96%		
P9	ZNF813	19	53993731	53993731	Missense_Mutation	SNP	C	T	5.94%		
P9	ZNF671	19	58238819	58238819	Silent	SNP	C	T	3.87%		
P9	CNGA2	X	150911895	150911895	Nonsense_Mutation	SNP	G	A	10.67%		
P10	MTOR	1	11188084	11188084	Missense_Mutation	SNP	T	A		16.07%	9.52%
P10	HTR1D	1	23520224	23520224	Missense_Mutation	SNP	C	A		16.07%	5.56%
P10	FUCA1	1	24194745	24194745	Missense_Mutation	SNP	G	A		19.15%	5.26%
P10	ARID1A	1	27023307	27023307	Nonsense_Mutation	SNP	C	G	25.81%	22.86%	37.04%
P10	CD1B	1	158299753	158299753	Missense_Mutation	SNP	T	C	18.75%	20.00%	
P10	CEP350	1	180044230	180044230	Missense_Mutation	SNP	C	T		5.93%	
P10	DENND1B	1	197515905	197515905	Splice_Site	SNP	T	A	27.59%	21.21%	22.58%
P10	SLC30A10	1	220091634	220091634	Silent	SNP	T	C	16.00%	19.51%	11.76%
P10	RYR2	1	237660038	237660038	Missense_Mutation	SNP	T	A	14.29%	16.00%	17.65%
P10	TRIM58	1	248020642	248020642	Missense_Mutation	SNP	G	A	17.78%	26.67%	36.36%
P10	OR2T11	1	248790114	248790114	Missense_Mutation	SNP	C	A	19.40%	41.98%	22.06%
P10	NBAS	2	15608641	15608641	Missense_Mutation	SNP	C	A	5.63%	24.32%	14.29%
P10	STRN	2	37105158	37105158	Missense_Mutation	SNP	G	C	18.64%	19.15%	29.73%
P10	REG1A	2	79349162	79349162	Missense_Mutation	SNP	C	A	17.02%	12.07%	22.50%
P10	EPB41L5	2	120831693	120831693	Silent	SNP	C	T	12.90%	19.15%	15.09%
P10	LRP1B	2	141081604	141081604	Missense_Mutation	SNP	A	T	9.23%	13.95%	9.09%
P10	PRKRA	2	179300964	179300964	Missense_Mutation	SNP	C	A	18.35%	7.94%	15.85%
P10	DNAJC10	2	183584873	183584873	Missense_Mutation	SNP	G	C	12.28%	6.67%	16.00%
P10	CALCRL	2	188216647	188216647	Missense_Mutation	SNP	C	A		17.95%	12.64%
P10	LRTM1	3	54958843	54958843	Missense_Mutation	SNP	T	C	16.07%	15.22%	23.40%
P10	ST3GAL6	3	98506886	98506886	Silent	SNP	T	C	14.29%	16.28%	18.75%
P10	ST3GAL6	3	98506896	98506896	Missense_Mutation	SNP	G	T	17.50%	15.56%	20.59%
P10	DMXL1	5	118486047	118486047	Silent	SNP	T	C		21.95%	18.75%
P10	H2AC6	6	26124842	26124842	Missense_Mutation	SNP	A	C	10.77%	14.29%	2.78%
P10	NOTCH4	6	32188038	32188038	Missense_Mutation	SNP	T	A	28.07%	9.09%	12.82%
P10	MDGA1	6	37620063	37620063	Missense_Mutation	SNP	T	A	9.59%	10.94%	10.20%
P10	UNC5CL	6	41002483	41002483	Missense_Mutation	SNP	G	A		8.14%	
P10	CDC5L	6	44376358	44376358	Missense_Mutation	SNP	A	G	21.74%	52.05%	31.58%
P10	ADGRB3	6	69758226	69758226	Missense_Mutation	SNP	G	C	27.27%	18.92%	15.56%
P10	CD109	6	74476721	74476721	Silent	SNP	G	A	10.40%	5.10%	10.08%
P10	PPIL6	6	109748169	109748169	Missense_Mutation	SNP	C	G	15.79%	12.12%	14.71%

P10	ELFN1	7	1784688	1784688	Missense_Mutation	SNP	C	G	7.14%	12.77%	
P10	SDK1	7	4247779	4247779	Missense_Mutation	SNP	G	A	11.11%	25.00%	5.88%
P10	PKD1L1	7	47882608	47882608	Silent	SNP	C	G	18.84%	6.41%	8.33%
P10	CLIP2	7	73752961	73752961	Missense_Mutation	SNP	A	T	12.50%	16.33%	47.37%
P10	SAMD9L	7	92761778	92761778	Missense_Mutation	SNP	T	A	18.00%	13.33%	23.61%
P10	NYAP1	7	100088660	100088660	Missense_Mutation	SNP	G	T	11.27%	11.27%	4.35%
P10	CSMD1	8	2808727	2808727	Silent	SNP	G	T	16.67%	20.00%	12.90%
P10	ATP6V0D2	8	87111241	87111241	Missense_Mutation	SNP	G	A		5.71%	
P10	RIMS2	8	104898198	104898198	Missense_Mutation	SNP	G	T	20.00%	10.87%	13.89%
P10	CSMD3	8	114186014	114186014	Missense_Mutation	SNP	G	T	4.55%	10.53%	16.22%
P10	TRPS1	8	116616411	116616411	Silent	SNP	A	C	28.89%	20.00%	26.92%
P10	ADGRB1	8	143625012	143625014	Frame_Shift_Del	DEL	CC	-	6.61%	5.08%	2.94%
P10	AK3	9	4722605	4722605	Missense_Mutation	SNP	C	G	24.24%	11.63%	42.11%
P10	FKBP15	9	115935780	115935780	Missense_Mutation	SNP	C	A	27.59%	13.16%	6.25%
P10	MCM10	10	13228267	13228267	Missense_Mutation	SNP	A	G	30.77%	12.20%	15.62%
P10	SKIDA1	10	21806424	21806424	Missense_Mutation	SNP	G	A	16.18%	15.00%	19.35%
P10	PGBD3	10	50723552	50723552	Nonsense_Mutation	SNP	C	A	14.00%	5.19%	18.00%
P10	OR51S1	11	4869889	4869889	Missense_Mutation	SNP	G	T		25.71%	13.64%
P10	RAG1	11	36597740	36597741	Frame_Shift_Del	DEL	G	-	39.13%	73.13%	45.83%
P10	OR8J3	11	55905016	55905016	Missense_Mutation	SNP	T	G	13.70%	15.38%	19.05%
P10	VEGFB	11	64003459	64003459	Missense_Mutation	SNP	G	T	12.94%	44.44%	35.71%
P10	TRPC6	11	101342055	101342055	Missense_Mutation	SNP	C	A	19.67%	52.54%	32.79%
P10	RERG	12	15264322	15264322	Missense_Mutation	SNP	C	A	13.64%	16.33%	10.64%
P10	AMHR2	12	53823306	53823306	Missense_Mutation	SNP	C	G	16.67%	7.32%	5.77%
P10	PWP1	12	108104277	108104277	Silent	SNP	A	T		4.23%	
P10	GPR12	13	27333191	27333191	Silent	SNP	C	T	30.38%	56.94%	49.09%
P10	ATP7B	13	52523898	52523898	Missense_Mutation	SNP	C	A	35.71%	46.99%	28.05%
P10	MAGEL2	15	23890411	23890411	Missense_Mutation	SNP	A	C	22.58%	18.75%	5.88%
P10	HERC2	15	28510993	28510993	Missense_Mutation	SNP	C	A	11.69%	15.22%	25.93%
P10	TLN2	15	63040567	63040567	Missense_Mutation	SNP	T	C		7.78%	
P10	NFATC2IP	16	28962704	28962704	Silent	SNP	G	A		16.28%	4.76%
P10	CMTR2	16	71318159	71318159	Missense_Mutation	SNP	T	C	8.42%	7.09%	16.51%
P10	TP53	17	7578538	7578538	Missense_Mutation	SNP	T	A	51.11%	55.41%	73.91%
P10	COPS3	17	17152239	17152239	Splice_Site	SNP	C	T		8.11%	
P10	GSDMB	17	38062173	38062173	Missense_Mutation	SNP	G	C		18.60%	8.33%
P10	CLUL1	18	619274	619274	Missense_Mutation	SNP	T	G	18.10%	40.80%	21.43%
P10	PIP5K1C	19	3639015	3639015	Splice_Site	SNP	C	A	6.33%	13.24%	
P10	ATP13A1	19	19774087	19774087	Missense_Mutation	SNP	C	G	32.14%	28.00%	54.55%
P10	PLCB1	20	8639259	8639259	Missense_Mutation	SNP	G	C	16.67%	8.57%	10.00%
P10	TMPRSS15	21	19666695	19666695	Missense_Mutation	SNP	T	C	10.91%	10.23%	25.00%
P10	NXF2B	X	101615536	101615536	Nonsense_Mutation	SNP	T	A		6.58%	13.21%
P10	SPANXD	X	140785679	140785679	Silent	SNP	G	T		17.78%	6.78%
P10	TNNI3K	1	74835973	74835973	Missense_Mutation	SNP	A	T		16.25%	8.33%
P10	ETV3L	1	157062895	157062896	Frame_Shift_Del	DEL	C	-	12.70%	9.80%	15.15%
P10	AIM2	1	159043247	159043247	Missense_Mutation	SNP	C	T	11.36%	21.05%	21.88%
P10	ALDH9A1	1	165664526	165664526	Missense_Mutation	SNP	C	G	11.90%	25.53%	25.00%
P10	PTGS2	1	186645709	186645709	Frame_Shift_Ins	INS	-	AC	13.79%	27.66%	14.04%
P10	CCDC74A	2	132290488	132290488	Missense_Mutation	SNP	C	A	7.83%	21.28%	13.51%
P10	THSD7B	2	137813988	137813988	Splice_Site	SNP	A	G		23.64%	13.04%
P10	GABRA4	4	46973254	46973254	Splice_Site	SNP	T	A	23.68%	34.09%	15.56%
P10	ADAMTS3	4	73179408	73179408	Missense_Mutation	SNP	C	G	7.41%	20.25%	9.30%
P10	HCN1	5	45645439	45645440	Missense_Mutation	DNP	CC	AA	18.18%	13.10%	14.52%
P10	RGS7BP	5	63802615	63802615	Missense_Mutation	SNP	T	C	35.82%	37.50%	31.58%
P10	ARHGEF28	5	73045774	73045774	Missense_Mutation	SNP	G	T	59.62%	45.00%	18.75%
P10	N4BP3	5	177546676	177546676	Missense_Mutation	SNP	C	T	8.43%	15.94%	14.29%
P10	ZFP57	6	29641198	29641198	Silent	SNP	G	A	13.51%	20.00%	25.81%
P10	TFAP2B	6	50805754	50805754	Silent	SNP	C	A	8.89%	20.00%	13.73%
P10	SPATA31C2	9	90746828	90746828	Missense_Mutation	SNP	G	C		8.57%	17.65%
P10	CPXM2	10	125622170	125622170	Missense_Mutation	SNP	C	A	19.35%	43.48%	40.91%
P10	CPXM2	10	125622173	125622173	Missense_Mutation	SNP	T	A	18.75%	42.55%	34.62%
P10	CCDC59	12	82752132	82752132	Silent	SNP	C	G	30.63%	27.27%	21.43%
P10	GOLGA3	12	133393195	133393195	Nonsense_Mutation	SNP	C	A	19.15%	32.35%	27.78%
P10	DACH1	13	72049312	72049312	Missense_Mutation	SNP	G	T	52.38%	40.00%	13.64%
P10	RNF31	14	24629493	24629493	Silent	SNP	G	C	15.38%	32.43%	16.67%
P10	IL27	16	28511095	28511095	Silent	SNP	G	T	9.47%	15.00%	20.00%
P10	CHST6	16	75513711	75513711	Missense_Mutation	SNP	C	G	8.45%	15.00%	15.15%
P10	LAMA3	18	21343486	21343486	Missense_Mutation	SNP	A	G		18.57%	13.89%
P10	MEX3C	18	48703673	48703673	Missense_Mutation	SNP	C	A		17.86%	13.89%
P10	CACNA1A	19	13340904	13340904	Silent	SNP	G	A	11.54%	27.66%	12.82%
P10	ZNF180	19	44983545	44983545	Missense_Mutation	SNP	C	A		31.82%	12.77%
P10	FPR3	19	52327190	52327190	Silent	SNP	T	C	27.03%	33.96%	20.00%
P10	LILRA1	19	55106826	55106826	Missense_Mutation	SNP	T	A	10.00%	28.07%	34.78%
P10	KIR3DL2	19	55378042	55378042	Silent	SNP	G	A		11.63%	12.70%
P10	FOXS1	20	30433081	30433081	Missense_Mutation	SNP	C	T	50.83%	67.09%	83.33%
P10	PTPRT	20	41101117	41101117	Silent	SNP	C	T	13.46%	4.00%	10.71%
P10	SCAF4	21	33060709	33060709	Missense_Mutation	SNP	T	C	8.82%	19.44%	5.08%
P10	PAGE1	X	49455913	49455913	Silent	SNP	A	G	19.35%	32.00%	14.81%
P10	RGSL1	1	182517528	182517528	Missense_Mutation	SNP	C	T	13.04%	4.08%	
P10	OR2G2	1	247752516	247752516	Silent	SNP	C	A	12.77%	15.87%	18.33%

P10	TDRD15	2	21361970	21361970	Missense_Mutation	SNP	A	T	6.82%	16.44%	12.66%
P10	UGT1A3	2	234638159	234638159	Silent	SNP	G	T	40.95%	25.32%	19.74%
P10	ATP13A4	3	193156328	193156328	Missense_Mutation	SNP	C	T	15.22%	36.59%	25.81%
P10	KCTD8	4	44176855	44176855	Missense_Mutation	SNP	C	A	13.21%	30.00%	13.04%
P10	ADH1B	4	100232698	100232698	Missense_Mutation	SNP	C	G	17.65%	21.05%	25.53%
P10	DCHS2	4	155226289	155226289	Silent	SNP	G	A	14.29%	30.00%	22.22%
P10	ADGRV1	5	90136783	90136783	Missense_Mutation	SNP	T	C	29.03%	32.35%	51.85%
P10	DCDC2	6	24302208	24302208	Missense_Mutation	SNP	G	C	15.09%	20.41%	15.49%
P10	DEFB110	6	49976914	49976914	Silent	SNP	A	G	17.46%	6.56%	16.42%
P10	TINAG	6	54214679	54214679	Silent	SNP	C	T	5.81%	16.25%	17.11%
P10	ENTPD8	9	140332620	140332620	Missense_Mutation	SNP	C	T	15.25%	7.41%	
P10	CPNE8	12	39154659	39154659	Nonsense_Mutation	SNP	G	A	28.12%	26.67%	38.46%
P10	LGR5	12	71928958	71928958	Silent	SNP	T	A	18.92%	44.12%	35.14%
P10	CNBD2	20	34571992	34571992	Missense_Mutation	SNP	G	T	8.82%	2.82%	
P10	GBP5	1	89734432	89734432	Missense_Mutation	SNP	C	A	16.33%		7.84%
P10	CEP170	1	243349683	243349683	Missense_Mutation	SNP	C	T	6.48%		7.50%
P10	RANBP2	2	109367754	109367754	Silent	SNP	C	T			5.31%
P10	SCN1A	2	166847969	166847969	Missense_Mutation	SNP	G	A	15.09%		7.41%
P10	ERBB3	12	56489560	56489560	Missense_Mutation	SNP	G	T	5.92%		3.18%
P10	PLBD2	12	113796517	113796517	Missense_Mutation	SNP	C	A	7.45%		21.74%
P10	CDH11	16	65022247	65022247	Missense_Mutation	SNP	C	A	24.49%		20.37%
P10	ZFYVE9	1	52704023	52704023	Missense_Mutation	SNP	C	T	15.09%		2.00%
P10	GON4L	1	155785613	155785613	Missense_Mutation	SNP	C	A	12.70%		4.08%
P10	COL6A3	2	238280649	238280649	Silent	SNP	C	T	15.00%		11.11%
P10	CDKAL1	6	20955811	20955811	Missense_Mutation	SNP	C	G	16.67%		4.76%
P10	CSGALNACT1	8	19316006	19316006	Missense_Mutation	SNP	G	C	12.50%		13.58%
P10	POTEH	22	16267033	16267033	Silent	SNP	G	A	5.26%		2.73%
P10	PLB1	2	28849309	28849309	Missense_Mutation	SNP	G	T	13.46%		
P10	SLC8A1	2	40655690	40655690	Silent	SNP	G	A	5.11%		
P10	PLCH1	3	155199067	155199067	Missense_Mutation	SNP	C	A	17.78%		
P10	TMEM242	6	157739853	157739853	Silent	SNP	C	T	7.69%		
P10	GSN	9	124089678	124089678	Silent	SNP	C	T	6.06%		
P10	FBH1	10	5963485	5963485	Missense_Mutation	SNP	G	A	9.09%		
P10	MAPT	17	44096073	44096073	Missense_Mutation	SNP	G	A	24.32%		
P10	ICAM5	19	10403356	10403356	Missense_Mutation	SNP	T	C	13.11%		
P10	HIRA	22	19338914	19338914	Nonsense_Mutation	SNP	G	T	5.83%		
P11	FHAD1	1	15708543	15708543	Nonsense_Mutation	SNP	G	T	31.40%	24.86%	39.72%
P11	MEAF6	1	37980288	37980288	Silent	SNP	C	A	17.50%	10.87%	20.00%
P11	MACF1	1	39913235	39913235	Silent	SNP	C	T	6.06%	9.84%	2.88%
P11	CAP1	1	40535522	40535522	Silent	SNP	A	G	26.11%	23.35%	19.92%
P11	ORC1	1	52859466	52859466	Missense_Mutation	SNP	T	A	12.50%	20.99%	28.30%
P11	CACHD1	1	65068563	65068563	Missense_Mutation	SNP	G	T	31.00%	34.11%	40.58%
P11	ST6GALNAC3	1	77094421	77094421	Missense_Mutation	SNP	A	C	10.96%	1.90%	
P11	TRIM46	1	155150627	155150627	Missense_Mutation	SNP	C	A	4.08%		
P11	CD1E	1	158325164	158325164	Missense_Mutation	SNP	G	T	7.00%		
P11	ASPM	1	197074315	197074315	Missense_Mutation	SNP	C	G	5.22%		
P11	MYT1L	2	1855417	1855417	Missense_Mutation	SNP	G	A	16.13%	13.21%	13.21%
P11	PUM2	2	20458009	20458009	Nonsense_Mutation	SNP	G	A	27.78%	23.21%	38.78%
P11	SRD5A2	2	31805825	31805825	Missense_Mutation	SNP	G	T	23.68%	29.03%	30.51%
P11	C2orf92	2	98315532	98315532	Missense_Mutation	SNP	C	A	8.43%	18.06%	38.71%
P11	NEUROD1	2	182542720	182542721	Missense_Mutation	DNP	AC	TT	26.23%	24.24%	37.50%
P11	ANKRD44	2	197854370	197854370	Missense_Mutation	SNP	G	A	6.67%	13.48%	13.00%
P11	DOCK10	2	225639665	225639665	Missense_Mutation	SNP	G	C	8.24%		
P11	KIF1A	2	241713660	241713660	Missense_Mutation	SNP	G	A	20.93%	17.74%	23.60%
P11	RARB	3	25502819	25502819	Missense_Mutation	SNP	G	T	11.86%	25.00%	33.33%
P11	MYRIP	3	40085616	40085628	In_Frame_Del	DEL	CTGCTGCATGCG	-	0.98%	5.38%	
P11	RNF123	3	49753864	49753864	Missense_Mutation	SNP	G	A	21.21%	19.44%	19.72%
P11	FLNB	3	58134387	58134387	Missense_Mutation	SNP	A	T	7.94%	14.29%	20.65%
P11	BOC	3	112992073	112992073	Silent	SNP	C	T	13.73%	20.51%	16.42%
P11	MFSD1	3	158526977	158526977	Silent	SNP	C	A	3.02%		
P11	NOA1	4	57843650	57843650	Silent	SNP	C	T	11.32%	10.74%	14.95%
P11	SEC24B	4	110446052	110446052	Missense_Mutation	SNP	G	T	10.59%	10.00%	16.36%
P11	FASTKD3	5	7868083	7868083	Silent	SNP	C	T	3.23%		
P11	CDH10	5	24487850	24487850	Missense_Mutation	SNP	A	T	8.47%		
P11	CLPS	6	35763547	35763547	Silent	SNP	G	A	5.43%	8.54%	9.21%
P11	TREM2	6	41129059	41129059	Missense_Mutation	SNP	C	A	20.93%	12.90%	14.93%
P11	MDN1	6	90437625	90437625	Missense_Mutation	SNP	C	T	11.39%	16.47%	24.77%
P11	CLVS2	6	123332153	123332153	Missense_Mutation	SNP	G	A	15.09%	9.38%	28.26%
P11	MAP3K5	6	136882769	136882769	Missense_Mutation	SNP	G	T	8.70%		
P11	THBS2	6	169629763	169629764	Missense_Mutation	DNP	GG	TT	20.00%	6.00%	5.88%
P11	OSBPL3	7	24874319	24874319	Missense_Mutation	SNP	C	A	35.19%	27.14%	33.33%
P11	CACNA2D1	7	81588658	81588658	Missense_Mutation	SNP	G	A	9.23%	10.91%	12.24%
P11	TEX47	7	88424177	88424177	Missense_Mutation	SNP	C	T	4.58%		
P11	ZNF804B	7	88965573	88965573	Missense_Mutation	SNP	G	C	32.39%	27.39%	39.70%
P11	MUC3A	7	100550398	100550398	Missense_Mutation	SNP	A	G	38.06%	47.09%	61.76%
P11	BLK	8	11412882	11412882	Missense_Mutation	SNP	G	C	50.00%	49.66%	57.02%
P11	TRPM6	9	77418710	77418710	Missense_Mutation	SNP	C	A	36.59%	47.76%	49.28%
P11	SPATA31C1	9	90535877	90535877	Missense_Mutation	SNP	C	A	31.68%	32.93%	44.62%
P11	S1PR3	9	91616875	91616875	Missense_Mutation	SNP	G	A	47.30%	40.91%	63.54%

P11	RNF208	9	140115021	140115021	Missense_Mutation	SNP	G	A	50.00%	48.94%	58.49%
P11	SVIL	10	29818733	29818733	Missense_Mutation	SNP	C	A	26.96%	36.42%	33.53%
P11	MBL2	10	54530468	54530468	Missense_Mutation	SNP	C	A	11.54%	8.62%	12.12%
P11	OR4S1	11	48328083	48328083	Missense_Mutation	SNP	C	A	10.26%	16.67%	19.27%
P11	TMX2	11	57507615	57507615	Silent	SNP	G	T	9.80%	7.69%	20.29%
P11	DYNC2H1	11	103026090	103026090	Missense_Mutation	SNP	G	T	34.71%	33.64%	32.03%
P11	PZP	12	9310399	9310399	Silent	SNP	A	T	15.52%	12.12%	14.86%
P11	INTS13	12	27081181	27081181	Missense_Mutation	SNP	C	G	7.77%	8.03%	15.53%
P11	MUC19	12	40835789	40835789	Missense_Mutation	SNP	C	A	18.00%	20.59%	9.88%
P11	CNTN1	12	41316098	41316098	Nonsense_Mutation	SNP	C	T	9.20%		
P11	ADAMTS20	12	43824267	43824267	Missense_Mutation	SNP	G	T	40.00%	43.43%	50.62%
P11	ADAMTS20	12	43824272	43824272	Nonsense_Mutation	SNP	G	T	44.09%	44.79%	50.72%
P11	FAM186B	12	49997142	49997142	Missense_Mutation	SNP	G	C	13.21%	2.67%	4.62%
P11	NAV3	12	78444617	78444617	Nonsense_Mutation	SNP	C	T	9.09%	8.16%	11.11%
P11	ANKS1B	12	100048906	100048906	Missense_Mutation	SNP	A	C	8.60%		
P11	GNPTAB	12	102158064	102158064	Silent	SNP	A	T	29.03%	34.10%	27.12%
P11	TBX3	12	115120672	115120672	Missense_Mutation	SNP	G	A	10.11%	15.49%	16.23%
P11	MTUS2	13	29855946	29855946	Missense_Mutation	SNP	T	C	4.69%		
P11	FREM2	13	39448637	39448637	Missense_Mutation	SNP	G	T	30.00%	39.84%	48.12%
P11	ALG11	13	52598972	52598972	Missense_Mutation	SNP	A	G	35.00%	36.36%	40.43%
P11	AKAP6	14	33014853	33014853	Missense_Mutation	SNP	G	C	9.09%	11.39%	15.38%
P11	TEDC1	14	105965135	105965135	Missense_Mutation	SNP	G	C	15.00%	13.16%	27.59%
P11	CYFIP1	15	22955270	22955270	Missense_Mutation	SNP	C	T	16.67%	23.76%	22.68%
P11	SH3GL3	15	84245359	84245359	Missense_Mutation	SNP	C	A	8.11%	10.91%	10.91%
P11	ZNF200	16	3274407	3274407	Missense_Mutation	SNP	C	A	10.71%	7.77%	12.73%
P11	CCP110	16	19547320	19547320	Missense_Mutation	SNP	C	G	7.69%	16.22%	6.93%
P11	PHKB	16	47614219	47614219	Missense_Mutation	SNP	G	A	8.05%	14.53%	8.59%
P11	NPIPB15	16	74415479	74415479	Missense_Mutation	SNP	C	G	8.33%	2.91%	2.54%
P11	EFCAB5	17	28434960	28434960	Missense_Mutation	SNP	G	T	31.52%	33.33%	32.17%
P11	KLHL10	17	39994352	39994352	Silent	SNP	C	A	10.67%	10.24%	15.65%
P11	USP32	17	58260740	58260740	Silent	SNP	C	A	3.14%	12.93%	12.15%
P11	TACO1	17	61678634	61678634	Silent	SNP	C	A	15.09%	1.16%	0.86%
P11	OTOP3	17	72942904	72942904	Missense_Mutation	SNP	C	A	30.77%	30.06%	35.42%
P11	ST8SIA5	18	44260436	44260436	Missense_Mutation	SNP	G	T	10.00%	15.89%	11.81%
P11	MUC16	19	8966708	8966708	Silent	SNP	G	A	17.39%	21.05%	23.64%
P11	KLK1	19	51323643	51323643	Missense_Mutation	SNP	T	G	12.07%	23.46%	22.09%
P11	HCK	20	30681801	30681801	Missense_Mutation	SNP	G	C	6.25%	2.22%	
P11	AURKA	20	54958198	54958198	Missense_Mutation	SNP	G	A	9.68%	12.50%	11.97%
P11	ZNF831	20	57828084	57828084	Missense_Mutation	SNP	G	T	8.96%	14.43%	11.11%
P11	COL6A1	21	47418869	47418869	Missense_Mutation	SNP	A	C	7.50%		
P11	MPPED1	22	43821036	43821036	Silent	SNP	C	A	19.05%	26.58%	29.70%
P11	CT45A1	X	134856771	134856771	Silent	SNP	G	A	3.66%	6.49%	6.60%
P11	SYF2	1	25558615	25558615	Missense_Mutation	SNP	G	A	10.22%	15.67%	22.04%
P11	CNKSR1	1	26514800	26514800	Splice_Site	SNP	T	C	12.50%	40.54%	42.11%
P11	NUDC	1	27269463	27269463	Silent	SNP	G	T	18.75%	21.05%	14.78%
P11	CSMD2	1	34383716	34383716	Missense_Mutation	SNP	C	A	13.68%	28.57%	39.29%
P11	MPL	1	43804221	43804221	Missense_Mutation	SNP	C	A	21.74%	28.18%	18.04%
P11	LRRC7	1	70555434	70555434	Missense_Mutation	SNP	C	A	34.85%	40.91%	47.66%
P11	COL11A1	1	103363701	103363701	Missense_Mutation	SNP	C	T	25.81%	34.67%	39.18%
P11	DDX1	2	15763596	15763596	Missense_Mutation	SNP	A	T	14.61%	29.20%	34.65%
P11	DYSF	2	71795346	71795346	Silent	SNP	A	T	22.22%	31.01%	32.85%
P11	TTN	2	179648828	179648828	Missense_Mutation	SNP	C	A	20.93%	32.79%	34.04%
P11	FSIP2	2	186667587	186667587	Silent	SNP	C	A	21.85%	33.13%	33.33%
P11	AOX1	2	201531449	201531449	Missense_Mutation	SNP	A	G	45.28%	34.07%	30.67%
P11	FZD7	2	202901020	202901020	Silent	SNP	G	T	25.00%	29.51%	48.72%
P11	GIGYF2	2	233660894	233660894	Missense_Mutation	SNP	G	T	17.20%	31.75%	36.51%
P11	ADGRG7	3	100365450	100365450	Missense_Mutation	SNP	G	A	32.58%	46.67%	37.30%
P11	PIK3CB	3	138382803	138382803	Missense_Mutation	SNP	A	C	24.05%	23.57%	27.54%
P11	PPARGC1A	4	23825902	23825902	Splice_Site	SNP	C	A	10.81%	35.79%	29.03%
P11	DTHD1	4	36345325	36345325	Missense_Mutation	SNP	G	C	26.88%	40.17%	36.64%
P11	SULT1B1	4	70596226	70596226	Missense_Mutation	SNP	C	A	31.94%	46.27%	36.96%
P11	GRIA2	4	158284065	158284065	Missense_Mutation	SNP	G	A	21.77%	24.16%	37.50%
P11	FYB1	5	39202754	39202754	Silent	SNP	G	A	18.00%	24.87%	30.04%
P11	AGGF1	5	76348621	76348621	Missense_Mutation	SNP	C	G	8.33%	28.18%	30.70%
P11	PCDHA2	5	140176363	140176363	Missense_Mutation	SNP	C	A	21.74%	41.77%	55.41%
P11	PCDHB5	5	140516805	140516805	Nonsense_Mutation	SNP	C	T	11.11%	14.29%	31.58%
P11	PCDHGA6	5	140753711	140753711	Missense_Mutation	SNP	G	T	29.21%	37.78%	52.94%
P11	KIF4B	5	154395168	154395168	Missense_Mutation	SNP	G	C	13.87%	27.27%	20.97%
P11	TRIM7	5	180622431	180622431	Missense_Mutation	SNP	G	T	36.36%	41.98%	44.00%
P11	PRDM1	6	106554877	106554877	Missense_Mutation	SNP	C	A	14.04%	22.84%	40.28%
P11	C7orf65	7	47698526	47698526	Missense_Mutation	SNP	A	T	28.24%	35.90%	45.37%
P11	CSMD1	8	3565947	3565947	Missense_Mutation	SNP	T	A	17.17%	5.61%	
P11	ADAM2	8	39602384	39602384	Missense_Mutation	SNP	C	A	19.82%	38.52%	44.05%
P11	FER1L6	8	125035828	125035828	Missense_Mutation	SNP	T	A	30.43%	31.13%	38.76%
P11	FXYD4	10	43869213	43869213	Missense_Mutation	SNP	G	T	13.04%	21.05%	6.00%
P11	FAM170B	10	50339705	50339705	Silent	SNP	G	T	21.43%	39.47%	46.15%
P11	BICC1	10	60566374	60566374	Missense_Mutation	SNP	G	T	19.70%	32.00%	44.05%
P11	DNTT	10	98087326	98087326	Missense_Mutation	SNP	G	A	18.52%	27.35%	37.58%
P11	NLRP10	11	7982719	7982719	Missense_Mutation	SNP	A	T	31.94%	32.69%	53.01%

P11	LGR4		11	27402211	27402212	Missense_Mutation	DNP	TG	AA	25.81%	35.24%	40.68%
P11	ARHGEF12		11	120345267	120345267	Splice_Site	SNP	A	T	18.95%	38.04%	33.62%
P11	TMEM132B		12	125834604	125834604	Missense_Mutation	SNP	C	T	3.64%	9.41%	10.75%
P11	ARL11		13	50204845	50204845	Missense_Mutation	SNP	C	A	13.22%	23.35%	18.12%
P11	FLRT2		14	86088906	86088906	Missense_Mutation	SNP	G	A	18.87%	26.76%	31.17%
P11	GJD2		15	35045113	35045113	Missense_Mutation	SNP	G	T	33.33%	44.33%	55.65%
P11	CSPG4		15	75979829	75979829	Missense_Mutation	SNP	G	T	28.33%	41.94%	48.25%
P11	ANPEP		15	90347522	90347522	Silent	SNP	G	T	20.69%	10.00%	19.05%
P11	ZNF263		16	3339631	3339631	Missense_Mutation	SNP	G	T	25.00%	31.00%	39.67%
P11	ARMC5		16	31471108	31471108	Missense_Mutation	SNP	G	C	10.00%	21.43%	18.00%
P11	NOD2		16	50733785	50733785	Missense_Mutation	SNP	G	A	13.27%	7.96%	4.80%
P11	HS3ST3A1		17	13399887	13399887	Missense_Mutation	SNP	C	G	23.48%	26.22%	29.49%
P11	SDK2		17	71398110	71398110	Silent	SNP	C	A	23.61%	30.97%	28.00%
P11	CEP192		18	13099541	13099541	Missense_Mutation	SNP	G	T	31.08%	36.27%	24.78%
P11	LOXHD1		18	44172535	44172535	Missense_Mutation	SNP	C	A	29.23%	36.78%	41.41%
P11	SYT3		19	51128785	51128785	Missense_Mutation	SNP	C	A	10.34%	16.33%	34.04%
P11	KLK15		19	51330228	51330228	Silent	SNP	C	G	11.76%	17.65%	25.71%
P11	FPR1		19	52249819	52249819	Missense_Mutation	SNP	C	G	18.99%	26.32%	23.31%
P11	SUN5		20	31573650	31573650	Silent	SNP	G	A	36.07%	44.30%	34.48%
P11	SALL4		20	50408541	50408541	Missense_Mutation	SNP	G	T	6.59%	18.71%	14.21%
P11	TTLL12		22	43568504	43568504	Silent	SNP	C	T	17.81%	30.91%	29.66%
P11	TEX13A	X		104464772	104464772	Missense_Mutation	SNP	C	A	29.27%	48.44%	62.71%
P11	CT45A9	X		134947909	134947909	Missense_Mutation	SNP	C	T	41.18%	54.84%	69.23%
P11	MACF1		1	39789975	39789975	Silent	SNP	C	T	7.64%	15.38%	21.31%
P11	ST6GALNAC3		1	76877926	76877926	Silent	SNP	T	C	9.76%	20.87%	24.24%
P11	KCND3		1	112329591	112329591	Missense_Mutation	SNP	C	A	28.57%	27.78%	47.76%
P11	TTC24		1	156552174	156552174	Silent	SNP	C	T	8.05%	10.46%	17.49%
P11	OR2T8		1	248084856	248084856	Silent	SNP	C	A	0.65%	3.59%	3.54%
P11	PSD4		2	113940088	113940088	Silent	SNP	C	T	31.82%	30.23%	34.75%
P11	CLASP1		2	122204994	122204994	Nonsense_Mutation	SNP	T	A	10.34%	19.49%	21.60%
P11	PLA2R1		2	160889600	160889600	Silent	SNP	G	A	27.36%	23.14%	41.54%
P11	AGPS		2	178301743	178301743	Nonsense_Mutation	SNP	G	T	13.67%	11.76%	19.74%
P11	COL3A1		2	189864048	189864048	Missense_Mutation	SNP	G	T	9.80%	12.59%	18.72%
P11	ALS2		2	202617934	202617934	Missense_Mutation	SNP	C	A	16.00%	20.00%	40.00%
P11	TMEM43		3	14174435	14174435	Missense_Mutation	SNP	G	T	7.89%	28.57%	26.67%
P11	PP2D1		3	20043389	20043389	Missense_Mutation	SNP	G	T	15.00%	15.71%	28.67%
P11	PHF7		3	52455733	52455733	Nonsense_Mutation	SNP	C	T	14.12%	14.29%	28.30%
P11	RFT1		3	53140836	53140836	Missense_Mutation	SNP	C	A	17.76%	21.67%	27.91%
P11	ADCY5		3	123021902	123021902	Missense_Mutation	SNP	C	A	14.61%	21.60%	22.36%
P11	MANBA		4	103595178	103595178	Missense_Mutation	SNP	G	A	24.69%	23.08%	34.65%
P11	DHX29		5	54558757	54558757	Missense_Mutation	SNP	C	A	40.54%	38.36%	54.43%
P11	PCDHGB4		5	140767737	140767737	Missense_Mutation	SNP	T	C	25.81%	38.16%	50.59%
P11	RNF145		5	158603789	158603789	Missense_Mutation	SNP	C	T	11.93%	15.38%	17.93%
P11	RASGEF1C		5	179565033	179565033	Missense_Mutation	SNP	G	T	45.71%	25.00%	63.24%
P11	TRERF1		6	42227093	42227093	Silent	SNP	C	A	14.29%	15.32%	31.16%
P11	TTBK1		6	43214416	43214416	Silent	SNP	C	A	39.13%	51.06%	53.41%
P11	CENPQ		6	49456396	49456397	Missense_Mutation	DNP	AG	GT	12.90%	18.33%	17.92%
P11	KHDRBS2		6	62604579	62604579	Missense_Mutation	SNP	C	A	14.05%	13.73%	22.73%
P11	MED23		6	131914181	131914181	Missense_Mutation	SNP	T	A	9.52%	23.60%	35.78%
P11	ESR1		6	152332844	152332844	Missense_Mutation	SNP	C	A	37.18%	46.23%	52.63%
P11	SMOC2		6	168999669	168999669	Missense_Mutation	SNP	C	A	7.14%	14.37%	15.90%
P11	PCLO		7	82544695	82544695	Missense_Mutation	SNP	A	C	12.40%	9.52%	19.88%
P11	SEMA3A		7	83591095	83591095	Silent	SNP	T	A	9.94%	14.06%	22.67%
P11	CHD7		8	61763826	61763826	Missense_Mutation	SNP	T	A	13.33%	15.32%	33.96%
P11	CER1		9	14722647	14722647	Silent	SNP	C	T	14.41%	12.93%	21.53%
P11	MALRD1		10	19620375	19620375	Silent	SNP	C	T	10.34%	12.75%	24.06%
P11	PTCHD3		10	27702633	27702633	Missense_Mutation	SNP	G	T	34.21%	39.73%	48.61%
P11	ANTXRL		10	47701247	47701247	Missense_Mutation	SNP	C	G	8.06%	25.74%	31.43%
P11	SAA2		11	18267468	18267468	Silent	SNP	T	C	12.68%	13.91%	27.98%
P11	OR5AS1		11	55798843	55798843	Missense_Mutation	SNP	A	C	28.81%	26.73%	31.37%
P11	OR5AN1		11	59132782	59132782	Missense_Mutation	SNP	C	T	16.42%	29.47%	35.96%
P11	MS4A14		11	60183410	60183410	Missense_Mutation	SNP	A	T	11.34%	14.49%	30.46%
P11	SLCO2B1		11	74914466	74914466	Missense_Mutation	SNP	G	T	20.69%	15.33%	23.53%
P11	TRIM64B		11	89607224	89607224	Missense_Mutation	SNP	A	G	4.35%	9.24%	11.23%
P11	DDX10		11	108594031	108594031	Missense_Mutation	SNP	C	G	11.98%	14.37%	23.38%
P11	OR10G4		11	123887051	123887052	Frame_Shift_Del	DEL	T	-	10.38%	13.17%	20.59%
P11	PRR4		12	11002019	11002019	Silent	SNP	G	A	6.90%	13.51%	17.65%
P11	GALNT6		12	51771132	51771132	Silent	SNP	G	T	11.67%	15.12%	32.29%
P11	TMEM132B		12	126138170	126138170	Silent	SNP	T	A	10.47%	14.91%	26.49%
P11	MDGA2		14	47389232	47389232	Silent	SNP	G	T	18.92%	11.94%	27.66%
P11	SIPA1L1		14	72190392	72190392	Missense_Mutation	SNP	C	T	14.05%	12.15%	17.52%
P11	USP31		16	23079562	23079562	Silent	SNP	C	G	18.92%	10.98%	28.66%
P11	MYH4		17	10352340	10352340	Silent	SNP	C	T	10.00%	33.80%	35.80%
P11	MYO15A		17	18042224	18042224	Missense_Mutation	SNP	C	G	21.84%	25.23%	27.69%
P11	DBF4B		17	42824508	42824508	Missense_Mutation	SNP	G	T	23.26%	35.00%	45.00%
P11	KIF2B		17	51900994	51900994	Missense_Mutation	SNP	C	A	20.93%	26.53%	38.60%
P11	HEATR6		17	58147197	58147197	Missense_Mutation	SNP	G	C	4.46%	4.90%	7.69%
P11	SMCHD1		18	2752538	2752538	Missense_Mutation	SNP	G	T	17.58%	18.45%	30.71%
P11	CEP192		18	13059080	13059081	Frame_Shift_Del	DEL	G	-	14.63%	18.46%	10.67%

P11	CEP192	18	13059081	13059081	Missense_Mutation	SNP	G	T	15.00%	19.05%	10.39%
P11	RIT2	18	40695413	40695413	Silent	SNP	T	A	29.27%	32.69%	44.12%
P11	MBD1	18	47800664	47800665	Frame_Shift_Del	DEL	C	-	21.19%	11.68%	26.23%
P11	CHAF1A	19	4429579	4429579	Silent	SNP	G	T	11.76%	19.71%	23.29%
P11	SLC32A1	20	37356814	37356814	Silent	SNP	C	A	6.49%	10.53%	26.17%
P11	MXRA5	X	3229573	3229573	Missense_Mutation	SNP	C	A	26.79%	23.81%	40.24%
P11	CACHD1	1	65113500	65113500	Missense_Mutation	SNP	G	C		6.82%	
P11	APOB	2	21229784	21229784	Missense_Mutation	SNP	T	C		7.38%	7.52%
P11	GPN1	2	27851931	27851931	Silent	SNP	G	C		3.31%	3.23%
P11	ZAP70	2	98349465	98349465	Missense_Mutation	SNP	A	C		8.24%	
P11	ASIC4	2	220400003	220400003	Missense_Mutation	SNP	C	T		9.47%	
P11	PSMD1	2	231925981	231925981	Missense_Mutation	SNP	C	A		5.36%	
P11	TOMM70	3	100105119	100105119	Missense_Mutation	SNP	G	C		3.80%	
P11	ZBBX	3	167035282	167035282	Missense_Mutation	SNP	T	C		5.26%	
P11	AHSG	3	186338689	186338689	Nonsense_Mutation	SNP	T	A		8.91%	
P11	ATP13A4	3	193188693	193188693	Missense_Mutation	SNP	C	T		3.23%	
P11	USP17L22	4	9270321	9270321	Missense_Mutation	SNP	C	A		1.30%	1.04%
P11	ELF2	4	139980348	139980348	Missense_Mutation	SNP	C	G		4.48%	
P11	ETFDH	4	159620267	159620267	Missense_Mutation	SNP	T	G		5.26%	
P11	TULP4	6	158900939	158900939	Missense_Mutation	SNP	G	A		2.79%	
P11	NOL6	9	33466956	33466956	Missense_Mutation	SNP	T	C		8.08%	3.03%
P11	ZBTB39	12	57398125	57398125	Missense_Mutation	SNP	C	T		2.72%	
P11	ALKBH2	12	109530418	109530418	Silent	SNP	C	T		4.14%	0.47%
P11	LPAR6	13	48985569	48985569	Missense_Mutation	SNP	G	C		4.32%	
P11	COL4A1	13	110857735	110857735	Missense_Mutation	SNP	C	A		18.18%	8.57%
P11	MGA	15	42042132	42042135	In_Frame_Del	DEL	TGG	-		6.72%	0.86%
P11	IRX6	16	55360465	55360465	Missense_Mutation	SNP	T	A		4.23%	
P11	MAF	16	79632788	79632788	Missense_Mutation	SNP	C	G		8.64%	
P11	MYH9	22	36688150	36688150	In_Frame_Ins	INS	-	CGTAGG		5.97%	
P11	PINK1	1	20975018	20975018	Missense_Mutation	SNP	A	C		3.75%	9.64%
P11	SSRP1	11	57095813	57095813	Silent	SNP	G	T		2.20%	13.01%
P11	C1orf54	1	150248209	150248209	Splice_Site	SNP	G	T			6.98%
P11	BIRC6	2	32667236	32667236	Missense_Mutation	SNP	A	T			2.56%
P11	PHC3	3	169824626	169824626	Missense_Mutation	SNP	G	T			3.51%
P11	PPARGC1A	4	23831123	23831123	Missense_Mutation	SNP	T	A			5.56%
P11	SCD5	4	83552568	83552568	Missense_Mutation	SNP	C	G			3.79%
P11	NPY5R	4	164271619	164271619	Missense_Mutation	SNP	C	A			4.70%
P11	CUL9	6	43174210	43174210	Missense_Mutation	SNP	G	A			4.79%
P11	RELN	7	103293094	103293094	Missense_Mutation	SNP	T	C			4.11%
P11	HHLA1	8	133090134	133090134	Missense_Mutation	SNP	G	A			5.42%
P11	KCNT1	9	138670651	138670651	Silent	SNP	C	T			14.52%
P11	MMP26	11	5012670	5012670	Missense_Mutation	SNP	G	A			8.97%
P11	OR10A2	11	6891478	6891478	Missense_Mutation	SNP	T	G			4.70%
P11	NCOR1	17	15978995	15978995	Missense_Mutation	SNP	T	C			4.97%
P11	SKOR2	18	44775448	44775448	Missense_Mutation	SNP	A	T			9.38%
P11	EPOR	19	11492727	11492727	Silent	SNP	C	T			5.66%
P11	PHKA1	X	71932701	71932701	Missense_Mutation	SNP	C	G			12.00%
P12	CRIM1	2	36669820	36669820	Missense_Mutation	SNP	G	T	22.86%		45.83%
P12	TOPBP1	3	133327743	133327743	Missense_Mutation	SNP	G	A	8.75%		
P12	BLTP1	4	123192707	123192707	Silent	SNP	G	A	9.80%		16.67%
P12	UGT3A1	5	35965590	35965590	Missense_Mutation	SNP	C	A	15.00%	20.00%	53.19%
P12	MSH3	5	79968655	79968655	Silent	SNP	A	T	15.79%		
P12	GPR158	10	25887177	25887177	Silent	SNP	G	A	7.41%		
P12	ATAD1	10	89514489	89514489	Silent	SNP	C	T	11.11%		
P12	CTSZ	20	57571730	57571730	Silent	SNP	C	T	12.00%		
P12	PDE4DIP	1	145021126	145021126	Missense_Mutation	SNP	G	A	10.71%	22.22%	5.58%
P12	WDR97	8	145164472	145164472	Missense_Mutation	SNP	G	T	43.24%	26.67%	19.15%
P12	BTBD7	14	93712590	93712590	Missense_Mutation	SNP	T	A	16.44%	8.33%	25.00%
P12	MED13	17	60059844	60059844	Missense_Mutation	SNP	C	T	2.38%		8.45%
P12	TTC3	21	38510969	38510969	Silent	SNP	C	T	31.03%	10.00%	25.35%
P12	SFPQ	1	35658469	35658469	Missense_Mutation	SNP	G	A		9.09%	
P12	INPP5B	1	38332184	38332184	Missense_Mutation	SNP	G	A		24.32%	1.74%
P12	GRM1	6	146480697	146480697	Missense_Mutation	SNP	G	T		15.69%	
P12	EIF3E	8	109247264	109247264	Nonsense_Mutation	SNP	G	T		25.81%	
P12	FUT11	10	75532375	75532375	Missense_Mutation	SNP	G	A		13.04%	
P12	STN1	10	105648836	105648836	Missense_Mutation	SNP	G	A		19.35%	2.27%
P12	TTC17	11	43419530	43419530	Missense_Mutation	SNP	G	A		10.53%	
P12	ZSWIM7	17	15902876	15902876	Silent	SNP	C	T		10.17%	
P12	BAIAP2	17	79031731	79031731	Missense_Mutation	SNP	G	A		16.22%	
P12	NECTIN2	19	45349818	45349818	Silent	SNP	G	A		10.34%	
P12	TASP1	20	13561608	13561608	Missense_Mutation	SNP	C	T		20.51%	1.15%
P12	ADORA3	1	112031602	112031602	Missense_Mutation	SNP	C	T			6.48%
P12	ANTXR1	2	69420546	69420546	Missense_Mutation	SNP	C	T			2.70%
P12	ABI3BP	3	100617687	100617687	Missense_Mutation	SNP	G	A			8.33%
P12	USP13	3	179481955	179481955	Missense_Mutation	SNP	C	T			1.40%
P12	ZPBP	7	50129273	50129273	Missense_Mutation	SNP	G	A			10.96%
P12	GRIA4	11	105769104	105769104	Nonsense_Mutation	SNP	G	A			9.09%
P12	TMEM45B	11	129725712	129725712	Silent	SNP	G	A			10.53%
P12	SACS	13	23911433	23911433	Silent	SNP	C	T			14.81%

P12	SIPA1L1	14	72054667	72054667	Silent	SNP	C	T			7.32%
P12	KAT7	17	47893192	47893192	Missense_Mutation	SNP	C	T			8.45%
P12	MBD1	18	47803517	47803517	Silent	SNP	C	T			7.06%
P12	LMTK3	19	49003145	49003145	Missense_Mutation	SNP	G	A			9.23%
P13	EPHA2	1	16464590	16464590	Missense_Mutation	SNP	C	T		5.94%	
P13	FAM189B	1	155224497	155224497	Missense_Mutation	SNP	C	T		9.52%	
P13	TBX19	1	168262467	168262467	Missense_Mutation	SNP	C	T	4.57%	6.71%	
P13	PCNX2	1	233394109	233394109	Missense_Mutation	SNP	G	T		7.89%	
P13	MSH2	2	47702410	47702410	Splice_Site	SNP	G	C		5.56%	
P13	MOGS	2	74692175	74692175	Missense_Mutation	SNP	C	T		6.52%	
P13	SCN9A	2	167060528	167060528	Missense_Mutation	SNP	G	T		6.67%	
P13	CTNNB1	3	41266113	41266113	Missense_Mutation	SNP	C	T	43.64%	39.77%	22.22%
P13	FRAS1	4	79350274	79350274	Silent	SNP	G	A	0.75%	8.24%	1.99%
P13	UTRN	6	144814553	144814553	Silent	SNP	G	T		8.43%	
P13	TRPM6	9	77436688	77436688	Missense_Mutation	SNP	C	T	5.00%	12.50%	
P13	OLFM1	9	137982177	137982177	Missense_Mutation	SNP	A	G		13.64%	
P13	PSMA1	11	14535166	14535166	Missense_Mutation	SNP	G	A	2.27%	8.09%	
P13	KRT18	12	53344140	53344140	Missense_Mutation	SNP	G	A	3.20%	4.26%	
P13	BAZ2A	12	56993081	56993081	Missense_Mutation	SNP	C	A		6.19%	
P13	NACA	12	57106577	57106577	Missense_Mutation	SNP	G	A		6.82%	
P13	DUOXA1	15	45409813	45409813	Missense_Mutation	SNP	T	C	4.86%	11.98%	
P13	HERC1	15	63922808	63922808	Missense_Mutation	SNP	G	A		7.41%	
P13	PLCG2	16	81973580	81973580	Missense_Mutation	SNP	C	T		10.00%	
P13	RTN4RL1	17	1841032	1841032	Silent	SNP	G	A		13.33%	
P13	AURKB	17	8108230	8108230	Missense_Mutation	SNP	G	A		8.33%	
P13	PRPSAP1	17	74324820	74324821	Frame_Shift_Del	DEL	C	-		5.94%	
P13	NAPA	19	48018142	48018142	Missense_Mutation	SNP	C	T		4.29%	
P13	TTC28	22	28378320	28378320	Silent	SNP	C	T		7.32%	
P13	FAM118A	22	45728575	45728575	Nonsense_Mutation	SNP	C	A		10.17%	
P13	PLPP3	1	56977808	56977808	Missense_Mutation	SNP	C	T	7.89%	2.44%	
P13	RORC	1	151785768	151785768	Missense_Mutation	SNP	C	T	11.54%	4.00%	
P13	FCGR2A	1	161483723	161483723	Splice_Site	SNP	G	A	14.08%	5.71%	7.04%
P13	PPIC	5	122359609	122359609	Silent	SNP	G	A	7.48%	1.74%	
P13	RSF1	11	77389140	77389140	Missense_Mutation	SNP	C	T	9.09%	1.15%	
P13	DRAM1	12	102313901	102313901	Silent	SNP	C	T	9.23%	1.92%	
P13	PNN	14	39645335	39645335	Missense_Mutation	SNP	G	A	4.96%	1.59%	
P13	ABCC1	16	16110422	16110422	Missense_Mutation	SNP	G	A	7.59%	2.33%	
P13	RINL	19	39361361	39361361	Silent	SNP	A	T	23.08%	44.44%	15.79%
P13	PREX1	20	47295933	47295933	Silent	SNP	G	A	12.24%	4.76%	
P13	EIF4E1B	5	176072415	176072415	Missense_Mutation	SNP	G	A		2.70%	9.38%
P13	ANO6	12	45833519	45833519	Missense_Mutation	SNP	G	A	3.30%	2.50%	5.26%
P13	MTHFR	1	11861417	11861417	Silent	SNP	G	A	9.09%		
P13	LIX1L	1	145498130	145498130	Missense_Mutation	SNP	C	T	9.68%		
P13	DHX9	1	182827280	182827280	Missense_Mutation	SNP	G	A	10.34%		
P13	EPRS1	1	220205789	220205789	Silent	SNP	G	T	9.76%		
P13	STRN	2	37152329	37152329	Missense_Mutation	SNP	C	T	5.83%		
P13	C2orf68	2	85839054	85839054	Silent	SNP	C	T	5.63%		
P13	MAP4K4	2	102490655	102490655	Missense_Mutation	SNP	C	T	10.71%		
P13	ARHGAP15	2	144381776	144381776	Missense_Mutation	SNP	C	T	7.50%		
P13	AGPS	2	178326699	178326699	Missense_Mutation	SNP	C	T	9.68%		
P13	MKRN2	3	12623664	12623664	Missense_Mutation	SNP	G	A	8.22%		
P13	CELSR3	3	48698643	48698643	Silent	SNP	C	T	4.62%		
P13	IMPDH2	3	49065156	49065156	Missense_Mutation	SNP	C	T	13.95%		
P13	DNAH1	3	52404649	52404649	Nonsense_Mutation	SNP	C	T	8.82%		
P13	NFKBIZ	3	101575927	101575927	Missense_Mutation	SNP	G	A	6.86%		
P13	SEC62	3	169684616	169684616	Silent	SNP	G	A	5.94%		
P13	YEATS2	3	183508631	183508631	Missense_Mutation	SNP	C	T	7.69%		
P13	SENP2	3	185327065	185327065	Silent	SNP	C	A	11.11%		3.03%
P13	TNPO1	5	72178913	72178913	Missense_Mutation	SNP	C	T	7.41%		
P13	ARAP3	5	141034944	141034944	Silent	SNP	G	A	5.36%		
P13	GALNT10	5	153674474	153674474	Silent	SNP	C	T	12.68%		
P13	HNRNPH1	5	179045146	179045146	Missense_Mutation	SNP	C	T	11.11%		
P13	RPP21	6	30314541	30314541	Missense_Mutation	SNP	G	A	9.84%		
P13	VARS2	6	30884696	30884696	Missense_Mutation	SNP	C	T	12.50%		
P13	BAG6	6	31608043	31608043	Missense_Mutation	SNP	C	T	11.11%		
P13	AIG1	6	143382186	143382186	Silent	SNP	C	T	5.96%		
P13	OPRM1	6	154412501	154412501	Missense_Mutation	SNP	G	A	17.65%		
P13	ARID1B	6	157222604	157222604	Missense_Mutation	SNP	C	T	10.34%		
P13	IGF2R	6	160511040	160511040	Missense_Mutation	SNP	C	T	13.95%		
P13	AP5Z1	7	4825264	4825264	Missense_Mutation	SNP	C	T	4.62%		
P13	PMS2	7	6026708	6026708	Missense_Mutation	SNP	C	G	10.13%		
P13	GPNMB	7	23313763	23313763	Missense_Mutation	SNP	C	T	5.94%		
P13	LMTK2	7	97821513	97821513	Missense_Mutation	SNP	C	T	7.69%		
P13	POLR2J3	7	102212948	102212948	Silent	SNP	G	A	3.90%		
P13	SULF1	8	70541839	70541839	Missense_Mutation	SNP	G	A	8.22%		
P13	GNAQ	9	80343536	80343536	Nonsense_Mutation	SNP	G	C	8.00%		
P13	WDFY4	10	50155011	50155011	Silent	SNP	G	T	7.89%		
P13	IFIT1	10	91162592	91162592	Missense_Mutation	SNP	G	A	9.52%		
P13	COX15	10	101483814	101483814	Missense_Mutation	SNP	G	A	7.69%		

P13	KCNJ11		11	17409531	17409531	Silent	SNP	C	T	9.84%		
P13	TNKS1BP1		11	57069402	57069402	Missense_Mutation	SNP	C	T	7.69%		
P13	CDC42BPG		11	64597001	64597001	Silent	SNP	G	A	6.82%		
P13	CARNS1		11	67186982	67186982	Missense_Mutation	SNP	C	T	5.56%		
P13	MMP3		11	102710890	102710890	Missense_Mutation	SNP	G	A	12.24%		
P13	LIMA1		12	50589612	50589612	Splice_Site	SNP	C	T	10.19%		
P13	ZNF385A		12	54764469	54764469	Missense_Mutation	SNP	C	T	11.32%		
P13	ZC3H10		12	56514727	56514727	Silent	SNP	G	A	7.59%		
P13	ALDH1L2		12	105464484	105464484	Missense_Mutation	SNP	C	T	9.09%		
P13	ARPC3		12	110883315	110883315	Silent	SNP	G	A	8.45%		
P13	SPATA13		13	24858369	24858369	Missense_Mutation	SNP	G	A	7.89%		
P13	SYNE2		14	64537555	64537555	Missense_Mutation	SNP	C	T	8.70%		
P13	HSPA2		14	65008028	65008028	Missense_Mutation	SNP	C	T	7.23%		
P13	RPS6KL1		14	75388032	75388032	Silent	SNP	C	T	13.95%		
P13	ANKS3		16	4748894	4748894	Silent	SNP	G	A	8.82%		
P13	KIFC3		16	57832085	57832085	Missense_Mutation	SNP	C	T	7.62%		
P13	DERL2		17	5389436	5389436	Missense_Mutation	SNP	C	T	9.38%		5.71%
P13	MAPK7		17	19282274	19282274	Missense_Mutation	SNP	G	A	4.41%		2.13%
P13	RNF112		17	19316685	19316685	Missense_Mutation	SNP	G	A	14.63%		
P13	HSD17B1		17	40706745	40706745	Missense_Mutation	SNP	G	A	4.76%		
P13	MYOM1		18	3112381	3112381	Silent	SNP	G	A	10.00%		
P13	URI1		19	30502071	30502071	Missense_Mutation	SNP	G	A	8.57%		
P13	PPP1R13L		19	45885919	45885919	Missense_Mutation	SNP	C	T	8.45%		
P13	BFSP1		20	17475023	17475023	Missense_Mutation	SNP	C	T	16.46%		
P13	ACSS2		20	33507350	33507350	Missense_Mutation	SNP	C	T	2.40%		
P13	PKNOX1		21	44437037	44437037	Missense_Mutation	SNP	C	T	13.04%		
P13	SDF2L1		22	21997251	21997251	Silent	SNP	C	T	8.57%		
P13	XAGE3	X		52895531	52895531	Missense_Mutation	SNP	G	T	5.77%		
P13	TET2		4	106157275	106157275	Nonsense_Mutation	SNP	C	T	2.35%		4.08%
P13	ALDH7A1		5	125929067	125929067	Missense_Mutation	SNP	G	T	1.63%		2.02%
P13	TUT1		11	62346076	62346076	Missense_Mutation	SNP	G	A	2.56%		20.00%
P13	MYCBP		1	39333254	39333254	Missense_Mutation	SNP	C	A			24.32%
P13	USP33		1	78167130	78167130	Silent	SNP	G	A			14.04%
P13	TRIP12		2	230705552	230705552	Silent	SNP	G	A			10.00%
P13	SERPINB9		6	2892155	2892155	Missense_Mutation	SNP	A	T			11.54%
P13	FLNC		7	128498571	128498571	Silent	SNP	C	T			2.17%
P13	CCNE2		8	95897401	95897401	Missense_Mutation	SNP	G	T			26.67%
P13	KANK1		9	740857	740857	Missense_Mutation	SNP	G	A			4.62%
P13	EIF3A		10	120817546	120817546	Silent	SNP	C	T			12.96%
P13	SRGAP1		12	64491113	64491113	Missense_Mutation	SNP	C	A			14.29%
P13	SUPT6H		17	27010770	27010770	Missense_Mutation	SNP	A	C			10.71%
P13	C17orf64		17	58508590	58508590	Missense_Mutation	SNP	T	G			5.22%
P13	PIEZO2		18	10731483	10731483	Missense_Mutation	SNP	C	A			21.05%
P13	FTL		19	49469585	49469585	Silent	SNP	T	A			16.28%
P14	CSDE1		1	115273222	115273222	Silent	SNP	C	T	7.14%		
P14	VTCN1		1	117699342	117699342	Missense_Mutation	SNP	C	T	14.63%	1.02%	
P14	LCE2B		1	152659555	152659555	Missense_Mutation	SNP	G	A	17.24%	14.08%	9.76%
P14	EFR3B		2	25315984	25315984	Missense_Mutation	SNP	G	A	11.11%	17.39%	13.95%
P14	RUNX2		6	45399744	45399744	Missense_Mutation	SNP	C	T	18.42%	19.54%	17.02%
P14	DST		6	56346923	56346923	Silent	SNP	C	T	8.33%		
P14	EYS		6	65707494	65707494	Missense_Mutation	SNP	G	T	15.00%	10.00%	17.57%
P14	UTRN		6	144835170	144835170	Silent	SNP	G	A	13.21%	8.70%	4.44%
P14	PCLO		7	82785656	82785656	Missense_Mutation	SNP	C	T	6.32%		
P14	PTGS1		9	125141086	125141086	Missense_Mutation	SNP	T	C	25.71%	1.43%	25.00%
P14	OGA		10	103559017	103559017	Missense_Mutation	SNP	G	T	7.14%		
P14	NLRP14		11	7059892	7059892	Silent	SNP	G	A	20.00%	14.10%	10.87%
P14	HECTD4		12	112691953	112691953	Missense_Mutation	SNP	G	T	15.00%		
P14	MIER2		19	327172	327172	Missense_Mutation	SNP	A	T	15.79%		
P14	MRPL39		21	26976196	26976196	Missense_Mutation	SNP	T	A	19.44%	10.00%	12.50%
P14	ELOVL4		6	80634740	80634740	Nonsense_Mutation	SNP	C	A	20.83%	10.53%	14.89%
P14	PAX6		11	31811541	31811541	Missense_Mutation	SNP	G	T	14.29%	10.59%	6.67%
P14	MUC19		12	40858978	40858978	Silent	SNP	C	A	51.02%	42.53%	54.69%
P14	C8A		1	57349285	57349285	Silent	SNP	G	A	43.33%	41.89%	40.54%
P14	SOX13		1	204093908	204093908	Silent	SNP	C	G	25.00%	29.38%	6.67%
P14	COL12A1		6	75855946	75855947	Missense_Mutation	DNP	CT	AA	57.89%	43.53%	30.61%
P14	C8orf49		8	11619211	11619211	Missense_Mutation	SNP	C	A	50.88%	52.53%	38.67%
P14	NEFM		8	24776021	24776021	Missense_Mutation	SNP	G	A	6.45%	13.85%	14.29%
P14	PSG1		19	43372352	43372353	Frame_Shift_Del	DEL	G	-	65.12%	56.04%	54.72%
P14	NLGN4X	X		5811542	5811542	Missense_Mutation	SNP	G	T	50.00%	59.49%	72.50%
P14	NUP210		3	13407550	13407550	Missense_Mutation	SNP	C	T			8.54%
P14	LRRC58		3	120054736	120054736	Missense_Mutation	SNP	G	T			10.00%
P14	PRDM8		4	81123072	81123072	Silent	SNP	G	A			4.17%
P14	TNXB		6	32020463	32020463	Silent	SNP	G	A		11.95%	21.43%
P14	ARHGAP19		10	98989540	98989540	Missense_Mutation	SNP	C	T			8.33%
P14	KDM2B		12	122018770	122018770	Missense_Mutation	SNP	C	A			15.00%
P14	NUP58		13	25911107	25911107	Missense_Mutation	SNP	C	A			6.67%
P14	EIF2S1		14	67850104	67850104	Missense_Mutation	SNP	G	A			5.71%
P14	PAK5		20	9624884	9624884	Silent	SNP	C	T			8.82%
P14	RNF168		3	196214307	196214307	Missense_Mutation	SNP	C	T		3.85%	

P14	HLA-A	6	29912152	29912153	Missense_Mutation	DNP	CA	AG		4.92%	
P14	CENPJ	13	25486132	25486132	Missense_Mutation	SNP	C	T		15.58%	
P14	UNC79	14	93944021	93944021	Missense_Mutation	SNP	T	A		23.81%	
P15	OR10J3	1	159283829	159283829	Silent	SNP	G	T	21.95%	10.00%	23.61%
P15	PCNX2	1	233335965	233335965	Missense_Mutation	SNP	G	A		2.33%	
P15	PPP1R21	2	48734434	48734434	Missense_Mutation	SNP	C	A		5.71%	
P15	GOLGB1	3	121412872	121412872	Silent	SNP	C	T		2.75%	
P15	ANKRD17	4	73964154	73964154	Missense_Mutation	SNP	C	T		5.13%	
P15	KLHDC3	6	42985888	42985888	Missense_Mutation	SNP	C	A		4.17%	
P15	UBAP2	9	33988974	33988974	Missense_Mutation	SNP	C	T		3.39%	
P15	OR1S2	11	57971565	57971565	Missense_Mutation	SNP	A	T	12.50%	12.50%	36.54%
P15	LRRIQ1	12	85460581	85460581	Missense_Mutation	SNP	T	A	31.58%	24.49%	23.44%
P15	GOLGA8H	15	30902223	30902223	Missense_Mutation	SNP	G	C		4.90%	
P15	LRRC37A	17	44408875	44408876	Missense_Mutation	DNP	GA	TC	3.80%	3.87%	
P15	C1QTNF1	17	77043850	77043850	Missense_Mutation	SNP	G	T	11.11%	17.14%	23.87%
P15	SYNJ1	21	34025614	34025614	Missense_Mutation	SNP	C	T		3.00%	
P15	ESPN	1	6485254	6485254	Missense_Mutation	SNP	C	A		14.29%	16.67%
P15	CELA3A	1	22333988	22333988	Missense_Mutation	SNP	T	G	8.00%	2.63%	3.51%
P15	TINAGL1	1	32043024	32043024	Missense_Mutation	SNP	G	A		14.29%	5.26%
P15	FLNB	3	58139239	58139239	Missense_Mutation	SNP	G	A	36.84%	9.09%	38.05%
P15	ANKH	5	14769261	14769261	Missense_Mutation	SNP	C	G		3.17%	5.04%
P15	ITK	5	156649957	156649957	Missense_Mutation	SNP	C	T		1.41%	7.79%
P15	NSD3	8	38205049	38205049	Missense_Mutation	SNP	T	C	12.50%	18.24%	42.48%
P15	SYNPO2L	10	75408059	75408059	Missense_Mutation	SNP	G	T	46.34%	3.33%	29.17%
P15	GRIN2B	12	13716526	13716526	Missense_Mutation	SNP	G	A	23.53%	30.77%	33.00%
P15	CCDC63	12	111311720	111311720	Silent	SNP	G	A		1.80%	8.75%
P15	EP400	12	132445384	132445384	Missense_Mutation	SNP	A	G		8.16%	29.41%
P15	PABPC3	13	25671699	25671700	Missense_Mutation	DNP	GT	CC	18.75%	8.33%	7.69%
P15	MYH7	14	23889358	23889358	Missense_Mutation	SNP	C	T	33.33%	30.00%	39.60%
P15	KRT31	17	39551295	39551295	Missense_Mutation	SNP	G	A	13.73%	8.20%	17.19%
P15	FMNL1	17	43320687	43320687	Missense_Mutation	SNP	C	A	16.00%	26.67%	16.76%
P15	ABCA6	17	67124894	67124894	Missense_Mutation	SNP	T	A	2.56%	8.11%	12.99%
P15	ISOC2	19	55967828	55967828	Missense_Mutation	SNP	C	T	23.53%	49.06%	30.23%
P15	COL9A3	20	61461718	61461718	Missense_Mutation	SNP	C	T	30.77%	29.41%	42.67%
P15	KRTAP10-7	21	46020720	46020721	Missense_Mutation	DNP	CG	TA	15.79%	10.81%	29.82%
P15	RLIM	X	73811648	73811648	Missense_Mutation	SNP	G	A	6.25%	2.15%	6.90%
P15	OR10R2	1	158449770	158449770	Silent	SNP	C	T	8.45%	1.02%	
P15	PMS2	7	6042169	6042169	Missense_Mutation	SNP	C	T	18.18%	1.87%	
P15	USP17L2	8	11995987	11995987	Silent	SNP	G	A	13.33%	5.97%	
P15	USP17L2	8	11995998	11995998	Missense_Mutation	SNP	T	A	13.95%	6.25%	
P15	TRMT10B	9	37762640	37762640	Missense_Mutation	SNP	C	A	3.19%	0.62%	
P15	OR2M3	1	248367118	248367118	Missense_Mutation	SNP	T	G			3.24%
P15	FXR1	3	180679304	180679304	Missense_Mutation	SNP	G	A			7.84%
P15	DCLK2	4	151023867	151023867	Missense_Mutation	SNP	C	G			5.31%
P15	ZAN	7	100364730	100364730	Silent	SNP	G	A	40.00%		31.43%
P15	FUOM	10	135171495	135171495	Missense_Mutation	SNP	C	A	43.75%		24.24%
P15	NOX4	11	89075276	89075276	Missense_Mutation	SNP	C	T			14.29%
P15	TRIP11	14	92470558	92470558	Silent	SNP	C	T			26.95%
P15	MEIS2	15	37388589	37388589	Missense_Mutation	SNP	G	C			5.00%
P15	ACAN	15	89395238	89395238	Missense_Mutation	SNP	C	G			12.00%
P15	OR7A17	19	14991852	14991852	Missense_Mutation	SNP	G	C			12.68%
P15	GGT1	22	25016911	25016911	Missense_Mutation	SNP	C	T			4.00%
P15	MACF1	1	39854248	39854248	Missense_Mutation	SNP	C	T	21.95%		
P15	STIL	1	47726113	47726113	Missense_Mutation	SNP	G	T	12.07%		
P15	POGZ	1	151396606	151396606	Missense_Mutation	SNP	T	G	7.79%		
P15	YOD1	1	207222484	207222484	Missense_Mutation	SNP	A	G	21.21%		
P15	YOD1	1	207222486	207222486	Missense_Mutation	SNP	T	G	18.75%		
P15	AGT	1	230839956	230839956	Missense_Mutation	SNP	G	A	12.00%		
P15	EIF2B4	2	27587290	27587290	Nonsense_Mutation	SNP	G	A	19.05%		
P15	PLEKHB2	2	131890529	131890529	Missense_Mutation	SNP	C	T	15.79%		
P15	MGAT5	2	135075110	135075110	Silent	SNP	C	T	13.64%		
P15	BAZ2B	2	160295634	160295634	Missense_Mutation	SNP	G	T	13.64%		
P15	ITGA4	2	182399580	182399580	Missense_Mutation	SNP	G	A	14.63%		
P15	SLC25A38	3	39431041	39431041	Missense_Mutation	SNP	C	T	14.89%		
P15	PLXNB1	3	48459932	48459932	Missense_Mutation	SNP	T	G	18.18%		
P15	CCDC158	4	77283349	77283349	Missense_Mutation	SNP	G	T	7.06%		
P15	ADH4	4	100048452	100048452	Missense_Mutation	SNP	G	A	13.33%		
P15	SPEF2	5	35646854	35646854	Missense_Mutation	SNP	G	A	6.19%		
P15	C7	5	40958189	40958189	Missense_Mutation	SNP	C	G	12.66%		
P15	SLC30A5	5	68413125	68413125	Silent	SNP	G	A	8.45%		
P15	KCTD9	8	25290947	25290947	Missense_Mutation	SNP	G	A	12.77%		
P15	ITIH2	10	7755146	7755146	Missense_Mutation	SNP	C	T	7.50%		
P15	ERC1	12	1346030	1346030	Missense_Mutation	SNP	G	A	4.69%		
P15	MTHFD1	14	64916300	64916300	Missense_Mutation	SNP	C	T	12.50%		
P15	ZC3H14	14	89041181	89041181	Missense_Mutation	SNP	G	A	8.22%		
P15	CHST6	16	75513647	75513647	Missense_Mutation	SNP	G	A	12.77%		
P15	KSR1	17	25924315	25924315	Missense_Mutation	SNP	C	T	4.14%		
P15	NF1	17	29687605	29687605	Missense_Mutation	SNP	C	T	4.51%		
P15	PYCR1	17	79892801	79892801	Splice_Site	SNP	C	T	20.55%		

P15	IRF3		19	50168054	50168054	Silent	SNP	C	T	10.71%		
P15	POLD1		19	50912069	50912069	Silent	SNP	G	A	15.38%		
P15	SLC25A6	X		1505534	1505534	Silent	SNP	G	A	5.76%		
P15	MBNL3	X		131526278	131526278	Missense_Mutation	SNP	C	T	14.00%		
P16	PADI1		1	17552548	17552548	Missense_Mutation	SNP	A	C	17.65%	37.74%	61.54%
P16	POMGNT1		1	46655573	46655573	Nonsense_Mutation	SNP	G	A	11.59%		
P16	ZNF806		2	133075213	133075213	Missense_Mutation	SNP	A	G	40.00%	30.00%	13.04%
P16	PGAP1		2	197710708	197710708	Missense_Mutation	SNP	T	C	18.18%	19.44%	31.71%
P16	TRAK2		2	202245772	202245772	Missense_Mutation	SNP	C	G	9.46%		
P16	ITIH1		3	52816017	52816017	Missense_Mutation	SNP	C	A	18.52%	36.11%	21.05%
P16	VGLL3		3	87017991	87017991	Missense_Mutation	SNP	T	A	28.24%	30.41%	37.29%
P16	WDR5B		3	122133739	122133739	Missense_Mutation	SNP	C	G	7.08%		
P16	PRRT1		6	32118612	32118612	Missense_Mutation	SNP	C	G	4.48%	0.60%	1.00%
P16	DST		6	56483955	56483955	Missense_Mutation	SNP	C	T	5.00%		
P16	RAET1E		6	150209736	150209736	Silent	SNP	C	T	7.80%		
P16	RAC1		7	6442009	6442009	Missense_Mutation	SNP	G	A	6.90%		
P16	MUC12		7	100634454	100634454	Missense_Mutation	SNP	G	C	9.72%		
P16	CTTNBP2		7	117432597	117432597	Missense_Mutation	SNP	C	T	14.55%		
P16	ZNF777		7	149153028	149153028	Missense_Mutation	SNP	C	A	25.58%	19.15%	30.00%
P16	DPY19L4		8	95738648	95738648	Missense_Mutation	SNP	A	G	13.21%		
P16	FAM102A		9	130742323	130742323	Missense_Mutation	SNP	A	C	17.65%	30.00%	20.83%
P16	PRRC2B		9	134330488	134330488	Silent	SNP	T	A	26.32%	18.75%	55.56%
P16	NPFFR1		10	72014954	72014954	Missense_Mutation	SNP	C	A	20.59%	1.18%	
P16	PTEN		10	89720788	89720789	Frame_Shift_Del	DEL	G	-	10.91%	38.10%	58.93%
P16	EEF2KMT		16	5140254	5140254	Missense_Mutation	SNP	G	T	10.34%	2.78%	
P16	PIEZO1		16	88800317	88800317	Missense_Mutation	SNP	C	T	17.14%		
P16	WSB1		17	25638570	25638570	Missense_Mutation	SNP	C	T	6.25%		
P16	TMEM98		17	31266505	31266505	Missense_Mutation	SNP	G	A	10.00%		
P16	KAT2A		17	40271348	40271348	Missense_Mutation	SNP	G	A	11.32%		
P16	SPC24		19	11258566	11258566	Missense_Mutation	SNP	C	T	11.29%		
P16	LILRB1		19	55147987	55147988	Missense_Mutation	DNP	GA	CC	16.67%	6.35%	
P16	SERINC3		20	43150569	43150569	Silent	SNP	G	A	9.80%	7.14%	24.00%
P16	LAMA5		20	60897756	60897756	Silent	SNP	C	T	5.61%		
P16	KDM6A	X		44938582	44938582	Nonsense_Mutation	SNP	C	T	11.32%		
P16	MAGEA4	X		151092862	151092862	Missense_Mutation	SNP	G	T	12.50%	8.33%	7.14%
P16	USH2A		1	216051153	216051153	Missense_Mutation	SNP	C	A	32.79%	31.11%	62.50%
P16	NEK1		4	170384514	170384514	Missense_Mutation	SNP	G	A	8.33%	51.16%	13.51%
P16	PCDHB1		5	140432705	140432705	Silent	SNP	G	T	45.83%	29.41%	24.32%
P16	CARMIL1		6	25538141	25538141	Missense_Mutation	SNP	G	T	30.88%	34.29%	37.93%
P16	NF1		17	29556484	29556484	Splice_Site	SNP	G	A	6.90%	24.32%	20.00%
P16	CDC27		17	45249365	45249365	Missense_Mutation	SNP	C	T	2.38%	11.84%	8.05%
P16	DCAF12L1	X		125686024	125686024	Missense_Mutation	SNP	G	A	27.78%	20.30%	27.27%
P16	NEIL3		4	178231220	178231220	Missense_Mutation	SNP	G	T	31.76%	30.70%	51.52%
P16	ZNF716		7	57522810	57522810	Silent	SNP	C	A	25.84%	38.69%	26.25%
P16	MGAM		7	141731533	141731533	Silent	SNP	T	A	29.09%	38.61%	41.54%
P16	TRPM6		9	77376645	77376645	Silent	SNP	C	T	54.90%	55.07%	57.50%
P16	NPY4R		10	47087582	47087582	Missense_Mutation	SNP	C	G	2.96%	6.12%	5.71%
P16	OR4C5		11	48387784	48387784	Silent	SNP	G	T	19.30%	40.00%	33.96%
P16	KIFC3		16	57794765	57794765	Missense_Mutation	SNP	G	A	70.37%	64.71%	46.15%
P16	ZC3H4		19	47589701	47589701	In_Frame_Ins	INS	-	CTGCCCCTGCCTCGG	25.35%	21.95%	36.36%
P16	DSCAM		21	41719667	41719667	Silent	SNP	C	A	56.52%	20.00%	50.00%
P16	BRWD3	X		79990644	79990644	Missense_Mutation	SNP	A	T	48.15%	56.52%	55.00%
P16	GABPB2		1	151089955	151089955	Missense_Mutation	SNP	G	A			5.88%
P16	TRAM1L1		4	118006074	118006074	Missense_Mutation	SNP	C	A			15.79%
P16	NALCN		13	101763560	101763560	Missense_Mutation	SNP	C	T			7.23%
P16	FH		1	241663818	241663818	Missense_Mutation	SNP	C	T		7.69%	0.69%
P16	SUMO4		6	149721599	149721599	Silent	SNP	A	G		4.65%	2.06%
P16	USP17L1		8	7191336	7191336	Silent	SNP	A	G		8.45%	8.11%
P16	USP17L1		8	7191451	7191451	Missense_Mutation	SNP	G	T		5.08%	1.92%
P16	USP17L2		8	11995987	11995987	Silent	SNP	G	A		15.09%	2.63%
P16	GALE		1	24124721	24124721	Splice_Site	SNP	C	T		6.86%	
P16	RHBDL2		1	39384719	39384719	Missense_Mutation	SNP	C	T		8.54%	
P16	USP17L2		8	11995998	11995998	Missense_Mutation	SNP	T	A		16.67%	
P16	ALKAL1		8	53452430	53452430	Nonsense_Mutation	SNP	G	A		4.65%	
P16	TMC1		9	75445574	75445575	Frame_Shift_Del	DEL	A	-		3.77%	
P16	MIA2		14	39816923	39816923	Nonsense_Mutation	SNP	C	G		5.94%	
P16	ACSBG1		15	78472094	78472094	Missense_Mutation	SNP	C	T		7.59%	
P16	MORC4	X		106185268	106185268	Missense_Mutation	SNP	G	T		5.17%	
P17	ZNF669		1	247264387	247264387	Silent	SNP	C	G	10.00%		25.64%
P17	EXOSC9		4	122724103	122724103	Silent	SNP	C	T	10.00%	13.33%	16.98%
P17	WRN		8	30977813	30977813	Missense_Mutation	SNP	C	A	16.22%		
P17	ATG2B		14	96788915	96788915	Missense_Mutation	SNP	G	C	15.00%		10.87%
P17	NPAP1		15	24922294	24922294	Missense_Mutation	SNP	C	A	14.29%		
P17	GOLGA8H		15	30900683	30900683	Missense_Mutation	SNP	C	G	16.67%		10.00%
P17	ZNF799		19	12502862	12502862	Missense_Mutation	SNP	G	C	13.51%	16.28%	13.51%
P17	ARHGAP6	X		11272798	11272798	Silent	SNP	G	T	11.11%		
P17	DMD	X		32382827	32382827	Missense_Mutation	SNP	C	G	17.24%	28.57%	38.24%
P17	RPGR	X		38160587	38160587	Silent	SNP	G	A	19.35%	14.58%	9.43%
P17	ADAMTS19		5	129030478	129030478	Missense_Mutation	SNP	G	C	7.69%		16.67%

P17	OR51B2		11	5344976	5344976	Missense_Mutation	SNP	T	G	22.81%	14.04%	13.79%
P17	POTEG		14	19573100	19573100	Missense_Mutation	SNP	G	A	3.77%		6.90%
P17	CDC27		17	45221317	45221318	Missense_Mutation	DNP	TA	CC	12.24%		2.97%
P17	CDC27		17	45234360	45234360	Nonsense_Mutation	SNP	A	C	4.44%	11.76%	16.00%
P17	AMY2B		1	104117887	104117887	Silent	SNP	A	G	3.70%	8.57%	
P17	TARS1		5	33453409	33453409	Missense_Mutation	SNP	C	A	1.03%	2.97%	
P17	ZNF394		7	99091159	99091159	Missense_Mutation	SNP	C	T	3.12%	6.52%	26.15%
P17	RABGGTB		1	76257000	76257000	Missense_Mutation	SNP	C	T			3.77%
P17	GRIA1		5	153174271	153174271	Nonsense_Mutation	SNP	C	A			6.38%
P17	SFRP4		7	37951822	37951822	Silent	SNP	G	A			9.86%
P17	PLXDC2		10	20506493	20506493	Missense_Mutation	SNP	C	A			3.21%
P17	HPSE2		10	100904139	100904139	Missense_Mutation	SNP	C	T			14.29%
P17	HDC		15	50546860	50546860	Missense_Mutation	SNP	C	T			10.71%
P17	NFIA		1	61553998	61553998	Missense_Mutation	SNP	C	A		10.39%	
P17	RGS2		1	192780234	192780234	Missense_Mutation	SNP	G	A		8.75%	
P17	PTPN14		1	214571258	214571258	Nonsense_Mutation	SNP	G	T		13.04%	
P17	MAP4K4		2	102503607	102503607	Missense_Mutation	SNP	C	A		12.77%	
P17	ULK4		3	41723053	41723053	Missense_Mutation	SNP	C	T		9.84%	
P17	SMARCC1		3	47752247	47752247	Missense_Mutation	SNP	C	T		7.50%	
P17	RBM6		3	50099528	50099528	Missense_Mutation	SNP	G	A		5.44%	
P17	GTF2I		7	74119516	74119516	Missense_Mutation	SNP	G	A		7.27%	
P17	PCLO		7	82785691	82785691	Missense_Mutation	SNP	C	T		8.27%	
P17	CACNB2		10	18439879	18439879	Missense_Mutation	SNP	C	A		5.31%	
P18	GBP2		1	89575374	89575374	Missense_Mutation	SNP	C	T		12.96%	
P18	CCNC		6	100006390	100006390	Missense_Mutation	SNP	G	A		10.00%	
P18	PRPS1L1		7	18067301	18067301	Missense_Mutation	SNP	G	T		10.91%	2.17%
P18	TRIM55		8	67066345	67066345	Missense_Mutation	SNP	G	A		16.28%	4.00%
P18	EPPK1		8	144940725	144940725	Missense_Mutation	SNP	C	T	0.29%	3.09%	0.40%
P18	IFNA10		9	21206569	21206569	Silent	SNP	C	A	5.81%	10.96%	14.52%
P18	CDH23		10	73558971	73558971	Silent	SNP	C	A	61.90%	26.47%	50.00%
P18	LCOR		10	98709025	98709025	Nonsense_Mutation	SNP	A	T	55.56%	30.00%	30.95%
P18	OR5A1		11	59210722	59210722	Missense_Mutation	SNP	G	C	28.57%	19.51%	46.67%
P18	TPTE2		13	20010418	20010419	Missense_Mutation	DNP	CG	TC	23.68%	22.22%	27.27%
P18	DICER1		14	95577657	95577658	Frame_Shift_Del	DEL	T	-	56.58%	56.41%	54.29%
P18	TEX9		15	56676154	56676154	Missense_Mutation	SNP	A	T	22.22%	26.32%	18.18%
P18	COG4		16	70551534	70551534	Missense_Mutation	SNP	C	A		7.41%	
P18	USP32		17	58372067	58372067	Missense_Mutation	SNP	T	A		6.03%	
P18	BCAS3		17	59445832	59445832	Missense_Mutation	SNP	G	A		5.77%	
P18	SLC5A3		21	35469398	35469398	Missense_Mutation	SNP	G	C		9.23%	
P18	TEX11	X		69898664	69898664	Missense_Mutation	SNP	C	G		19.51%	
P18	AMY2B		1	104116929	104116929	Silent	SNP	A	T		1.42%	6.00%
P18	ANKRD20A2		9	42410356	42410356	Missense_Mutation	SNP	G	A		1.15%	6.54%
P18	NOD2		16	50733706	50733706	Silent	SNP	G	T	56.06%	66.67%	38.46%
P18	CDC27		17	45234343	45234343	Missense_Mutation	SNP	T	G	13.73%	4.55%	13.04%
P18	CDC27		17	45247393	45247393	Missense_Mutation	SNP	T	A		1.35%	9.09%
P18	SLK		10	105761229	105761229	Missense_Mutation	SNP	A	C	21.43%	42.50%	51.16%
P18	WFIKKN1		16	683313	683313	Silent	SNP	G	A	54.55%	43.48%	56.67%
P18	CDC27		17	45249330	45249330	Silent	SNP	T	G	5.75%	2.74%	6.72%
P18	USP17L2		8	11994842	11994842	Silent	SNP	T	C	4.41%		9.72%
P18	ANKRD18A		9	38577989	38577989	Missense_Mutation	SNP	C	T			8.11%
P18	CYP2C8		10	96827040	96827040	Missense_Mutation	SNP	T	C			16.33%
P18	ZNF257		19	22256329	22256329	Missense_Mutation	SNP	T	G	1.04%		6.90%
P18	FAM217B		20	58519887	58519887	Missense_Mutation	SNP	T	A			10.94%
P18	KALRN		3	124066062	124066062	Nonsense_Mutation	SNP	C	T	5.10%		
P18	WWC2		4	184169876	184169877	Missense_Mutation	DNP	AA	CT	17.39%		
P18	PCDHGA4		5	140736123	140736123	Silent	SNP	C	T	10.53%		
P18	KCP		7	128533894	128533894	Missense_Mutation	SNP	C	T	7.23%		
P18	CACNG5		17	64876760	64876760	Missense_Mutation	SNP	C	T	4.32%		
P18	CENPM		22	42335128	42335128	Missense_Mutation	SNP	C	T	6.90%		
P19	CD52		1	26646716	26646716	Missense_Mutation	SNP	G	A			13.79%
P19	ARHGAP29		1	94697078	94697078	Silent	SNP	C	T			17.65%
P19	COL11A1		1	103487276	103487276	Missense_Mutation	SNP	G	T			15.28%
P19	CEP350		1	179983395	179983395	Nonsense_Mutation	SNP	C	T			10.17%
P19	ELF3		1	201981097	201981097	Nonsense_Mutation	SNP	G	A			12.00%
P19	MARK1		1	220808765	220808765	Silent	SNP	C	A			21.21%
P19	LPIN1		2	11913774	11913774	Nonsense_Mutation	SNP	C	T			25.81%
P19	CNOT11		2	101885710	101885710	Silent	SNP	C	A			17.78%
P19	MCM6		2	136623734	136623734	Missense_Mutation	SNP	G	A		1.20%	8.33%
P19	CYTIP		2	158291259	158291259	Missense_Mutation	SNP	G	T			5.61%
P19	RESP18		2	220197896	220197896	Missense_Mutation	SNP	C	T			4.51%
P19	SPICE1		3	113166909	113166909	Missense_Mutation	SNP	G	A			15.79%
P19	TXNRD3		3	126349726	126349726	Silent	SNP	G	T			14.93%
P19	TMCC1		3	129442808	129442808	Missense_Mutation	SNP	G	T			6.45%
P19	TIPARP		3	156396114	156396114	Missense_Mutation	SNP	C	A			20.59%
P19	POLR2B		4	57891096	57891096	Silent	SNP	T	C			13.73%
P19	AFM		4	74364889	74364889	Missense_Mutation	SNP	C	A			18.42%
P19	SLC9B1		4	103832638	103832638	Missense_Mutation	SNP	C	T			30.00%
P19	FAM149A		4	187084073	187084073	Silent	SNP	G	A			11.32%
P19	NUP153		6	17628933	17628933	Missense_Mutation	SNP	G	T			9.68%

P19	BMP5	6	55684594	55684594	Missense_Mutation	SNP	C	T			15.38%
P19	UFL1	6	97000370	97000370	Missense_Mutation	SNP	C	A			13.56%
P19	ASCC3	6	101103574	101103574	Splice_Site	SNP	C	T			18.75%
P19	EPB41L2	6	131247757	131247757	Silent	SNP	G	A			17.65%
P19	SHPRH	6	146256533	146256533	Missense_Mutation	SNP	C	T			17.14%
P19	GRM1	6	146480707	146480707	Silent	SNP	C	T		1.41%	3.87%
P19	BZW2	7	16725652	16725652	Missense_Mutation	SNP	C	A			12.50%
P19	IKZF1	7	50444253	50444253	Silent	SNP	T	G			14.00%
P19	BAIAP2L1	7	97939779	97939779	Silent	SNP	T	C	27.50%	15.69%	10.71%
P19	COG5	7	106898761	106898761	Missense_Mutation	SNP	G	T			21.05%
P19	RBM28	7	127953292	127953292	Missense_Mutation	SNP	G	A			22.73%
P19	USP17L1	8	7191486	7191486	Silent	SNP	G	T			5.94%
P19	CDH17	8	95186418	95186418	Nonsense_Mutation	SNP	G	T			15.38%
P19	TJP2	9	71849446	71849446	Missense_Mutation	SNP	C	A			14.29%
P19	TRAF6	11	36514144	36514144	Missense_Mutation	SNP	G	A			10.53%
P19	CWF19L2	11	107326407	107326407	Silent	SNP	A	G			18.18%
P19	SDHD	11	111965581	111965581	Missense_Mutation	SNP	G	A			19.05%
P19	CD3E	11	118178024	118178024	Splice_Site	SNP	G	A			10.77%
P19	GLIPR1L1	12	75763870	75763870	Missense_Mutation	SNP	C	A			16.28%
P19	MPHOSPH9	12	123648541	123648541	Missense_Mutation	SNP	C	T			16.22%
P19	TMCO3	13	114157899	114157899	Missense_Mutation	SNP	G	A			34.21%
P19	SEC23A	14	39508296	39508296	Missense_Mutation	SNP	C	T			16.22%
P19	LTBP2	14	74973493	74973493	Missense_Mutation	SNP	C	T			9.23%
P19	VPS13C	15	62304324	62304324	Missense_Mutation	SNP	G	T			14.04%
P19	BLM	15	91347539	91347539	Missense_Mutation	SNP	G	A			19.35%
P19	KAT8	16	31131576	31131576	Missense_Mutation	SNP	T	A	32.00%	21.43%	20.00%
P19	SHCBP1	16	46652147	46652147	Nonsense_Mutation	SNP	G	A			15.85%
P19	MT1X	16	56717925	56717925	Nonsense_Mutation	SNP	C	A			17.07%
P19	COG4	16	70551559	70551559	Silent	SNP	G	A			9.20%
P19	SF3B3	16	70595592	70595592	Silent	SNP	C	T			18.75%
P19	GLG1	16	74526915	74526915	Nonsense_Mutation	SNP	G	A			29.27%
P19	GPATCH8	17	42477259	42477259	Missense_Mutation	SNP	C	T			16.67%
P19	MED13	17	60033185	60033185	Missense_Mutation	SNP	G	T			11.76%
P19	ZNF101	19	19791092	19791092	Silent	SNP	C	T		5.26%	13.64%
P19	ZNF101	19	19791101	19791101	Nonsense_Mutation	SNP	G	T			13.95%
P19	SIGLEC1	20	3674929	3674929	Silent	SNP	A	G			3.64%
P19	ZFP64	20	50704984	50704984	Missense_Mutation	SNP	G	A			16.22%
P19	THOC5	22	29939466	29939466	Silent	SNP	G	A			12.77%
P19	SLC25A6	X	1505498	1505498	Silent	SNP	G	T			2.14%
P19	OFD1	X	13775833	13775833	Missense_Mutation	SNP	C	A			15.69%
P19	HUWE1	X	53613843	53613843	Silent	SNP	C	T			14.29%
P19	CHM	X	85236789	85236789	Nonsense_Mutation	SNP	C	T			8.43%
P19	PPP2R5A	1	212534032	212534032	Missense_Mutation	SNP	C	A		6.00%	9.52%
P19	CFAP92	3	128696921	128696921	Missense_Mutation	SNP	C	T	2.65%	4.05%	6.21%
P19	HAUS3	4	2242278	2242278	Missense_Mutation	SNP	G	T	13.89%	19.51%	5.00%
P19	CLCN3	4	170608847	170608847	Silent	SNP	A	T	23.53%	16.00%	11.11%
P19	ZNF804B	7	88962974	88962974	Missense_Mutation	SNP	A	T	11.43%	10.71%	3.12%
P19	KCNMA1	10	78771724	78771724	Splice_Site	SNP	C	T		2.50%	3.45%
P19	TEP1	14	20848433	20848433	Missense_Mutation	SNP	C	T	9.09%	16.07%	5.56%
P19	DICER1	14	95560321	95560321	Missense_Mutation	SNP	C	G	46.88%	19.35%	50.00%
P19	SPAG17	1	118623799	118623799	Missense_Mutation	SNP	G	A	10.71%		1.39%
P19	SLC2A2	3	170715836	170715836	Silent	SNP	G	T	19.35%	12.12%	6.25%
P19	SPEN	1	16258741	16258741	Missense_Mutation	SNP	T	A		9.64%	
P19	PGM1	1	64117370	64117370	Silent	SNP	C	T	4.35%	8.96%	
P19	PEX11B	1	145518205	145518205	Silent	SNP	C	T		9.09%	
P19	TMEM79	1	156256161	156256161	Missense_Mutation	SNP	C	T		15.79%	
P19	DDR2	1	162740130	162740130	Silent	SNP	C	T		20.00%	
P19	HNRNPU	1	245021396	245021396	Missense_Mutation	SNP	C	G		9.38%	
P19	C2orf50	2	11273517	11273517	Silent	SNP	G	A		30.93%	
P19	LTBP1	2	33586514	33586514	Missense_Mutation	SNP	G	A		8.27%	
P19	EML6	2	55071272	55071272	Silent	SNP	G	A		6.90%	
P19	HNRNPA3	2	178081295	178081295	Silent	SNP	G	A		12.00%	
P19	TRIP12	2	230656894	230656894	Nonsense_Mutation	SNP	G	C		8.14%	
P19	AQP12A	2	241631369	241631369	Silent	SNP	C	T		15.38%	
P19	WDR48	3	39135434	39135434	Silent	SNP	C	A		19.35%	
P19	FBXO40	3	121340879	121340879	Silent	SNP	G	A		5.41%	
P19	ILDR1	3	121720620	121720620	Silent	SNP	G	T		8.24%	
P19	SLC12A8	3	124829080	124829080	Missense_Mutation	SNP	T	C		12.50%	
P19	XRN1	3	142094700	142094700	Missense_Mutation	SNP	A	T		7.23%	
P19	TMEM41A	3	185212411	185212411	Missense_Mutation	SNP	C	T		10.29%	
P19	FRYL	4	48537712	48537712	Missense_Mutation	SNP	A	T		10.00%	
P19	DCHS2	4	155180788	155180788	Missense_Mutation	SNP	T	G		14.63%	
P19	C7	5	40955520	40955520	Silent	SNP	G	A		9.23%	
P19	APC	5	112163667	112163667	Silent	SNP	G	T		17.95%	
P19	HSPA4	5	132408997	132408997	Nonsense_Mutation	SNP	C	T		22.22%	
P19	FAM83B	6	54791271	54791271	Missense_Mutation	SNP	T	A		10.61%	
P19	IBTK	6	82914613	82914613	Missense_Mutation	SNP	G	A		12.73%	
P19	HACE1	6	105219808	105219808	Missense_Mutation	SNP	T	C		14.75%	
P19	FNDC1	6	159655481	159655481	Nonsense_Mutation	SNP	C	T		27.78%	

P19	ACAT2	6	160184078	160184078	Missense_Mutation	SNP	G	C		15.22%	
P19	BZW2	7	16725603	16725603	Missense_Mutation	SNP	G	A		2.99%	
P19	BAZ1B	7	72891453	72891453	Missense_Mutation	SNP	G	A		7.89%	
P19	RBM33	7	155499735	155499735	Silent	SNP	G	A		10.47%	
P19	DOK2	8	21767183	21767183	Missense_Mutation	SNP	C	T	21.88%	7.69%	
P19	SLCO5A1	8	70585448	70585448	Missense_Mutation	SNP	G	A		2.96%	
P19	RDH10	8	74209489	74209489	Missense_Mutation	SNP	G	A		16.00%	
P19	EFR3A	8	132952779	132952779	Missense_Mutation	SNP	G	A		5.08%	
P19	NSUN6	10	18903422	18903422	Missense_Mutation	SNP	C	T		12.50%	
P19	AIFM2	10	71880935	71880935	Silent	SNP	C	T	2.53%	4.09%	
P19	CCDC81	11	86133678	86133678	Missense_Mutation	SNP	A	T	10.00%	20.00%	
P19	ACAT1	11	108017050	108017050	Missense_Mutation	SNP	T	A		16.07%	
P19	C12orf57	12	7055016	7055016	Silent	SNP	G	A		6.67%	
P19	CSNK1A1L	13	37678594	37678594	Missense_Mutation	SNP	C	T		11.86%	
P19	RNASEH2B	13	51517472	51517472	Missense_Mutation	SNP	A	C		23.64%	
P19	COQ6	14	74428600	74428600	Silent	SNP	A	G		12.50%	
P19	TCF12	15	57212185	57212185	Missense_Mutation	SNP	C	T		6.67%	
P19	DENND4A	15	65960419	65960419	Missense_Mutation	SNP	C	G		20.83%	
P19	FBXO22	15	76205617	76205617	Missense_Mutation	SNP	G	A		17.07%	
P19	RNF40	16	30776624	30776624	Silent	SNP	G	A		4.83%	
P19	PSME3IP1	16	57207746	57207746	Silent	SNP	A	T		4.57%	
P19	CRISPLD2	16	84900554	84900554	Missense_Mutation	SNP	C	T		3.66%	
P19	YWHAE	17	1264477	1264477	Missense_Mutation	SNP	G	C		23.08%	
P19	SLC13A5	17	6590962	6590962	Silent	SNP	C	T		14.67%	
P19	MYH10	17	8397150	8397150	Silent	SNP	C	T		4.52%	
P19	ARHGAP44	17	12819325	12819325	Silent	SNP	G	A		14.38%	
P19	KPNA2	17	66041948	66041948	Missense_Mutation	SNP	T	G	4.76%	8.57%	
P19	ZC3H4	19	47597824	47597824	Missense_Mutation	SNP	C	T		3.70%	
P19	CST7	20	24939636	24939636	Missense_Mutation	SNP	C	T		4.65%	
P19	PYGB	20	25252076	25252076	Missense_Mutation	SNP	G	A		10.00%	
P19	KRTAP21-1	21	32127679	32127679	Silent	SNP	G	A		10.34%	
P19	SAMM50	22	44368837	44368837	Nonsense_Mutation	SNP	T	A		11.76%	
P19	STS	X	7223152	7223152	Missense_Mutation	SNP	G	A		12.70%	
P19	ARHGAP6	X	11272788	11272788	Missense_Mutation	SNP	G	A		1.53%	
P19	KLF8	X	56295915	56295915	Nonsense_Mutation	SNP	C	T		3.43%	
P19	NKAP	X	119070327	119070327	Silent	SNP	C	T		4.84%	
P19	MAP7D3	X	135326810	135326810	Missense_Mutation	SNP	T	A		1.99%	
P19	GFPT1	2	69597212	69597212	Missense_Mutation	SNP	T	A	21.88%	18.75%	
P19	KRT3	12	53189241	53189241	Missense_Mutation	SNP	C	T	14.29%	12.73%	
P19	MYCBP	1	39333246	39333246	Missense_Mutation	SNP	T	C	18.03%		
P19	MTMR11	1	149903306	149903306	Missense_Mutation	SNP	C	T	9.52%		
P19	EPC2	2	149526792	149526792	Missense_Mutation	SNP	G	A	6.19%		
P19	PLCL1	2	198949805	198949805	Missense_Mutation	SNP	C	T	8.96%		
P19	TUBA4A	2	220115085	220115085	Missense_Mutation	SNP	C	T	13.95%		
P19	TMCC1	3	129442827	129442827	Missense_Mutation	SNP	G	A	8.57%		
P19	CBR4	4	169923325	169923325	Silent	SNP	G	T	10.53%		
P19	FSCN3	7	127235643	127235643	Missense_Mutation	SNP	C	A	17.14%		
P19	AGPAT5	8	6588294	6588294	Missense_Mutation	SNP	G	T	10.71%		
P19	HEATR6	17	58147194	58147194	Missense_Mutation	SNP	C	T	12.77%		
P20	PRDX1	1	45981340	45981340	Silent	SNP	G	A			9.20%
P20	SMAD4	18	48584790	48584790	Missense_Mutation	SNP	C	A			5.88%
P20	TTC17	11	43419541	43419541	Missense_Mutation	SNP	C	A		4.29%	0.50%
P20	VPS13B	8	100844628	100844628	Missense_Mutation	SNP	G	A	9.30%		6.48%
P20	EPHX1	1	226027012	226027012	Silent	SNP	C	T	10.13%	5.33%	
P20	ACTL6A	3	179291231	179291231	Missense_Mutation	SNP	C	T		12.50%	
P20	ACTC1	15	35083496	35083496	Missense_Mutation	SNP	C	T		2.64%	
P20	ALOX12B	17	7990662	7990662	Silent	SNP	G	A		6.06%	
P20	AFG3L2	18	12353022	12353022	Silent	SNP	G	A		7.06%	
P20	HECW1	7	43540849	43540849	Nonsense_Mutation	SNP	C	T	6.19%	1.63%	
P20	KIFC1	6	33377445	33377445	Missense_Mutation	SNP	C	T	4.19%		
P20	LIMA1	12	50589643	50589643	Missense_Mutation	SNP	C	T	8.33%		
P20	GALNT6	12	51752036	51752036	Missense_Mutation	SNP	C	T	8.11%		
P20	XRCC1	19	44047554	44047554	Missense_Mutation	SNP	G	A	6.50%		

Supplementary Data 3. Significant focal level SCNA in H-FLAC and L-FLAC

Significant focal level SCNA in H-FLAC patients

Unique Name	Descriptor	Wide Peak Limits	Peak Limits	Region Limits	q values	Residual q values after removing segments shared with higher peaks
Amplification	1q21.3	chr1:151969447-153325683(probes 8150:8168)	chr1:151979411-153317867(probes 8151:8167)	chr1:151979411-153346299(probes 8151:8170)	0.020386	0.020386
Amplification	4p16.3	chr4:328362-896485(probes 33324:33334)	chr4:338301-886492(probes 33325:33333)	chr4:338301-916472(probes 33325:33336)	0.025456	0.025456
Amplification	11q13.3	chr11:69063901-70921195(probes 82611:82694)	chr11:69070801-70911387(probes 82612:82693)	chr11:69070801-70940811(probes 82612:82696)	0.025592	0.025592
Amplification	11q13.4	chr11:74340301-75072559(probes 82884:82932)	chr11:74345501-75062699(probes 82885:82931)	chr11:74336701-75142709(probes 82883:82940)	0.0014835	0.0014835
Amplification	14q13.3	chr14:34995407-39881588(probes 96072:96274)	chr14:35005331-39871599(probes 96073:96273)	chr14:33203037-50248749(probes 96009:96661)	2.54E-05	3.45E-05
Amplification	14q23.1	chr14:58718901-60203249(probes 97129:97226)	chr14:58719001-60193799(probes 97130:97225)	chr14:52186967-61457528(probes 96777:97283)	5.60E-08	7.31E-08
Amplification	19q12	chr19:29831689-30511437(probes 112900:112906)	chr19:29841512-30501549(probes 112901:112905)	chr19:24309501-37593957(probes 112871:113179)	5.60E-08	2.53E-07
Amplification	19q13.11	chr19:32845601-33934407(probes 113021:113036)	chr19:32845901-33924438(probes 113022:113035)	chr19:24309501-37593957(probes 112871:113179)	4.50E-05	0.058139
Amplification	20q13.12	chr20:41914066-44002089(probes 115920:115990)	chr20:41923950-43992199(probes 115921:115989)	chr20:41306901-44925041(probes 115885:116009)	0.020386	0.042507
Amplification	20q13.33	chr20:57611991-63025520(probes 116583:116659)	chr20:57621830-63025520(probes 116584:116659)	chr20:54913880-63025520(probes 116463:116659)	0.0014835	0.002167
Deletion	2q37.3	chr2:235609530-243199373(probes 23759:23894)	chr2:236319308-243199373(probes 23780:23894)	chr2:236539239-243199373(probes 23782:23894)	0.081361	0.081361
Deletion	5q33.1	chr5:148028940-167724830(probes 47341:48285)	chr5:148108921-154881889(probes 47349:47638)	chr5:148118919-148829174(probes 47350:47351)	0.028996	0.028996
Deletion	6p22.2	chr6:25773536-26488784(probes 49693:49722)	chr6:25783533-26488784(probes 49694:49722)	chr6:26017601-26037827(probes 49697:49698)	0.10029	0.10029
Deletion	6q22.1	chr6:111536750-116621066(probes 53092:53309)	chr6:111566746-116621066(probes 53095:53309)	chr6:96044701-171115067(probes 52402:55588)	0.00038729	0.00038729
Deletion	13q14.2	chr13:48658165-50316089(probes 92795:92829)	chr13:48668161-50316089(probes 92796:92829)	chr13:1-58236699(probes 91489:93028)	0.0038057	0.0038057
Deletion	14q24.2	chr14:68886401-107349540(probes 97695:99380)	chr14:69236401-80431028(probes 97710:98176)	chr14:69256401-69276597(probes 97712:97713)	0.052807	0.052807
Deletion	17p13.2	chr17:1-8834974(probes 105674:105847)	chr17:1-8834974(probes 105674:105847)	chr17:1-19680411(probes 105674:106317)	3.24E-08	3.47E-05
Deletion	17p12	chr17:10560158-19303060(probes 105966:106316)	chr17:10570139-19303060(probes 105967:106316)	chr17:1-19680411(probes 105674:106317)	6.98E-07	0.068717
Deletion	19q13.33	chr19:45967761-58142832(probes 113525:113687)	chr19:45977544-58142832(probes 113526:113687)	chr19:45997109-46436951(probes 113528:113529)	0.081361	0.081361
Deletion	20q12	chr20:31004795-51592745(probes 115379:116366)	chr20:31808310-51016676(probes 115394:116308)	chr20:31044201-52861472(probes 115383:116398)	0.016705	0.016705

Significant focal level SCNA in L-FLAC patients

Unique Name	Descriptor	Wide Peak Limits	Peak Limits	Region Limits	q values	Residual q values after removing segments shared with higher peaks
Amplification	1p34.3	chr1:33917201-35740049(probes 1452:1536)	chr1:33926726-35730054(probes 1453:1535)	chr1:33838101-35760038(probes 1443:1538)	0.029849	0.03347
Amplification	1p33	chr1:44492764-52030216(probes 1968:2462)	chr1:44502176-52020284(probes 1969:2461)	chr1:44502176-52050079(probes 1969:2464)	0.029849	0.038369
Amplification	1q21.3	chr1:151969686-153332428(probes 8203:8220)	chr1:151979639-153322718(probes 8204:8219)	chr1:151979639-153351847(probes 8204:8222)	0.00097699	0.0011015
Amplification	1q23.1	chr1:157057601-159237524(probes 8441:8497)	chr1:157067321-159227605(probes 8442:8496)	chr1:156814201-159780783(probes 8415:8532)	0.022308	0.029611
Amplification	2q37.2	chr2:237019330-237735863(probes 23790:23814)	chr2:237029301-237725864(probes 23791:23813)	chr2:234374179-237755861(probes 23673:23816)	0.029037	0.029037
Amplification	5q31.3	chr5:139973015-140866261(probes 47588:47616)	chr5:139982904-140856264(probes 47589:47615)	chr5:139982904-140886255(probes 47589:47618)	0.038644	0.038644
Amplification	5q34	chr5:159625278-180915260(probes 48566:49392)	chr5:159635253-180915260(probes 48567:49392)	chr5:159635253-180915260(probes 48567:49392)	0.035201	0.035201
Amplification	6p22.1	chr6:25781506-37459516(probes 50352:50438)	chr6:25791505-37449545(probes 50353:50437)	chr6:25791505-37479458(probes 50353:50440)	0.029849	0.029849
Amplification	6p12.3	chr6:49171319-52063904(probes 51019:51137)	chr6:49181297-52053999(probes 51020:51136)	chr6:49181297-52133778(probes 51020:51145)	0.022308	0.022308
Amplification	8q24.3	chr8:80668401-146364022(probes 65559:68294)	chr8:142159309-146364022(probes 68276:68294)	chr8:80678401-146364022(probes 65560:68294)	0.022308	0.022308
Amplification	11p11.11	chr11:48006808-56747441(probes 83272:83719)	chr11:48016808-56737584(probes 83273:83718)	chr11:48016808-57023697(probes 83273:83726)	0.0016531	0.0016531
Amplification	12q13.13	chr12:52562770-53542944(probes 89299:89309)	chr12:52572762-53532945(probes 89300:89308)	chr12:52572762-53562942(probes 89300:89311)	0.043338	0.043338
Amplification	13q34	chr13:111875752-115169878(probes 97214:97240)	chr13:111885743-115169878(probes 97215:97240)	chr13:111885743-115169878(probes 97215:97240)	0.0062524	0.0062524
Amplification	17q21.2	chr17:38990835-39990638(probes 110071:110088)	chr17:38991033-39983807(probes 110072:110087)	chr17:38991033-40004299(probes 110072:110090)	0.053028	0.053028
Deletion	1q23.3	chr1:161071701-161199799(probes 8643:8655)	chr1:161071701-161199799(probes 8643:8655)	chr1:161070701-161229170(probes 8642:8658)	0.010665	0.010665
Deletion	2p11.2	chr2:85117933-86070837(probes 16653:16675)	chr2:85117933-86070837(probes 16653:16675)	chr2:85629201-85680632(probes 16655:16656)	0.010665	0.010665
Deletion	5q31.3	chr5:139943346-140906249(probes 47585:47620)	chr5:139973015-140906249(probes 47588:47620)	chr5:139982904-140365185(probes 47589:47590)	0.00019106	0.00019106
Deletion	6p22.2	chr6:25781506-26467317(probes 50352:50377)	chr6:25781506-26467317(probes 50352:50377)	chr6:25791505-26531697(probes 50353:50382)	0.00018253	0.00018253
Deletion	6q21	chr6:108498536-109616484(probes 53928:53992)	chr6:108498536-109496496(probes 53928:53980)	chr6:108768501-108817915(probes 53935:53936)	0.022143	0.02569
Deletion	6q25.1	chr6:151932345-153335756(probes 56091:56149)	chr6:152112327-152510530(probes 56109:56126)	chr6:152202319-152232314(probes 56118:56119)	0.010665	0.011558
Deletion	10q22.2	chr10:75488401-76810187(probes 78016:78150)	chr10:75497001-76810187(probes 78017:78150)	chr10:75497001-76757249(probes 78017:78142)	0.010775	0.011558
Deletion	12p13.31	chr12:5913541-7508113(probes 87120:87152)	chr12:5923539-7498233(probes 87121:87151)	chr12:5933538-7018862(probes 87122:87123)	0.010665	0.011558
Deletion	12q13.2	chr12:55619727-56563049(probes 89408:89433)	chr12:55888194-56563049(probes 89409:89433)	chr12:55898137-56160014(probes 89410:89411)	0.010665	0.010665
Deletion	14q12	chr14:24045646-25048989(probes 97341:97366)	chr14:24055519-25048989(probes 97342:97366)	chr14:24065392-24633272(probes 97343:97344)	0.010665	0.011558
Deletion	17q21.1	chr17:38093006-38528761(probes 109997:110021)	chr17:38103005-38528761(probes 109998:110021)	chr17:38133001-38153046(probes 110001:110002)	0.088953	0.087092
Deletion	19p13.2	chr19:1-24277544(probes 115157:115757)	chr19:1-24267548(probes 115157:115756)	chr19:1-24287541(probes 115157:115758)	0.010665	0.011558

Supplementary Data 4. Differentially methylation regions (DMR) and Differentially expressed genes (DEG) between tumor and normal samples

a Top Differentially methylation regions (DMR) between tumor and normal samples in H-FLAC

chr	start	end	pvalue	qvalue	meth.diff	dmr.type
chr8	101822101	101822200	3.97E-121	2.24E-120	50.87183408	hypermethylation
chr8	101822001	101822100	0	0	46.06952423	hypermethylation
chr14	37136301	37136400	1.51E-300	2.73E-298	42.72871696	hypermethylation
chr16	1581001	1581100	3.00E-238	4.27E-236	40.0470837	hypermethylation
chr1	214153401	214153500	9.28E-303	1.19E-300	39.65137692	hypermethylation
chr16	1584401	1584500	4.68E-84	6.14E-83	39.43956256	hypermethylation
chr6	100896001	100896100	1.74E-177	5.09E-176	39.10843834	hypermethylation
chr19	58715301	58715400	1.68E-238	2.16E-236	38.68795825	hypermethylation
chr7	27178801	27178900	2.07E-69	8.94E-69	38.59875042	hypermethylation
chr19	58715601	58715700	1.20E-196	9.15E-195	38.57084782	hypermethylation
chr2	114035501	114035600	5.47E-76	3.78E-75	38.51644615	hypermethylation
chr3	128764901	128765000	2.39E-106	6.46E-105	37.99833773	hypermethylation
chr14	37136201	37136300	0	0	37.75247305	hypermethylation
chr1	146550401	146550500	2.61E-283	3.08E-281	37.73688238	hypermethylation
chr7	156797401	156797500	5.18E-226	3.35E-224	37.70200665	hypermethylation
chr16	1584501	1584600	2.66E-159	1.82E-157	37.70169918	hypermethylation
chr19	35159301	35159400	2.09E-104	4.52E-103	-46.89804916	hypomethylation
chr7	145058201	145058300	6.03E-121	6.90E-120	-47.23091273	hypomethylation
chr7	49750601	49750700	6.80E-42	1.65E-41	-47.60933459	hypomethylation
chr1	2731001	2731100	1.36E-34	5.10E-34	-47.7849478	hypomethylation
chr7	152852101	152852200	2.81E-139	4.40E-138	-47.87396785	hypomethylation
chr5	44975001	44975100	4.30E-100	3.86E-99	-48.01352759	hypomethylation
chr7	49750701	49750800	2.29E-112	2.21E-111	-48.32915248	hypomethylation
chr5	7964601	7964700	2.55E-106	2.46E-105	-48.36074291	hypomethylation
chr3	153807201	153807300	3.13E-227	6.52E-225	-49.04081027	hypomethylation
chr10	133951901	133952000	0	0	-49.47012549	hypomethylation
chr1	69329601	69329700	4.01E-201	2.23E-199	-50.03500626	hypomethylation
chr6	64151801	64151900	1.48E-240	6.26E-239	-50.21227968	hypomethylation
chr1	237473401	237473500	2.14E-235	1.84E-233	-50.65955053	hypomethylation
chr10	133952001	133952100	3.73E-119	4.96E-118	-50.96066296	hypomethylation
chr10	133951801	133951900	0	0	-51.21395303	hypomethylation
chr6	18123201	18123300	0	0	-51.3272369	hypomethylation
chr17	80278501	80278600	3.36E-90	5.31E-89	-51.44262131	hypomethylation
chr6	64151901	64152000	3.18E-146	6.89E-145	-52.27364656	hypomethylation
chr10	98129901	98130000	2.36E-233	1.52E-231	-53.83670094	hypomethylation
chr3	153807101	153807200	8.46E-274	2.43E-271	-55.05162001	hypomethylation

b. Top Differentially methylation regions (DMR) between tumor and normal samples in L-FLAC

chr	start	end	pvalue	qvalue	meth.diff	dmr.type
chr6	27228201	27228300	1.08E-141	8.93E-140	26.91971708	hypermethylation
chr1	17020401	17020500	3.89E-191	3.30E-189	25.30748161	hypermethylation
chr10	63212201	63212300	2.88E-110	8.97E-109	24.22724358	hypermethylation
chr6	50808601	50808700	2.65E-106	1.28E-104	22.70596104	hypermethylation
chr6	27182901	27183000	5.60E-168	6.50E-166	22.69579445	hypermethylation
chr1	17020301	17020400	8.17E-241	1.04E-238	22.3968666	hypermethylation
chr6	30459501	30459600	4.22E-30	3.77E-29	22.24557252	hypermethylation
chr14	61119201	61119300	1.12E-85	1.86E-83	21.43141421	hypermethylation
chr13	113584101	113584200	9.16E-43	5.60E-42	21.32940773	hypermethylation
chr17	76840401	76840500	1.78E-06	4.69E-06	20.95871982	hypermethylation
chr1	17086201	17086300	6.72E-76	1.37E-74	20.4683769	hypermethylation

chr1	17051601	17051700	7.24E-50	1.15E-48	20.2446265	hypermethylation
chr2	157178801	157178900	5.31E-43	1.18E-41	20.07273557	hypermethylation
chr20	57393201	57393300	8.03E-64	1.58E-62	-35.41165903	hypomethylation
chr20	62209601	62209700	2.56E-242	1.13E-240	-35.41993847	hypomethylation
chr16	88442501	88442600	3.76E-135	1.42E-133	-35.459122	hypomethylation
chr15	29431401	29431500	6.40E-123	6.78E-121	-35.59177874	hypomethylation
chr17	79958601	79958700	8.39E-84	2.49E-82	-35.70702595	hypomethylation
chr17	73996201	73996300	7.74E-90	2.71E-88	-35.8393396	hypomethylation
chr5	1912101	1912200	4.73E-196	8.87E-194	-36.10421961	hypomethylation
chr16	84885201	84885300	1.89E-104	5.95E-103	-36.17078651	hypomethylation
chr9	137673001	137673100	1.23E-54	3.83E-53	-36.41854584	hypomethylation
chr13	113429201	113429300	3.05E-78	3.56E-77	-36.51843166	hypomethylation
chr16	21567001	21567100	0	0	-37.89018786	hypomethylation
chr5	859601	859700	3.51E-119	1.64E-117	-38.10842457	hypomethylation
chr20	60470001	60470100	9.13E-92	2.70E-90	-38.84116633	hypomethylation
chr7	105279401	105279500	4.89E-109	3.33E-107	-39.45913139	hypomethylation
chr13	111979001	111979100	1.71E-117	3.14E-116	-39.56210345	hypomethylation
chr3	153807101	153807200	4.25E-94	7.32E-92	-39.8399228	hypomethylation
chr17	42346901	42347000	1.98E-49	2.94E-48	-40.02281781	hypomethylation
chr5	16721801	16721900	2.32E-92	5.44E-91	-41.15034299	hypomethylation
chr13	113474101	113474200	0	0	-44.3289352	hypomethylation
chr13	113472501	113472600	0	0	-50.50621528	hypomethylation

c. Top Differentially expressed genes (DEG) between tumor and normal samples in H-FLAC

GeneID	GeneName	Log2FoldChange	pvalue	padjust
ENSG00000110917.3	MLEC	1.15051642886912	9.99647846918364e-05	0.0010
ENSG00000223572.5	CKMT1A	3.8704365399668	9.98338033050148e-07	0.0000
ENSG00000137507.7	LRRC32	-1.39182681108252	9.9676116983307e-09	0.0000
ENSG00000118939.13	UCHL3	1.55531539413364	9.95234053380653e-06	0.0001
ENSG00000166961.10	MS4A15	-2.55776579310556	9.9494561097345e-05	0.0010
ENSG00000256655.1	TMEM75	1.6604209370222	9.94890791295655e-05	0.0010
ENSG00000173848.14	NET1	1.08443567937643	9.92201616527929e-06	0.0001
ENSG00000100307.8	CBX7	-1.83393907703652	9.90527504325178e-12	0.0000
ENSG00000160957.8	RECQL4	3.39399461036923	9.9034271480539e-18	0.0000
ENSG00000046889.14	PREX2	-2.01191760293172	9.87767749419613e-08	0.0000
ENSG00000124615.13	MOCS1	-1.42881615637927	9.86578437088224e-07	0.0000
ENSG00000104415.9	WISP1	2.72550704322085	9.8603115756788e-17	0.0000
ENSG00000115306.11	SPTBN1	-1.74804275420941	9.85614153041585e-18	0.0000
ENSG00000159063.8	ALG8	1.66049160317924	9.85132170819758e-08	0.0000
ENSG00000230615.2	RP5-1198O20.4	-2.09195025372517	9.85105027931659e-05	0.0010
ENSG00000187840.4	EIF4EBP1	1.56084265433445	9.85066284711608e-07	0.0000
ENSG00000068001.9	HYAL2	-1.28504482072585	9.85058637495527e-13	0.0000
ENSG00000198937.8	CCDC167	1.77943061978389	9.84514756635643e-08	0.0000
ENSG00000180440.3	SERTM1	-3.3001336760747	9.84344005067055e-06	0.0001
ENSG00000149418.6	ST14	2.22100949264284	9.83160469120815e-12	0.0000
ENSG00000043143.16	PHF15	-1.00061992849755	2.67629511694709e-06	0.0000
ENSG00000163697.12	APBB2	-1.00111723755699	4.75426084332297e-15	0.0000
ENSG00000227155.3	RP11-165F24.3	-1.00316627087045	0.00147170630916432	0.0092
ENSG00000232611.1	RP11-1114A5.4	-1.00341213533426	0.000410119118449049	0.0032
ENSG00000151689.8	INPP1	-1.00384857024212	1.25935889170383e-05	0.0002
ENSG00000070614.10	NDST1	-1.00423005386325	5.89190705918435e-05	0.0007
ENSG00000142227.6	EMP3	-1.00461145322661	2.0395249729664e-05	0.0003
ENSG00000177272.7	KCNA3	-1.00475311372185	8.78122411212346e-07	0.0000
ENSG00000129538.9	RNASE1	-1.00528896605507	0.000976341677726179	0.0066
ENSG00000197121.10	PGAP1	-1.00557349088542	0.00155635609803434	0.0096

ENSG00000122042.9	UBL3	-1.00572463258113	3.33510972120635e-06	0.0001
ENSG00000061918.8	GUCY1B3	-1.00618217939999	3.40681524747177e-05	0.0004
ENSG00000065989.11	PDE4A	-1.00623335638535	0.000216554790133124	0.0019
ENSG00000172348.10	RCAN2	-1.00782906074771	0.000412096768995534	0.0033
ENSG00000197057.4	DTHD1	-1.00834822047495	0.00144437790295454	0.0091
ENSG00000224086.3	LL22NC03-86G7.	-1.00924576932333	0.000324437112351403	0.0027
ENSG00000163820.10	FYCO1	-1.00968371884312	2.34794052699977e-07	0.0000
ENSG00000175489.9	LRRC25	-1.01070486530381	0.000617658676011945	0.0045
ENSG00000241014.1	RP11-244H3.1	-1.010737019652	1.69830084799381e-06	0.0000
ENSG00000185989.9	RASA3	-1.01084439034988	0.00101449227019905	0.0068

d. Top Differentially expressed genes (DEG) between tumor and normal samples in L-FLAC

GeneID	GeneName	Log2FoldChange	pvalue	padjust
ENSG00000170608.2	FOXA3	5.46190520058625	1.17531552422553e-10	1.46E-08
ENSG00000143320.4	CRABP2	5.07727431500205	1.40869911522651e-22	2.11E-19
ENSG00000130294.10	KIF1A	4.99025363300608	3.97380694793914e-13	1.02E-10
ENSG00000146166.12	LGSN	4.8818332745113	4.80798341149338e-18	3.71E-15
ENSG00000172461.6	FUT9	4.49454550403029	7.36360007803582e-08	4.42E-06
ENSG00000242575.1	AC012501.3	4.37954249607799	1.65509183625103e-07	8.65E-06
ENSG00000218274.2	RP3-407E4.3	4.27471710604621	4.9579701637584e-07	2.18E-05
ENSG00000171004.13	HS6ST2	4.27137432578115	3.52501248041646e-13	9.36E-11
ENSG00000218048.2	RP3-407E4.4	4.26958457073195	1.29587484915862e-06	4.96E-05
ENSG00000268869.1	ESPNP	4.26645943649806	1.15703750324707e-06	4.49E-05
ENSG00000183010.12	PYCR1	4.19766669732095	2.59595765727462e-13	7.38E-11
ENSG00000151012.9	SLC7A11	4.17223754960131	1.6529992361332e-11	2.70E-09
ENSG00000157399.10	ARSE	4.16276510251982	1.31667738180086e-13	4.20E-11
ENSG00000170373.4	CST1	4.12381468077558	3.1897660667599e-11	4.81E-09
ENSG00000272214.1	AC079602.1	4.0496112949541	4.2066429672949e-23	7.15E-20
ENSG00000227036.2	LINC00511	4.02738764391224	1.18724308771801e-34	7.57E-31
ENSG00000171759.4	PAH	4.01014160516511	1.35403555934932e-06	5.13E-05
ENSG00000164749.7	HNF4G	4.00176987285278	1.48496991978054e-08	1.09E-06
ENSG00000169684.9	CHRNA5	3.98857931124937	1.96057831085844e-07	1.00E-05
ENSG00000263680.2	RP11-57A1.1	3.98375889660543	1.23844239505504e-06	4.77E-05
ENSG00000145632.10	PLK2	-1.00207648356479	3.28185807384707e-06	0.000109
ENSG00000162104.5	ADCY9	-1.00221171660062	1.26449444331421e-06	4.85E-05
ENSG00000226688.2	ENTPD1-AS1	-1.0024324454865	2.03680094485465e-07	1.03E-05
ENSG00000075618.13	FSCN1	-1.00436458767902	0.00089648606571919	0.009503
ENSG00000242539.2	AC007620.3	-1.00675160910508	5.25243305558751e-06	0.000158
ENSG00000198467.9	TPM2	-1.00784578018956	7.33947976563443e-07	3.05E-05
ENSG00000006740.12	ARHGAP44	-1.00942239704406	0.000708238715488472	0.007969
ENSG00000241839.5	PLEKHO2	-1.00981917031941	0.000928866892952743	0.009753
ENSG00000130988.8	RGN	-1.01480601904451	0.000791953098005607	0.008646
ENSG00000114841.13	DNAH1	-1.01531585984466	0.000855071282902077	0.00919
ENSG00000228960.4	OR2A9P	-1.01537940933772	1.09828593100729e-05	0.00029
ENSG00000168038.6	ULK4	-1.01650044740795	0.000193833449680844	0.0029
ENSG00000140937.9	CDH11	-1.01659407098782	9.70987766850637e-06	0.00026
ENSG00000183688.4	FAM101B	-1.01682176345821	0.000391238358288063	0.005063
ENSG00000107796.8	ACTA2	-1.01921016374462	0.000942695339103898	0.009845
ENSG00000143842.10	SOX13	-1.02090346059188	7.23329449106667e-09	5.74E-07
ENSG00000259073.1	FOXN3-AS2	-1.02134920472515	0.000184582947865104	0.002789
ENSG00000116991.6	SIPA1L2	-1.02157488042094	1.77988952133336e-06	6.45E-05
ENSG00000152661.7	GJA1	-1.02167883337485	6.97624874548144e-05	0.001271
ENSG00000033327.8	GAB2	-1.02262940424793	2.10191517409543e-16	1.28E-13
